# Unified total synthesis of the brevianamide alkaloids enabled by chemical investigations into their biosynthesis†

**DOI:** 10.1039/d1sc05801k

**Published:** 2021-12-29

**Authors:** Robert C. Godfrey, Helen E. Jones, Nicholas J. Green, Andrew L. Lawrence

**Affiliations:** EaStCHEM School of Chemistry, University of Edinburgh Joseph Black Building David Brewster Road Edinburgh EH9 3FJ UK a.lawrence@ed.ac.uk

## Abstract

The bicyclo[2.2.2]diazaoctane alkaloids are a vast group of natural products which have been the focus of attention from the scientific community for several decades. This interest stems from their broad range of biological activities, their diverse biosynthetic origins, and their topologically complex structures, which combined make them enticing targets for chemical synthesis. In this article, full details of our synthetic studies into the chemical feasibility of a proposed network of biosynthetic pathways towards the brevianamide family of bicyclo[2.2.2]diazaoctane alkaloids are disclosed. Insights into issues of reactivity and selectivity in the biosynthesis of these structures have aided the development of a unified biomimetic synthetic strategy, which has resulted in the total synthesis of all known bicyclo[2.2.2]diazaoctane brevianamides and the anticipation of an as-yet-undiscovered congener.

## Abstract 2

A divergent biomimetic total synthesis of all known bicyclo[2.2.2]diazaoctane brevianamide alkaloids has been achieved. These synthetic studies have also resulted in the anticipation of an as-yet-undiscovered congener, which we name brevianamide Z.

## Graphical abstract

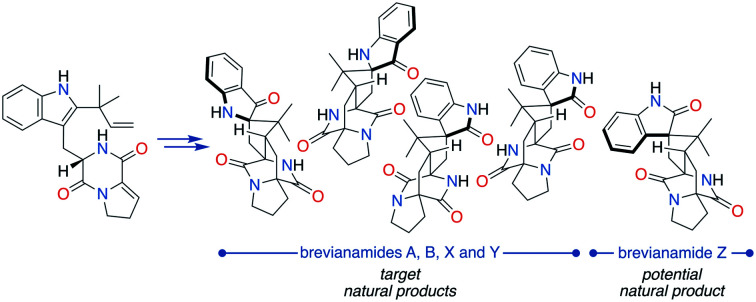

## Introduction

**Fig. 1:**
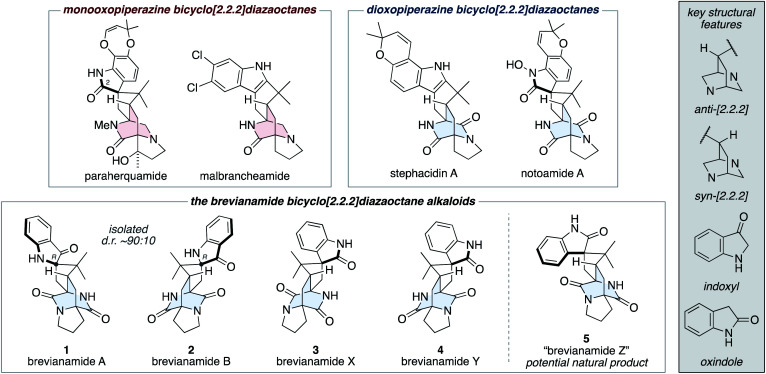
Representative bicyclo[2.2.2]diazaoctane alkaloids, including brevianamides A (1), B (2), X (3), Y (4), and Z (5).

The fungal-derived bicyclo[2.2.2]diazaoctane alkaloids have been the focus of attention from the scientific community for several decades.^1^ They are of significant interest to synthetic chemists as topologically complex targets, which have diverse biosynthetic origins and exhibit a broad range of biological activities. There are two types of biosynthetically distinct bicyclo[2.2.2]diazaoctane alkaloid, the monooxopiperazine structures, which include the paraherquamides^2^ and malbrancheamides;^3^ and the dioxopiperazine structures, which include the stephacidins^4^ and notoamides (Fig. 1).^5^

### The brevianamide alkaloids

The first reported bicyclo[2.2.2]diazaoctane alkaloids were brevianamide A (1) and B (2), which are diastereomeric dioxopiperazine-type structures, isolated by Birch and Wright from the fungus *Penicillium brevicompactum* in 1969 (Fig. 1).^6^ Structurally, they share a common (*R*)-configured spiro-indoxyl stereogenic centre and they possess ‘enantiomeric’ *anti*-configured bicyclo[2.2.2]diazaoctane cores (*anti* refers to the relative configuration of the methine C–H and proximal piperazine nitrogen, see grey box in Fig. 1). They are isolated from the fungus in a consistent diastereomeric ratio of approximately 90 : 10.^7^ In 2017, the closely related natural products, brevianamides X (3) and Y (4) were isolated by Qi and co-workers from the deep-sea-derived *P. brevicompactum* DFFSCS025.^8^ In contrast to brevianamides A (1) and B (2), they contain spiro-oxindole rings and brevianamide X (3) is further differentiated by having a *syn*-configured bicyclo[2.2.2]-diazaoctane core.

We recently reported the first chemical synthesis of brevianamide A (1),^9^ the archetypal bicyclo[2.2.2]diazaoctane alkaloid.^1^ Our synthetic strategy was modelled on a proposed biosynthetic pathway that built upon decades of biosynthetic speculation and investigation. In this article we provide full details of our synthetic endeavors, which have resulted in the development of a unified total synthesis of all known bicyclo[2.2.2]diazaoctane brevianamide alkaloids (1–4) and the anticipation of an as-yet-undiscovered congener (5) (Fig. 1). We also outline experimental investigations into complexity-generating biomimetic cascade reactions and discuss how these relate to recent biosynthetic studies.^10^ Our exploration of the (bio)synthetic relationships between these complex alkaloids has allowed us to identify conditions that favor formation of specific scaffolds from a common intermediate.

### Biological activity

The bicyclo[2.2.2]diazaoctane alkaloids have diverse and often potent biological activities. For example, the stephacidins and notoamides are cytotoxic,^4,5^ the malbrancheamides are calmodulin inhibitors,^3^ and the paraherquamides are anthelmintics.^2^ A modified paraherquamide, derquantel (2-deoxy-paraherquamide), is currently used in combination with the macrocyclic lactone, abamectin, under the trade name STARTECT^®^ to treat sheep with gastrointestinal nematodes.^2^ With regards to the brevianamides, brevianamide A (1) is reported to have potent antifeedant activity against the larvae of the insect pests *Spodoptera frugiperda* (fall armyworm) and *Heliothis virescens* (tobacco budworm),^11^ but no biological activity has yet been reported for brevianamides B (2), X (3) and Y (4). Our synthetic access to practical quantities of brevianamide A (1) is facilitating ongoing studies into its antifeedant properties and we envisage that access to the other brevianamide alkaloids, and synthetic analogues, will similarly enable investigations into their biological profiles.

### Previous synthetic studies

**Scheme 1:**
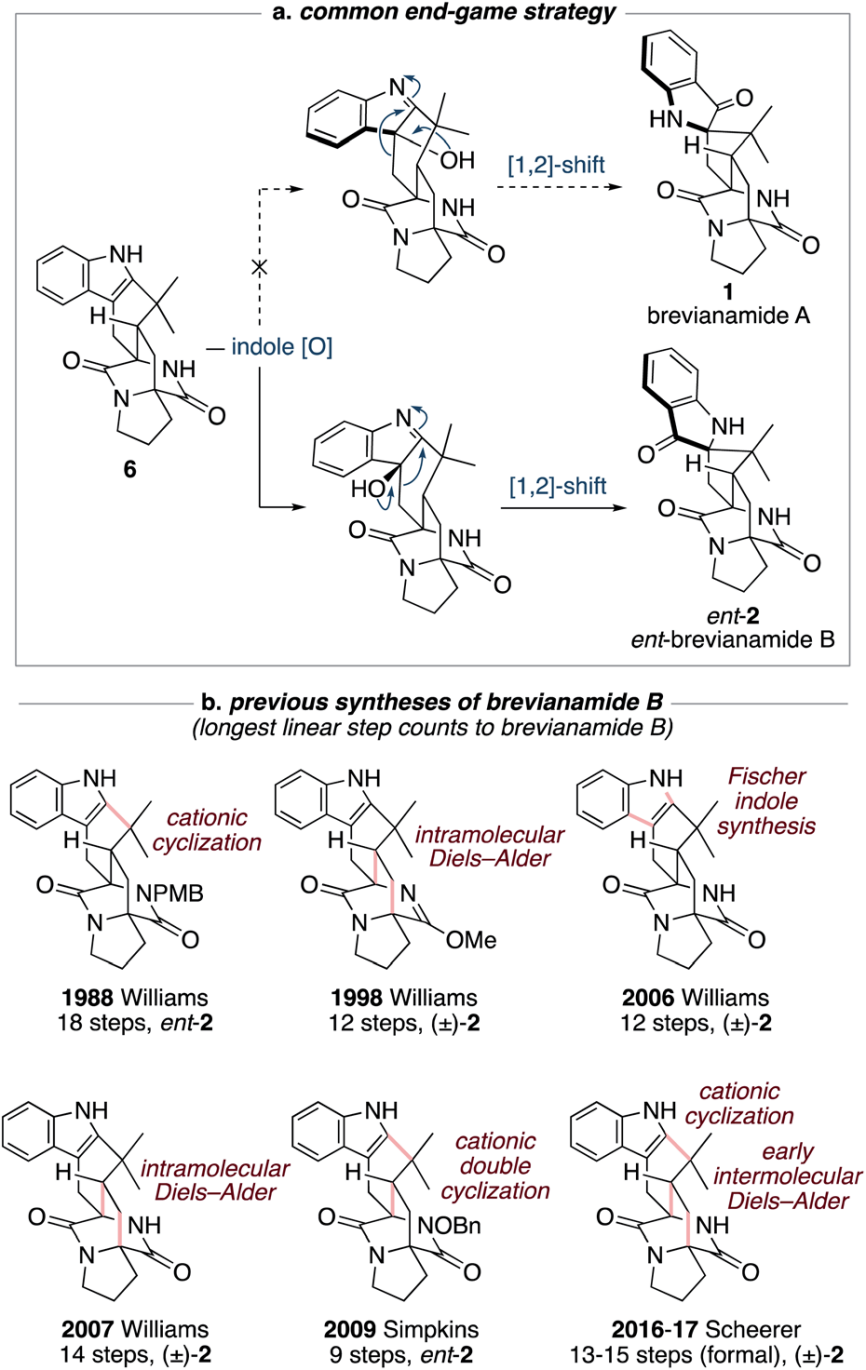
Previous synthetic approaches towards brevianamides A (1) and B (2).

The historical failure to develop a viable synthetic route to brevianamide A (1) is attributable to all previously explored approaches featuring a common and ultimately doomed end-game strategy (Scheme 1a). This strategy hinges on a polycyclic indole intermediate (*e.g.*, 6) undergoing a selective indole-oxidation and [1,2]-shift ring contraction to give brevianamide A (1) (Scheme 1a). Unfortunately, all examples of this type of late-stage indole-oxidation exhibit complete selectivity for oxidation occurring on the sterically more accessible convex face. Following the stereospecific [1,2]-shift this then leads to the unnatural enantiomeric framework of brevianamide B (Scheme 1a).^12^ Thus, although this strategy hasn't provided a route to brevianamide A (1), several syntheses of brevianamide B (2) have been achieved that proceed *via* intermediate 6, or protected analogues thereof (see structures in Scheme 1b).^12^ Most notably, Williams and co-workers have achieved four syntheses of brevianamide B (2). In 1988, they reported the first total synthesis of *ent*-brevianamide B (*ent*-2) using a late-stage cationic cyclization, which resulted in an 18-step total synthesis.^12*a*–*c*^ A decade later they reported a 12-step total synthesis of (±)-brevianamide B (2), in which they utilized a bio-inspired intramolecular Diels–Alder reaction to forge the bicyclo[2.2.2]diazaoctane core, albeit with diastereoselectivity not favoring the desired diastereomer (d.r. 2 : 1, *vide infra*).^12*d*,*e*^ In their third synthesis, Williams and co-workers utilized a late-stage Fischer indole-synthesis to gain access to (±)-brevianamide B (2) in 12 steps.^12*f*^ Finally, in 2007 they reappraised their biomimetic intramolecular Diels–Alder strategy, this time avoiding protection of the lactam functionality, and achieved a 14-step synthesis of (±)-brevianamide B (2).^12*g*,*h*^ The shortest total synthesis of *ent*-brevianamide B (*ent*-2), prior to this present study, was achieved by Simpkins and co-workers in 2009.^12*i*,*j*^ The brevity of their 9-step synthesis can be traced back to an elegant cationic double-cyclization strategy, wherein two new C–C bonds and three new rings are formed in one step. Unfortunately, the diastereoselectivity of this cascade reaction did not favor the required diastereomer (d.r. 4 : 1). In 2016 and 2017, Scheerer and co-workers reported two formal syntheses of (±)-brevianamide B (2), which rely on early intermolecular Diels–Alder reactions to forge the bicyclo[2.2.2]diazaoctane ring system.^12*k*,*l*^ A series of functional group interconversions and a late-stage cationic cyclization, as pioneered by Williams and co-workers in 1988,^12*a*^ result in 13 and 15 step formal syntheses of (±)-brevianamide B (2). In summary, the confluence of previous synthetic approaches on one common end-game strategy (Scheme 1a) has precluded access to brevianamide A (1) but resulted in several syntheses of brevianamide B (2), either in its unnatural enantiomeric form or as a racemate (Scheme 1b).

### Biosynthesis of the brevianamides

**Scheme 2:**
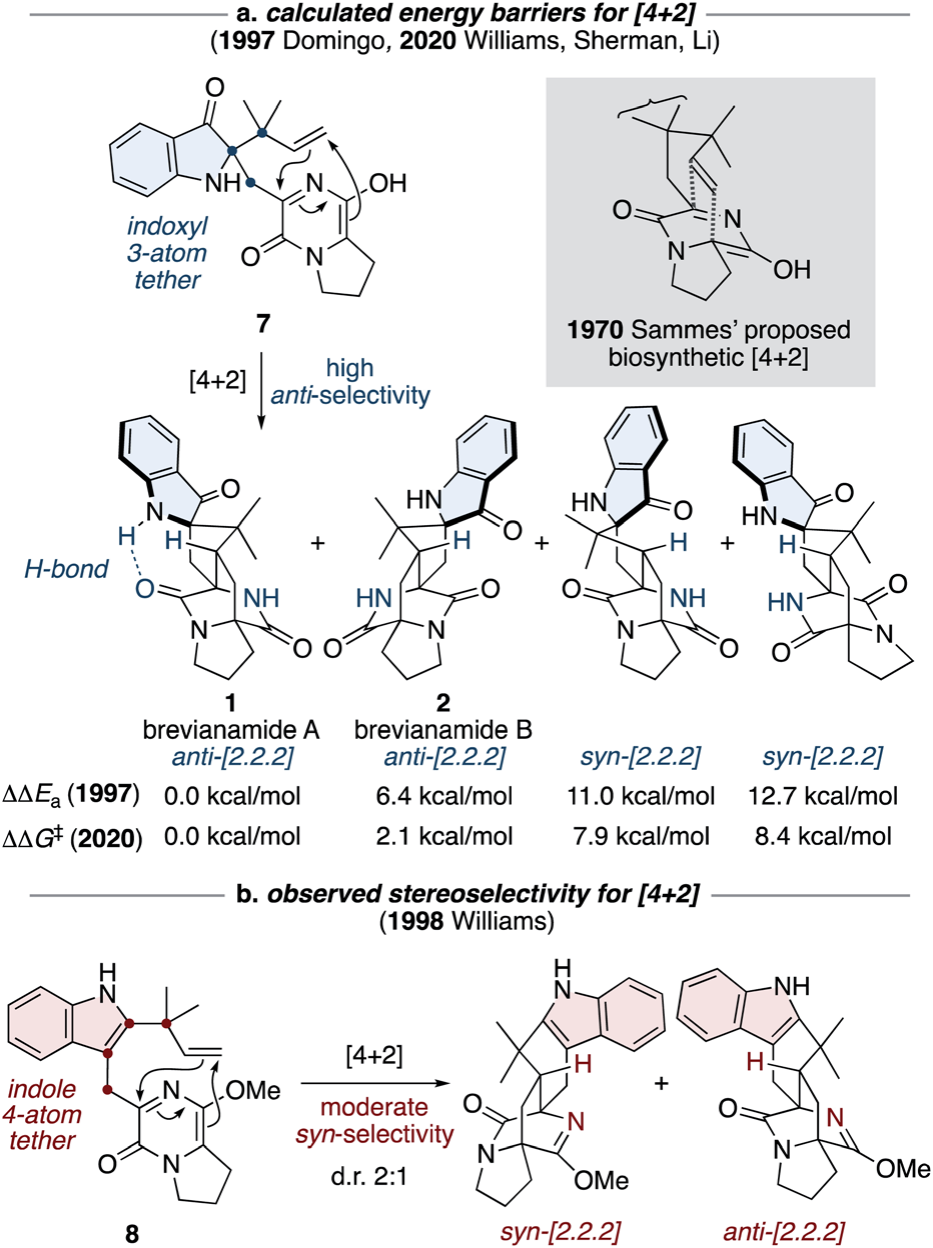
Diastereoselectivity of intramolecular Diels–Alder reactions related to Sammes' biosynthetic proposal.^10,12*d*,*e*,16^

The most notable transformation in the biosynthesis of the brevianamides (1-4) is an intramolecular Diels–Alder reaction, which was originally proposed by Sammes and Porter in 1970 (see grey box in Scheme 2).^13^ This insightful proposal has since been extended to other bicyclo[2.2.2]diazaoctane alkaloid biosyntheses.^1^ Diels–Alderase enzymes have been identified in the biosynthetic gene clusters responsible for the malbrancheamide and paraherquamide monooxopiperazine alkaloids.^14^ However, the involvement of Diels–Alderase enzymes in the biosynthesis of the dioxopiperazine alkaloids has been a topic of debate for decades, particularly with regards to the brevianamides.^1,15^ DFT calculations by Domingo and co-workers in 1997 indicated that for precursor 7, where the azadiene and dienophile are tethered *via* three atoms through an indoxyl ring system (*i.e.*, post indole oxidation), the Diels–Alder reaction is expected to be inherently *anti*-diastereoselective (Scheme 2a).^16^ This was further supported last year in computational studies reported by Williams, Sherman, Li and co-workers (Scheme 2a).^10^ Both studies also revealed that an intramolecular hydrogen bond, between the indoxyl N–H and proximal lactam C=O, can stabilize the transition state for brevianamide A (1), relative to B (2), thus potentially accounting for the greater quantity of brevianamide A (1) isolated from the fungus (d.r. A : B ∼90 : 10).^10,16^ On the other hand, in 1998 Williams and co-workers discovered that the Sammes-type intramolecular Diels–Alder reaction on an unoxidized indole intermediate, 8, exhibits moderate *syn*-diastereoselectivity (Scheme 2b).^12*d*,*e*^ Thus, the necessity of a Diels–Alderase enzyme to control stereoselectivity in the biogenesis of the brevianamides appeared to be dependent on the relative order of the indole-oxidation and Diels–Alder reaction.^17^ Indeed, this point highlights the fact that without experimental data (synthetic, biochemical, or computational) there are numerous plausible permutations of biosynthetic transformations towards these alkaloids which could be, and indeed have been, proposed.^1^

**Scheme 3:**
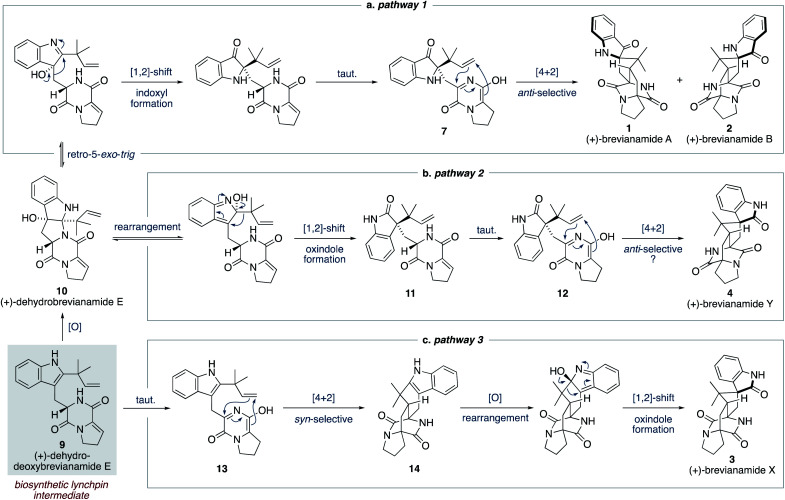
Our proposed biosynthesis of the bicyclo[2.2.2]diazaoctane brevianamides A, B, X and Y (1–4) from dehydrodeoxybrevianamide E (9).

We proposed a modified biosynthetic pathway for brevianamide A (1) in 2020.^9^ Our modified pathway relies on an early indole oxidation followed by a late-stage, substrate controlled, *anti*-diastereoselective Diels–Alder reaction (Scheme 3a).^16^ A key modification in our proposed pathway was the involvement of dehydrodeoxybrevianamide E (9), a natural product isolated from various *Penicillium* and *Aspergillus* fungi.^18^ We invoked this known natural product, 9, as a biosynthetic intermediate because its diketopiperazine ring is already at the correct oxidation level for the late-stage Diels–Alder reaction.^19^ Our pathway begins with diastereoselective indole-oxidation of 9 to give dehydrobrevianamide E (10), which we propose may be an as-yet-undiscovered natural product. Retro-5-*exo-trig* ring opening, followed by stereospecific [1,2]-shift and tautomerization then gives enantiopure azadiene 7 (Scheme 3a). A final Sammes-type Diels–Alder cycloaddition then gives brevianamides A (1) and B (2), which we were confident would proceed with the required *anti*-diastereoselectivity thanks to the DFT calculations reported by Domingo and co-workers.^16^ Our recent success in mimicking this proposed pathway *in vitro* (*vide infra*) led us to expand this biosynthetic speculation to account for the formation of all known bicyclo[2.2.2]diazaoctane brevianamides (1–4).

For brevianamide Y (4), we propose that dehydrobrevianamide E (10) undergoes a different rearrangement to give a thermodynamically more favorable oxindole intermediate 11 (Scheme 3b). This type of rearrangement (hydroxyindolenine to oxindole) is well known and is believed to proceed *via* transient epoxide intermediates.^20^ Subsequent tautomerization of oxindole 11 then gives an enantiopure azadiene intermediate 12, which undergoes a Sammes-type Diels–Alder cycloaddition to give brevianamide Y (4). We set-out to demonstrate the feasibility of this new Diels–Alder reaction and to probe its inherent diastereoselectivity through chemical synthesis.

To account for the formation of the atypical *syn*-configured bicyclo[2.2.2]diazaoctane core of brevianamide X (3) we propose an early Diels–Alder cycloaddition of dehydrodeoxybrevianamide E (9) (Scheme 3c). This is predicated on Williams' observation of inherent *syn*-diastereoselectivity in Diels–Alder reactions with indole oxidation-level substrates (*e.g.*, 8) (Scheme 2b).^12*d*,*e*^ It is reasonable to propose that a Diels–Alderase enzyme might be involved in this pathway as it involves an achiral intermediate, 13.^21^ There are, however, alternative explanations for the isolation of brevianamide X (3) in enantioenriched form. A spontaneous (*i.e.*, non-enzyme mediated) biosynthetic Diels–Alder reaction might be followed by a kinetic resolution of racemic 14 in the subsequent enzymatic oxidation, or semi-pinacol [1,2]-shift.^22^ Alternatively, selective degradation of the unobserved enantiomer might be occurring. Detailed biosynthetic investigations will be required to unequivocally explain the isolation of brevianamide X (3) in enantioenriched form. Nevertheless, we were hopeful that we could establish the chemical feasibility of this pathway through chemical synthesis.

## Results and discussion

### Total synthesis of dehydrodeoxybrevianamide E

**Scheme 4:**
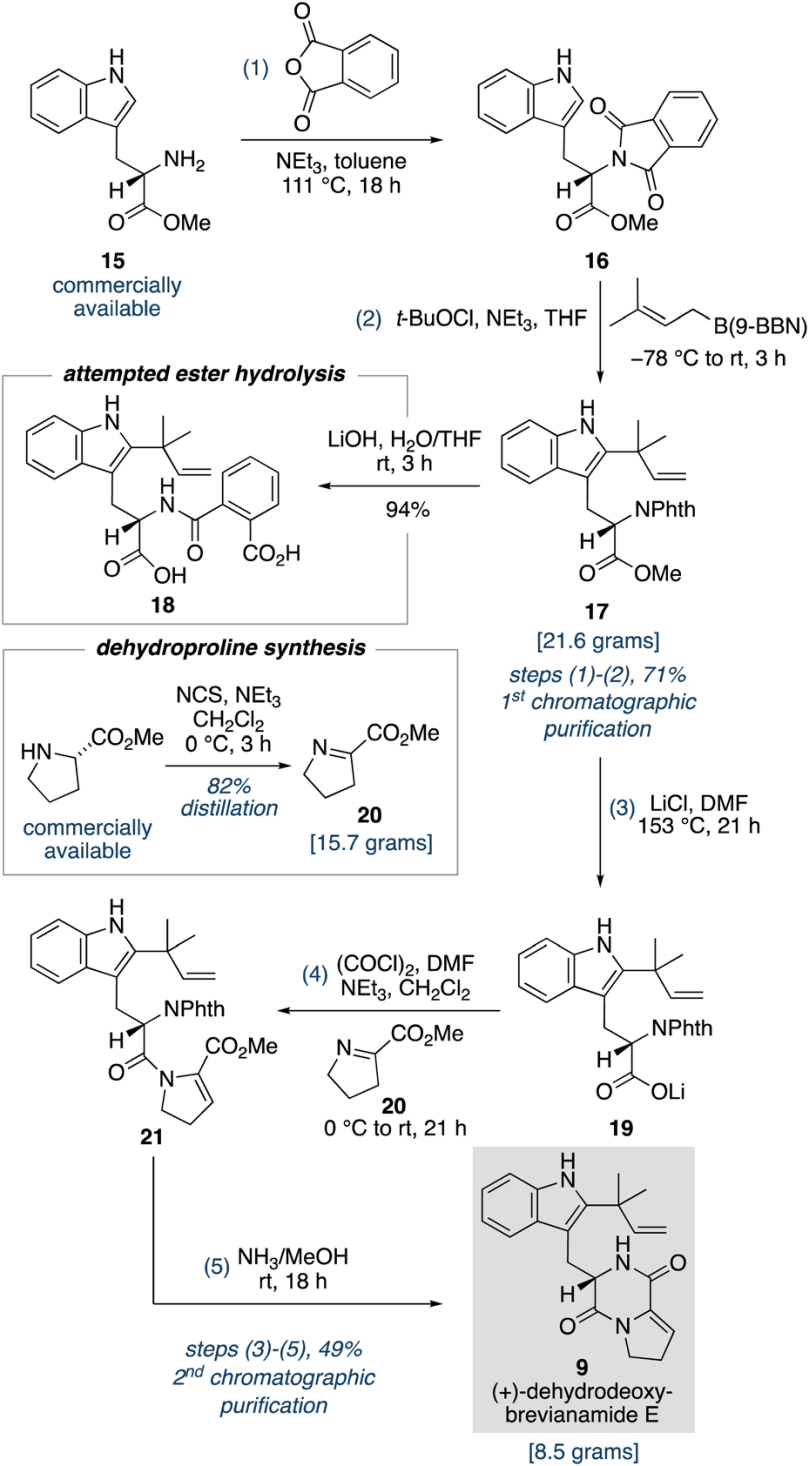
Five-step, gram-scale total synthesis of (+)-dehydrodeoxybrevianamide E (9).

To fully investigate the chemical feasibility of our proposed network of biosynthetic pathways (Scheme 3) we first needed access to our lynchpin (bio)synthetic intermediate, (+)-dehydrodeoxybrevianamide E (9). Phthaloyl protection of l-tryptophan methyl ester 15 was followed by reverse prenylation of the indole-C2 position using Danishefsky's conditions to give intermediate 17 in 71% yield over the two steps (Scheme 4).^23,24^ Hydrolysis of methyl ester 17 resulted in unwanted ring opening of the phthaloyl group to give diacid 18. This presumably explains why a deprotection/reprotection sequence is undertaken in related synthetic endeavours.^24,25^ To avoid these extraneous two steps, we explored the possibility of a Krapcho-type demethylation of methyl ester 17. Use of LiI in EtOAc, as reported by Fisher and Trinkle,^26^ successfully gave the desired product 19. However, we found using LiCl in DMF resulted in a cleaner reaction. Lithium carboxylate 19 was then converted to *N*-acyl enamine 21 through a one-pot acyl chloride formation and imine acylation reaction using dehydroproline 20,^27^ which can be easily accessed in practical quantities from proline methyl ester using *N*-chlorosuccinimide as the oxidant (Scheme 4). Standard phthaloyl deprotection reagents, such as hydrazine, ethylene diamine, methylamine, hydroxylamine, ethanolamine and phenylhydrazine, resulted in unwanted cleavage of the amide bond in intermediate 21. However, the less nucleophilic ammonia in methanol not only deprotected the primary amine but also resulted in spontaneous cyclization to give (+)-dehydrodeoxybrevianamide E (9) in 49% yield over the three steps from methyl ester 17.^28^ Thus, the total synthesis of (+)-dehydrodeoxybrevianamide E (9) has been achieved in a longest linear sequence of 5 steps, in 35% overall yield, and requires just 2 chromatographic purifications (*cf.* previous synthesis: 12 steps, 8% overall yield).^12*g*,*h*^

### Total synthesis of brevianamides A and B

**Scheme 5:**
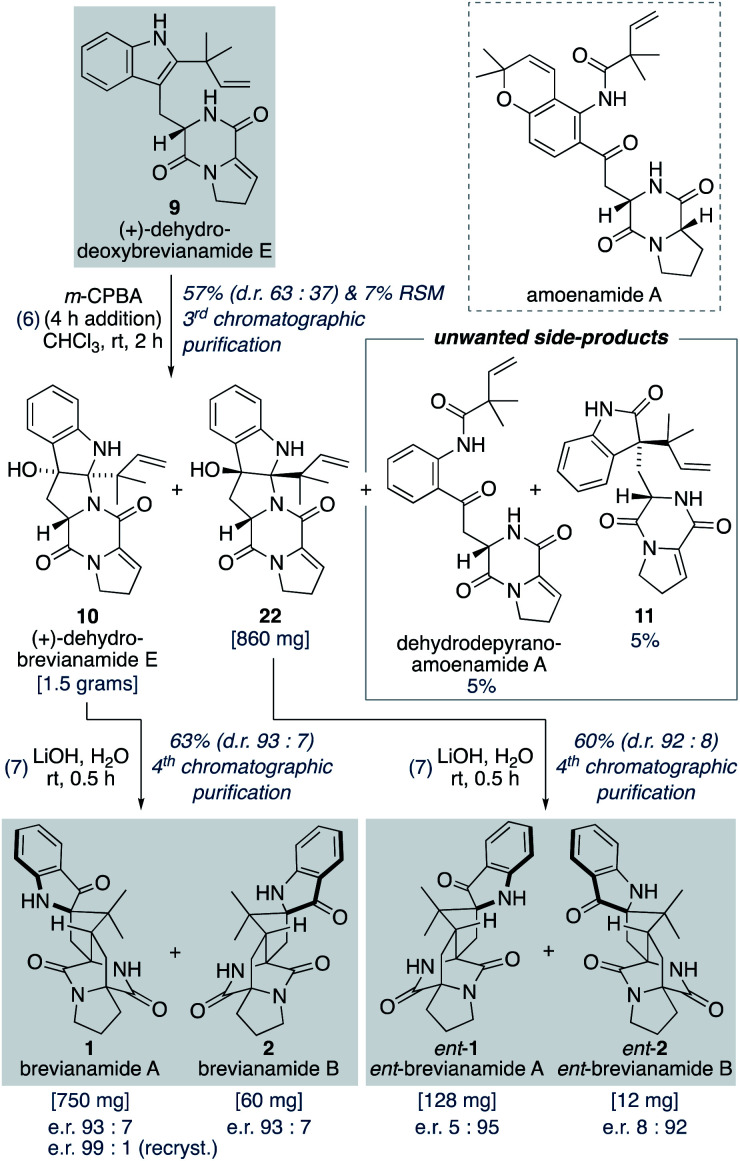
Total synthesis of (+) and (−)-brevianamides A (1) and B (2).

Oxidation of (+)-dehydrodeoxybrevianamide E (9) using a variety of oxidants (for example, peroxy acids, singlet oxygen,^29^ dioxiranes,^24^ and oxaziridines^15*a*^) gave dehydrobrevianamide E (10), alongside diastereomer 22 (Scheme 5). The stereoselectivity of the corresponding biosynthetic indole oxidation is controlled by flavin-dependent monooxygenase enzymes.^1*b*^ Achieving diastereoselectivity using chemical oxidants proved to be very challenging. For example, use of Davis' oxaziridine resulted in a relatively clean reaction but with no diastereoselectivity. The best we could achieve was a d.r. of 63 : 37 (10 : 22) using *m*-CPBA at room temperature, with no appreciable improvement observed at lower temperatures or when using other peroxy acids. Use of an excess of *m*-CPBA, to improve conversion, resulted in the formation of an unwanted side-product, dehydro-depyranoamoenamide A (Scheme 5). This unexpected double oxidation/fragmentation reaction could be of future utility for the total synthesis of amoenamide A, a natural product isolated from *Aspergillus amoenus* NRRL 35600 by Tsukamoto and co-workers in 2017 (Scheme 5).^30^ To suppress this unwanted reaction an equimolar quantity of *m*-CPBA was added slowly to (+)-dehydrodeoxybrevianamide E (9) at room temperature to give dehydrobrevianamide E (10) and diastereomer 22 in 57% combined yield (d.r. 63 : 37). Under these conditions, conversion was satisfactorily high with minimal formation of unwanted side-products; 5% dehydro-depyranoamoenamide A, 5% oxindole 11, and 7% recovered starting material. Synthetic access to both diastereomers 10 and 22 is valuable as it enables the preparation of both the natural-(+) and unnatural-(−) enantiomers of brevianamides A (1) and B (2) (*vide infra*). Exposure of dehydrobrevianamide E (10) to LiOH in water at ambient temperature for 30 min successfully gave the natural (+)-enantiomers of brevianamide A (1) and B (2) in a combined 63% yield. Keeping the reaction time to a minimum and avoiding elevated temperatures was essential to prevent alkaline hydrolysis of the products, as previously observed by Birch and co-workers.^31^ Following only the fourth chromatographic purification in the entire seven-step synthesis, 750 mg of (+)-brevianamide A (1) and 60 mg of (+)-brevianamide B (2) were isolated. Chiral-HPLC analysis revealed both products (1 and 2) were isolated in a 93 : 7 enantiomeric ratio, which is not surprising given many of our synthetic intermediates may have undergone partial racemization. Recrystallization gave (+)-brevianamide A (1) in an enantiomeric ratio of 99 : 1. The unnatural (−)-enantiomers of brevianamide A (*ent*-1) and B (*ent*-2) were similarly accessed by subjecting the minor diastereomer, compound 22, to the same reaction conditions (Scheme 5).

**Scheme 6:**
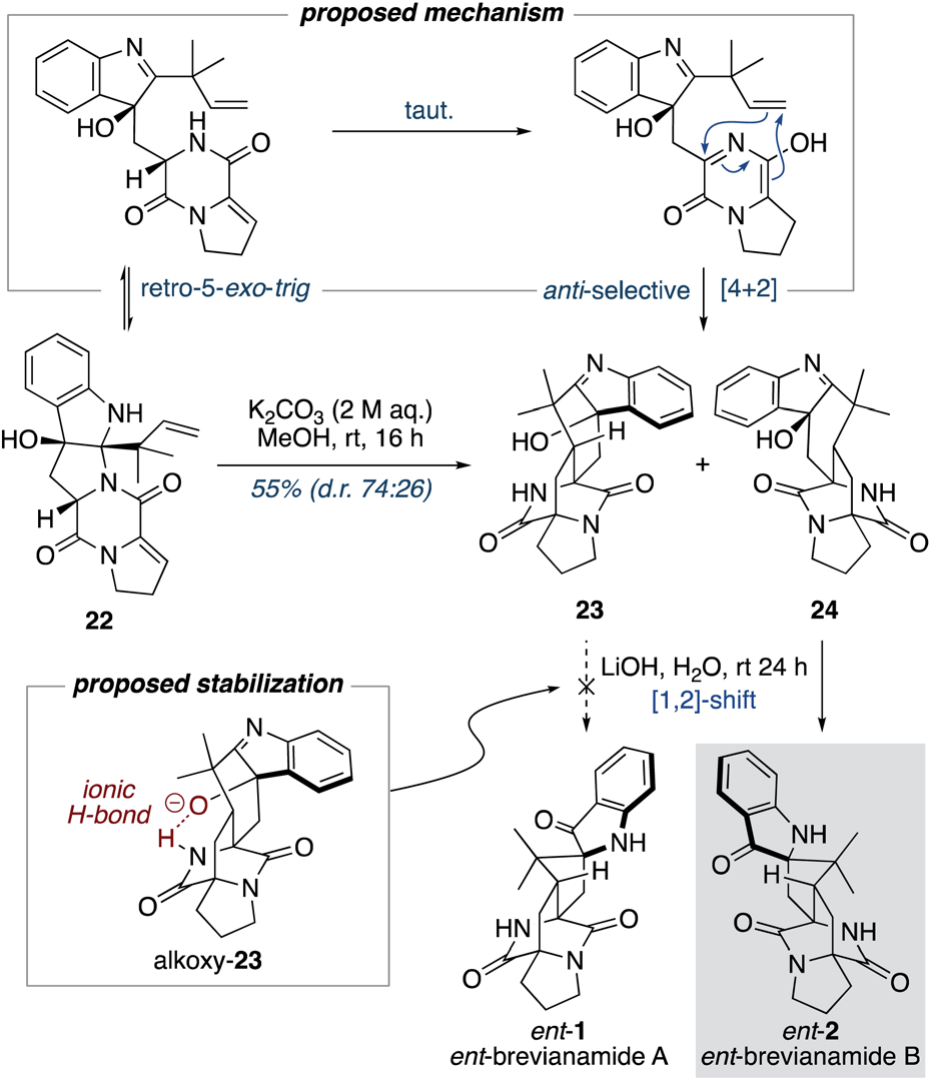
Mechanistic investigations into the final biomimetic cascade reaction.

Williams and co-workers have reported the use of aqueous KOH in methanol for closely related biomimetic reactions.^12*d*,*g*^ For our reaction, however, we found that aqueous KOH in methanol resulted in a lower yield (44%) with the formation of a minor hydroxy-indolenine side-product 23 (11%) (*vide infra*). Use of KOH or NaOH in water also resulted in slightly lower yields. Mechanistic insights into the importance of the reaction conditions for the final cascade reaction (*i.e.*, 10 to 1/2 and 22 to *ent*-1/*ent*-2) were acquired through further investigations using the minor diastereomer 22. It was found that when using a weaker base (aqueous K_2_CO_3_) in methanol two diastereomeric hydroxy-indolenine structures, 23 and 24, could be formed as the major products (Scheme 6). This presumably occurs *via* a domino retro-5-*exo-trig*/tautomerization/Diels–Alder reaction sequence, with the Diels–Alder reaction exhibiting *anti*-diastereoselectivity. These hydroxy-indolenines, 23 and 24, could conceivably serve as intermediates in the synthesis of *ent*-brevianamides A (*ent*-1) and B (*ent*-2) through a final semi-pinacol [1,2]-shift (Scheme 6). We conducted a series of experiments to further probe this mechanistic possibility. Hydroxy-indolenine 23 was subjected to aqueous LiOH conditions and no conversion to *ent*-brevianamide A (*ent*-1) was observed. In contrast, *ent*-brevianamide B (*ent*-2) was formed when hydroxy-indolenine 24 was subjected to aqueous LiOH conditions. We suspect that this stark difference in reactivity might be due to a stabilizing intramolecular ionic hydrogen bond in alkoxy-indolenine 23 (Scheme 6).^32^ Overall, these results indicate that our successful synthesis of brevianamides A (1) and B (2) does not involve hydroxy-indolenine intermediates (23/24). It appears that use of a strong base in water ensures the semi-pinacol [1,2]-shift occurs prior to the Diels–Alder reaction. This reactivity issue is reflected in the recent biosynthetic studies reported by Williams, Sherman, Li and co-workers,^10^ which revealed that the final enzyme in the biogenesis of brevianamides A (1) and B (2) is a semi-pinacolase. Thus, our use of LiOH in the chemical synthesis of brevianamides A (1) and B (2) appears to be serving the same role as the semi-pinacolase enzyme in the biosynthesis.

The close agreement between the diastereoselectivity observed in our biomimetic Diels–Alder reaction (d.r. 93 : 7) and the ratio in which brevianamides A (1) and B (2) are isolated from *P. brevicompactum* (appx. 90 : 10) prompted us to speculate that “this Diels–Alder reaction might be a spontaneous process that occurs naturally without direct enzyme participation”.^9^ Our proposal of a Diels–Alderase-free biosynthesis, based purely on synthetic observations, aligns with the subsequent biosynthetic studies reported by Williams, Sherman, Li and co-workers.^10^ This result underlines the important role synthetic studies can have for our understanding of reactivity and selectivity in biosynthetic pathways.

During the preparation of this manuscript, a second total synthesis of brevianamide A (1) was reported by Smith and Xu.^33^ In their synthesis, they also relied on an inherently *anti*-diastereoselective final Diels–Alder reaction to complete an impressive seven-step synthesis of brevianamide A (1).

### Total synthesis of brevianamide Y

**Scheme 7:**
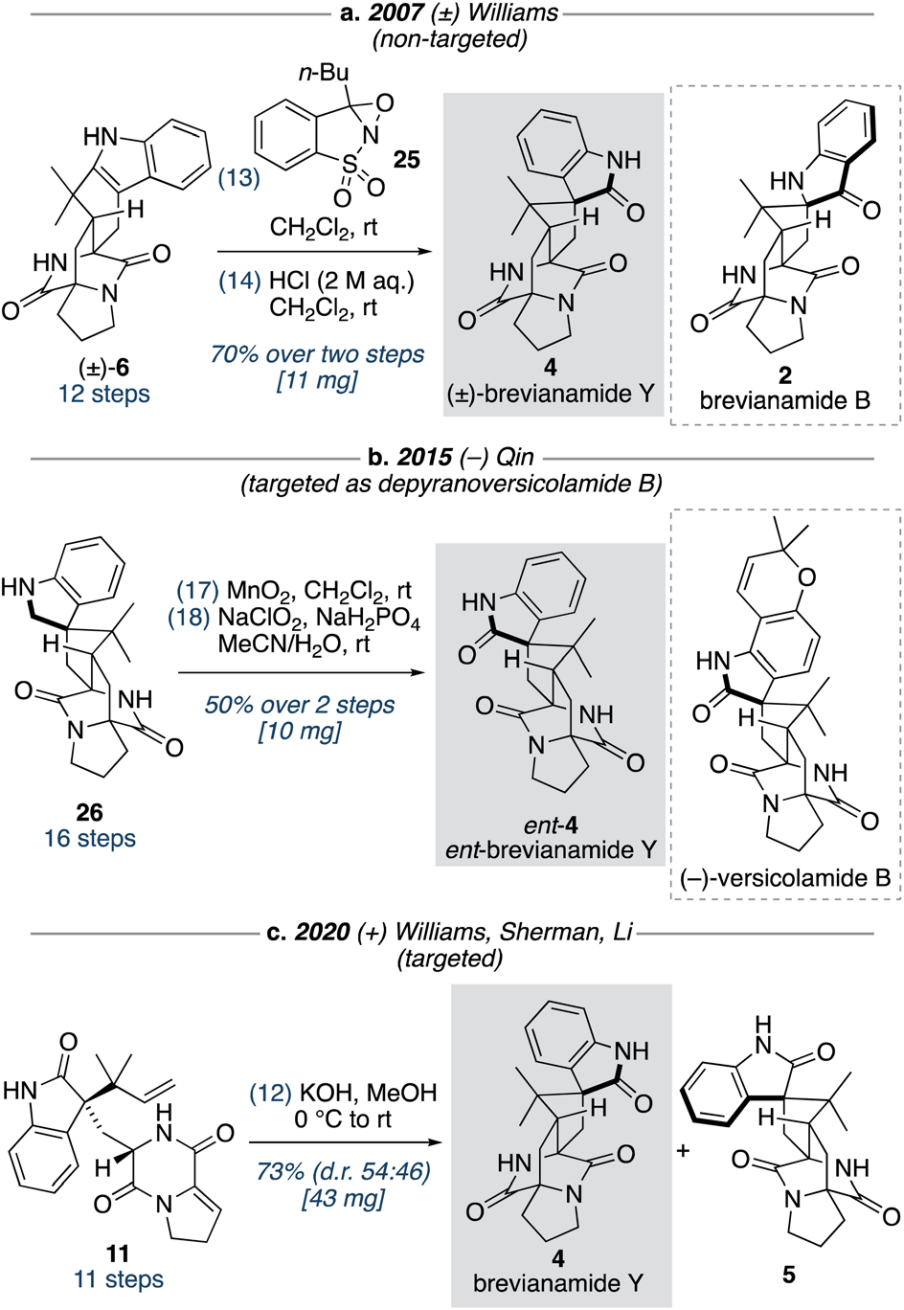
Previous synthetic approaches towards brevianamide Y (4).

Although brevianamide Y (4) was only identified as a natural product in 2017,^8^ it was prepared in racemic-form a decade earlier by Williams and co-workers in a 14-step, non-targeted synthesis (Scheme 7a).^12*g*,*h*^ During a screen of conditions to oxidize indole 6 to give (±)-brevianamide B (2), Williams found that using oxaziridine 25 followed by treatment with 2 M HCl, to facilitate the hydroxyindolenine to oxindole rearrangement, gave exclusive formation of oxindole 4 (*i.e.*, (±)-brevianamide Y). In 2015, Qin and co-workers reported a targeted 18-step synthesis of the unnatural (−)-enantiomer of brevianamide Y (*ent*-4), as a simplified analogue of the related natural product, (−)-versicolamide B (Scheme 7b).^34^ An indoline intermediate 26, which was accessed through Diels–Alder chemistry, was oxidized over two steps to give *ent*-4. In 2020, as part of their detailed biosynthetic studies, Williams, Sherman, Li and co-workers achieved a 12-step synthesis of brevianamide Y (4) (Scheme 7c).^10^ This targeted synthesis involved a biomimetic Diels–Alder reaction of oxindole 11, which aligns with our biosynthetic speculation (Scheme 3b). We envisaged that an extension of our synthetic strategy could provide an even shorter route to brevianamide Y (4).

**Scheme 8:**
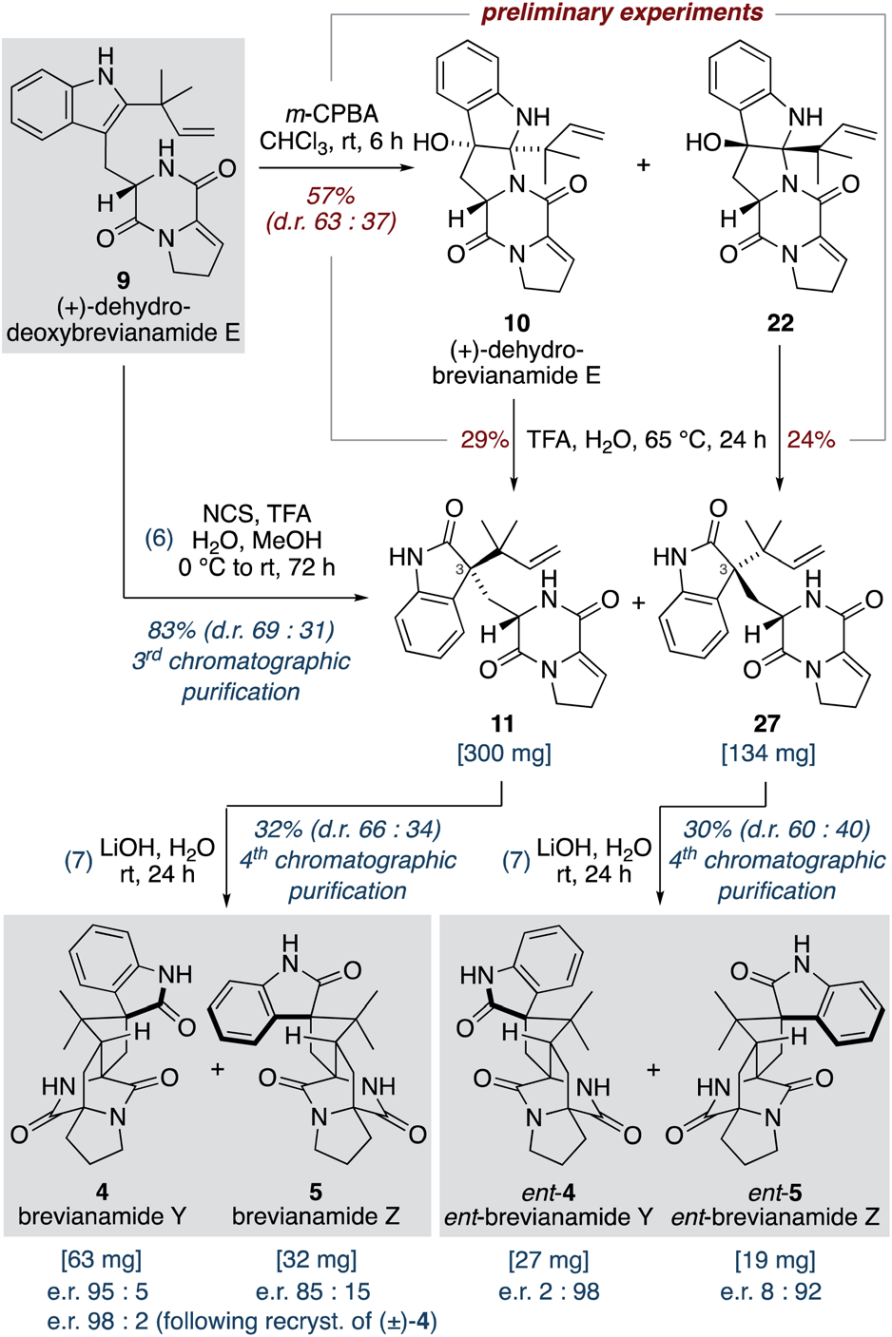
Total synthesis of (+) and (−)-brevianamides Y (4) and Z (5).

Our studies commenced with investigating the rearrangement of (+)-dehydrobrevianamide E (10) to oxindole 11 (Scheme 8, preliminary experiments). Under acidic conditions we were pleased to observe formation of the desired oxindole 11, albeit in low yield. Similarly, we were able to transform the minor diastereomer 22 into the corresponding oxindole 27. However, we had previously observed direct formation of oxindole 11 as a minor side-product when oxidizing (+)-dehydrodeoxybrevianamide E (9) with *m*-CPBA (Scheme 5). Through further experimentation with different oxidants and acidic additives, we found that use of *N*-chlorosuccinimide and aqueous trifluoroacetic acid in methanol converted (+)-dehydrodeoxybrevianamide E (9) directly into oxindoles 11 and 27 in excellent yield and with slightly improved diastereoselectivity (d.r. 69 : 31). Reactions conducted in chloroform gave less clean reactions, and the use of *N*-iodosuccinimide gave unreliable results. Access to both diastereomers, 11 and 27, was again fortuitous as it enables access to both enantiomers of the target molecule, brevianamide Y (4). When the major oxindole 11 was subjected to LiOH in water at ambient temperature for 24 h (+)-brevianamide Y (4) was formed in 32% combined yield alongside a minor diastereomer 5, which we herein name (+)-brevianamide Z (Scheme 8). Thus, our results alongside the synthetic results obtained by Williams, Sherman, Li and co-workers (Scheme 7c),^10^ demonstrate that the Sammes-type Diels–Alder reaction of oxindole 12 proceeds with the same inherent *anti*-diastereoselectivity as indoxyl 7 (Scheme 3a/b). The diastereomeric relationship between brevianamide Y (4) and Z (5) is similar to that which exists between brevianamide A (1) and B (2). That is, they share a common spiro-stereogenic centre and have ‘enantiomeric’ bicyclo[2.2.2]diazaoctane cores. Thus, we propose that (+)-brevianamide Z (5) is an as-yet-undiscovered natural product, worthy of directed isolation efforts. Indeed, if (+)-brevianamide Z (5) is later identified as a minor natural product isolated alongside (+)-brevianamide Y (4), akin to the isolation of B (2) alongside A (1), it would lend support to our biosynthetic proposal and suggest that the biosynthetic Diels–Alder reaction might also be a spontaneous process that occurs without direct enzyme participation. The comparatively lower yield for the final biomimetic transformation towards brevianamides Y (4) and Z (5) (32% yield; Scheme 8) compared to that for brevianamides A (1) and B (2) (63% yield; Scheme 5) is attributable to nonspecific decomposition and associated difficulties encountered with chromatographic purification. The unnatural (−)-enantiomers of brevianamide Y (*ent*-4) and Z (*ent*-5) were accessed under identical conditions from the minor oxindole 27. Again, we were not surprised to discover that some erosion of enatiopurity had occurred during these syntheses.^35^ We discovered that the enantiopurity of (+)-brevianamide Y (4) could be improved through selective crystallization and removal of the racemate. In summary, through our biomimetic studies we have developed a 7-step total synthesis of (+)-brevianamide Y (4), proceeding in 4.1% overall yield and requiring just 4 chromatographic purifications.

### Total synthesis of (±)-brevianamide X

**Scheme 9:**
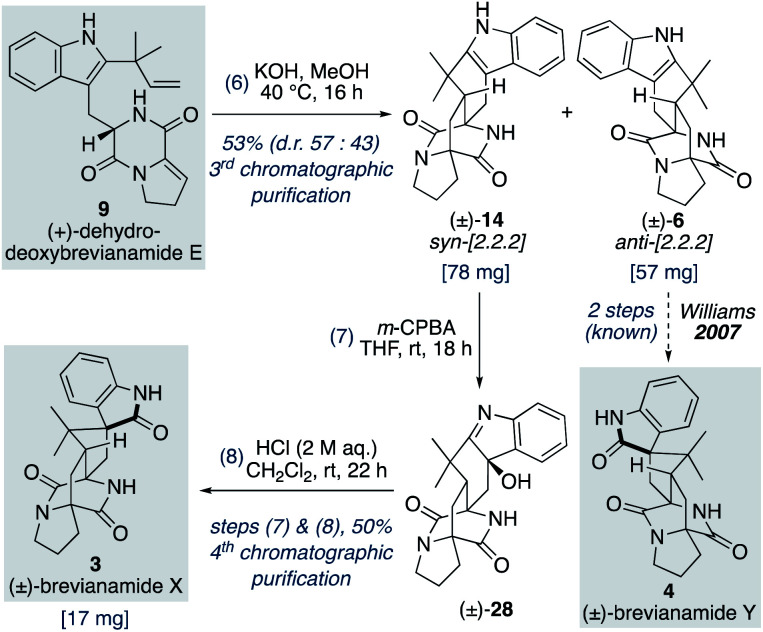
Total synthesis of (±)-brevianamide X (3).

We found that for the Diels–Alder cyclization of (+)-dehydrodeoxybrevianamide E (9) Williams' conditions of methanolic KOH gave a better result than our aqueous LiOH conditions (Scheme 9).^12^ Racemic *syn*- and *anti*-configured bicyclo[2.2.2]diazaoctane products, 14 and 6, were isolated in 53% yield with modest *syn*-diastereoselectivity (d.r. 57 : 43). The major diastereomer 14 was then oxidized with *m*-CPBA to give exclusive formation of hydroxyindolenine 28. This crude material, 28, was then immediately treated to acidic conditions to promote the hydroxyindolenine-to-oxindole rearrangement to give (±)-brevianamide X (3) in 50% yield over the two steps. Thus, we have achieved the total synthesis of (±)-brevianamide X (3) in 8 steps and 5.2% overall yield, with just 4 chromatographic purifications required. In 2020, Williams, Sherman, Li and co-workers reported a 12-step synthesis of brevianamide X (3),^10^ which utilized the same end-game strategy. Our attempts to access brevianamide X (3) in enantioenriched form *via* kinetic resolution of indole 14, using chiral enantiopure oxidants, have so far failed. Investigations into how (+)-brevianamide X (3) is biosynthesized in enantioenriched form will hopefully provide further inspiration for the development of a chemical synthesis of enantioenriched material.

## Conclusions

By undertaking a curiosity-driven study to probe the chemical feasibility of several proposed biosynthetic pathways, we have developed a unified synthetic strategy to the bicyclo[2.2.2]diazaoctane brevianamide alkaloids. All our syntheses are either the first and/or shortest reported total syntheses of the target structures, thus demonstrating the enabling power of the biomimetic approach in natural product synthesis. We have also synthesized an as-yet-undiscovered congener, (+)-brevianamide Z (5), which we hope will help direct future isolation studies. Work is ongoing in our laboratories to now further expand this biomimetic strategy to access other bicyclo[2.2.2]diazaoctane alkaloids in similarly step-economical syntheses.

## Data availability

All experimental procedures and spectral data are available in the ESI.†

## Author contributions

The manuscript was written through contributions of all authors. R. C. G. and H. E. J. contributed equally.

## Conflicts of interest

There are no conflicts to declare.

## Supplementary Material

SC-013-D1SC05801K-s001
