# Supplementary material for: Unified total synthesis of the brevianamide alkaloids enabled by chemical investigations into their biosynthesis
Source: Chem Sci. 2021 Dec 29;13(5):1313–22. doi: 10.1039/d1sc05801k (PMC8809396; doi:10.1039/d1sc05801k)

Electronic Supplementary Material (ESI) for Chemical Science.
This journal is © The Royal Society of Chemistry 2021

SUPPORTING INFORMATION

Unified Total Synthesis of the Brevianamide Alkaloids Enabled by Chemical Investigations into their Biosynthesis.

Robert C. Godfrey, Helen E. Jones, Nicholas J. Green, Andrew L. Lawrence*

EaStCHEM School of Chemistry, University of Edinburgh, Joseph Black Building, David Brewster Road, Edinburgh, EH9 3FJ, U.K.

Supporting Information – Table of Contents

1 Experimental Conditions 6
1.1 Experimental Procedure for Compound **16** 7
1.2 Experimental Procedure for Compound **17** 8
1.3 Experimental Procedure for Compound **18** 9
1.4 Experimental Procedure for Compound **19** 10
1.5 Experimental Procedure for Compound **21** 11
1.6 Experimental Procedure for (+)-dehydrodeoxy-brevianamide E (**9**)............................13
1.7 Experimental Procedure for (+)-dehydro-brevianamide E (**10**) and Compound **22**......14
1.8 Experimental Procedure for (+)-Brevianamide A (**1**) and (–)-Brevianamide B (**2**) 17
1.9 Experimental Procedure for (–)-Brevianamide A (*ent*-**1**) and B (*ent*-**2**)........................20
1.10 Experimental Procedure for Compound **20** 22
1.11 Experimental Procedure for *B*-Prenyl-9-BBN 23
1.12 Experimental Procedure for *t*-BuOCl 25
1.13 Experimental Procedure for Compounds **23** and **24** 26
1.14 Solvent Screen with LiOH and Compound **22** 28
1.15 Solvent Screen with K_2CO_3 and Compound **22** 29
1.16 Solvent Screen with LiOH and Compound **23** 30
1.18 Solvent Screen with LiOH and (–)-brevianamide A (*ent*-**1**)..32
1.19 Experimental Procedure for Compounds **11** and **27** 33
1.20 Experimental Procedure for (+)-Brevianamide Y (**4**) and (+)-Brevianamide Z (**5**) 35
1.21 Experimental Procedure for (–)-Brevianamide Y (*ent*-**4**) and Z (*ent*-**5**)......................37
1.22 Comparison of ^{1}H and ^{13}C NMR Data for Synthetic/Natural Brevianamide Y (**4**).....38
1.23 Experimental Procedure for Compounds (±)-**14** and (±)-**6**..40
1.24 Experimental Procedure for (±)-Brevianamide X (**3**) 42
1.25 Comparison of ^{1}H and ^{13}C NMR Data for Synthetic/Natural Brevianamide X (**3**).....44
2 Chiral HPLC Chromatograms..46
2.1 Chiral HPLC Chromatograms of Brevianamide A (**1**) 46
2.2 Chiral HPLC Chromatograms of Brevianamide B (**2**) 48
2.3 Chiral HPLC Chromatograms of Brevianamide Y (**4**) 50
2.4 Chiral HPLC Chromatograms of Brevianamide Z (**5**)..53
3 NMR Spectra 55
3.1 ^{1}H NMR Spectrum of Compound **16** (500 MHz, $CDCl_3$) 55
3.2 ^{13}C NMR Spectrum of Compound **16** (126 MHz, $CDCl_3$) 55
3.3 ^{1}H NMR Spectrum of Compound **17** (600 MHz, $CDCl_3$) 56
3.4 ^{13}C NMR Spectrum of Compound **17** (151 MHz, $CDCl_3$) 56

3.5 ^{1}H NMR Spectrum of Compound **18** (500 MHz, CD_3OD) 57
3.6 ^{13}C NMR Spectrum of Compound **18** (126 MHz, CD_3OD) 57
3.7 ^{1}H NMR Spectrum of Compound **19** (500 MHz, CD_3OD) 58
3.8 ^{13}C NMR Spectrum of Compound **19** (126 MHz, CD_3OD) 58
3.9 ^{1}H NMR Spectrum of Compound **21** (500 MHz, CD_2Cl_2) 59
3.10 ^{13}C NMR Spectrum of Compound **21** (151 MHz, CD_2Cl_2) 59
3.11 ^{1}H NMR Spectrum of (+)-dehydrodeoxy-brevianamide E (**9**) (500 MHz, $CDCl_3$) 60
3.12 ^{13}C NMR Spectrum of (+)-dehydrodeoxy-brevianamide E (**9**) (126 MHz, $CDCl_3$) ... 60
3.13 ^{1}H NMR Spectrum of (+)-dehydro-brevianamide E (**10**) (500 MHz, $CDCl_3$) 61
3.14 ^{13}C NMR Spectrum of (+)-dehydro-brevianamide E (**10**) (126 MHz, $CDCl_3$) 61
3.15 ^{1}H NMR Spectrum of Compound **22** (500 MHz, $CDCl_3$) 62
3.16 ^{13}C NMR Spectrum of Compound **22** (126 MHz, $CDCl_3$) 62
3.17 ^{1}H NMR Spectrum of Compound **22** (500 MHz, $(CD_3)_2SO$) 63
3.18 ^{13}C NMR Spectrum of Compound **22** (126 MHz, $(CD_3)_2SO$) 63
3.19 ^{1}H NMR Spectrum of dehydro-depyranoamoenamide A (601 MHz, $CDCl_3$) 64
3.20 ^{13}C NMR Spectrum of dehydro-depyranoamoenamide A (151 MHz, $CDCl_3$) 64
3.21 ^{1}H NMR Spectrum of Brevianamide A (**1**) (500 MHz, $CDCl_3$, ~3.0 mg/mL) 65
3.22 ^{13}C NMR Spectrum of Brevianamide A (**1**) (126 MHz, $CDCl_3$, ~3.0 mg/mL) 65
3.23 ^{1}H-^{1}H COSY Spectrum of Brevianamide A (**1**) ($CDCl_3$) 66
3.24 ^{1}H-^{13}C HSQC Spectrum of Brevianamide A (**1**) ($CDCl_3$) 66
3.25 ^{1}H-^{13}C HMBC Spectrum of Brevianamide A (**1**) ($CDCl_3$) 67
3.26 ^{1}H-^{1}H NOESY Spectrum of Brevianamide A (**1**) ($CDCl_3$) 67
3.27 ^{1}H NMR Spectra of Brevianamide A (**1**) (500 MHz, $CDCl_3$ – conc. dependence) 68
3.28 ^{1}H NMR Spectrum of Brevianamide A (**1**) (500 MHz, $(CD_3)_2SO$) 69
3.29 ^{13}C NMR Spectrum of Brevianamide A (**1**) (126 MHz, $(CD_3)_2SO$) 69
3.30 ^{1}H-^{1}H COSY Spectrum of Brevianamide A (**1**) ($(CD_3)_2SO$) 70
3.31 ^{1}H-^{13}C HSQC Spectrum of Brevianamide A (**1**) ($(CD_3)_2SO$) 70
3.32 ^{1}H-^{13}C HMBC Spectrum of Brevianamide A (**1**) ($(CD_3)_2SO$) 71
3.33 ^{1}H-^{1}H NOESY Spectrum of Brevianamide A (**1**) ($(CD_3)_2SO$) 71
3.34 ^{1}H NMR Spectrum of Brevianamide B (**2**) (500 MHz, $CDCl_3$ 72
3.35 ^{13}C NMR Spectrum of Brevianamide B (**2**) (126 MHz, $CDCl_3$) 72
3.36 ^{1}H NMR Spectrum of Brevianamide B (**2**) (500 MHz, $(CD_3)_2SO$) 73
3.37 ^{13}C NMR Spectrum of Brevianamide B (**2**) (126 MHz, $(CD_3)_2SO$) 73
3.38 ^{1}H-^{1}H COSY Spectrum of Brevianamide B (**2**) ($(CD_3)_2SO$) 74
3.39 ^{1}H-^{13}C HSQC Spectrum of Brevianamide B (**2**) ($(CD_3)_2SO$) 74
3.40 ^{1}H-^{13}C HMBC Spectrum of Brevianamide B (**2**) ($(CD_3)_2SO$) 75
3.41 ^{1}H-^{1}H NOESY Spectrum of Brevianamide B (**2**) ($(CD_3)_2SO$) 75

3.42 ^{1}H NMR Spectrum of Compound **20** (500 MHz, $CDCl_3$) 76
3.43 ^{13}C NMR Spectrum of Compound **20** (126 MHz, $CDCl_3$) 76
3.44 ^{1}H NMR Spectrum of 3-Chloro-3-methyl-1-butyne (500 MHz, $CDCl_3$) 77
3.45 ^{13}C NMR Spectrum of 3-Chloro-3-methyl-1-butyne (101 MHz, $CDCl_3$) 77
3.46 ^{1}H NMR Spectrum of 1,1-Dimethylallene (500 MHz, $CDCl_3$) 78
3.47 ^{13}C NMR Spectrum of 1,1-Dimethylallene (151 MHz, $CDCl_3$) 78
3.48 ^{1}H NMR Spectrum of *t*-BuOCl (500 MHz, $CDCl_3$) 79
3.49 ^{13}C NMR Spectrum of *t*-BuOCl (126 MHz, $CDCl_3$) 79
3.50 ^{1}H NMR Spectrum of Compound **23** (500 MHz, $CDCl_3$) 80
3.51 ^{13}C NMR Spectrum of Compound **23** (126 MHz, $CDCl_3$) 80
3.52 ^{1}H NMR Spectrum of Compound **23** (500 MHz, $(CD_3)_2SO$) 81
3.53 ^{1}H NMR Spectrum of Compound **24** (500 MHz, $CDCl_3$) 82
3.54 ^{13}C NMR Spectrum of Compound **24** (126 MHz, $CDCl_3$) 82
3.55 ^{1}H NMR Spectrum of Compound **24** (500 MHz, $(CD_3)_2SO$) 83
3.56 ^{13}C NMR Spectrum of Compound **24** (126 MHz, $(CD_3)_2SO$) 83
3.57 ^{1}H-^{1}H COSY Spectrum of Compound **24** ($CDCl_3$) 84
3.58 ^{1}H-^{13}C HSQC Spectrum of Compound **24** ($CDCl_3$) 84
3.59 ^{1}H-^{13}C HMBC Spectrum of Compound **24** ($CDCl_3$) 85
3.60 ^{1}H-^{1}H NOESY Spectrum of Compound **24** ($CDCl_3$) 85
3.61 ^{1}H NMR Spectrum of Compound **11** (500 MHz, $CDCl_3$) 86
3.62 ^{13}C NMR Spectrum of Compound **11** (126 MHz, $CDCl_3$) 86
3.63 ^{1}H-^{1}H COSY Spectrum of Compound **11** ($CDCl_3$) 87
3.64 ^{1}H-^{13}C HSQC Spectrum of Compound **11** ($CDCl_3$) 87
3.65 ^{1}H-^{13}C HMBC Spectrum of Compound **11** ($CDCl_3$) 88
3.66 ^{1}H-^{1}H NOESY Spectrum of Compound **11** ($CDCl_3$) 88
3.67 ^{1}H NMR Spectrum of Compound **27** (500 MHz, $CDCl_3$) 89
3.68 ^{13}C NMR Spectrum of Compound **27** (126 MHz, $CDCl_3$) 89
3.69 ^{1}H-^{1}H COSY Spectrum of Compound **27** ($CDCl_3$) 90
3.70 ^{1}H-^{13}C HSQC Spectrum of Compound **27** ($CDCl_3$) 90
3.71 ^{1}H-^{13}C HMBC Spectrum of Compound **27** ($CDCl_3$) 91
3.72 ^{1}H-^{1}H NOESY Spectrum of Compound **27** ($CDCl_3$) 91
3.73 ^{1}H NMR Spectrum of Brevianamide Y (**4**) (500 MHz, $(CD_3)_2SO$) 92
3.74 ^{13}C NMR Spectrum of Brevianamide Y (**4**) (126 MHz, $(CD_3)_2SO$) 92
3.75 ^{1}H-^{1}H COSY Spectrum of Brevianamide Y (**4**) ($(CD_3)_2SO$) 93
3.76 ^{1}H-^{13}C HSQC Spectrum of Brevianamide Y (**4**) ($(CD_3)_2SO$) 93
3.77 ^{1}H-^{13}C HMBC Spectrum of Brevianamide Y (**4**) ($(CD_3)_2SO$) 94
3.78 ^{1}H-^{1}H NOESY Spectrum of Brevianamide Y (**4**) ($(CD_3)_2SO$) 94

3.79 ^{1}H NMR Spectrum of Brevianamide Z (**5**) (500 MHz, $(CD_3)_2SO$) 95
3.80 ^{13}C NMR Spectrum of Brevianamide Z (**5**) (126 MHz, $(CD_3)_2SO$) 95
3.81 ^{1}H-^{1}H COSY Spectrum of Brevianamide Z (**5**) ($(CD_3)_2SO$) 96
3.82 ^{1}H-^{13}C HSQC Spectrum of Brevianamide Z (**5**) ($(CD_3)_2SO$) 96
3.83 ^{1}H-^{13}C HMBC Spectrum of Brevianamide Z (**5**) ($(CD_3)_2SO$) 97
3.84 ^{1}H-^{1}H NOESY Spectrum of Brevianamide Z (**5**) ($(CD_3)_2SO$)....................................97
3.85 ^{1}H NMR Spectrum of Compound (±)-**14** (500 MHz, $CDCl_3$) 98
3.86 ^{13}C NMR Spectrum of Compound (±)-**14** (126 MHz, $CDCl_3$)....................................98
3.87 ^{1}H NMR Spectrum of Compound (±)-**6** (500 MHz, $CDCl_3$) 99
3.88 ^{13}C NMR Spectrum of Compound (±)-**6** (126 MHz, $CDCl_3$) 99
3.89 ^{1}H NMR Spectrum of Brevianamide X (**3**) (500 MHz, $(CD_3)_2SO$)..........................100
3.90 ^{13}C NMR Spectrum of Brevianamide X (**3**) (126 MHz, $(CD_3)_2SO$)..........................100
3.91 ^{1}H-^{1}H COSY Spectrum of Brevianamide X (**3**) ($(CD_3)_2SO$).....................................101
3.92 ^{1}H-^{13}C HSQC Spectrum of Brevianamide X (**3**) ($(CD_3)_2SO$)...................................101
3.93 ^{1}H-^{13}C HMBC Spectrum of Brevianamide X (**3**) ($(CD_3)_2SO$)..................................102
3.94 ^{1}H-^{1}H NOESY Spectrum of Brevianamide X (**3**) ($(CD_3)_2SO$) 102

1 Experimental Conditions

NMR Spectroscopy: ^{1}H NMR spectra were recorded at 600 MHz on a Bruker 600 spectrometer with a AVANCE 3HD console, at 500 MHz on Ascend 500 spectrometers with AVANCE 3 and AVANCE 3HD consoles, and at 400 MHz on a Bruker 400 spectrometer with AVANCE 3 console. Residual solvent peaks were used as an internal reference for ^{1}H NMR spectra ($CDCl_3$ δ 7.26 ppm, CD_3OD δ 3.31 ppm, CD_2Cl_2 δ 5.32 ppm, and $(CD_3)_2SO$ δ 2.50 ppm). Coupling constants (*J*) are quoted to the nearest 0.1 Hz. ^{13}C NMR spectra were recorded at 151 MHz on a Bruker 600 spectrometer with an AVANCE 3HD console, 126 MHz on Ascend 500 spectrometers with AVANCE 3 and AVANCE 3HD consoles, and at 101 MHz on a Bruker 400 spectrometer with AVANCE 3 console. Solvent peaks were used as an internal reference for ^{13}C NMR spectra ($CDCl_3$ δ 77.16 ppm, CD_3OD δ 49.00 ppm, CD_2Cl_2 δ 54.00 ppm, and $(CD_3)_2SO$ δ 39.52 ppm). Assignment of ^{1}H and ^{13}C NMR signals was assisted by ^{1}H-^{1}H COSY, ^{1}H-^{13}C HSQC, ^{1}H-^{13}C HMBC and ^{1}H-^{1}H NOESY experiments.

IR Spectroscopy: IR spectra were recorded as neat samples on a Shimadzu IR affinity-1 FTIR spectrometer fitted with an ATR attachment.

Mass Spectroscopy: EI mass spectra were recorded on a MAT 900 XP double focussing high resolution sector, run at 70 eV. ESI spectra were recorded on a Bruker microTOF, calibrated with sodium formate clusters, with data analysis using Data Analysis 4.1 (Bruker Daltonics).

Analytical TLC: TLC analyses were performed on Merck silica plates coated with silica gel 60 F254 (0.2mm) and were visualised with UV light and by staining with *p*-anisaldehyde or $KMnO_4$ standard TLC stain solutions, followed by heating.

Flash Chromatography: Flash chromatography was performed using Merck silica gel 60 (40–63 µm) for routine separations and Fluorochem silica gel 60 (20–45 µm) for more challenging separations. Neutralised silica refers to columns packed with solvent containing 1% NEt_3 and rinsed with between 1 and 2 column volumes of solvent (free of NEt_3) before use.

Optical Rotation: Optical rotations were recorded using a Bellingham Stanley ADP450 polarimeter with a Bellingham Stanley 0.5 mL cell (l = 0.25 dm). Concentrations (c) are reported in g/100 mL.

Chiral HPLC: Analytical chiral HPLC was conducted using a modular Shimadzu system using a LC-20AD pump, DGU-$20A_{5R}$ degassing unit, SIL-20A HT autosampler, CTO-20A column oven, SPD-20A UV/Vis detector and CDM-20A communications module.

Experimental Procedures, Reagents and Solvents: Unless stated otherwise, reactions were performed under a positive pressure of dry nitrogen using anhydrous solvents. Commercially available chemicals were used as received, unless specified otherwise. Solvents and reagents dried over 4 Å molecular sieves were dried for at least 24 h before use. Distillates were collected in flasks cooled with a dry ice/acetone bath to avoid loss of material. For reactions performed under anhydrous reaction conditions the reaction vessels were dried with a heat gun under vacuum prior to use. For anhydrous reactions, NEt_3 was dried over activated 4 Å molecular sieves for at least 24 h before use.

1.1 Experimental Procedure for Compound 16

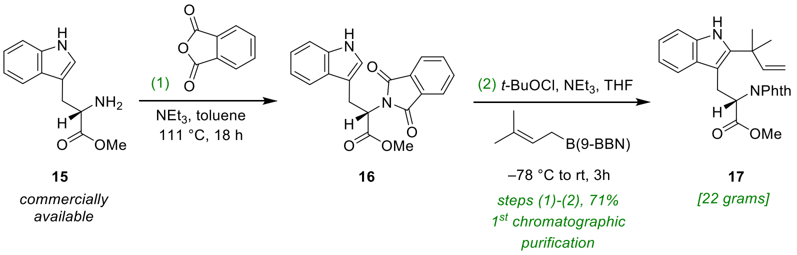

Step (1) – Based on conditions reported by Vederas and co-workers.[1] To a stirred suspension of L-tryptophan methyl ester hydrochloride (**15**) (25.0 g, 98.2 mmol) and phthalic anhydride (14.5 g, 98.2 mmol) in toluene (1000 mL) was added NEt_3 (27.4 mL, 196 mmol) and the mixture heated at reflux (111 °C) for 18 h. The reaction was allowed to cool to room temperature and a mixture of saturated aq. NH_4Cl (150 mL) and water (150 mL) was added. The organic phase was separated, washed with brine (300 mL), dried (Na_2SO_4) and concentrated under reduced pressure to give crude **16** (33.4 g, 98% crude mass recovery), which was used without further purification in the next step. A small sample was purified by flash chromatography (2:3 EtOAc/petroleum spirit) to give compound **16** as a pale-yellow foam suitable for characterisation. All data for compound **16** matched literature values.[1]

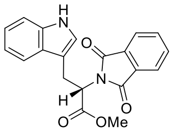

$\mathbf{R_f}$ 0.24 (2:3 EtOAc/petroleum spirit);

^{1}H NMR (500 MHz, $CDCl_3$) δ 7.97 (br. s, 1H), 7.74 (dd, J = 5.5, 3.1 Hz, 2H), 7.65 (dd, J = 5.5, 3.0 Hz, 2H), 7.60 (app. dt, J = 7.9, 0.9 Hz, 1H), 7.25 (app. dt, J = 7.3, 0.9, 0.9 Hz, 1H), 7.12 (ddd, J = 8.1, 7.0, 1.2 Hz, 1H), 7.05 (ddd, J = 8.0, 7.0, 1.1 Hz, 1H), 6.99 (d, J = 2.3 Hz, 1H), 5.28 (dd, J = 9.9, 6.1 Hz, 1H), 3.79 (s, 3H), 3.77 – 3.71 (m, 2H) ppm;

^{13}C NMR (126 MHz, $CDCl_3$) δ 169.8, 167.7, 136.2, 134.1, 131.8, 127.3, 123.5, 122.7, 122.2, 119.6, 118.6, 111.24, 111.21, 53.0, 52.7, 24.9 ppm;

IR (film, cm^{-1}) 3404, 3057, 3013, 2953, 2924, 2849, 1775, 1740, 1705, 1612, 1555;

HRMS (ESI$^+$) calc. for $C_{20}H_{16}N_2O_4$ ([M + H]$^+$): 349.1183; found: 349.1191), ([M + Na]$^+$): 371.1003; found: 371.1002;

$[\alpha]_D^{23.3}$ −201.1° (c 1.07, $CHCl_3$).

Reference:

1. Liu, H., Pattabiraman, V. R. & Vederas, J. C. Stereoselective Syntheses of 4-Oxa Diaminopimelic Acid and Its Protected Derivatives via Aziridine Ring Opening. *Org. Lett.* **9,** 4211–4214 (2007).

1.2 Experimental Procedure for Compound **17**

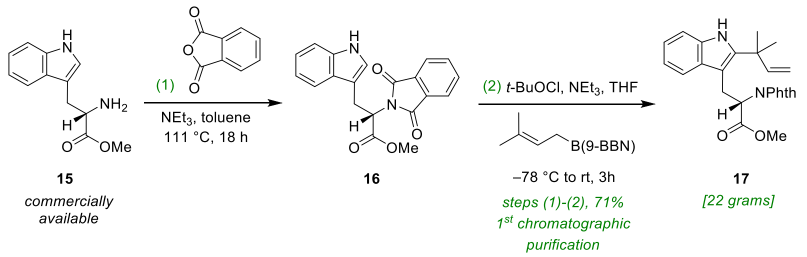

Step (2) – Based on a procedure reported by Danishefsky and co-workers.[1] Reaction performed under anhydrous reaction conditions. A solution of crude compound **16** (25.0 g) and dry NEt_3 (12.0 mL, 86.1 mmol) in THF (250 mL) was cooled to −78 °C in the absence of light. *t*-BuOCl (9.7 mL, 86.1 mmol, see page 25 for preparation) was added dropwise *via* a syringe pump over 30 minutes and the reaction stirred for 30 minutes at −78 °C. A solution of *B*-Prenyl-9-BBN (360 mL, 180 mmol, 0.5 M in THF, see pages 23-24 for preparation) was added dropwise at −78 °C, over 50 min *via* a cannula. After stirring for 1 h at −78 °C the cooling bath was removed and the reaction allowed to slowly warm to room temperature. Reaction progress was monitored by TLC analysis; starting material **16**, R_f 0.24 (1:1 EtOAc/petroleum spirit), intermediate, R_f 0.63 (1:1 EtOAc/petroleum spirit). After stirring for 2 h saturated aq. K_2CO_3 (75 mL) was added. The layers were separated and the aqueous layer extracted with EtOAc (3 × 125 mL). The combined organic layers were dried ($MgSO_4$) and concentrated under reduced pressure. The crude product was filtered through silica with CH_2Cl_2, concentrated and purified by flash chromatography (1:9 to 3:7 EtOAc/petroleum spirit) to give compound **17** (21.6 g, 51.9 mmol, 71% over 2 steps) as a pale-yellow foam. All spectroscopic data matched literature values.[1]

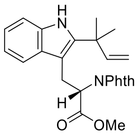

R_f 0.55 (1:1 EtOAc/petroleum spirit), 0.24 (1:4 EtOAc/petroleum spirit);

^{1}H NMR (600 MHz, $CDCl_3$) δ 7.86 (br. s, 1H), 7.69 (dd, J = 5.5, 3.0 Hz, 2H), 7.62 (dd, J = 5.5, 3.0 Hz, 2H), 7.28 (app. dt, J = 7.9, 0.9 Hz, 1H), 7.13 (app. dt, J = 8.0, 0.8 Hz, 1H), 6.90 (ddd, J = 8.1, 7.0, 1.1 Hz, 1H), 6.71 (ddd, J = 8.0, 7.0, 1.0 Hz, 1H), 6.19 (dd, J = 17.5, 10.5 Hz, 1H), 5.23 – 5.17 (m, 2H), 5.15 (dd, J = 10.5, 1.0 Hz, 1H), 3.86 (dd, J = 15.4, 3.9 Hz, 1H), 3.78 (s, 3H), 3.67 (dd, J = 15.4, 11.3 Hz, 1H), 1.58 (s, 3H), 1.57 (s, 3H) ppm;

^{13}C NMR (151 MHz, $CDCl_3$) δ 169.7, 167.8, 146.0, 140.3, 134.0, 134.0, 132.0, 129.9, 123.3, 121.3, 119.3, 117.9, 112.3, 110.3, 106.4, 53.6, 52.9, 39.3, 27.8, 27.7, 24.6 ppm;

$[\alpha]_D^{23.7}$ −221.6° (c 3.97, $CHCl_3$), literature: $[\alpha]_D^{25}$ −180.8° (c 3.9, $CHCl_3$).[1]

Reference:

1. Schkeryantz, J. M., Woo, J. C. G., Siliphaivanh, P., Depew, K. M. & Danishefsky, S. J. Total synthesis of gypsetin, deoxybrevianamide E, brevianamide E, and tryprostatin B: Novel constructions of 2,3-disubstituted indoles. *J. Am. Chem. Soc.* **121,** 11964–11975 (1999).

1.3 Experimental Procedure for Compound **18**

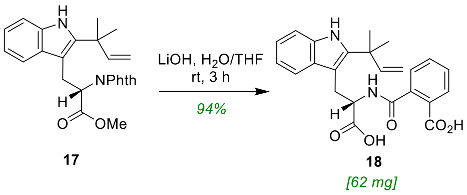

Compound **18** was prepared based on conditions reported by Ley and co-workers.[1] To a solution of compound **17** (67 mg, 0.16 mmol) in 1:1 THF/H2O (2 mL) was added solid LiOH (19 mg, 0.79 mmol). The solution was stirred at rt for 3 h before being neutralised with 1 M aq. HCl and the aqueous layer extracted with Et_2O (3 × 5 mL). The combined organics were washed with H_2O (2 × 2.5 mL), brine (2.5 mL), dried (Na_2SO_4) and concentrated under reduced pressure to give crude compound **18** (62 mg, 0.15 mmol, 94% crude mass recovery) as a yellow foam, which was sufficiently pure for characterisation purposes.

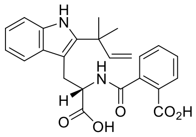

^{1}H NMR (500 MHz, CD_3OD) δ 7.91 (dd, *J* = 7.6, 1.6 Hz, 1H), 7.60 (d, *J* = 7.9 Hz, 1H), 7.46 (app. td, *J* = 7.6, 1.6 Hz, 1H), 7.42 (app. td, *J* = 7.5, 1.6 Hz, 1H), 7.31 (d, *J* = 8.0 Hz, 1H), 7.04 (ddd, *J* = 8.1, 6.9, 1.2 Hz, 1H), 6.98 – 6.93 (m, 1H), 6.89 (dd, *J* = 7.4, 1.5 Hz, 1H), 6.26 (dd, *J* = 17.5, 10.6 Hz, 1H), 5.17 (dd, *J* = 17.4, 1.3 Hz, 1H), 5.11 (dd, *J* = 10.5, 1.2 Hz, 1H), 4.98 (app. t, *J* = 7.8 Hz, 1H), 3.51 (dd, *J* = 14.5, 7.7 Hz, 1H), 3.27 (dd, *J* = 14.5, 7.9 Hz, 1H), 1.59 (s, 3H), 1.59 (s, 3H) ppm;

^{13}C NMR (126 MHz, CD_3OD) δ 175.3, 172.2, 169.2, 147.6, 142.1, 139.4, 136.4, 132.9, 131.2, 131.1, 130.5, 130.4, 129.0, 121.7, 119.6, 119.3, 112.1, 111.6, 106.6, 55.6, 40.4, 28.9, 28.5, 28.4 ppm;

IR (film, cm^{-1}) 3000, 1771, 1694, 1597, 1525, 1462, 1389;

HRMS (EI^+) calc. for $C_{24}H_{24}N_2O_5$ ($[M + H]^+$): 420.16797; found: 420.16655);

$[\boldsymbol{\alpha}]_{\boldsymbol{D}}^{\mathbf{23.7}}$ −28.3° (c 0.10, MeOH).

Reference:

1. Hewitt, P. R., Cleator, E. & Ley, S. V. A concise total synthesis of (+)-okaramine C. *Org. Biomol. Chem.* **2,** 2415–2417 (2004).

1.4 Experimental Procedure for Compound **19**

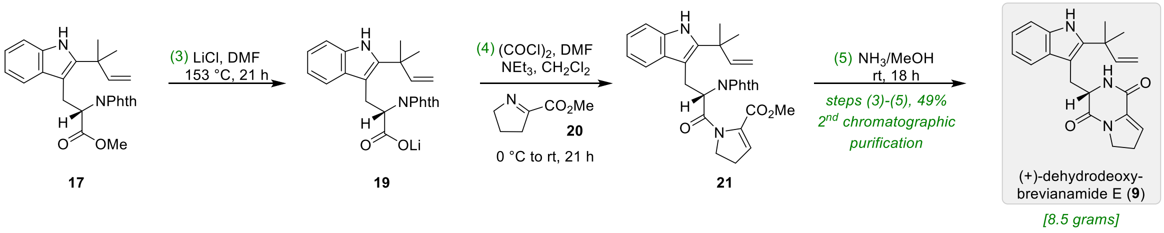

Step (3) – Adapted from the work of Hell,[1,2] and Fisher.[3] Compound **17** (24.6 g, 59.0 mmol) and LiCl (50.0 g, 1180 mmol) were suspended in DMF (125 mL) and the resulting mixture heated at 153 °C for 21 h under a flow of nitrogen. The thick brown suspension was diluted with CH_2Cl_2 (300 mL) and the solid was washed with CH_2Cl_2 (100 mL) and removed by filtration. The residual solid was broken up, suspended in CH_2Cl_2 (300 mL), stirred vigorously for 30 minutes. The solution was then filtered and the solid was again washed with CH_2Cl_2 (100 mL). The combined filtrates were concentrated under reduced pressure, then dried at 160 °C under reduced pressure. The crude product was re-dissolved in CH_2Cl_2 (200 mL), filtered and concentrated. This process was repeated to remove all residual LiCl, before being dried at 160 °C under reduced pressure to give crude lithium carboxylate **19** (23.6 g) as a brown foam, which was used without further purification in the next step.

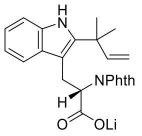

^{1}H NMR (500 MHz, CD_3OD) δ 7.62 (app. s, 4H), 7.21 (app. dt, *J* = 8.1, 0.9 Hz, 1H), 7.11 (app. t, *J* = 8.0, 0.9 Hz, 1H), 6.75 (ddd, *J* = 8.1, 7.0, 1.1 Hz, 1H), 6.53 (ddd, *J* = 8.0, 7.0, 1.0 Hz 1H), 6.26 (dd, *J* = 17.4, 10.6 Hz, 1H), 5.13 (dd, *J* = 17.4, 1.2 Hz, 1H), 5.04 (dd, *J* = 10.6, 1.2 Hz, 1H), 4.95 (dd, *J* = 12.0, 3.3 Hz, 1H), 3.84 (dd, *J* = 15.3, 3.3 Hz, 1H), 3.58 (dd, *J* = 15.3, 12.0 Hz, 1H), 1.59 (s, 3H), 1.54 (s, 3H) ppm;

^{13}C NMR (126 MHz, CD_3OD) δ 176.1, 170.1, 147.8, 141.3, 136.2, 134.6, 133.7, 131.1, 123.5, 121.2, 118.9, 118.5, 111.7, 111.3, 108.4, 57.8, 40.4, 28.6, 28.3, 26.0 ppm;

IR (film, cm^{-1}) 2924, 2365, 1773, 1701, 1611, 1466, 1396, 1350;

HRMS (ESI^-) calc. for $C_{24}H_{21}N_2O_4$ ($[M - Li]^-$): 401.1507; found: 401.1496);

$[\alpha]_D^{22.9}$ −226.0° (*c* 0.20, MeOH).

References:

1. Fölling, J., Belov, V., Kunetsky, R., Medda, R., Schönle, A., Egner, A., Eggeling, C., Bossi, M. & Hell, S. W. Photochromic rhodamines provide nanoscopy with optical sectioning. *Angew. Chem. Int. Ed.* **46,** 6266–6270 (2007).

2. Belov, V. N., Bossi, M. L., Fölling, J., Boyarskiy, V. P. & Hell, S. W. Rhodamine spiroamides for multicolor single-molecule switching fluorescent nanoscopy. *Chem. Eur. J.* **15,** 10762-10776 (2009).

3. Fisher, J. W. & Trinkle, K. L. Iodide dealkylation of benzyl, PMB, PNB, and t-Butyl N-acyl amino acid esters via lithium ion coordination. *Tetrahedron Lett.* **35,** 2505-2508 (1994).

1.5 Experimental Procedure for Compound **21**

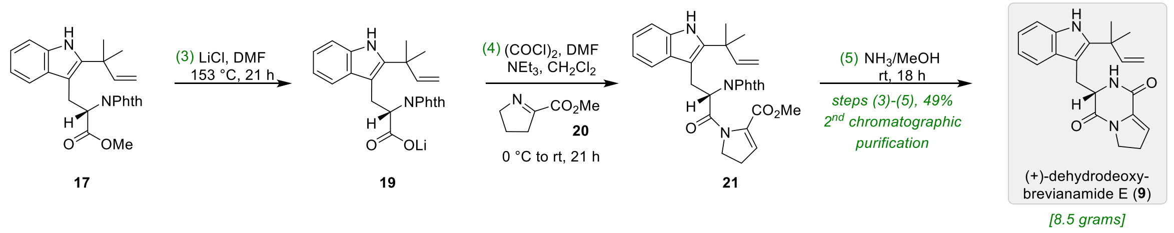

Step (4) – Adapted from the work of Schmalz,[1] and Soai.[2] Reaction performed under anhydrous reaction conditions. DMF (1.90 mL, 24.6 mmol) was added dropwise to a solution of oxalyl chloride (6.24 mL, 73.8 mmol) in CH_2Cl_2 (200 mL) at 0 °C. A suspension of crude compound **19** from **Step (3)** (20.1 g) in CH_2Cl_2 (250 mL) was then added *via* a cannula dropwise over 30 min at 0 °C and the resulting mixture was stirred at 0 °C for 30 min. A solution of compound **20** (5.84 mL, 49.2 mmol, see page 22 for preparation) and NEt_3 (10.3 mL, 73.8 mmol) in CH_2Cl_2 (200 mL) was added *via* a cannula over 20 min at 0 °C. The cooling bath was then removed and the reaction stirred for 21 h at rt. The reaction was quenched by the addition of 1 M aq. HCl (80 mL), the phases separated and the aqueous phase extracted with CH_2Cl_2 (3 × 50 mL). The combined organics were washed with saturated aq. $NaHCO_3$ (80 mL), and the aqueous layer then back extracted with CH_2Cl_2 (50 mL). The combined organics were washed with brine (120 mL), dried (Na_2SO_4) and concentrated under reduced pressure to give crude compound **21** (23.9 g) which was used without further purification in the next step. A small sample could be purified by flash chromatography (1:1 EtOAc/petroleum spirit) to give compound **21** as a lemon-yellow foam suitable for characterisation.

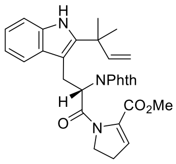

$\mathbf{R_f}$ 0.28 (1:1 EtOAc/petroleum spirit);

^{1}H NMR (500 MHz, CD_2Cl_2) δ 7.99 (s, 1H), 7.63 (app. s, 4H), 7.17 – 7.13 (m, 2H), 6.86 (dd, *J* = 8.2, 7.1 Hz, 1H), 6.61 (app. t, *J* = 7.4 Hz, 1H), 6.20 (dd, *J* = 17.4, 10.5 Hz, 1H), 5.87 (dd, *J* = 3.5, 2.5 Hz, 1H), 5.24 (dd, *J* = 10.4, 4.4 Hz, 1H), 5.20 (dd, *J* = 17.5, 1.1 Hz, 1H), 5.13 (dd, *J* = 10.6, 1.1 Hz, 1H), 3.94 (br. s, 1H), 3.83 (dd, *J* = 15.4, 4.4 Hz, 1H), 3.72 (s, 3H), 3.62 (app. q, *J* = 10.5 Hz, 1H), 3.46 (dd, *J* = 15.4, 10.4 Hz, 1H), 2.62 (app. dtd, *J* = 17.6, 10.0, 2.5 Hz, 1H), 2.39 (app. ddt, *J* = 17.5, 10.6, 3.4 Hz, 1H), 1.55 (s, 6H);

^{13}C NMR (151 MHz, CD_2Cl_2) δ 167.8, 167.7, 162.1, 146.6, 140.9, 137.0, 134.52, 134.48, 131.9, 130.3, 123.6, 123.5, 121.5, 119.4, 118.1, 112.3, 110.6, 106.6, 54.2 (beneath CD_2Cl_2 solvent peak), 52.6, 50.4 (br.), 39.7, 29.6, 27.91, 27.89, 25.4;

IR (film, cm^{-1}) 1775, 1713, 1655, 1639, 1611, 1437, 1381;

HRMS (ESI$^+$) calc. for $C_{30}H_{29}N_3O_5$ ($[M + H]^+$): 512.2180; found: 512.2202); ($[M + Na]^+$): 534.1999; found: 534.2016;

$[\boldsymbol{\alpha}]_{\boldsymbol{D}}^{\mathbf{23.0}}$ −130.1° (*c* 0.38, MeOH).

References:

1. Huy, P., Neudörfl, J.-M. & Schmalz, H.-G. A practical synthesis of trans-3-substituted proline derivatives through 1,4-addition. *Org. Lett.* **13,** 216-219 (2011).

2. Ookawa, A. & Soai, K. Asymmetric synthesis of optically active threo- and erythro-pyrrolidinylbenzyl alcohol by the highly stereospecific arylation of (*S*)-proline and the subsequent highly diastereoselective reduction of the α-amino ketone. *J. Chem. Soc., Perkin Trans. 1* 1465-1465 (1987).

1.6 Experimental Procedure for (+)-dehydrodeoxy-brevianamide E (**9**)

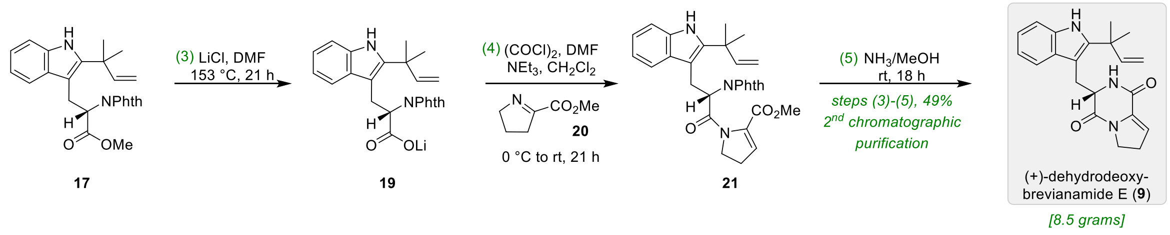

Step (5) – The crude product **21** (23.9 g) from **Step (4)** was dissolved in a solution of NH_3 in MeOH (7 M, 1200 mL) and stirred at room temperature for 18 h. The crude reaction mixture was concentrated under reduced pressure until approximately 25 mL of solvent was left. This was directly purified by flash chromatography (7:3 to 8:2 EtOAc/petroleum spirit) to give (+)-dehydrodeoxy-brevianamide E (**9**) (8.51 g, 24.4 mmol, 49% over 3 steps) as a pale-yellow foam. All spectroscopic data matched literature values.[1]

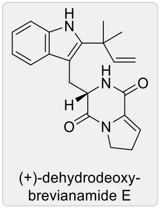

R_f 0.19 (8:2 EtOAc/petroleum spirit);

^{1}H NMR (500 MHz, $CDCl_3$) δ 8.07 (br. s, 1H), 7.53 (d, *J* = 7.9 Hz, 1H), 7.32 (app. dt, *J* = 8.1, 0.9 Hz, 1H), 7.17 (ddd, *J* = 8.1, 7.1, 1.2 Hz, 1H), 7.11 (ddd, *J* = 8.1, 7.1, 1.1 Hz, 1H), 6.14 (app. t, *J* = 3.1 Hz, 1H), 6.12 (dd, *J* = 17.2, 10.4 Hz, 1H), 5.66 (br. s, 1H), 5.20 – 5.15 (m, 2H), 4.52 (ddd, *J* = 11.3, 3.5, 1.8 Hz, 1H), 4.14 – 4.02 (m, 2H), 3.73 (dd, *J* = 14.7, 3.7 Hz, 1H), 3.23 (dd, *J* = 14.6, 11.3 Hz, 1H), 2.78 (app. td, *J* = 9.1, 3.1 Hz, 2H), 1.55 (s, 3H), 1.55 (s, 3H) ppm;

^{13}C NMR (126 MHz, $CDCl_3$) δ 162.7, 156.6, 145.8, 141.8, 134.4, 133.2, 128.9, 122.3, 120.2, 118.9, 118.3, 112.6, 110.9, 104.7, 57.6, 45.7, 39.2, 30.9, 28.1, 28.0, 27.9 ppm;

IR (film, cm^{-1}) 3331 (br.), 2967, 2926, 1672, 1639, 1435;

HRMS (ESI^+) calc. for $C_{21}H_{23}N_3O_2$ ($[M + H]^+$): 350.1863; found: 350.1875); ($[M + Na]^+$): 372.1682; found: 372.1689;

$[\boldsymbol{\alpha}]_{\boldsymbol{D}}^{\mathbf{23.1}}$ −33.2° (*c* 1.30, $CHCl_3$), literature: $[\boldsymbol{\alpha}]_{\boldsymbol{D}}^{\mathbf{22}}$ −38° (*c* 1.3, $CHCl_3$).[2]

References:

1. Greshock, T. J. & Williams, R. M. Improved biomimetic total synthesis of d,l-stephacidin A. *Org. Lett.* **9,** 4255–4258 (2007).

2. Steyn, P. S. The structures of five diketopiperazines from *Aspergillus ustus*. *Tetrahedron* **29,** 107–120 (1973).

1.7 Experimental Procedure for (+)-dehydro-brevianamide E (**10**) and Compound **22**

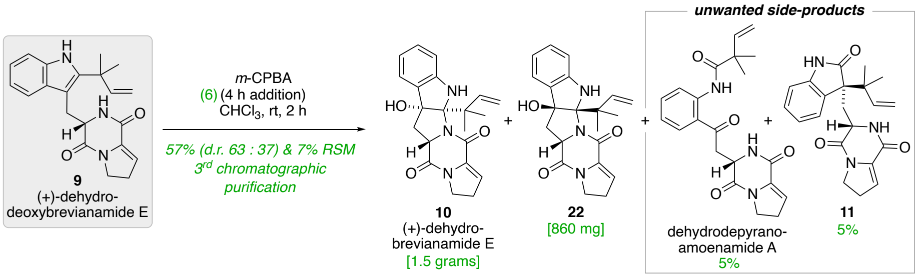

Step (6) – Adapted from the work of Kametani[1] and Wolff.[2] To a rapidly stirred solution of (+)-dehydrodeoxy-brevianamide E (**9**) (4.00 g, 11.5 mmol) in $CHCl_3$ (80 mL) at room temperature was added dropwise over 4 h a solution of *m*-CPBA in $CHCl_3$ (0.29 M, 40 mL, 11.7 mmol).* The reaction was stirred for 2 h at room temperature and quenched by the addition of saturated aq. $Na_2S_2O_3$ (20 mL) and saturated aq. $NaHCO_3$ (20 mL). The reaction mixture was diluted with EtOAc (200 mL) and $CHCl_3$ (150 mL) and the phases separated. The aqueous phase was back-extracted with $CHCl_3$ (50 mL) and the combined organics washed with saturated aq. $NaHCO_3$ (80 mL), brine (80 mL), dried (Na_2SO_4) and concentrated under reduced pressure. A diastereomeric ratio of 63:37 for **10**:**22** was determined by analysis of the ^{1}H NMR spectrum of this crude reaction product. Flash column chromatography (1.5:7:3 petroleum spirit/CH_2Cl_2/*i*-Pr_2O to 7:3 CH_2Cl_2/*i*-Pr_2O to EtOAc to *i*-PrOH) of the crude reaction product gave (+)-dehydro-brevianamide E (**10**) (1.43 g, 3.91 mmol, 34%) as a white foam, a fraction containing mixed (+)-dehydro-brevianamide E (**10**) and compound **22** (1.37 g, 18:82 **10/22**, 3.75 mmol, 33%) as a cream foam, recovered starting material (+)-dehydrodeoxy-brevianamide E (**9**) (0.270 g, 0.774 mmol, 7%) as a cream foam, and a mixed fraction containing dehydro-depyranoamoenamide A and oxindole **7** (0.414 g, 0.585 mmol/0.521 mmol, 5%/5%). A second round of chromatography on the mixed **10**/**22** fraction gave additional pure (+)-dehydro-brevianamide E (**10**) (0.108 g, 0.296 mmol, 3%) as a cream glass, pure minor diastereomer **22** (0.454 g, 1.24 mmol, 11%) as a white foam, and a fraction containing mixed dehydro-brevianamide E (**10**) and compound **22** (0.638 g, 9:91 **10/22**, 1.75 mmol, 15%). A third round of chromatography on this mixed **10/22** fraction gave additional pure minor diastereomer **22** (0.405 g, 1.11 mmol, 10%) as a white foam. Overall, purification by column chromatography gave (+)-dehydro-brevianamide E (**10**) (1.54 g, 4.21 mmol, 37%), compound **22** (0.859 g, 2.35 mmol, 20%), and recovered (+)-dehydrodeoxy-brevianamide E (**9**) (0.270 g, 0.774 mmol, 7%).

* *m*-CPBA (69% w/w by iodometric titration, 4.38 g, 17.5 mmol) was added to $CHCl_3$ (60 mL) and dried over Na_2SO_4 (27.5 g) with stirring for 40 mins.

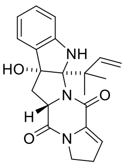

R_f 0.15 (7:3 CH_2Cl_2/*i*-Pr_2O);

^{1}H NMR (500 MHz, $CDCl_3$) δ 7.24 (dd, *J* = 7.5, 1.2 Hz, 1H), 7.18 (app. td, *J* = 7.7, 1.3 Hz, 1H), 6.81 (td, *J* = 7.4, 1.0 Hz, 1H), 6.74 (app. dt, *J* = 7.9, 0.8 Hz, 1H), 6.40 – 6.34 (m, 2H), 6.16 (app. t, *J* = 3.1 Hz, 1H), 5.15 (dd, *J* = 17.7, 1.4 Hz, 1H), 5.07 (dd, *J* = 10.9, 1.3 Hz, 1H),

4.06 (ddd, *J* = 12.0, 8.7, 7.3 Hz, 1H), 3.93 – 3.83 (m, 1H), 3.81 (dd, *J* = 11.5, 7.3 Hz, 1H), 2.79 – 2.69 (m, 4H), 2.43 (br. s, 1H), 1.34 (s, 3H), 1.27 (s, 3H) ppm;

^{13}C NMR (126 MHz, $CDCl_3$) δ 163.7, 162.1, 149.4, 144.7, 136.4, 131.0, 129.8, 123.8, 120.6, 120.2, 113.4, 111.2, 91.5, 89.3, 60.3, 45.8, 45.0, 36.7, 29.0, 27.6, 23.2 ppm;

IR (film, cm^{-1}) 3362, 2967, 2926, 1670, 1634, 1609, 1485, 1468, 1437, 1393, 1360;

HRMS (ESI^+) calc. for $C_{21}H_{23}N_3O_3$ ($[M + H]^+$): 366.1812; found: 366.1814); ($[M + Na]^+$): 388.1632; found: 388.1629;

$[\boldsymbol{\alpha}]_{\boldsymbol{D}}^{\mathbf{20.9}}$ −208° (*c* 0.23, EtOH).

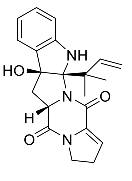

R$_f$ 0.09 (7:3 CH_2Cl_2/*i*-Pr_2O);

^{1}H NMR (500 MHz, $CDCl_3$) δ 7.22 (d, *J* = 7.5 Hz, 1H), 7.13 (app. td, *J* = 7.7, 1.3 Hz, 1H), 6.76 (app. td, *J* = 7.4, 0.9 Hz, 1H), 6.62 (app. dt, *J* = 7.9, 0.8 Hz, 1H), 6.47 (br. s, 1H), 6.34 (dd, *J* = 17.7, 10.8 Hz, 1H), 6.06 (app. t, *J* = 3.0 Hz, 1H), 5.22 (dd, *J* = 17.7, 1.3 Hz, 1H), 5.10 (dd, *J* = 10.9, 1.3 Hz, 1H), 4.73 (app. t, *J* = 8.8 Hz, 1H), 4.00 (ddd, *J* = 12.4, 11.1, 6.1 Hz 1H), 3.82 (dddd, *J* = 12.4, 11.4, 8.3, 0.7 Hz, 1H), 2.86 (ddd, *J* = 13.6, 8.6, 0.7 Hz, 1H), 2.80 – 2.62 (m, 3H), 2.57 (br. s 1H), 1.45 (s, 3H), 1.35 (s, 3H) ppm;

^{1}H NMR (500 MHz, $(CD_3)_2SO$) δ 7.16 (dd, *J* = 7.4, 1.4 Hz, 1H), 7.02 (td, *J* = 7.6, 1.3 Hz, 1H), 6.74 (s, 1H), 6.70 – 6.62 (m, 2H), 6.49 (dd, *J* = 17.6, 10.8 Hz, 1H), 5.90 (t, *J* = 3.0 Hz, 1H), 5.73 (s, 1H), 5.10 (dd, *J* = 17.6, 1.6 Hz, 1H), 5.00 (dd, *J* = 10.7, 1.6 Hz, 1H), 4.67 (dd, *J* = 10.2, 6.8 Hz, 1H), 3.86 – 3.76 (m, 1H), 3.63 (td, *J* = 11.6, 8.2 Hz, 1H), 2.74 (dd, *J* = 13.5, 6.8 Hz, 1H), 2.66 – 2.52 (m, 3H), 1.30 (d, *J* = 2.4 Hz, 6H) ppm;

^{13}C NMR (126 MHz, $CDCl_3$) δ 161.9, 156.2, 147.0, 144.8, 134.7, 131.5, 130.4, 123.8, 119.5, 119.3, 114.1, 109.9, 95.3, 89.6, 60.5, 46.6, 45.4, 43.4, 28.2, 27.2, 22.5 ppm;

^{13}C NMR (126 MHz, $(CD_3)_2SO$) δ 162.5, 155.5, 148.1, 145.5, 134.9, 133.0, 129.6, 124.1, 118.8, 118.7, 112.9, 110.1, 96.0, 87.4, 59.6, 45.5, 41.1, 31.2, 28.0, 25.3, 24.7 ppm;

IR (film, cm^{-1}) 3360, 2965, 2924, 2854, 1665, 1632, 1611, 1487, 1466, 1439, 1414, 1371;

HRMS (ESI^+) calc. for $C_{21}H_{23}N_3O_3$ ($[M + H]^+$): 366.1812; found: 366.1807;

$[\boldsymbol{\alpha}]_{\boldsymbol{D}}^{\mathbf{22.3}}$ +86.3° (*c* 0.19, EtOH).

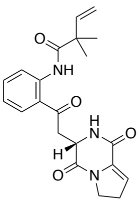

dehydrodepyrano-
amoenamide A

An analytically pure sample of dehydro-depyranoamoenamide A was purified by flash chromatography (1:9 MeOH/EtOAc).

R$_f$ 0.10 (EtOAc);

^{1}**H NMR** (601 MHz, $CDCl_3$) δ 11.49 (br. s, 1H), 8.81 (dd, *J* = 8.6, 1.1 Hz, 1H), 7.84 (dd, *J* = 8.1, 1.6 Hz, 1H), 7.58 (ddd, *J* = 8.7, 7.2, 1.6 Hz, 1H), 7.10 (ddd, *J* = 8.2, 7.3, 1.2 Hz, 1H), 6.49 (br. s, 1H), 6.24 (app. t, *J* = 3.0 Hz, 1H), 6.11 (dd, *J* = 17.4, 10.6 Hz, 1H), 5.36 (dd, *J* = 17.5, 0.8 Hz, 1H), 5.32 (dd, *J* = 10.6, 0.8 Hz, 1H), 4.68 (d, *J* = 10.2 Hz, 1H), 4.08 (app. t, *J* = 9.2 Hz, 2H), 4.00 (dd, *J* = 18.3, 2.2 Hz, 1H), 3.44 (dd, *J* = 18.3, 10.1 Hz, 1H), 2.86 – 2.80 (m, 2H), 1.43 (s, 3H), 1.42 (s, 3H) ppm;

^{13}**C NMR** (151 MHz, $CDCl_3$) δ 201.2, 176.1, 161.7, 156.5, 142.3, 141.6, 136.0, 132.7, 130.6, 122.4, 121.1, 120.8, 119.7, 115.0, 53.6, 46.9, 45.8, 44.9, 27.9, 24.7, 24.7 ppm;

IR (cm^{-1}) 3694, 3680, 3238, 2967, 2922, 2866, 2845, 1680, 1645;

HRMS (ESI^+) *m/z* 382.1750 (calculated $[M + H]^+$ 382.1761), 404.1564 (calculated $[M + Na]^+$ 404.1581).

References:

1. Kametani, T., Kanaya, N. & Ihara, M. Asymmetric total synthesis of brevianamide E. *J. Am. Chem. Soc.* **1980,** *102* (11), 3974-3975.

2. Adam, W., Bosio, S. G. & Wolff, B. T. Chiral-Auxiliary-Controlled Diastereoselectivity in the Epoxidation of Enecarbamates with DMD and *m*CPBA. *Org. Lett.* **2003,** *5* (6), 819-822.

1.8 Experimental Procedure for (+)-Brevianamide A (**1**) and (–)-Brevianamide B (**2**)

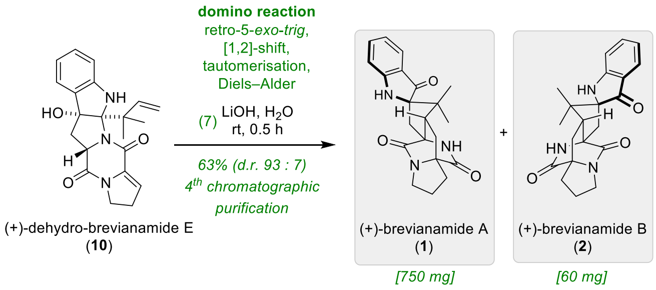

Step (7) – The synthesis of brevianamide A (**1**) was carried out under aerobic conditions, adapted from the work of Kishi[1] and Williams.[2] Effort was made to limit the exposure of the material to light once the reaction was complete, to prevent photolysis of brevianamide A (**1**) to brevianamide C, D and B (**2**).[3] To a solution of (+)-dehydro-brevianamide E (**10**) (1.30 g, 3.56 mmol) was added a 1 M solution of aq. LiOH (450 mL) and the reaction stirred rapidly, with vigorous manual shaking every 2–3 minutes to facilitate gradual dissolution of the starting material. After 30 minutes the reaction mixture was extracted with CH_2Cl_2 (2 × 450 mL, 2 × 200 mL), and the combined organics dried (Na_2SO_4). Flash column chromatography (2:8 to 3:7 to 1:1 THF/$CHCl_3$ to 25:25:1 THF/$CHCl_3$/*i*-PrOH) gave (+)-brevianamide A (**1**) (905 mg, 16.2% $CHCl_3$ w/w, 2.07 mmol, 58%)* as a yellow amorphous solid and (+)-brevianamide B (**2**) (59.8 mg, 0.164 mmol, 5%) as a yellow crystalline solid. A sample of (+)-brevianamide A (**1**) was crystallised from $CHCl_3$ by slow vapour diffusion with Et_2O.

*In Birch's original isolation paper brevianamide A '*crystallized from $CHCl_3$ in needles containing one molecule of solvent of crystallisation*'.[4] The reported crystal structure for 5-bromo-brevianamide A also showed co-crystallisation with one molecule of acetone.[5] Consistent with these observations, we found that a certain portion of solvent could not be removed from brevianamide A under high vacuum. Proteo-solvent was replaced with deutero-solvent to record NMR spectra for characterisation.

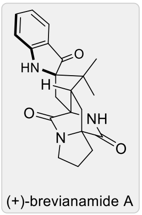

Spectroscopic data matched literature values reported by Zhu Weiming and co-workers.[6] Subtle differences in ^1H NMR data are attributed to concentration effects in $CDCl_3$ (see page 68).

R$_f$ 0.21 (3:7 THF/$CHCl_3$);

MP 173–179 °C, literature: 175–180 °C '*with loss of solvent*';[4]

^{1}H NMR (500 MHz, $CDCl_3$) δ 7.57 (dd, *J* = 7.7, 1.2 Hz, 1H), 7.44 (ddd, *J* = 8.3, 7.1, 1.4 Hz, 1H), 6.84 – 6.78 (m, 2H), 6.62 (br. s, 1H), 4.98 (br. s, 1H), 3.52 – 3.41 (m, 2H), 2.81 – 2.74 (m, 2H), 2.40 (ddd, *J* = 9.8, 7.3, 1.1 Hz, 1H), 2.35 (d, *J* = 15.6 Hz, 1H), 2.04 (app. pd, *J* = 6.8, 1.6 Hz, 2H), 1.96 – 1.81 (m, 3H), 1.12 (s, 3H), 0.93 (s, 3H) ppm;

^{13}C NMR (126 MHz, $CDCl_3$) δ 202.2, 172.4, 169.8, 160.2, 137.8, 124.8, 121.2, 119.3, 112.1, 78.9, 69.5, 67.8, 55.7, 48.4, 44.1, 37.4, 29.2, 29.0, 25.1, 24.1, 19.9 ppm;

^{1}H NMR (500 MHz, $(CD_3)_2SO$) δ 8.65 (br. s, 1H), 7.84 (br. s, 1H), 7.41 (ddd, *J* = 8.3, 7.0, 1.4 Hz, 1H), 7.37 (d, *J* = 7.7 Hz, 1H), 6.81 (dd, *J* = 8.2, 0.8 Hz, 1H), 6.64 (ddd, *J* = 7.8, 7.0, 0.8 Hz, 1H), 3.34 – 3.23 (m, 2H), 2.55 (ddd, *J* = 10.1, 7.5, 1.1 Hz, 1H), 2.49 – 2.44 (m, 2H), 2.39 (d, *J* = 15.1 Hz, 1H), 2.01 – 1.91 (m, 1H), 1.90 – 1.77 (m, 3H), 1.68 (dd, *J* = 13.1, 7.6 Hz, 1H), 0.98 (s, 3H), 0.68 (s, 3H) ppm;

^{13}C NMR (126 MHz, $(CD_3)_2SO$) δ 199.9, 172.1, 169.5, 160.5, 137.2, 123.6, 119.4, 116.7, 111.1, 79.0, 68.5, 66.4, 53.1, 48.3, 43.2, 39.5, 28.4, 27.2, 24.6, 21.2, 17.7 ppm;

IR (film, cm^{-1}) 3314, 3229, 2938, 2876, 1667, 1618, 1491, 1466, 1395;

HRMS (ESI^+) calc. for $C_{21}H_{23}N_3O_3$ ($[M + H]^+$): 366.1812; found: 366.1818;

$[\boldsymbol{\alpha}]_{\boldsymbol{D}}^{\mathbf{23.5}}$ +316° (*c* 0.12, EtOH), literature: $[\boldsymbol{\alpha}]_{\boldsymbol{D}}^{\mathbf{25}}$ +413° (EtOH);[4]

e.r. 93:7, after crystallisation 99:1 (Chiralpak IA, 1:1 *i*-PrOH/hexane, 1 mL min^{-1}, λ 254 nm) t_{Rminor} = 5.41 min, t_{Rmajor} = 6.72 min. See pages 46-47 for chiral HPLC traces.

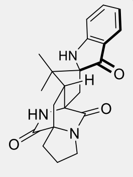

(+)-brevianamide B

Brevianamide B (**2**) was only sparingly soluble in $CDCl_3$, consistent with the observation in Birch's original isolation paper that brevianamide B '*was insoluble in most solvents except hot DMSO and CF_3CO_2H, the latter causing decomposition*'.[3] ^{1}H and ^{13}C NMR spectra could be recorded using a saturated sample in $CDCl_3$, to enable comparison to literature data,[7,8] however 2D NMR spectra were unobtainable. High quality 1D and 2D NMR spectra were instead recorded in $(CD_3)_2SO$.

R_f 0.08 (3:7 THF/$CHCl_3$);

MP 286–290 °C (decomposition), literature: 324–328 °C (decomposition);[3]

^{1}H NMR (500 MHz, $CDCl_3$) δ 7.56 (app. ddt, *J* = 7.6, 1.3, 0.7 Hz, 1H), 7.43 (ddd, *J* = 8.4, 7.1, 1.4 Hz, 1H), 6.84 – 6.78 (m, 2H), 5.78 (br. s, 1H), 4.73 (br. s, 1H), 3.50 – 3.44 (m, 2H), 3.31 (ddd, *J* = 10.3, 7.4, 1.3 Hz, 1H), 3.27 (d, *J* = 15.6 Hz, 1H), 2.74 (ddd, *J* = 13.1, 7.0, 6.1 Hz, 1H), 2.06 – 1.93 (m, 3H), 1.90 – 1.77 (m, 2H), 1.70 (d, *J* = 15.7, 1H), 1.14 (s, 3H), 0.83 (s, 3H) ppm;

^{13}C NMR (126 MHz, $CDCl_3$) δ 203.9, 173.4, 169.2, 160.3, 137.5, 125.2, 120.1, 119.2, 111.5, 77.7, 68.9, 66.5, 49.7, 46.6, 44.0, 36.8, 29.3, 28.6, 25.1, 22.6, 20.5 ppm;

^{1}H NMR (500 MHz, $(CD_3)_2SO$) δ 8.64 (br. s, 1H), 7.41 (ddd, *J* = 8.3, 7.1, 1.4 Hz, 1H), 7.33 (d, *J* = 7.7 Hz, 1H), 7.27 (br. s, 1H), 6.89 (app. dt, *J* = 8.3, 0.9 Hz, 1H), 6.64 (ddd, *J* = 7.8, 7.0, 0.9 Hz, 1H), 3.35 – 3.24 (m, 2H), 3.01 (ddd, *J* = 10.3, 7.5, 1.1 Hz, 1H), 2.67 (d, *J* = 15.1 Hz, 1H), 2.46 (dd, *J* = 11.9, 6.1 Hz, 1H), 2.02 – 1.90 (m, 3H), 1.85 – 1.74 (m, 2H), 1.63 (dd, *J* = 13.0, 7.5 Hz, 1H), 1.05 (s, 3H), 0.63 (s, 3H) ppm;

^{13}C NMR (126 MHz, $(CD_3)_2SO$) δ 204.7, 172.6, 169.2, 161.3, 137.2, 123.8, 118.1, 116.9, 111.4, 77.5, 68.1, 65.2, 48.8, 46.0, 43.3, 33.4, 28.4, 27.5, 24.4, 21.9, 19.7 ppm;

IR (film, cm^{-1}) 3348, 3231, 2949, 2878, 1705, 1664, 1653, 1616, 1587, 1495, 1468;

HRMS (ESI^+) calc. for $C_{21}H_{23}N_3O_3$ ($[M + H]^+$): 366.1812; found: 366.1796;

$[\boldsymbol{\alpha}]_{\boldsymbol{D}}^{\mathbf{24.7}}$ +136° (*c* 0.23, 2.5% HCO_2H in CH_2Cl_2), literature (enantiomer): $[\boldsymbol{\alpha}]_{\boldsymbol{D}}^{\mathbf{25}}$ −147° (*c* 0.23, 2.5% HCO_2H in CH_2Cl_2),[8] $[\boldsymbol{\alpha}]_{\boldsymbol{D}}^{\mathbf{25}}$ −124° (*c* 0.81, 2.5% HCO_2H in CH_2Cl_2);[7]

e.r. 93:7 (Chiralpak IA, 1:3 EtOH/hexane, 1 mL min^{-1}, λ 254 nm) t_{Rminor} = 13.99 min, t_{Rmajor} = 26.10 min. See pages 48-49 for chiral HPLC traces.

References:

1. Hutchison, A. J. & Kishi, Y. Stereospecific total synthesis of dl-austamide. *J. Am. Chem. Soc.* **101,** 6786–6788 (1979).

2. Greshock, T. J. & Williams, R. M. Improved biomimetic total synthesis of d,l- stephacidin A. *Org. Lett.* **9,** 4255–4258 (2007).

3. Birch, A. J. & Russell, R. A. Studies in relation to biosynthesis—XLIV: Structural elucidations of brevianamides-B, -C, -D and -F. *Tetrahedron* **28,** 2999–3008 (1972).

4. Birch, A. J. & Wright, J. J. Studies in relation to biosynthesis—XLII: The structural elucidation and some aspects of the biosynthesis of the brevianamides-A and -E. *Tetrahedron* **26,** 2329–2344 (1970).

5. Coetzer, J. The structure and absolute configuration of 5-bromobrevianamide A. *Acta Crystallographica Section B* **30,** 2254–2256 (1974).

6. Lingling, C., Tonghan, Z., Guoliang, Z., Yunlong, L., Cong, W., Pawinee, P., Arthit, C. & Weiming, Z. Bioactive Natural Products from the Marine-Derived *Penicillium brevicompactum* OUCMDZ-4920. *Chin. J. Org. Chem.* **37,** 2752–2762 (2017).

7. Williams, R. M., Glinka, T., Kwast, E., Coffman, H. & Stille, J. K. Asymmetric, stereocontrolled total synthesis of (-)-brevianamide B. *J. Am. Chem. Soc.* **112,** 808–821 (1990).

8. Frebault, F. C. & Simpkins, N. S. A cationic cyclisation route to prenylated indole alkaloids: synthesis of malbrancheamide B and brevianamide B, and progress towards stephacidin A. *Tetrahedron* **66,** 6585–6596 (2010).

1.9 Experimental Procedure for (–)-Brevianamide A (***ent*-1**) and (–)-Brevianamide B (***ent*-2**)

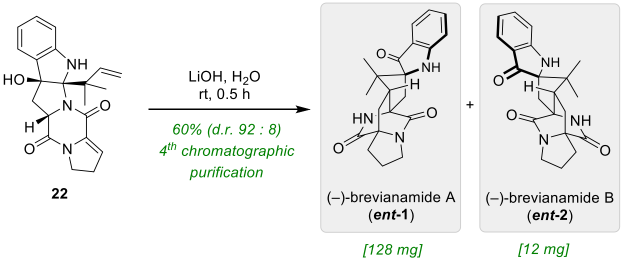

To a solution of compound **22** (234 mg, 0.640 mmol) was added a 1 M solution of aq. LiOH (80 mL) and the reaction stirred rapidly, with vigorous manual shaking every 2–3 minutes to facilitate gradual dissolution of the starting material. After 30 minutes the reaction mixture was extracted with CH_2Cl_2 (4 × 100 mL), and the combined organics dried (Na_2SO_4). Flash column chromatography (2:8 to 3:7 to 1:1 THF/$CHCl_3$ to 25:25:1 THF/$CHCl_3$/*i*-PrOH) gave (–)-brevianamide A (***ent*-1**) (155 mg, 17.5% $CHCl_3$ w/w, 0.350 mmol, 55%)* as a yellow amorphous solid and (–)-brevianamide B (***ent*-2**) (12 mg, 0.032 mmol, 5%) as a yellow crystalline solid.

*In Birch's original isolation paper brevianamide A '*crystallized from CHCl₃ in needles containing one molecule of solvent of crystallisation*'.[1] The reported crystal structure for 5-bromo-brevianamide A also showed co-crystallisation with one molecule of acetone.[2] Consistent with these observations, we found that a certain portion of solvent could not be removed from brevianamide A under high vacuum. Proteo-solvent was replaced with deutero-solvent to record NMR spectra for characterisation.

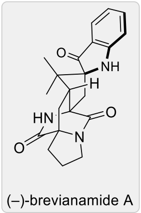

$[\alpha]_D^{24.7}$ −281° (*c* 0.12, EtOH), literature (enantiomer): $[\alpha]_D^{25}$ +413° (EtOH);[1]

e.r. 95:5 (Chiralpak IA, 1:1 *i*-PrOH/hexane, 1 mL min^{-1}, λ 254 nm) t_{Rmajor} = 5.38 min, t_{Rminor} = 6.74 min. See Pages 46-47 for chiral HPLC traces.

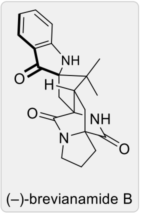

$[\alpha]_D^{25.1}$ −119° (*c* 0.23, 2.5% HCO_2H in CH_2Cl_2), literature: $[\alpha]_D^{25}$ −147° (*c* 0.23, 2.5% HCO_2H in CH_2Cl_2),[3] $[\alpha]_D^{25}$ −124° (*c* 0.81, 2.5% HCO_2H in CH_2Cl_2);[4]

e.r. 92:8 (Chiralpak IA, 1:3 EtOH/hexane, 1 mL min^{-1}, λ 254 nm) t_{Rmajor} = 13.86 min, t_{Rminor} = 26.14 min. See Pages 48-49 for chiral HPLC traces.

References:

1. Birch, A. J., & Wright, J. J. Studies in relation to biosynthesis—XLII: The structural elucidation and some aspects of the biosynthesis of the brevianamides-A and -E. *Tetrahedron* **1970,** *26* (10), 2329-2344.

2. Coetzer, J. The structure and absolute configuration of 5-bromobrevianamide A. *Acta Crystallographica Section B* **30,** 2254–2256 (1974).

3. Frebault, F. C. & Simpkins, N. S. A cationic cyclisation route to prenylated indole alkaloids: synthesis of malbrancheamide B and brevianamide B, and progress towards stephacidin A. *Tetrahedron* **66,** 6585–6596 (2010).

4. Williams, R. M., Glinka, T., Kwast, E., Coffman, H. & Stille, J. K. Asymmetric, stereocontrolled total synthesis of (-)-brevianamide B. *J. Am. Chem. Soc.* **112,** 808–821 (1990).

1.10 Experimental Procedure for Compound **20**

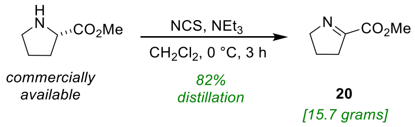

Compound **20** was prepared under anhydrous conditions, based on a procedure reported by Schmalz and co-workers.[1] To a rapidly stirred suspension of L-proline methyl ester hydrochloride (25.0 g, 151 mmol) and NEt_3 (48.3 mL, 347 mmol) in CH_2Cl_2 (450 mL) at 0 °C was added slowly, in 2.5 g portions, solid *N*-chlorosuccinimide (22.2 g, 166 mmol). The mixture was stirred at room temperature for 3 h and then diluted with CH_2Cl_2 (350 mL). A mixture of saturated aq. NH_4Cl (150 mL) and water (150 mL) was added, the organic phase was separated and the aqueous phase was extracted with CH_2Cl_2 (2 × 150 mL). The combined organics were washed with a mixture of saturated aq. NH_4Cl (100 mL) and water (100 mL), saturated aq. $NaHCO_3$ (150 mL), brine (150 mL), dried (Na_2SO_4) and concentrated under reduced pressure. Vacuum distillation with a 15 cm vigreux column under reduced pressure (5 mbar) with vigorous heating gave imine **20** (15.7 g, 123 mmol, 82%) as a pale-yellow liquid. All spectroscopic data matched literature values.[2]

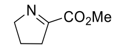

BP 65–69 °C (5 mbar);

^{1}H NMR (500 MHz, $CDCl_3$) δ 4.04 (app. tt, *J* = 7.6, 2.6 Hz, 2H), 3.80 (s, 3H), 2.76 (app. ddt, *J* = 8.5, 7.6, 2.6 Hz, 2H), 1.96 – 1.89 (m, 2H) ppm;

^{13}C NMR (126 MHz, $CDCl_3$) δ 168.2, 163.2, 62.5, 52.5, 35.3, 22.1 ppm.

References:

1. Huy, P., Neudörfl, J.-M. & Schmalz, H.-G. A practical synthesis of *trans*-3- substituted proline derivatives through 1,4-addition. *Org. Lett.* **13,** 216–219 (2011).

2. Sezen, B. & Sames, D. Oxidative C-Arylation of Free (NH)-Heterocycles via Direct (sp^3) C−H Bond Functionalization. *J. Am. Chem. Soc.* **126,** 13244–13246 (2004).

1.11 Experimental Procedure for *B*-Prenyl-9-BBN

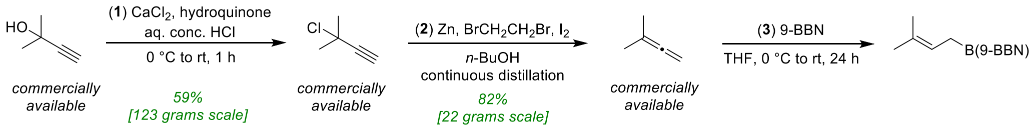

1,1-Dimethylallene is a commercially available reagent, but was prepared on large scale based on a procedure reported by Pfeffer and co-workers.[1]

Step (1) – Concentrated hydrochloric acid (800 mL) was cooled to 0 °C and stirred for 10 minute. To this solution was added 2-methyl-3-butyn-2-ol (200 mL, 2.06 mol) and hydroquinone (1.82 g, 16.5 mmol). Anhydrous $CaCl_2$ (229 g, 2.06 mol) was added in 20 g portions to the stirred mixture at 0 °C over 7 minutes and the reaction was stirred for 1 h at room temperature. The layers were seperated and solid K_2CO_3 added cautiously to the upper organic phase until effervescence ceased. The organic phase was then distilled under reduced pressure, collected up to 50 °C at 147 mbar. The crude distilate was redistilled under reduced pressure (147 mbar) to give 3-chloro-3-methyl-1-butyne (123 g, 1.00 mol, 59%) as a colourless oil. All spectroscopic data matched a comercially available sample.

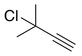

BP 32–36 °C (147 mbar);

^{1}H NMR (500 MHz, $CDCl_3$) δ 2.62 (s, 1H), 1.87 (s, 6H) ppm;

^{13}C NMR (101 MHz, $CDCl_3$) δ 86.7, 72.0, 57.1, 34.7 ppm.

Step (2) – Zinc powder (52.0 g, 760 mmol) was washed with 3% aq. HCl (4 × 40 mL), 2% aq. $CuSO_4$ (2 × 60 mL), ethanol (2 × 60 mL) and *n*-butanol (2 × 60 mL). The treated zinc was suspended in *n*-butanol (110 mL) in a 3 neck RBF with a dropping funnel, magnetic stirrer bar and a distillation head fitted with a 30 cm Vigreux column. 3-Chloro-3-methyl-1-butyne (44.4 mL, 390 mmol) was placed in the dropping funnel along with 1,2-dibromoethane (2.50 mL, 29.0 mmol). To the zinc butanol slurry was added a few crystals of iodine and 1,2-dibromoethane (2.50 mL, 29.0 mmol). Then a portion of the alkyne mixture (3 mL) was added dropwise to the slurry. The mixture was then heated cautiously (using a temperature controlled heat gun set slowly raised to 290 °C) with stirring until the reaction initiated (signalled by rapid expansion of the zinc slurry up the Vigreux column and then back into the reaction flask). The mixture was then kept under a controlled reflux from external heating with heat gun, while slow addition of the remaining alkyne mixture. The crude product was collected by distillation from the reaction mixture during the course of the reaction, and external heat applied to continue collection up to a temperature of 60 °C. The crude distillate was redistilled to give 1,1-dimethylallene (21.7 g, 319 mmol, 82%) as a colourless liquid. All spectroscopic data matched literature values.[1]

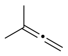

BP 40–42 °C;

^{1}H NMR (500 MHz, $CDCl_3$) δ 4.52 (hept, J = 3.1 Hz, 2H), 1.69 (t, J = 3.2 Hz, 6H) ppm;

^{13}C NMR (151 MHz, $CDCl_3$) δ 206.8, 94.2, 72.7, 20.3 ppm.

Step (3) – A solution of *B*-Prenyl-9-BBN was prepared under anhydrous conditions, based on a procedure reported by Trauner and co-workers.[66] A solution of 9-BBN in THF (360 mL, 179 mmol, 0.5 M in THF) was cooled to 0 °C and stirred for 20 minutes. Once the solution was observed to go cloudy, 1,1-dimethylallene (20.5 mL, 208 mmol) was added dropwise *via* a syringe pump over 30 minutes with stirring. The reaction was warmed to room temperature and stirred for 18 h before being used directly in the reverse prenylation procedure described on page 8.

References:

1. Chengebroyen, J., Linke, M., Robitzer, M., Sirlin, C. & Pfeffer, M. Palladium-mediated intramolecular C–N bond formation involving allyl substituted pyridines. Application to a novel strategy for the synthesis of the skeleton of berberinium derivatives. *J. Organomet. Chem.* **687,** 313–321 (2003).

2. Kuttruff, C. A., Zipse, H. & Trauner, D. Concise Total Syntheses of Variecolortides A and B through an Unusual Hetero-Diels–Alder Reaction. *Angew. Chem. Int. Ed.* **50,** 1402–1405 (2011).

1.12 Experimental Procedure for *t*-BuOCl

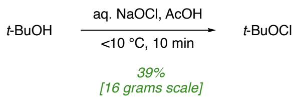

t-BuOCl was prepared based on a procedure reported by Mintz and co-workers.[1] For safety reasons exposure of the reaction and product to light was minimised.

An aqueous solution of bleach (500 mL, 0.767 M, 384 mmol)* was cooled below 10 °C with stirring. A pre mixed solution of *t*-BuOH (43.3 mL, 383 mmol) and glacial acetic acid (24.1 mL, 421 mmol) was added in a single portion to the rapidly stirred bleach and the stirring continued for 10 min in the absence of light. The entire reaction mixture was transferred to a separating funnel, the aqueous layer discarded, the yellow organic layer washed with and water (50 mL). The product was dried ($CaCl_2$ approximately 3 g) and decanted into its final container via pipette. The *t*-BuOCl (16.4 g, 151 mmol, 39%) was stored in a fridge over $CaCl_2$ in an amber glass bottle as a yellow liquid.

*Bleach solution was titrated to a concentration of 0.767 M by iodometric titration using sodium thiosulfate prior to use.

^{1}H NMR (500 MHz, $CDCl_3$) δ 1.33 (s, 9H) ppm;

^{13}C NMR (126 MHz, $CDCl_3$) δ 84.1, 27.0 ppm.

References:

1. Mintz, M. J. & Walling, C. *t*-BUTYL HYPOCHLORITE. *Org. Synth.* **49**, 9 (1969).

1.13 Experimental Procedure for Compounds 23 and 24

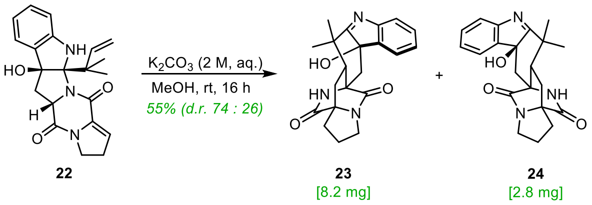

To a solution of compound **22** (20 mg, 54.7 µmol) in MeOH (1 mL) was added a solution of 2 M aq. K_2CO_3 (1 mL) and the reaction stirred at room temperature for 16 h. The reaction was acidified with 1 M aq. HCl (3 mL), extracted with CH_2Cl_2 (3 × 5 mL), dried (Na_2SO_4) and concentrated under reduced pressure. Preparative TLC (1:9 *i*-PrOH/$CHCl_3$) gave **23** (8.2 mg, 22.0 µmol, 41%) as a white glass and **24** (2.8 mg, 7.7 µmol, 14%) as a white glass.

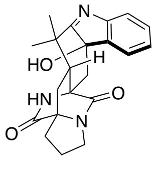

23

R_f 0.33 (1:9 *i*-PrOH/$CHCl_3$), 0.32 (3:7 THF/$CHCl_3$);

^{1}H NMR (500 MHz, $CDCl_3$) δ 7.51 – 7.46 (m, 1H), 7.38 (ddd, J = 7.3, 2.4, 1.4 Hz, 1H), 7.32 (app. td, J = 7.6, 1.2 Hz, 1H), 7.21 – 7.13 (m, 2H), 3.95 (app. hept, J = 6.1 Hz, 1H), 3.36 (tt, J = 6.5, 1.8 Hz, 2H), 2.75 (d, J = 15.8 Hz, 1H), 2.71 – 2.64 (m, 1H), 2.06 – 1.86 (m, 4H), 1.78 (dt, J = 13.0, 7.4 Hz, 1H), 1.39 (s, 3H), 1.29 (s, 3H) ppm;

^{1}H NMR (500 MHz, $(CD_3)_2SO$) δ 7.58 (s, 1H), 7.53 – 7.43 (m, 2H), 7.37 (td, J = 7.6, 1.3 Hz, 1H), 7.25 (td, J = 7.4, 1.0 Hz, 1H), 6.36 (d, J = 2.1 Hz, 1H), 3.34 (dt, J = 8.0, 5.5 Hz, 1H), 3.28 (ddd, J = 11.1, 5.6, 2.2 Hz, 1H), 2.69 (d, J = 15.4 Hz, 1H), 2.55 – 2.51 (m, 1H), 2.17 – 2.11 (m, 1H), 2.02 – 1.89 (m, 3H), 1.87 – 1.78 (m, 2H), 1.71 (dd, J = 15.4, 2.2 Hz, 1H), 1.38 (s, 3H), 1.22 (s, 3H) ppm;

^{13}C NMR (126 MHz, $CDCl_3$) δ 188.3, 172.4, 168.3, 151.9, 140.7, 129.9, 126.4, 122.3, 121.0, 82.2, 67.1, 62.0, 50.3, 44.0, 40.4, 37.9, 32.5, 29.0, 27.3, 24.3, 20.0 ppm;

IR (film, cm^{-1}) 3362, 2968, 2882, 1674, 1580, 1458, 1390;

HRMS (ESI$^+$) calc. for $C_{21}H_{23}N_3O_3$ ([M + H]$^+$): 366.1812; found: 366.1809;

$[\boldsymbol{\alpha}]_{\boldsymbol{D}}^{\mathbf{21.9}}$ +89.0 (c 0.53, MeOH).

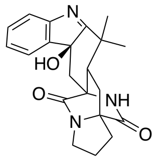

24

R_f 0.29 (1:9 *i*-PrOH/$CHCl_3$);

^{1}H NMR (500 MHz, $CDCl_3$) δ 7.50 (dddd, J = 6.4, 4.9, 1.5, 0.7 Hz, 2H), 7.37 (td app, J = 7.7, 1.3 Hz, 1H), 7.24 (dd, J = 7.5, 0.9 Hz, 1H), 6.28 (s, 1H), 3.80 – 3.70 (m, 1H), 3.56 – 3.33 (m, 4H), 2.80 – 2.68 (m, 1H), 2.14 (dd, J = 13.1, 10.2 Hz, 1H), 2.06 – 1.98 (m, 2H), 1.95 (dd, J = 13.2, 7.0 Hz, 1H), 1.87 (dt, J = 13.1, 7.1 Hz, 1H), 1.39 (s, 3H), 1.35 (app. s, 4H) ppm;

^{1}H NMR (500 MHz, $(CD_3)_2SO$) δ 8.24 (s, 1H), 7.54 – 7.47 (m, 1H), 7.43 (dd, *J* = 7.6, 0.9 Hz, 1H), 7.33 (app. td, *J* = 7.6, 1.3 Hz, 1H), 7.21 (app. td, *J* = 7.4, 1.0 Hz, 1H), 5.88 (d, *J* = 1.6 Hz, 1H), 3.55 (d, *J* = 15.0 Hz, 1H), 3.42 – 3.32 (m, 2H), 3.21 (dd, *J* = 10.5, 5.6 Hz, 1H), 2.17 (dd, *J* = 13.3, 10.5 Hz, 1H), 2.02 – 1.93 (m, 1H), 1.88 – 1.73 (m, 3H), 1.27 (s, 3H), 1.25 (s, 3H) ppm;

^{13}C NMR (126 MHz, $CDCl_3$) δ 189.3, 173.0, 170.5, 153.4, 139.4, 130.0, 126.5, 122.4, 120.8, 81.9, 67.1, 60.1, 44.4, 43.3, 39.0, 34.8, 30.5, 29.0, 28.1, 24.6, 23.4 ppm;

^{13}C NMR (126 MHz, $(CD_3)_2SO$) δ 192.1, 173.0, 169.2, 153.8, 142.0, 129.4, 126.1, 123.0, 120.4, 81.7, 66.8, 60.0, 44.3, 42.8, 38.9, 33.6, 30.7, 29.3, 29.1, 24.5, 23.0 ppm;

IR (film, cm^{-1}) 3269, 2970, 2879, 1694, 1674, 1578, 1456, 1404;

HRMS (ESI^+) calc. for $C_{21}H_{23}N_3O_3$ ($[M + H]^+$): 366.1812; found: 366.1821; ($[M + Na]^+$): 388.1632; found: 388.1635;

$[\alpha]_D^{23.3}$ +80.8 (*c* 0.24, MeOH).

References:

1. Williams, R. M., Sherman, D. H. & Shengying, L. Fungal-derived brevianamide assembly by a Stereoselective Semipinacolase. *Nature Cat.* **3**, 497-506 (2020).

1.14 Solvent Screen with LiOH and Compound 22

The following reactions show qualitatively that treating compound **22** with LiOH in different solvent systems favours the formation of (–)-brevianamide A (***ent*-1**).

To compound **22** (5.0 mg, 1.0 equiv., 14 µmol) was added LiOH (2 mL, 1 M, solution see table for solvent) at rt. The reaction mixture was left to stir for 24 h. The reaction was then acidified with 1 M aq. HCl (2 mL), extracted with $CHCl_3$ (3 × 5 mL), dried (Na_2SO_4) and concentrated under reduced pressure. The residue was transferred to an NMR tube with $CHCl_3$ (1.5 mL) and concentrated under reduced pressure. To the NMR tube was then added $(CD_3)_2SO$ containing ethylene carbonate internal standard (400 µL) and a ^{1}H NMR Spectrum (500 MHz, in $(CD_3)_2SO$) was recorded.

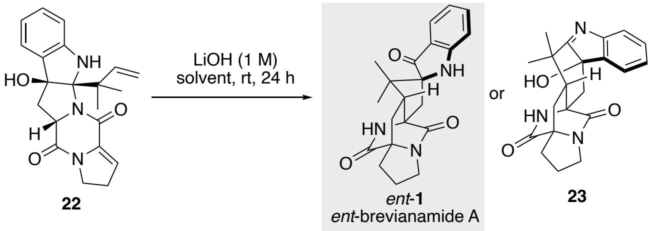

solvent		
experiment 1	experiment 2	experiment 3
H_2O	H_2O/MeOH (1:1)	MeOH
brevianamide A (*ent*-**1**) formed	brevianamide A (*ent*- **1**) formed	brevianamide A (*ent*-**1**) formed

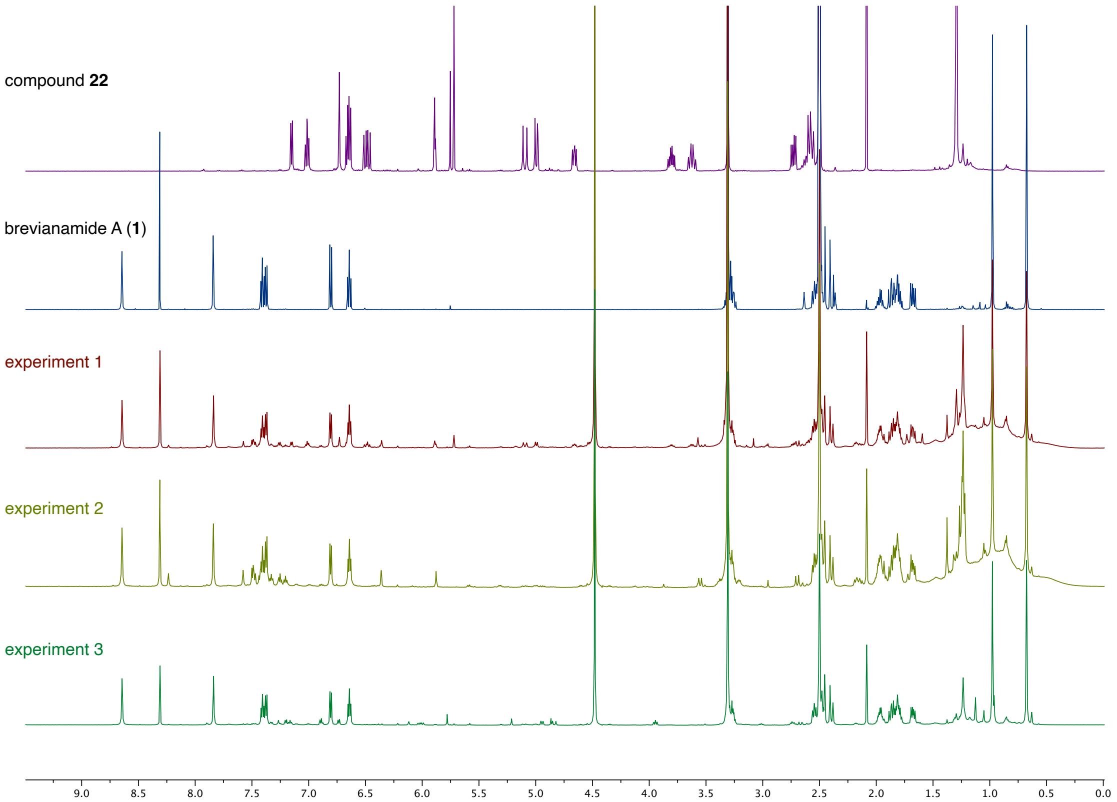

^{1}H NMR Spectrum (500 MHz, in $(CD_3)_2SO$)
peaks: 4.50 ppm is ethylene carbonate (internal standard), 8.31 ppm is $CHCl_3$ and 3.30 ppm is water.

1.15 Solvent Screen with K_2CO_3 and Compound 22

The following reactions show qualitatively that compound **23** is formed when compound **22** is treated with K_2CO_3 in a solvent system containing methanol.

To compound **22** (5.0 mg, 1.0 equiv., 14 µmol) was added K_2CO_3 (2 mL, 1 M, solution see table for solvent) at rt. The reaction mixture was left to stir for 24 h. The reaction was then acidified with 1 M aq. HCl (2 mL), extracted with $CHCl_3$ (3 × 5 mL), dried (Na_2SO_4) and concentrated under reduced pressure. The residue was transferred to an NMR tube with $CHCl_3$ (1.5 mL) and concentrated under reduced pressure. To the NMR tube was then added $(CD_3)_2SO$ containing ethylene carbonate internal standard (400 µL) and a ^{1}H NMR Spectrum (500 MHz, in $(CD_3)_2SO$) was recorded.

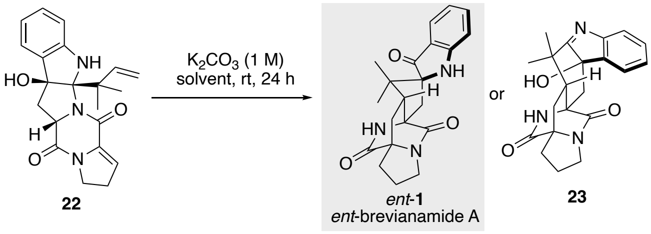

solvent		
experiment 1	experiment 2	experiment 3
H_2O	H_2O/MeOH (1:1)	MeOH
no reaction	compound **23** formed	compound **23** formed

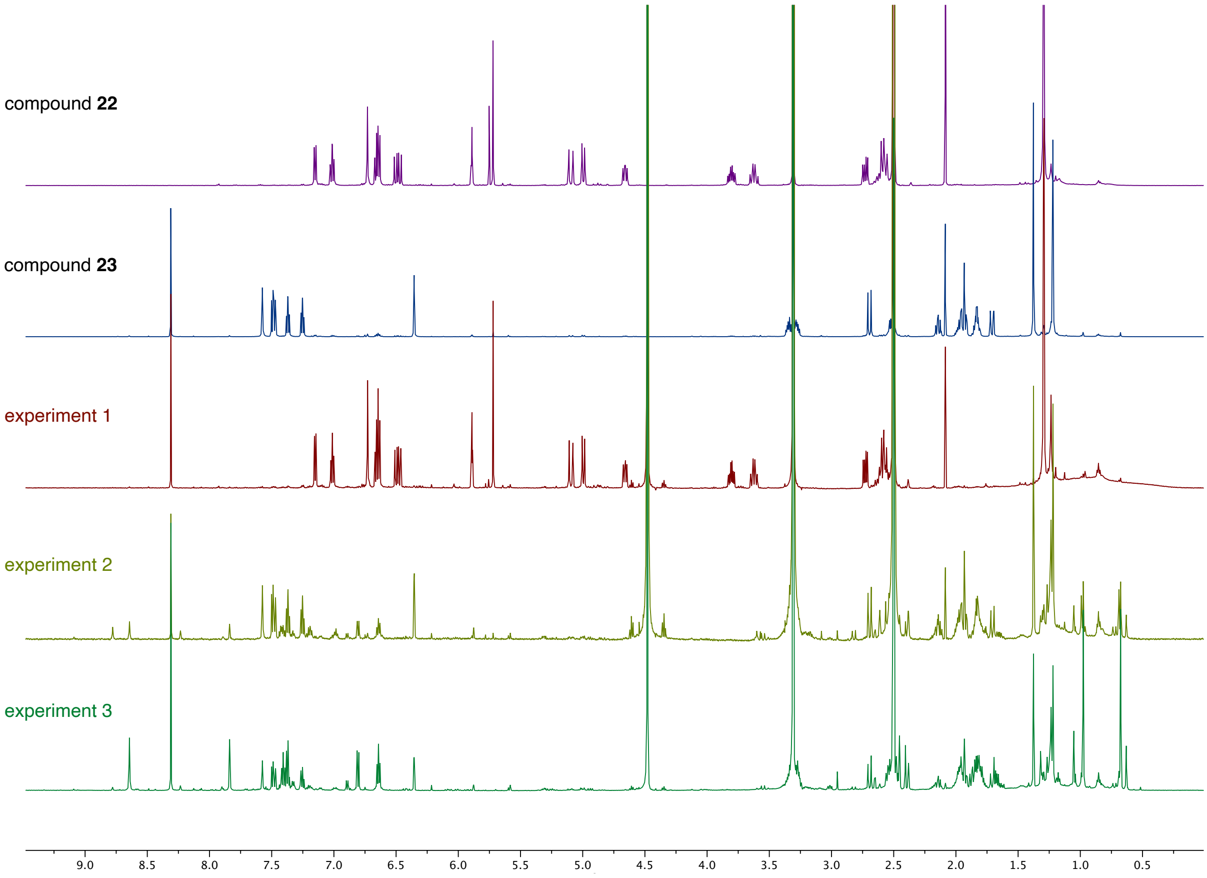

^{1}H NMR Spectrum (500 MHz, in $(CD_3)_2SO$)
peaks: 4.50 ppm is ethylene carbonate (internal standard), 8.31 ppm is $CHCl_3$ and 3.30 ppm is water.

1.16 Solvent Screen with LiOH and Compound 23

The following reactions show qualitatively that treating compound **23** to LiOH, in different solvent systems, does not promote the semi-pinacol rearrangement to give (–)-brevianamide A (***ent*-1**).

To compound **23** (5.0 mg, 1.0 equiv., 14 µmol) was added LiOH (2 mL, 1 M, solution see table for solvent) at rt. The reaction mixture was left to stir for 24 h. The reaction was then acidified with 1 M aq. HCl (2 mL), extracted with $CHCl_3$ (3 × 5 mL), dried (Na_2SO_4) and concentrated under reduced pressure. The residue was transferred to an NMR tube with $CHCl_3$ (1.5 mL) and concentrated under reduced pressure. To the NMR tube was then added $(CD_3)_2SO$ containing ethylene carbonate internal standard (400 µL) and a ^1H NMR Spectrum (500 MHz, in $(CD_3)_2SO$) was recorded.

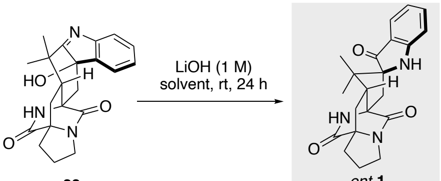

23 *ent*-**1** *ent*-brevianamide A

solvent		
experiment 1	experiment 2	experiment 3
H_2O	H_2O/MeOH (1:1)	MeOH
no reaction	no reaction	no reaction

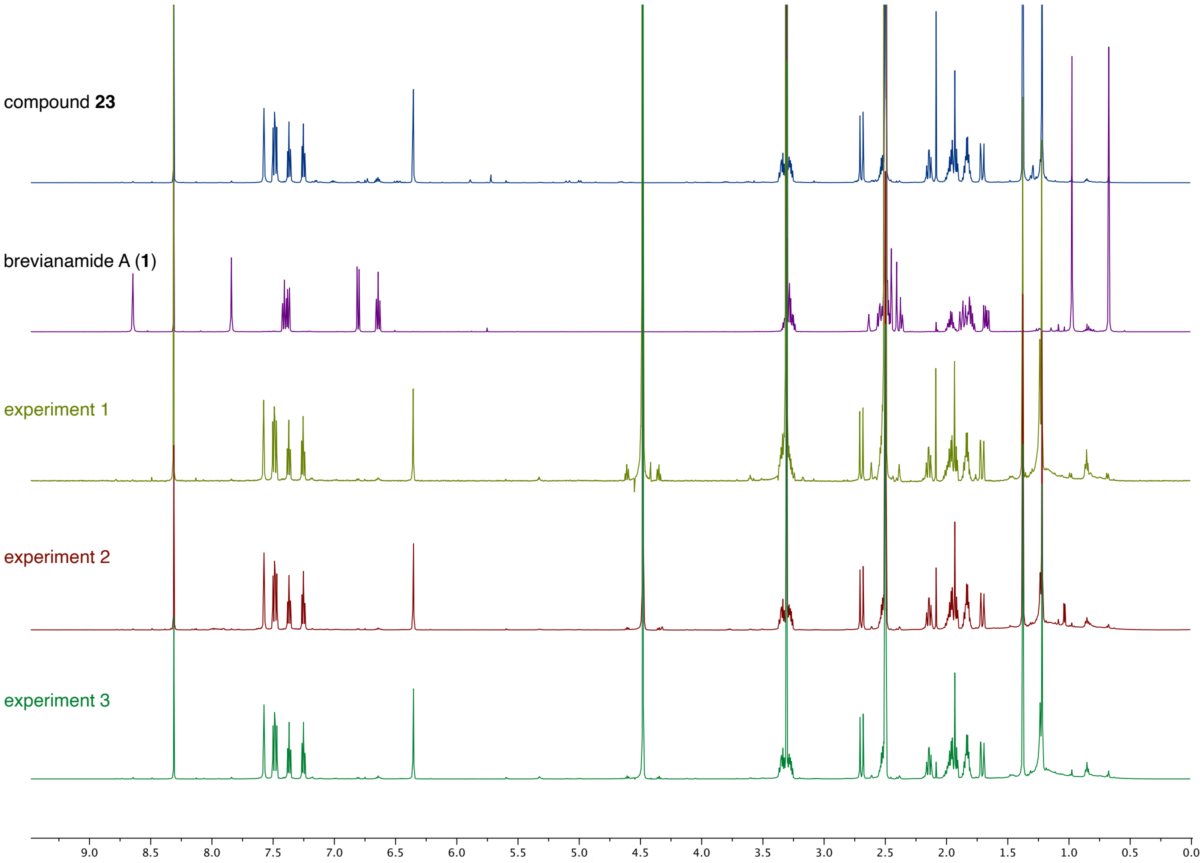

^1H NMR Spectrum (500 MHz, in $(CD_3)_2SO$)
peaks: 4.50 ppm is ethylene carbonate (internal standard), 8.31 ppm is $CHCl_3$ and 3.30 ppm is water.

1.17 Solvent Screen with LiOH and Compound 24

The following reactions show qualitatively that treating compound **24** to LiOH, in different solvent systems, results in the formation of (–)-brevianamide B (***ent*-2**) through a base induced semi-pinacol rearrangement.

To compound **24** (5.0 mg, 1.0 equiv., 14 µmol) was added LiOH (2 mL, 1 M, solution see table for solvent) at rt. The reaction mixture was left to stir for 24 h. The reaction was then acidified with 1 M aq. HCl (2 mL), extracted with $CHCl_3$ (3 × 5 mL), dried (Na_2SO_4) and concentrated under reduced pressure. The residue was transferred to an NMR tube with $CHCl_3$ (1.5 mL) and concentrated under reduced pressure. To the NMR tube was then added $(CD_3)_2SO$ containing ethylene carbonate internal standard (400 µL) and a ^{1}H NMR Spectrum (500 MHz, in $(CD_3)_2SO$) was recorded.

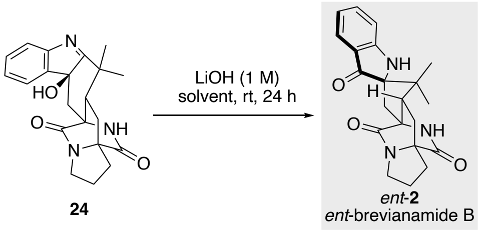

solvent		
experiment 1	experiment 2	experiment 3
H_2O	H_2O/MeOH (1:1)	MeOH
(–)-brevianamide B (***ent*-2**) observed	(–)-brevianamide B (***ent*-2**) observed	(–)-brevianamide B (***ent*-2**) observed

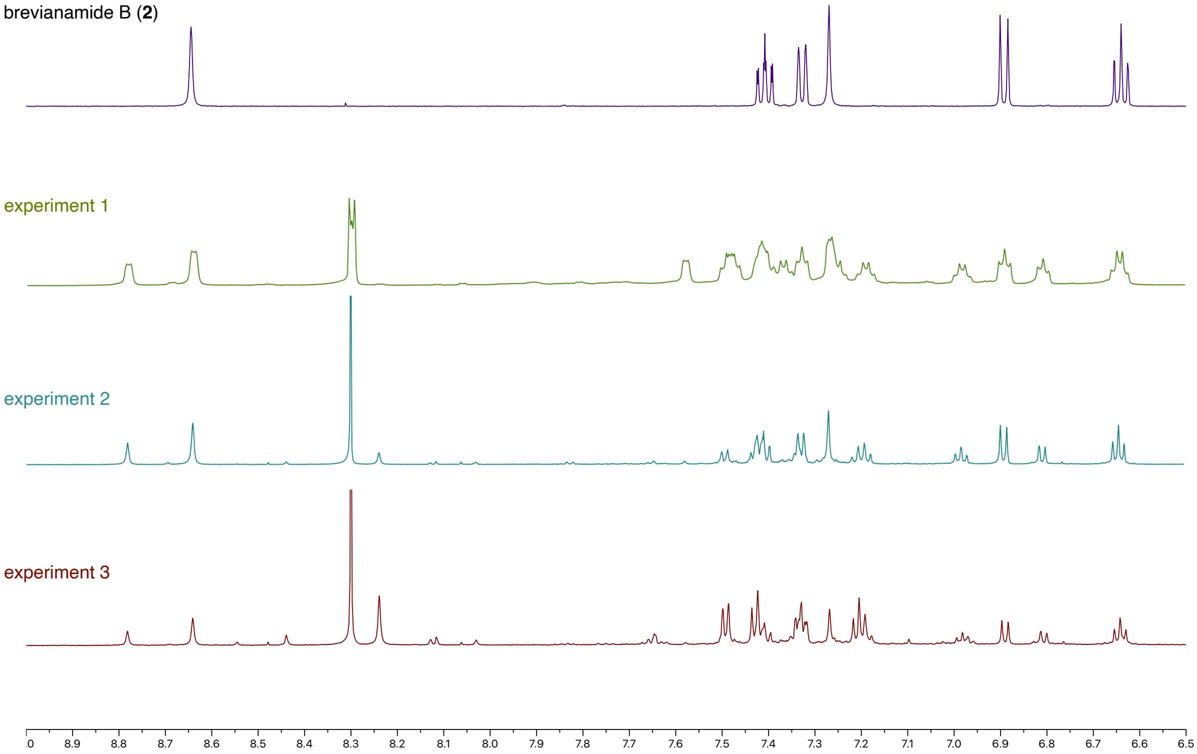

^{1}H NMR Spectrum (500 MHz, in $(CD_3)_2SO$)
peaks: 8.31 ppm is $CHCl_3$.

1.18 Solvent Screen with LiOH and (–)-brevianamide A (*ent*-1)

The following reactions show qualitatively that (–)-brevianamide A (***ent*-1**) is relatively stable to LiOH in different solvent systems and does not, for example, result in the formation of compound **23**, through a base induced retro-semi-pinacol rearrangement.

To (–)-brevianamide A (***ent*-1**) (5.0 mg, 1.0 equiv., 14 µmol) was added LiOH (2 mL, 1 M, solution see table for solvent) at rt. The reaction mixture was left to stir for 24 h. The reaction was then acidified with 1 M aq. HCl (2 mL), extracted with $CHCl_3$ (3 × 5 mL), dried (Na_2SO_4) and concentrated under reduced pressure. The residue was transferred to an NMR tube with $CHCl_3$ (1.5 mL) and concentrated under reduced pressure. To the NMR tube was then added $(CD_3)_2SO$ containing ethylene carbonate internal standard (400 µL) and a ^{1}H NMR Spectrum (500 MHz, in $(CD_3)_2SO$) was recorded.

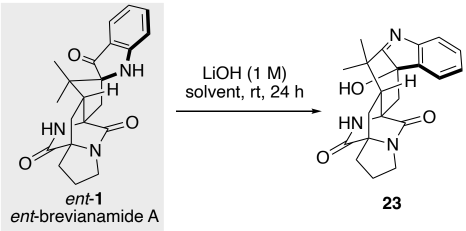

solvent		
experiment 1	experiment 2	experiment 3
H_2O	H_2O/MeOH (1:1)	MeOH
no reaction	no reaction	no reaction

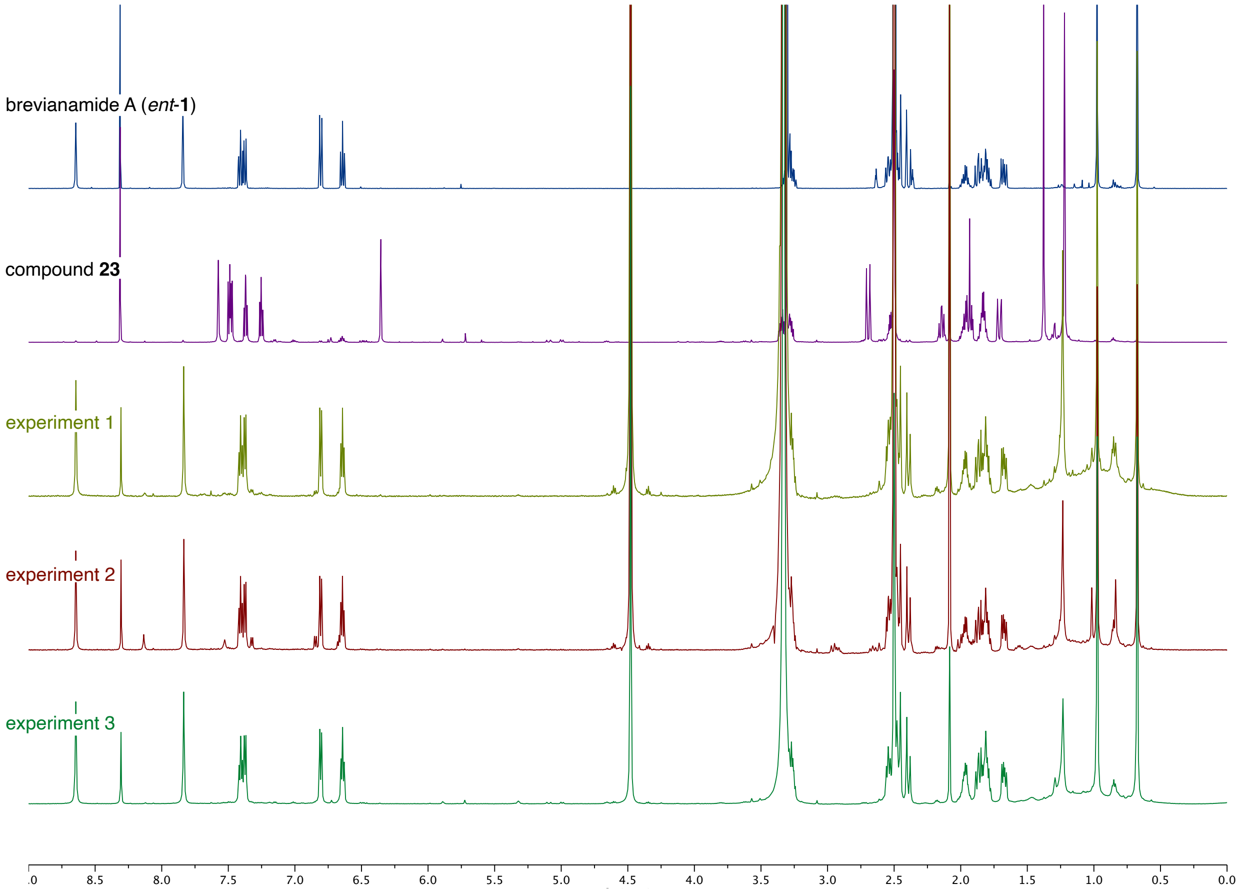

^{1}H NMR Spectrum (500 MHz, in $(CD_3)_2SO$)
peaks: 4.50 ppm is ethylene carbonate (internal standard), 8.31 ppm is $CHCl_3$ and 3.30 ppm is water.

1.19 Experimental Procedure for Compounds 11 and 27

Experimental procedure A

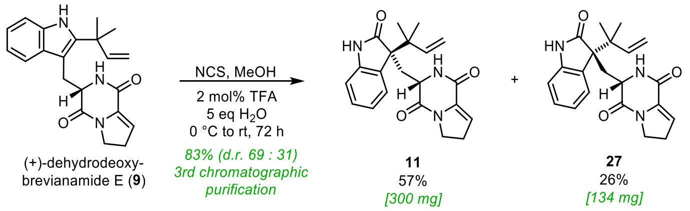

To a solution of (+)-dehydrodeoxy-brevianamide E (**9**) (500 mg, 1.43 mmol) in MeOH (30 mL) at 0 °C was added a solution of trifluoroacetic acid (2.2 µL, 0.029 mmol) in water (130 µL) with stirring, followed by addition of *N*-chlorosuccinimide (200 mg, 1.50 mmol). The reaction was allowed to warm slowly to room temperature and stirred for 72 h. The entire mixture was concentrated under reduced pressure. Flash column chromatography (2:8 to 13:7 MeCN/$CHCl_3$) gave major oxindole **11** (300 mg, 0.82 mmol, 57%) as an off-white foam and minor oxindole **27** (134 mg, 0.37 mmol, 26%) as a white foam.

Experimental procedure B

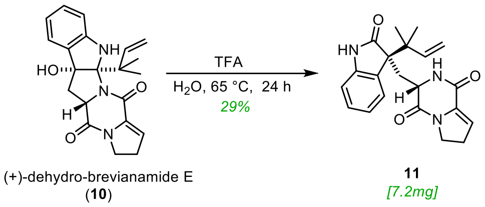

To a solution of (+)-dehydro-brevianamide E (**10**) (25 mg, 0.068 mmol) in H_2O (3.0 mL) was added trifluoroacetic acid (0.21 mL, 0.275 mmol). The reaction was heated at 65 °C for 24 h. The mixture was allowed to cool to room temperature and then quenched with $NaHCO_3$ (5 mL, sat., aq.) and extracted with $CHCl_3$ (5 × 10 mL), dried (Na_2SO_4) and concentrated under reduced pressure. Preparative TLC (7:3 $CHCl_3$/MeCN) gave oxindole **11** (7.2 mg, 0.020 mmol, 29%) as an off-white foam.

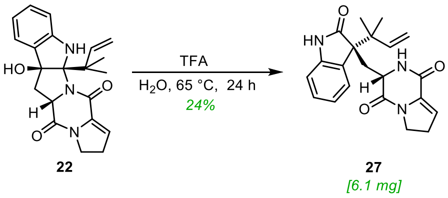

To a solution of **22** (25 mg, 0.068 mmol) in H_2O (3.0 mL) was added trifluoroacetic acid (0.21 mL, 0.275 mmol). The reaction was heated at 65 °C for 24 h. The mixture was allowed to cool to room temperature and then quenched with $NaHCO_3$ (5 mL, sat., aq.) and extracted with $CHCl_3$ (5 × 10 mL), dried (Na_2SO_4) and concentrated. Preparative TLC (7:3 $CHCl_3$/MeCN) gave oxindole **27** (6.1 mg, 0.017 mmol, 24%) as a white foam.

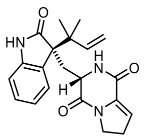

Major diastereomer:

R_f 0.11 (1:1 MeCN/$CHCl_3$);

^{1}H NMR (500 MHz, $CDCl_3$) δ 10.94 (br. s, 1H), 8.75 (br. s, 1H), 7.18 (dd, *J* = 7.6, 1.2 Hz, 1H), 7.11 (td, *J* = 7.7, 1.2 Hz, 1H), 6.94 – 6.85 (m, 2H), 6.08 (dd, *J* = 17.4, 10.8 Hz, 1H), 5.55 (app. t, *J* = 3.0 Hz, 1H), 5.10 (dd, *J* = 10.9, 1.2 Hz, 1H), 4.97 (dd, *J* = 17.4, 1.2 Hz, 1H), 4.31 (d, *J* = 7.2 Hz, 1H), 3.71 (app. t, *J* = 9.1 Hz, 2H), 3.03 (dd, *J* = 14.8, 1.5 Hz, 1H), 2.75 (dd, *J* = 14.8, 7.4 Hz, 1H), 2.51 – 2.43 (m, 2H), 1.11 (s, 3H), 0.97 (s, 3H) ppm;

^{13}C NMR (126 MHz, $CDCl_3$) δ 183.1, 162.6, 157.6, 143.1, 143.1, 132.1, 128.4, 128.0, 127.9, 120.5, 118.3, 114.2, 111.1, 56.3, 55.8, 45.2, 42.6, 33.7, 27.5, 22.2, 21.6 ppm;

IR (film, cm^{-1}) 3200, 3084, 2963, 2930, 1676, 1643, 1618, 1470, 1449;

HRMS (ESI^+) calc. for $C_{21}H_{23}N_3O_3$ ($[M + H]^+$): 366.1812; found: 366.1811; ($[M + Na]^+$): 388.1632; found: 388.1627;

$[\boldsymbol{\alpha}]_{\boldsymbol{D}}^{\mathbf{21.5}}$ +18.0 (*c* 0.20, MeOH).

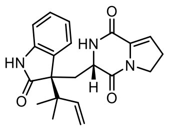

Minor diastereomer:

R_f 0.25 (1:1 MeCN/$CHCl_3$);

^{1}H NMR (500 MHz, $CDCl_3$) δ 10.42 (br. s, 1H), 7.58 (br. d, *J* = 3.7 Hz, 1H), 7.29 – 7.22 (m, 2H), 7.05 (td, *J* = 7.6, 1.1 Hz, 1H), 6.97 (dd, *J* = 8.2, 1.1 Hz, 1H), 6.06 (dd, *J* = 17.5, 10.8 Hz, 1H), 5.95 (app. t, *J* = 3.0 Hz, 1H), 5.13 (dd, *J* = 10.7, 1.1 Hz, 1H), 5.02 (dd, *J* = 17.4, 1.2 Hz, 1H), 4.05 (ddd, *J* = 12.3, 11.2, 5.4 Hz, 1H), 3.83 (ddd, *J* = 11.9, 11.8, 8.3 Hz, 1H), 3.56 (dt, *J* = 11.3, 3.2 Hz, 1H), 2.80 (dddd, *J* = 18.4, 11.2, 8.3, 2.8 Hz, 1H), 2.72 – 2.61 (m, 2H), 2.41 (dd, *J* = 14.0, 11.7 Hz, 1H), 1.15 (s, 3H), 1.05 (s, 3H) ppm;

^{13}C NMR (126 MHz, $CDCl_3$) δ 182.9, 163.6, 157.9, 142.7, 142.3, 133.3, 128.2, 127.9, 126.3, 121.9, 119.0, 114.6, 112.0, 56.7, 55.9, 45.7, 42.6, 38.9, 28.0, 22.0, 21.9 ppm;

IR (film, cm^{-1}) 3211, 2957, 2926, 1703, 1676, 1643, 1618, 1470, 1441;

HRMS (ESI^+) calc. for $C_{21}H_{23}N_3O_3$ ($[M + H]^+$): 366.1812; found: 366.1819; ($[M + Na]^+$): 388.1632; found: 388.1628;

$[\boldsymbol{\alpha}]_{\boldsymbol{D}}^{\mathbf{23.1}}$ −89.1 (*c* 0.19, MeOH).

1.20 Experimental Procedure for (+)-Brevianamide Y (4) and (+)-Brevianamide Z (5)

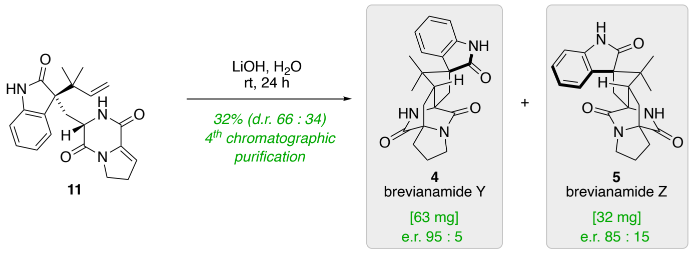

Oxindole **11** (300 mg, 0.82 mmol) was added to 1 M aq. LiOH (120 mL) at rt and the reaction was stirred for 24 h. The reaction was quenched with saturated aq. 1 M HCl (150 mL) and extracted with $CHCl_3$ (3 × 200 mL). The combined organics were dried (Na_2SO_4) and concentrated under reduced pressure. Preparative TLC (2:3 1,2-dimethoxyethane/MTBE) gave (+)-"brevianamide Z" (**5**) (32.4 mg, 0.089 mmol, 10.8%) as a white amorphous solid and (+)-brevianamide Y (**4**) (63.1 mg, 0.099 mmol, 21%) as a white amorphous solid.

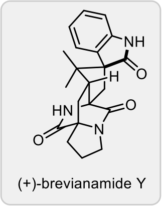

Spectroscopic data matched literature values.[1]

R$_f$ 0.15 (2:3 1,2-dimethoxyethane/MTBE);

^{1}H NMR (601 MHz, $(CD_3)_2SO$) δ 10.30 (br. s, 1H), 8.79 (br. s, 1H), 7.42 (dd, *J* = 7.6, 1.2 Hz, 1H), 7.19 (dd, *J* = 7.7, 1.2 Hz, 1H), 6.98 (app. td, *J* = 7.6, 1.1 Hz, 1H), 6.81 (dd, *J* = 7.7, 1.0 Hz, 1H), 3.36 – 3.22 (m, 2H), 3.17 (dd, *J* = 10.4, 7.2 Hz, 1H), 2.82 (d, *J* = 15.1 Hz, 1H), 2.46 (dd, *J* = 12.3, 6.6 Hz, 1H), 2.13 (d, *J* = 15.2 Hz, 1H), 2.02 – 1.96 (m, 1H), 1.93 (dd, *J* = 13.1, 10.4 Hz, 1H), 1.85 – 1.75 (m, 2H), 1.65* (dd, *J* = 13.0, 7.2 Hz, 1H), 0.99 (s, 3H), 0.69 (d, *J* = 2.1 Hz, 3H) ppm;

^{13}C NMR (126 MHz, $(CD_3)_2SO$) δ 181.8, 172.5, 169.0, 142.4, 129.8, 128.1, 126.3, 120.8, 109.0, 68.6, 67.1, 62.1, 50.0, 46.8, 43.2, 33.6, 28.4, 28.0, 24.4, 23.0, 20.4 ppm;

IR (film, cm^{-1}) 3225, 2970, 2928, 2872, 2359, 2324, 1719, 1694, 1668, 1595, 1470, 1445, 1404;

HRMS (ESI$^+$) calc. for $C_{21}H_{23}N_3O_3$ ($[M + H]^+$): 366.1812; found: 366.1813; ($[M + Na]^+$): 388.1632; found: 388.1624;

$[\alpha]_D^{26}$ +27.1 (*saturated*, MeOH) *attempts to prepare a solution of (c 0.2) resulted in a saturated solution*, literature: $[\alpha]_D^{25}$ +11.5 (*c* 0.2, MeOH),[1]

$[\alpha]_D^{23.3}$+10.9 (*c* 0.44, DMSO), literature: $[\alpha]_D^{25}$ +255.2 (*c* 0.37, DMSO) *we can't account for this discrepancy*.

e.r. 95:5 (Chiralpak IC, 42:58 EtOH/hexane, 1.0 mL min^{-1}, λ 254 nm) t_{Rmajor}= 11.06 min, t_{Rminor} = 14.66 min. See pages 50-52 for chiral HPLC traces.

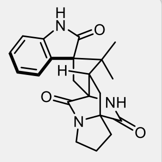

"(+)-brevianamide Z"

R_f 0.26 (2:3 1,2-dimethoxyethane/MTBE);

^{1}H NMR (500 MHz, $(CD_3)_2SO$) δ 10.27 (br. s, 1H), 8.69 (br. s, 1H), 7.17 (td, *J* = 7.6, 1.2 Hz, 1H), 7.06 (d, *J* = 7.4 Hz, 1H), 6.93 (td, *J* = 7.6, 1.1 Hz, 1H), 6.81 (dd, *J* = 7.7, 1.1 Hz, 1H), 3.40 (dt, *J* = 10.7, 6.8 Hz, 1H), 3.30 – 3.22 (m, 1H), 2.63 (dd, *J* = 10.1, 7.5 Hz, 1H), 2.59 (d, *J* = 14.9 Hz, 1H), 2.49 – 2.43 (m, 2H), 2.05 – 1.98 (m, 1H), 1.94 (dd, *J* = 13.0, 10.1 Hz, 1H), 1.89 – 1.75 (m, 2H), 1.66 (dd, *J* = 13.0, 7.4 Hz, 1H), 1.05 (s, 3H), 0.41 (s, 3H) ppm;

^{13}C NMR (126 MHz, $(CD_3)_2SO$) δ 178.6, 172.2, 169.4, 141.5, 134.3, 127.8, 124.0, 121.0, 109.1, 68.5, 67.5, 62.4, 53.0, 47.7, 43.3, 33.1, 28.4, 27.3, 24.4, 23.1, 19.5 ppm;

IR (film, cm^{-1}) 3235, 2961, 2930, 2880, 1686, 1678, 1618, 1470, 1395;

HRMS (ESI^{+}) calc. for $C_{21}H_{23}N_3O_3$ ($[M + H]^{+}$): 366.1812; found: 366.1816;

$[\boldsymbol{\alpha}]_{\boldsymbol{D}}^{24.1}$ +40.0 (*c* 0.13, DMSO);

e.r. 85:15 (Chiralpak IC, 25:75 EtOH/hexane, 1.0 mL min^{-1}, λ 254 nm) t_{Rmajor}= 19.73 min t_{Rminor} = 22.94 min. See pages 53-54 for chiral HPLC traces.

References:

1. Qi, S. Brevianamides and Mycophenolic Acid Derivatives from the Deep-Sea-Derived Fungus. *Mar. Drugs* **15,** *43* (2017).
2. Williams, R. M., Sherman, D. H. & Shengying, L. Fungal-derived brevianamide assembly by a Stereoselective Semipinacolase. *Nature Cat.* **3,** 497-506 (2020).

1.21 Experimental Procedure for (–)-Brevianamide Y (*ent*-4) and (–)-Brevianamide Z (*ent*-5)

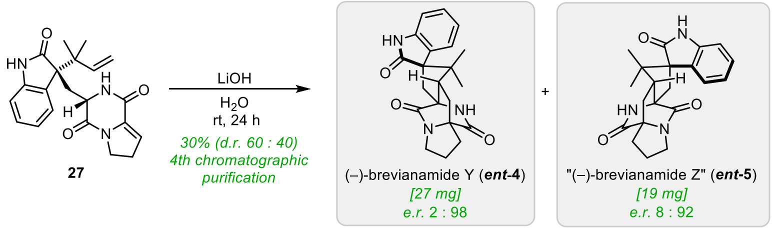

To a solution of **27** (150 mg, 0.41 mmol) at rt was added 1 M aq. LiOH (60 mL), and the reaction was stirred for 24 h. The reaction was quenched with saturated aq. 1 M HCl (75 mL) and extracted with $CHCl_3$ (3 × 100 mL). The combined organics were dried (Na_2SO_4) and concentrated under reduced pressure. Preparative TLC (2:3 1,2-dimethoxyethane/MTBE) gave compound "(–)-brevianamide Z" (***ent*-5)** (18.5 mg, 0.051 mmol, 12.0%) as a white amorphous solid and (–)-brevianamide Y (***ent*-4**) (27.4 mg, 0.075 mmol, 18.2%) as a white amorphous solid.

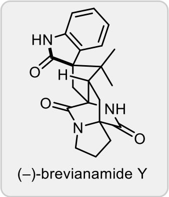

$[\alpha]_D^{23.0}$ –10.3 (*c* 0.39, DMSO);

e.r. 98:2 (Chiralpak IC, 42:58 EtOH/hexane, 1.0 mL min^{-1}, λ 254 nm) t_{Rmajor} = 11.59 min, t_{Rminor} = 14.42 min. See pages 50-52 for chiral HPLC traces.

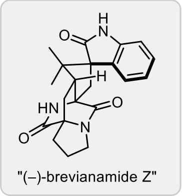

$[\alpha]_D^{24.2}$ –12.6 (*c* 0.19, DMSO);

e.r. 92:8 (Chiralpak IC, 25:75 EtOH/hexane, 1.0 mL min^{-1}, λ 254 nm) t_{Rmajor} = 20.01 min, t_{Rminor} = 22.98 min. See pages 53-54 for chiral HPLC traces.

1.22 Comparison of the ^{1}H and ^{13}C NMR Data for Synthetic and Natural (+)-Brevianamide Y (4)

Provided below is a tabulated comparison of the NMR data collected for synthetic (+)-brevianamide Y (4) versus the NMR data reported for the natural sample.[1]

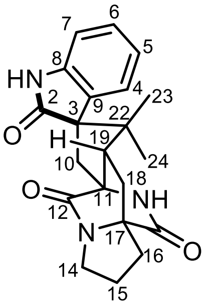

(–)-brevianamide Y

Atom	Synthetic brevianamide Y (4) $(CD_3)_2SO$			Natural brevianamide Y (4) $(CD_3)_2SO$				
	^{1}H δ (601 MHz)	m, *J* (Hz)	^{13}C δ (126 MHz)	^{1}H δ (500 MHz)	m, *J* (Hz)	^{13}C δ (125 MHz)	Δδ(^{1}H)	Δδ(^{13}C)
1	10.30	s	-	10.31	-	-	–0.01	-
2	-	-	181.8	-	-	182.3	-	–0.5
3	-	-	62.2	-	-	62.6	-	–0.4
4	7.42	dd, 7.6, 1.2	126.3	7.43	d, 17	126.8	–0.01	–0.5
5	6.98	td apt., 7.6, 1.1	120.8	6.99	dd, 7.5, 7.6	121.3	–0.01	–0.5
6	7.19	dd, 7.7, 1.2	128.1	7.20	dd, 7.6, 7.6	128.6	–0.01	–0.5
7	6.81	dd, 7.7,1.0	109.0	6.81	d, 7.6	109.5	0.00	–0.5
8	-	-	142.4	-	-	142.9	-	–0.5
9	-	-	129.8	-	-	130.2	-	–0.5
10	2.13	d, 15.2	33.6	2.14	d, 15.2	34.1	–0.01	–0.5
	2.82	d, 15.1	-	2.83	d, 15.2	-	–0.01	-
11	-	-	67.1	-	-	67.6	-	–0.5
12	-	-	169	-	-	169.5	-	–0.5
14	3.36-3.22	m	43.2	3.30	m	43.7	0.00	–0.5
15	1.85-1.75*	m	24.4	1.83	m	24.9	0.00	–0.5
	2.02-1.96	m	-	2.00	dd, 5.9, 12.1	-	0.00	-
16	1.85-1.75*	m	28.4	1.79†	m	28.9	0.00	–0.5
	2.46	dd, 12.3, 6.6	-	2.47†	dd, 6.4, 12.1	-	–0.01	-
17	-	-	68.6	-	-	69.1	-	–0.5
18	1.65†	dd, 13.0, 7.2†	28.0	1.79†	m	28.4	–0.14 (n/a)†	–0.5
	1.93†	dd, 13.1, 10.4†	-	2.47†	dd, 6.4, 12.1	-	–0.54 (n/a)†	-
19	3.17	dd, 7.2, 10.1	50.0	3.18	dd, 5.0, 10.0	50.5	–0.01	–0.5
20	-	-	172.5	-	-	173.0	-	–0.5
21	8.79	s	-	8.81	s	-	–0.02	-
22	-	-	46.8	-	-	47.3	-	–0.5
23	0.99	s	20.2	1.00	s	20.9	–0.01	–0.7
24	0.69	s	23.0	0.69	s	23.5	0.00	–0.5

The ^{1}H data are in good agreement, with all peaks found to be within ±0.02 ppm.

The ^{13}C data are in good agreement, with all peaks found to consistently vary by approximately –0.5 ppm, which is presumably a result of slightly different referencing.

† The literature table of ^{1}H NMR data doesn't include the peak at 1.65 ppm or 1.93 ppm. Instead, the table reports 'δ = 2.47, dd (6.4, 12.1)' and 'δ = 1.79, m' twice, which is presumably a typographical error.

* For the synthetic sample, the peak in the ^{1}H NMR at 1.85-1.75 ppm appears as a complex overlapping multiplet.

References:

1. Qi, S. Brevianamides and Mycophenolic Acid Derivatives from the Deep-Sea-Derived Fungus. *Mar. Drugs* **15**, *43* (2017).

1.23 Experimental Procedure for Compounds (±)-14 and (±)-6

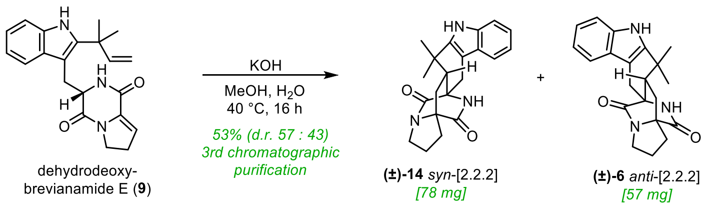

Compounds **(±)-6** and **(±)-14** were prepared based on a procedure reported by Williams and co-workers.[1]

To a solution of (+)-dehydrodeoxy-brevianamide E (**9**) (250 mg, 0.715 mmol) in MeOH (54.0 mL) was added 3.4 M aq. KOH (13.6 mL) and the reaction stirred at 40 °C for 16 h. The reaction was cooled to room temperature, quenched with saturated aq. NH_4Cl (25 mL) and extracted with CH_2Cl_2 (5 × 50 mL) and $CHCl_3$ (2 × 50 mL). The combined extracts were dried (Na_2SO_4) and concentrated under reduced pressure. Flash column chromatography (1:9 to 2:8 MeCN/$CHCl_3$) gave compound **(±)-14** (77.7 mg, 0.222 mmol, 31%) as a white foam and compound **(±)-6** (57.2 mg, 0.164 mmol, 23%) as a white foam.

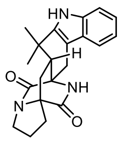

R_f 0.14 (2:8 MeCN/$CHCl_3$);

^{1}H NMR (500 MHz, $CDCl_3$) δ 7.79 (br. s, 1H), 7.53 (d, *J* = 7.8 1H), 7.33 (dt, *J* = 7.9, 0.9 Hz, 1H), 7.19 (ddd, *J* = 8.1, 7.1, 1.3 Hz, 1H), 7.13 (ddd, *J* = 8.0, 7.1, 1.0 Hz, 1H), 5.65 (br. s, 1H), 3.95 (d, *J* = 17.9 Hz, 1H), 3.56 (td, *J* = 7.1, 1.6 Hz, 2H), 2.92 (d, *J* = 17.9 Hz, 1H), 2.84 (ddd, *J* = 13.2, 7.1, 6.2 Hz, 1H), 2.37 (ddd, *J* = 9.9, 4.2, 0.9 Hz, 1H), 2.17 (dd, *J* = 13.5, 9.9 Hz, 1H), 2.10 (dd, *J* = 13.6, 4.2 Hz, 1H), 2.09 – 1.99 (m, 2H), 1.88 (dt, *J* = 13.1, 7.5 Hz, 1H), 1.34 (s, 3H), 1.31 (s, 3H) ppm;

^{13}C NMR (126 MHz, $CDCl_3$) δ 172.6, 169.0, 139.6, 136.4, 127.2, 122.2, 119.8, 118.4, 110.7, 103.6, 67.1, 61.7, 45.8, 44.2, 34.5, 32.7, 29.2, 29.1, 25.4, 24.5, 24.0 ppm;

IR (film, cm^{-1}) 3447, 3217, 2955, 2911, 2359, 1697, 1655, 1460, 1435;

HRMS (ESI^+) calc. for $C_{21}H_{23}N_3O_2$ ($[M + H]^+$): 350.1863; found: 350.1856; ($[M + Na]^+$): 372.1682; found: 372.1675).

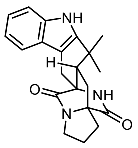

R_f 0.08 (2:8 MeCN/$CHCl_3$);

^{1}H NMR (500 MHz, $CDCl_3$) δ 7.74 (br. s, 1H), 7.51 (app. ddt, *J* = 7.7, 1.4, 0.7 Hz, 1H), 7.29 (dt, *J* = 8.0, 0.9 Hz, 1H), 7.16 (ddd, *J* = 8.1, 7.1, 1.3 Hz, 1H), 7.11 (ddd, *J* = 8.1, 7.1, 1.1 Hz, 1H), 5.93 (br. s, 1H), 3.90 (d, *J* = 15.2 Hz, 1H), 3.57 (dt, *J* = 11.4, 6.4 Hz, 1H), 3.41 (dt, *J* = 11.4, 7.2 Hz, 1H), 2.83 (dt, *J* = 13.1, 6.6 Hz, 1H), 2.64 (d, *J* = 15.3 Hz, 1H), 2.61 (dd, *J* = 10.4,

4.9 Hz, 1H), 2.26 (dd, J = 13.5, 10.3 Hz, 1H), 2.04 (app. p, J = 7.1 Hz, 2H), 1.97 (dd, J = 13.5, 4.9 Hz, 1H), 1.89 (dt, J = 13.0, 7.6 Hz, 1H), 1.32 (s, 3H), 1.13 (s, 3H) ppm;

^{13}C NMR (126 MHz, $CDCl_3$) δ 173.3, 168.6, 139.6, 136.6, 127.1, 122.1, 119.7, 118.6, 110.7, 104.8, 66.7, 60.6, 49.7, 44.3, 34.8, 31.2, 29.6, 28.5, 25.5, 24.7, 22.5 ppm;

IR (film, cm^{-1}) 3287, 3163, 3119, 3055, 2968, 2889, 2864, 1680, 1661, 1458, 1429, 1416, 1395, 1375;

HRMS (ESI^+) *m/z* 350.1859 (calculated $[M + H]^+$ 350.1863), 372.1669 (calculated $[M + Na]^+$ 372.1682).

References:

1. Greshock, T. J. & Williams, R. M. Improved Biomimetic Total Synthesis of D,L-stephacidin A. *Org. Lett.* **9** (21), 4255-8 (2007).

1.24 Experimental Procedure for (±)-Brevianamide X (3)

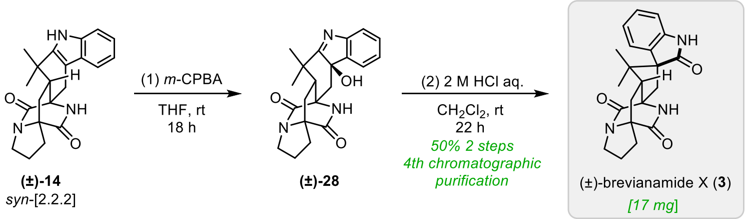

(±)-Brevianamide X (**3**) was prepared under anhydrous conditions, adapted from the work of Williams and co-workers.[1, 2]

***Step (1)* -** To a solution of compound **14** (32.9 mg, 94.2 µmol) in THF (5.7 mL) was added a solution of *m*-CPBA in THF (0.021 M, 5.7 mL, 0.12 mmol).* The reaction mixture was stirred at room temperature for 18 h before being quenched with DMS (50 µL) and concentrated under reduced pressure. The crude material was used directly in the next step.

Step (2) - The crude product from the first step was dissolved in CH_2Cl_2 (5.7 mL), 2 M aq. HCl (5.7 mL) was added and the reaction stirred rapidly at room temperature for 22 h. The reaction mixture was diluted with brine (5 mL), extracted with CH_2Cl_2 (4 × 10 mL) and the combined organics concentrated under reduced pressure. Column chromatography (EtOAc) followed by preparative TLC (2:3 MeCN/$CHCl_3$) gave brevianamide X (**3**) (17.3 mg, 47.3 µmol, 50%) as an amorphous white solid. Spectroscopic data matched literature values.[3]

**m*-CPBA (69% w/w by iodometric titration, 107 mg, 0.429 mmol) was added to THF (20 mL) and dried over Na_2SO_4 (6.6 g) with stirring for 20 minutes.

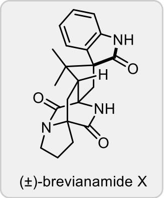

R_f 0.11 (2:3 MeCN/$CHCl_3$);

^{1}H NMR (500 MHz, $(CD_3)_2SO$) δ 10.34 (br. s, 1H), 9.09 (br. s, 1H), 7.23 (dd, *J* = 7.6, 1.2 Hz, 1H), 7.18 (dd, *J* = 7.7, 1.2 Hz, 1H), 6.97 (dd, *J* = 7.6, 1.1 Hz, 1H), 6.82 (dd, *J* = 7.8, 1.0 Hz, 1H), 3.45 – 3.34 (m, 2H), 3.22 (dd, *J* = 10.3, 8.2 Hz, 1H), 2.85 (d, *J* = 14.2 Hz, 1H), 2.55 – 2.51 (m, 1H), 2.19 (d, *J* = 14.2 Hz, 1H), 2.05 – 1.98 (m, 1H), 1.94 (dd, *J* = 13.0, 10.3 Hz, 1H), 1.85 – 1.72 (m, 3H), 0.74 (s, 3H), 0.71 (s, 3H) ppm;

^{13}C NMR (126 MHz, $(CD_3)_2SO$) δ 182.3, 173.0, 169.4, 142.3, 130.5, 128.0, 125.9, 121.0, 109.2, 68.1, 65.6, 61.4, 55.4, 45.1, 43.4, 33.1, 29.4, 29.0, 24.3, 23.2, 19.7 ppm;

IR (film, cm^{-1}) 3155, 3088, 2951, 2880, 1699, 1661, 1616, 1471, 1398;

HRMS (ESI$^+$) calc. for $C_{21}H_{23}N_3O_3$ ($[M + H]^+$): 366.1812; found: 366.1801; ($[M + Na]^+$): 388.1632; found: 388.1614.

References:

1. Williams, R. M. Asymmetric, Stereocontrolled Total Synthesis of (–)-brevianamide B. *J. Am. Chem. Soc.* **112** (2), 808-821 (1990).

2. Greshock, T. J. & Williams, R. M. Improved Biomimetic Total Synthesis of D,L-stephacidin A. *Org. Lett.* **14** (24), 6377-6378 (2012).

3. Qi, S. Brevianamides and Mycophenolic Acid Derivatives from the Deep-Sea-Derived Fungus. *Mar. Drugs* **15** (2), *43* (2017).

1.25 Comparison of the ^{1}H and ^{13}C NMR Data for Synthetic and Natural (±)-Brevianamide X (3)

Provided below is a tabulated comparison of the NMR data collected for synthetic (±)-brevianamide X (**3**) versus the NMR data reported for the natural sample.[1]

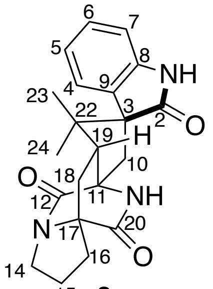

3
(±)-brevianamide X

Atom	Synthetic brevianamide X (3) $(CD_3)_2SO$			Natural brevianamide X (3) $(CD_3)_2SO$				
	^{1}H δ (500 MHz)	m, *J* (Hz)	^{13}C δ (126 MHz)	^{1}H δ (500 MHz)	m, *J* (Hz)	^{13}C δ (125 MHz)	Δδ(^{1}H)	Δδ(^{13}C)
1	10.34	s	-	10.36	s	-	–0.02	-
2	-	-	182.3	-	-	182.8	-	–0.5
3	-	-	61.4	-	-	61.9	-	–0.5
4	7.23	dd, 7.6, 1.2	125.9	7.23	d, 7.5	126.1	0.00	–0.2
5	6.97	dd, 7.6, 1.1	121.0	6.98	dd, 7.5, 7.6	121.5	–0.01	–0.5
6	7.18	dd, 7.7, 1.2	128.0	7.20	dd, 7.6, 7.6	128.3	–0.02	–0.3
7	6.82	dd, 7.8, 1.0	109.2	6.83	d, 7.6	109.6	–0.01	–0.4
8	-	-	142.3	-	-	142.8	-	–0.5
9	-	-	130.5	-	-	131.0	-	–0.5
10	2.19	d, 14.2	33.1	2.20	d, 14.2	33.6	–0.01	–0.5
	2.85	d, 14.2	-	2.86	d, 14.2	-	–0.01	-
11	-	-	65.6	-	-	66.1	-	–0.5
12	-	-	169.4	-	-	169.8	-	–0.4
14	3.45-3.34	m	43.4	3.41	m	43.8	0.00	–0.4
15	*1.85-1.72	m	24.3	1.80	m	24.8	0.00	–0.5
	2.05-1.98	m	-	1.99	m	-	0.00	-
16	2.55-2.51	m	29.0	2.50	overlapped	29.5	+0.01	–0.5
	*1.85-1.72	m	-	-	-	-		-
17	-	-	68.1	-	-	68.5	-	–0.4
18	*1.85-1.72	m	29.4	1.78	dd, 8.2, 12.8	29.9	0.00	–0.5
	1.94	dd, 13.0, 10.3	-	1.93	dd, 10.2, 12.9	-	+0.01	-
19	3.22	dd, 8.2, 10.3	55.4	3.23	dd, 8.3, 10.1	55.9	–0.01	–0.5
20	-	-	173.0	-	-	173.5	-	–0.5
21	9.09	s	-	9.13	s	-	–0.04	-
22	-	-	45.1	-	-	45.6	-	–0.5
23	0.74	s	19.7	0.74	s	20.2	0.00	–0.5
24	0.71	s	23.2	0.72	s	23.7	–0.01	–0.5

The ^{1}H data are in good agreement, with all peaks found to be within ±0.04 ppm.

The ^{13}C data are in good agreement, with all peaks found to consistently vary by approximately –0.5 ppm, which is presumably a result of slightly different referencing.

* For the synthetic sample, the peak in the ^{1}H NMR at 1.85-1.72 ppm appears as a complex overlapping multiplet.

References:

1. Qi, S. Brevianamides and Mycophenolic Acid Derivatives from the Deep-Sea-Derived Fungus. *Mar. Drugs* **15**, *43* (2017).

2 Chiral HPLC Chromatograms

2.1 Chiral HPLC Chromatograms of Brevianamide A (**1**)

Mix of (+) & (–)-brevianamide A (~**(±)-1**)
(Chiralpak IA, 1:1 *i*-PrOH/hexane, 1 mL min^{-1}, λ 254 nm)
t_{R1} = 5.40 min, t_{R2} = 6.74 min.

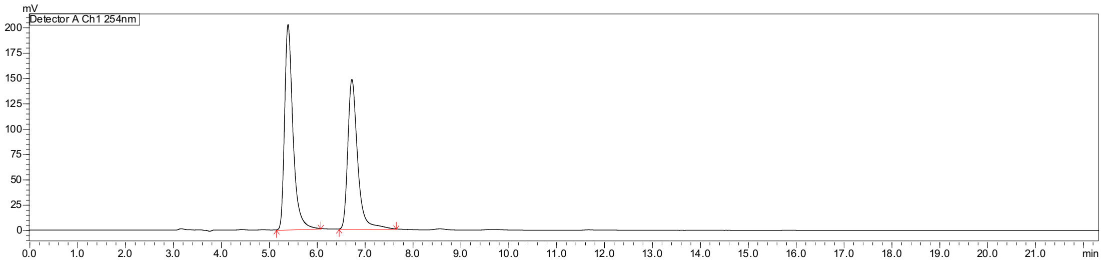

Peak #	Ret. Time	Area	Height	Conc.	Area %
1	5.402	2456350	202003	53.925	53.925
2	6.735	2098773	147492	46.075	46.075
Total		4555123	349495	100.000	100.000

(+)-brevianamide A (**1**) **e.r.** 93:7
(Chiralpak IA, 1:1 *i*-PrOH/hexane, 1 mL min^{-1}, λ 254 nm)
t_{Rminor} = 5.41 min, t_{Rmajor} = 6.72 min.

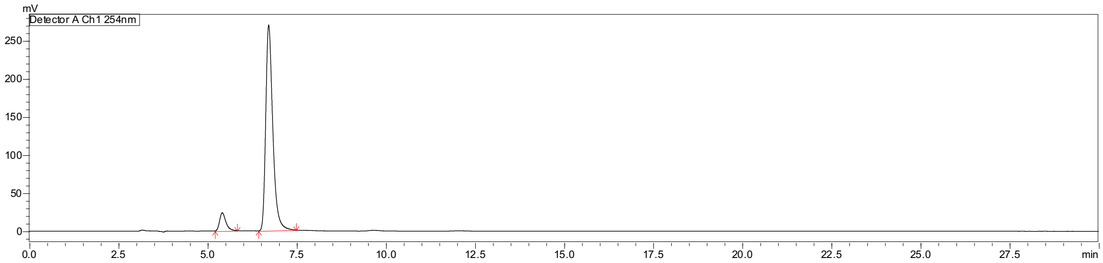

Peak #	Ret. Time	Area	Height	Conc.	Area %
1	5.414	289622	23737	7.232	7.232
2	6.717	3715018	269840	92.768	92.768
Total		4004641	293577	100.000	100.000

(+)-brevianamide A (**1**) **e.r.** 99:1 *after crystallisation*
(Chiralpak IA, 1:1 *i*-PrOH/hexane, 1 mL min^{-1}, λ 254 nm)
t_{Rminor} = 5.51 min, t_{Rmajor} = 6.84 min.

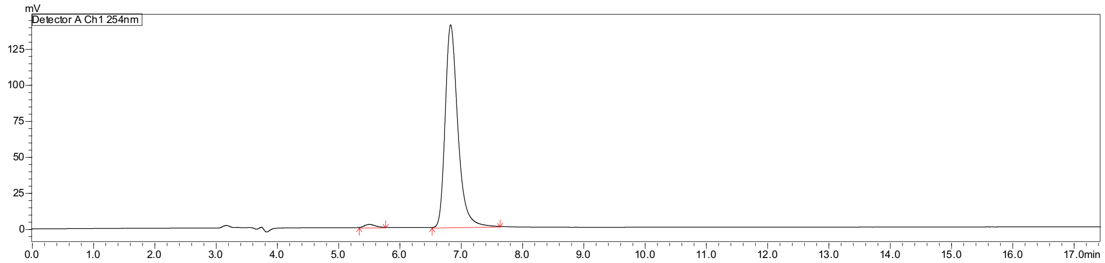

Peak #	Ret. Time	Area	Height	Conc.	Area %
1	5.507	24842	2112	1.220	1.220
2	6.835	2010703	140355	98.780	98.780
Total		2035545	142469	100.000	100.000

(–)-brevianamide A (***ent*-1**) **e.r.** 95:5
(Chiralpak IA, 1:1 *i*-PrOH/hexane, 1 mL min^{-1}, λ 254 nm)
t_{Rmajor} = 5.38 min, t_{Rminor} = 6.74 min.

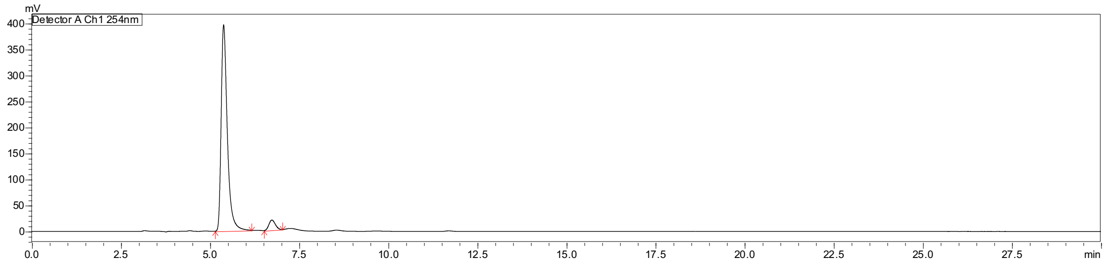

Peak #	Ret. Time	Area	Height	Conc.	Area %
1	5.383	4758681	396107	95.170	95.170
2	6.738	241511	19400	4.830	4.830
Total		5000192	415507	100.000	100.000

2.2 Chiral HPLC Chromatograms of Brevianamide B (**2**)

Mix of (+) & (–)-brevianamide B (~**(±)-2)**)
(Chiralpak IA, 1:3 EtOH/hexane, 1 mL min^{-1}, λ 254 nm)
t_{R1} = 13.86 min, t_{R2} = 26.03 min.

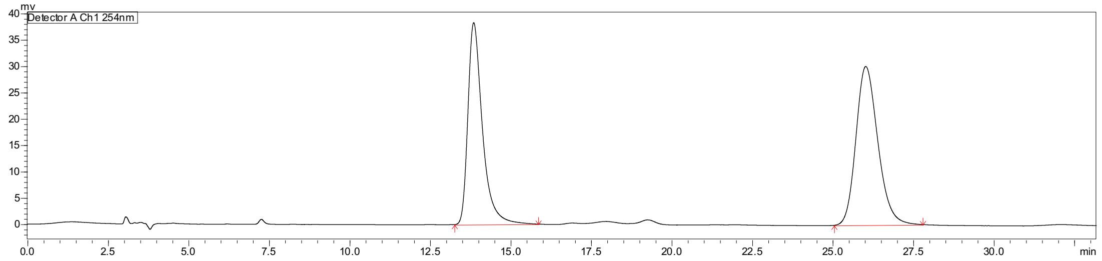

Peak #	Ret. Time	Area	Height	Conc.	Area %
1	13.860	1202069	38310	45.782	45.782
2	26.032	1423551	30114	54.218	54.218
Total		2625620	68424	100.000	100.000

(+)-brevianamide B (**2**) **e.r.** 93:7
(Chiralpak IA, 1:3 EtOH/hexane, 1 mL min^{-1}, λ 254 nm)
t_{Rminor} = 13.99 min, t_{Rmajor} = 26.10 min.

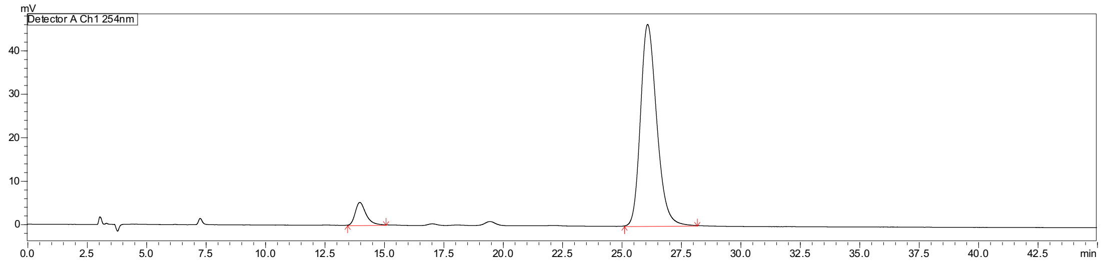

Peak #	Ret. Time	Area	Height	Conc.	Area %
1	13.987	162378	5219	6.875	6.875
2	26.097	2199609	46337	93.125	93.125
Total		2361987	51556	100.000	100.000

(–)-brevianamide B (***ent*-2**) **e.r.** 92:8
(Chiralpak IA, 1:3 EtOH/hexane, 1 mL min^{-1}, λ 254 nm)
t_{Rmajor} = 13.86 min, t_{Rminor} = 26.14 min.

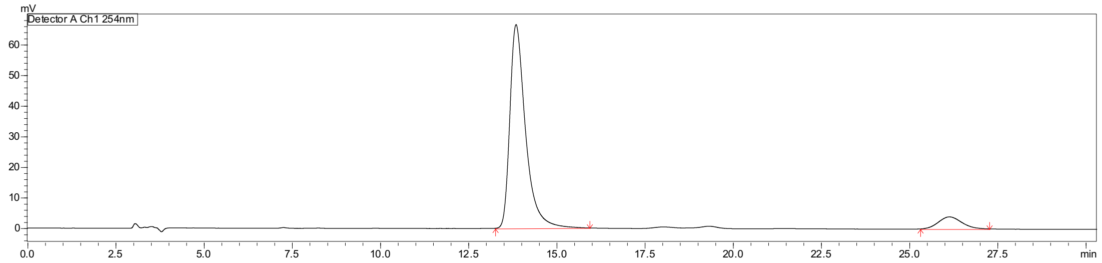

Peak #	Ret. Time	Area	Height	Conc.	Area %
1	13.856	2058078	66555	91.955	91.955
2	26.142	180065	3929	8.045	8.045
Total		2238144		100.000	100.000

2.3 Chiral HPLC Chromatograms of Brevianamide Y (**4**)

Mux of (+) & (–)-brevianamide Y (~**(±)-4)**
(Chiralpak IC, 42:58 EtOH/hexane, 1.0 mL min^{-1}, λ 254 nm)
t_{R1} = 11.26 min, t_{R2} = 14.52 min.

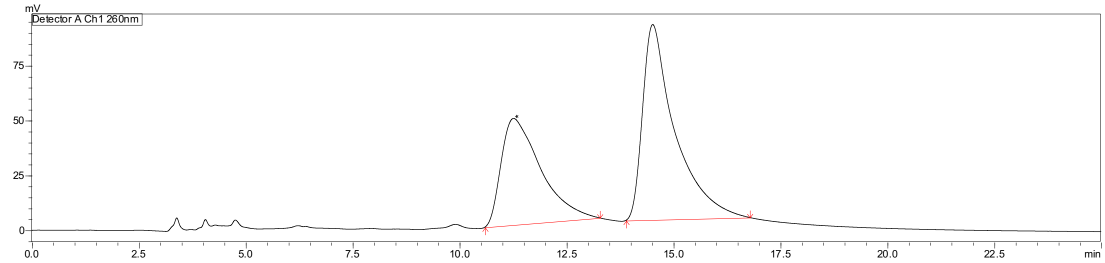

Peak #	Ret. Time	Area	Height	Conc.	Area %
1	11.264	3110505	48513	39.948	39.948
2	14.516	4675856	88917	60.052	60.052
Total		7786361	137430	100.000	100.000

(+)-brevianamide Y (**4**) **e.r.** 95:5
(Chiralpak IC, 42:58 EtOH/hexane, 1.0 mL min^{-1}, λ 254 nm)
t_{Rmajor} = 11.06 min, t_{Rminor} = 14.66 min.

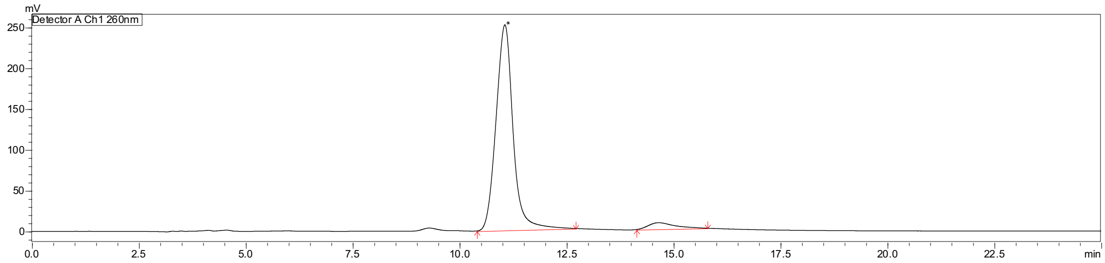

Peak #	Ret. Time	Area	Height	Conc.	Area %
1	11.059	6961488	251749	95.367	95.367
2	14.657	338197	7868	4.633	4.633
Total		7299684	259617	100.000	100.000

(+)-brevianamide Y (**4**) **e.r.** 98:2* liquor after recrystalisation
(Chiralpak IC, 42:58 EtOH/hexane, 1.0 mL min^{-1}, λ 254 nm)
t_{Rmajor} = 12.66 min, t_{Rminor} = 15.78 min.

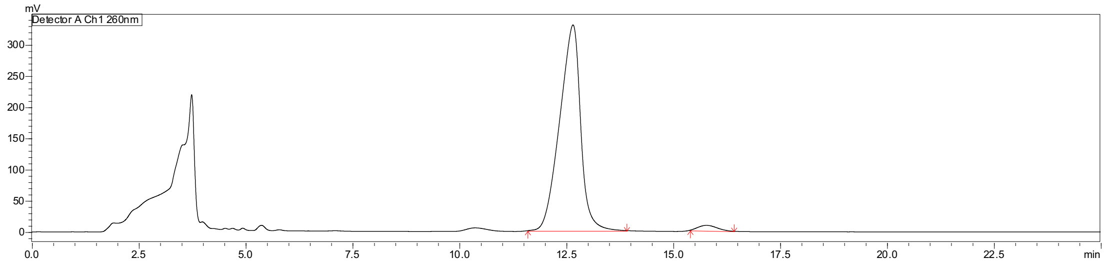

Peak #	Ret. Time	Area	Height	Conc.	Area %
1	12.658	11230063	329769	97.732	97.732
2	15.781	260570	8559	2.268	2.268
Total		11490633	338327	100.000	100.000

brevianamide Y (**(±)-4**) **e.r.** 50:50* prism crystals
(Chiralpak IC, 42:58 EtOH/hexane, 1.0 mL min^{-1}, λ 254 nm)
t_{R2} = 12.37 min, t_{R2} = 15.78 min.

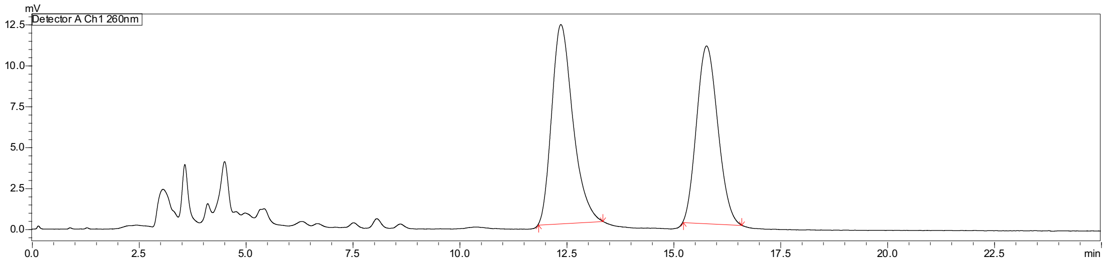

Peak #	Ret. Time	Area	Height	Conc.	Area %
1	12.372	390639	11786	50.286	50.286
2	15.778	386190	10996	49.714	49.714
Total		776829	22782	100.000	100.000

(–)-brevianamide Y (***ent*-4**) **e.r.** 98:2
(Chiralpak IC, 42:58 EtOH/hexane, 1.0 mL min^{-1}, λ 254 nm)
t_{Rminor} = 11.59 min, t_{Rmajor} = 14.42 min.

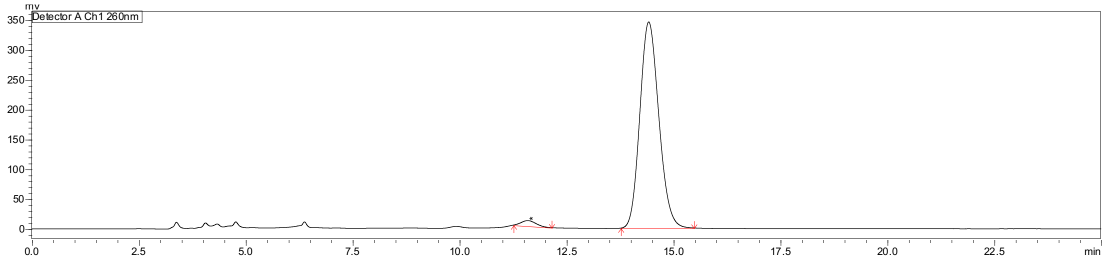

Peak #	Ret. Time	Area	Height	Conc.	Area %
1	11.594	217087	9163	1.997	1.997
2	14.422	10654930	345601	98.003	98.003
Total		10872017	354764	100.000	100.000

2.4 Chiral HPLC Chromatograms of Brevianamide Z (**5**)

Mix of (+) & (–)-brevianamide Z (~**(±)-5**)
(Chiralpak IC, 42:58 EtOH/hexane, 1.0 mL min^{-1}, λ 254 nm)
t_{R1} = 20.13 min, t_{R2} = 22.87 min.

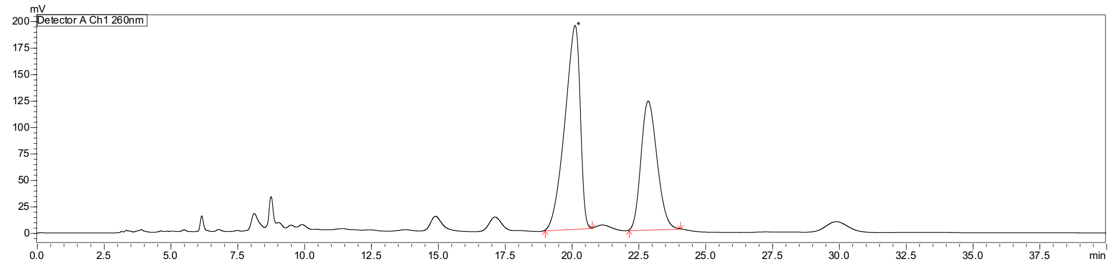

Peak #	Ret. Time	Area	Height	Conc.	Area %
1	20.134	7655394	192056	60.169	60.169
2	22.868	5067767	121468	39.831	39.831
Total		12723161	313524	100.000	100.000

(+)-brevianamide Z (**5**) **e.r.** 85:15
(Chiralpak IC, 25:75 EtOH/hexane, 1.0 mL min^{-1}, λ 254 nm)
t_{Rminor} = 19.73 min, t_{Rmajor} = 22.94 min.

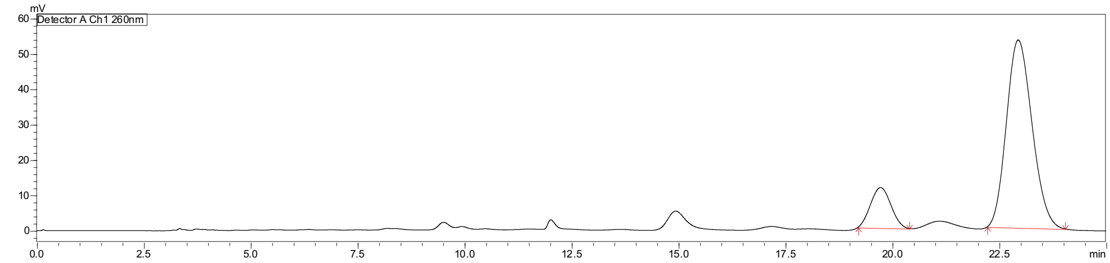

Peak #	Ret. Time	Area	Height	Conc.	Area %
1	19.726	376405	11409	14.516	14.516
2	22.944	2216619	53113	85.484	85.484
Total		2593024	64522	100.000	100.000

(–)-brevianamide Z (***ent*-5**) **e.r.** 92:8
(Chiralpak IC, 25:75 EtOH/hexane, 1.0 mL min^{-1}, λ 254 nm)
t_{Rmajor} = 20.01 min, t_{Rminor} = 22.98 min.

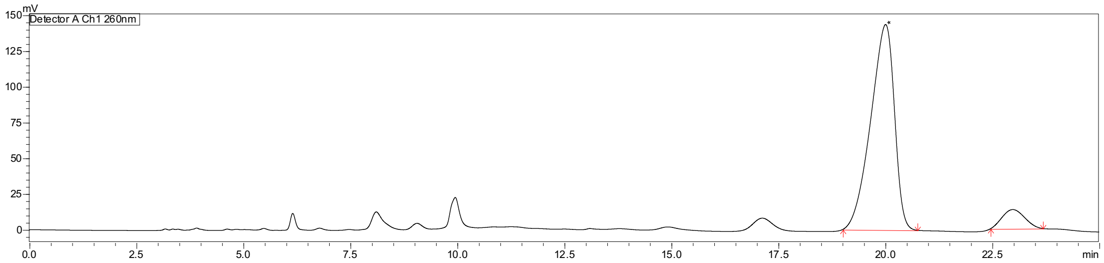

Peak #	Ret. Time	Area	Height	Conc.	Area %
1	20.009	5538964	143716	91.868	91.868
2	22.981	490327	13398	8.132	8.132
Total		6029291	157114	100.000	100.000

3 NMR Spectra

3.1 ^{1}H NMR Spectrum of Compound **16** (500 MHz, $CDCl_3$)

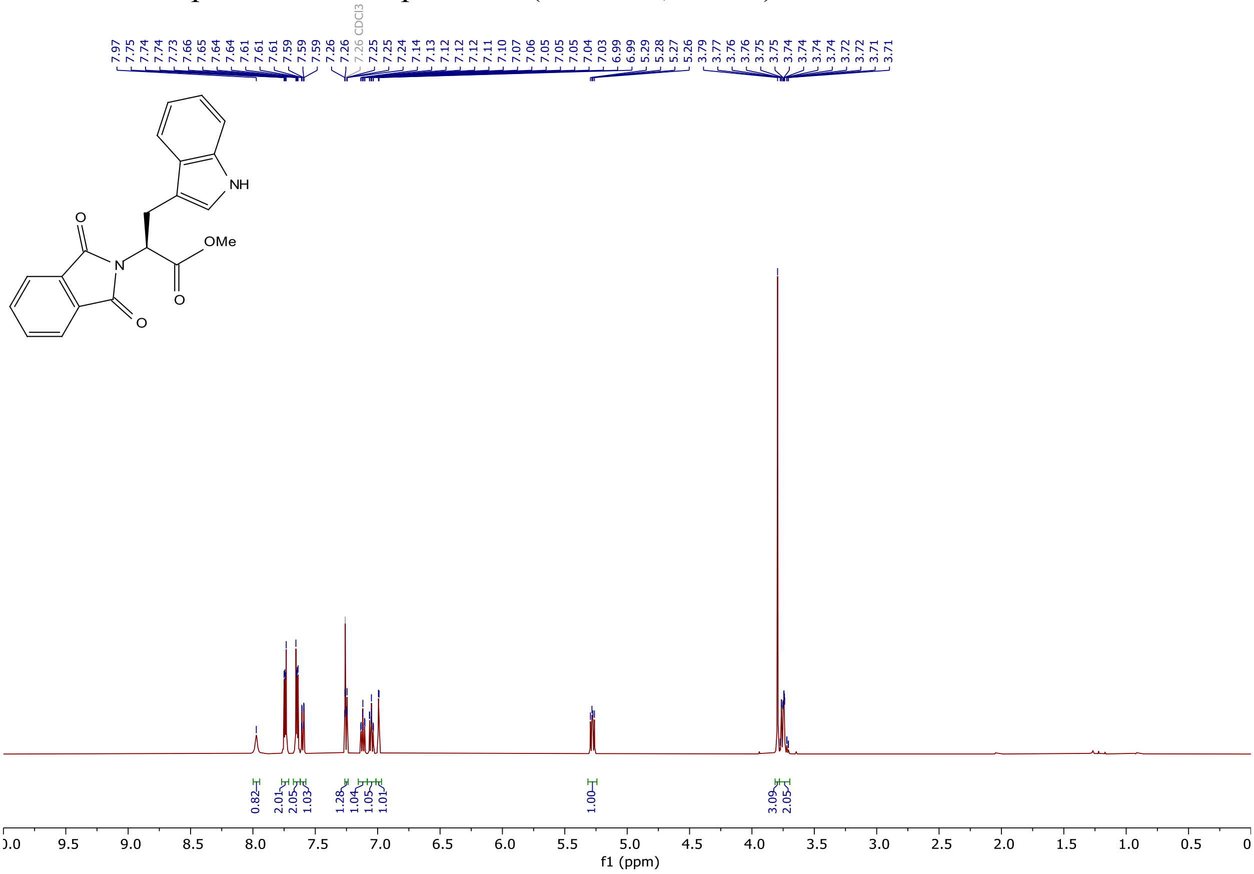

3.2 ^{13}C NMR Spectrum of Compound **16** (126 MHz, $CDCl_3$)

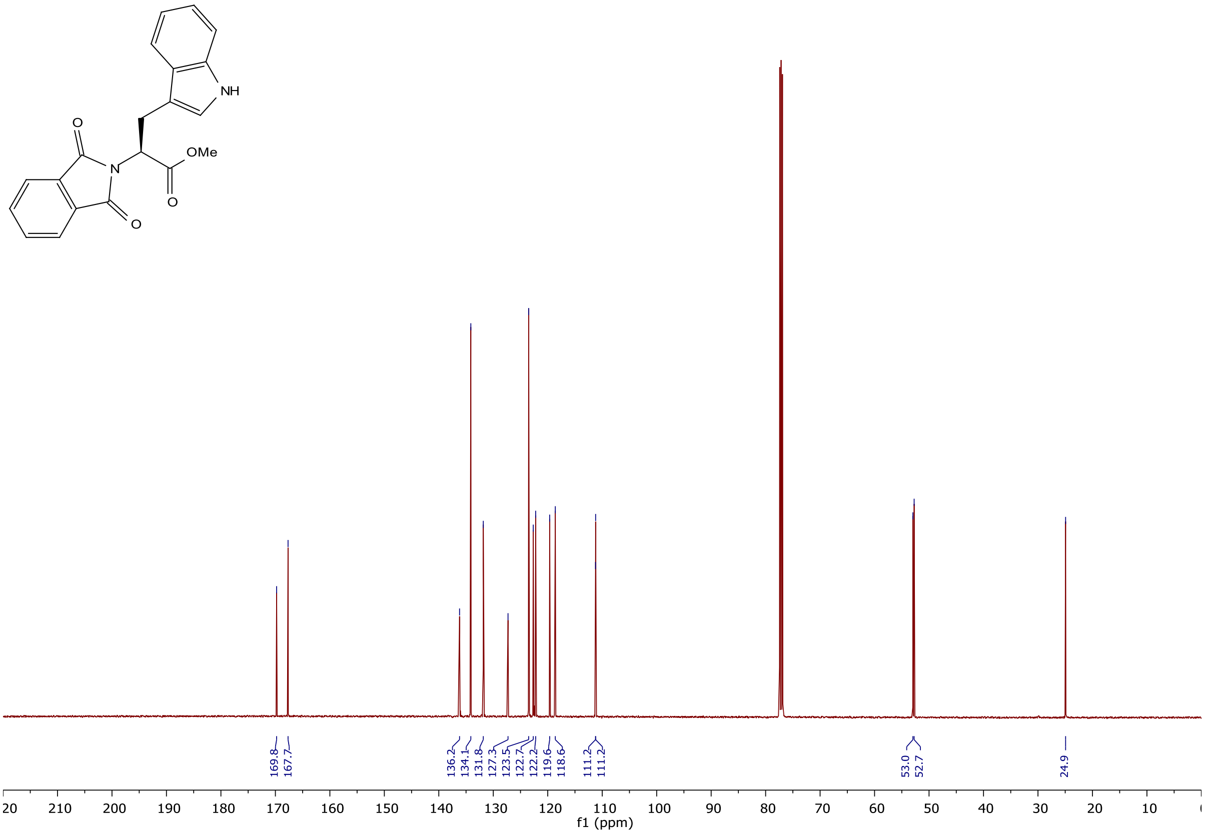

3.3 ^{1}H NMR Spectrum of Compound **17** (600 MHz, $CDCl_3$)

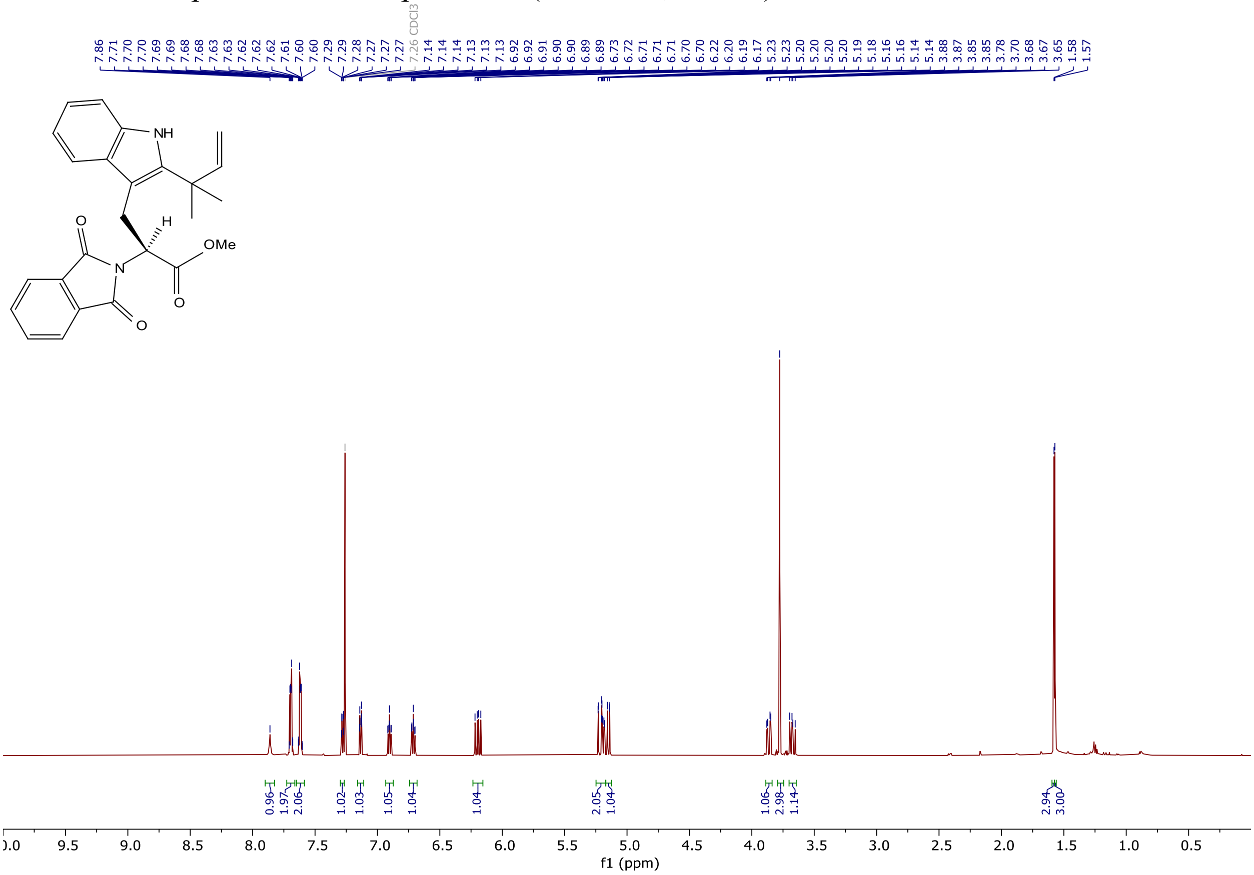

3.4 ^{13}C NMR Spectrum of Compound **17** (151 MHz, $CDCl_3$)

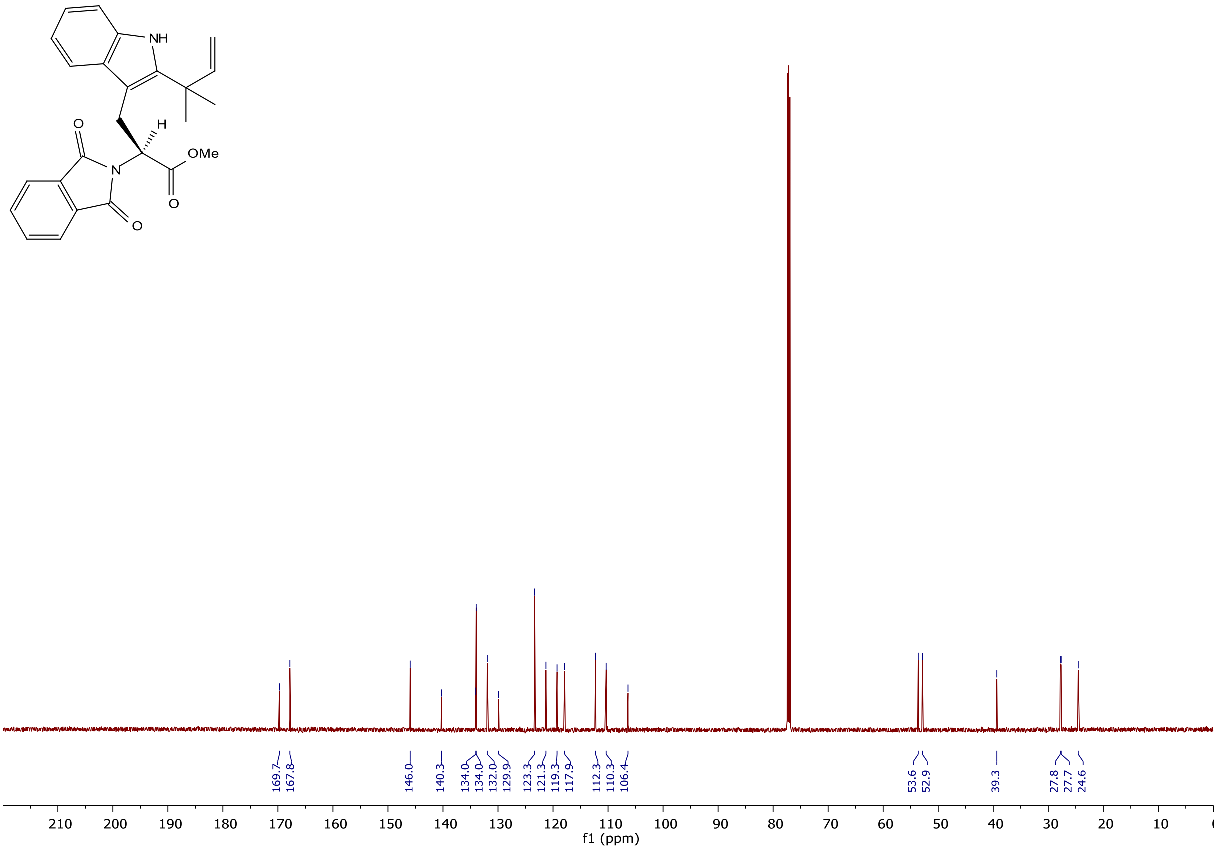

3.5 ^{1}H NMR Spectrum of Compound **18** (500 MHz, CD_3OD)

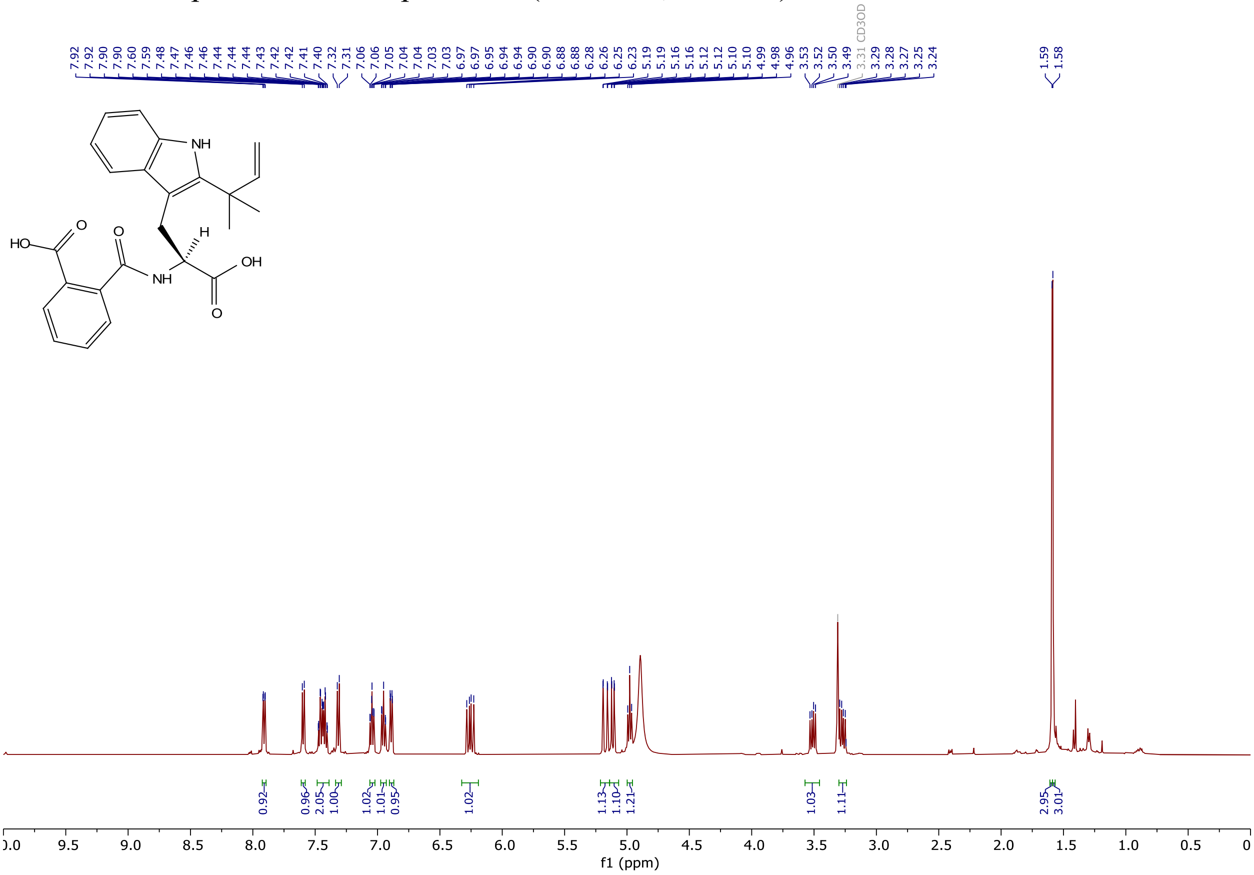

3.6 ^{13}C NMR Spectrum of Compound **18** (126 MHz, CD_3OD)

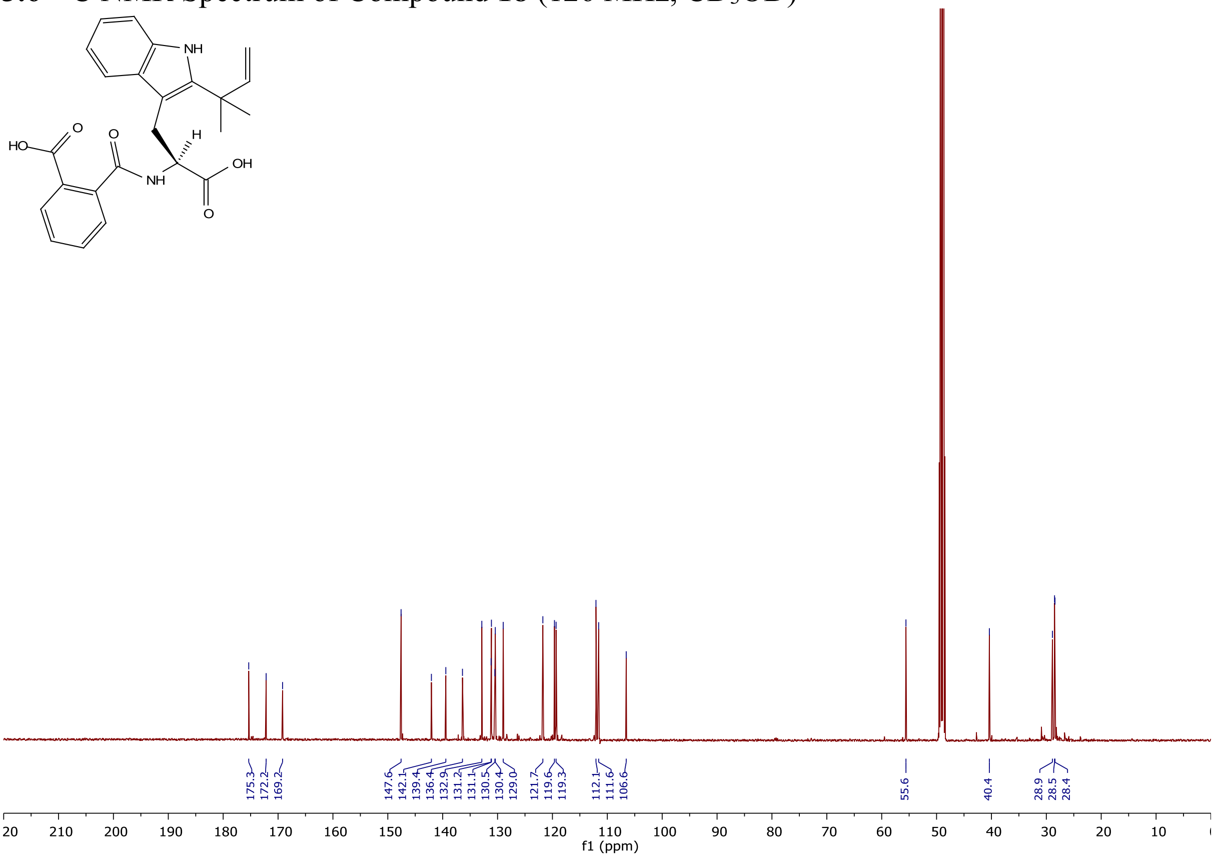

3.7 ^{1}H NMR Spectrum of Compound **19** (500 MHz, CD_3OD)

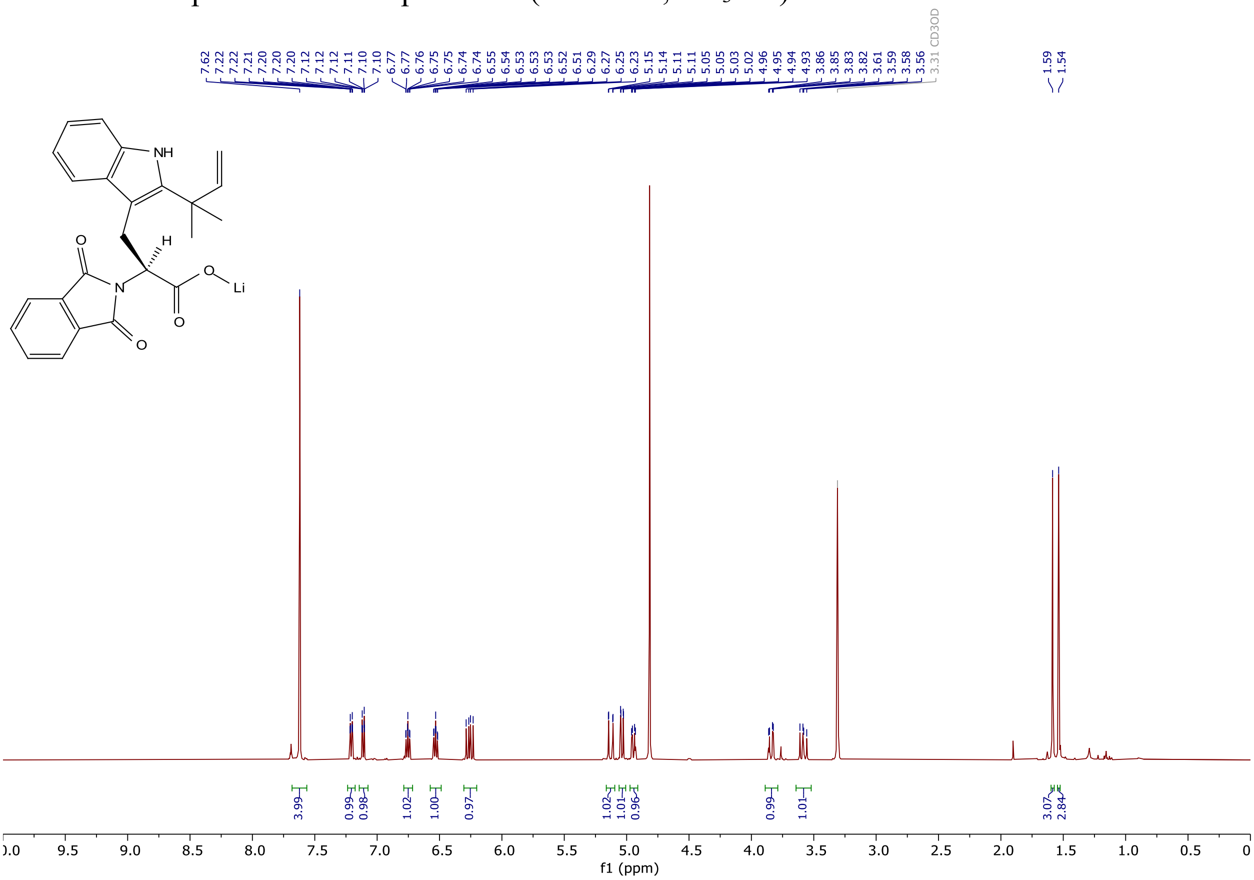

3.8 ^{13}C NMR Spectrum of Compound **19** (126 MHz, CD_3OD)

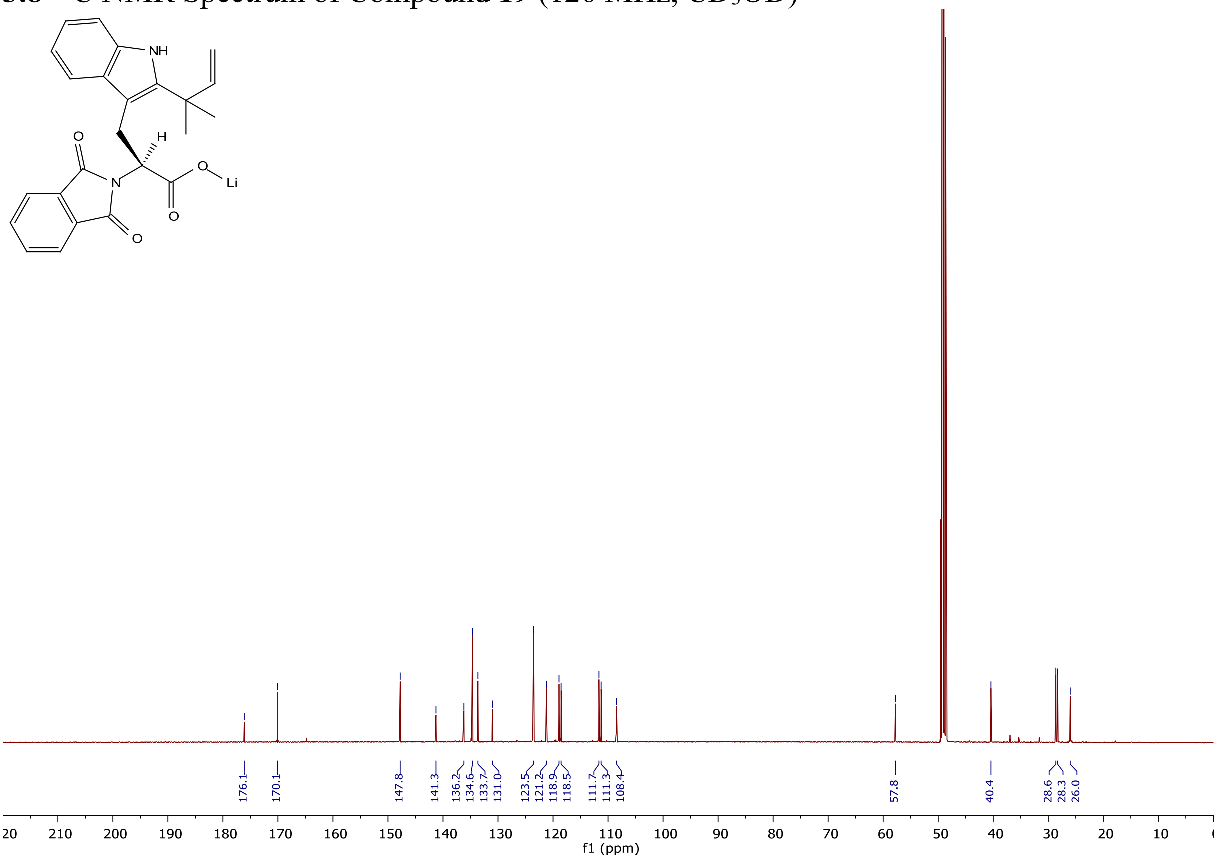

3.9 ^{1}H NMR Spectrum of Compound **21** (500 MHz, CD_2Cl_2)

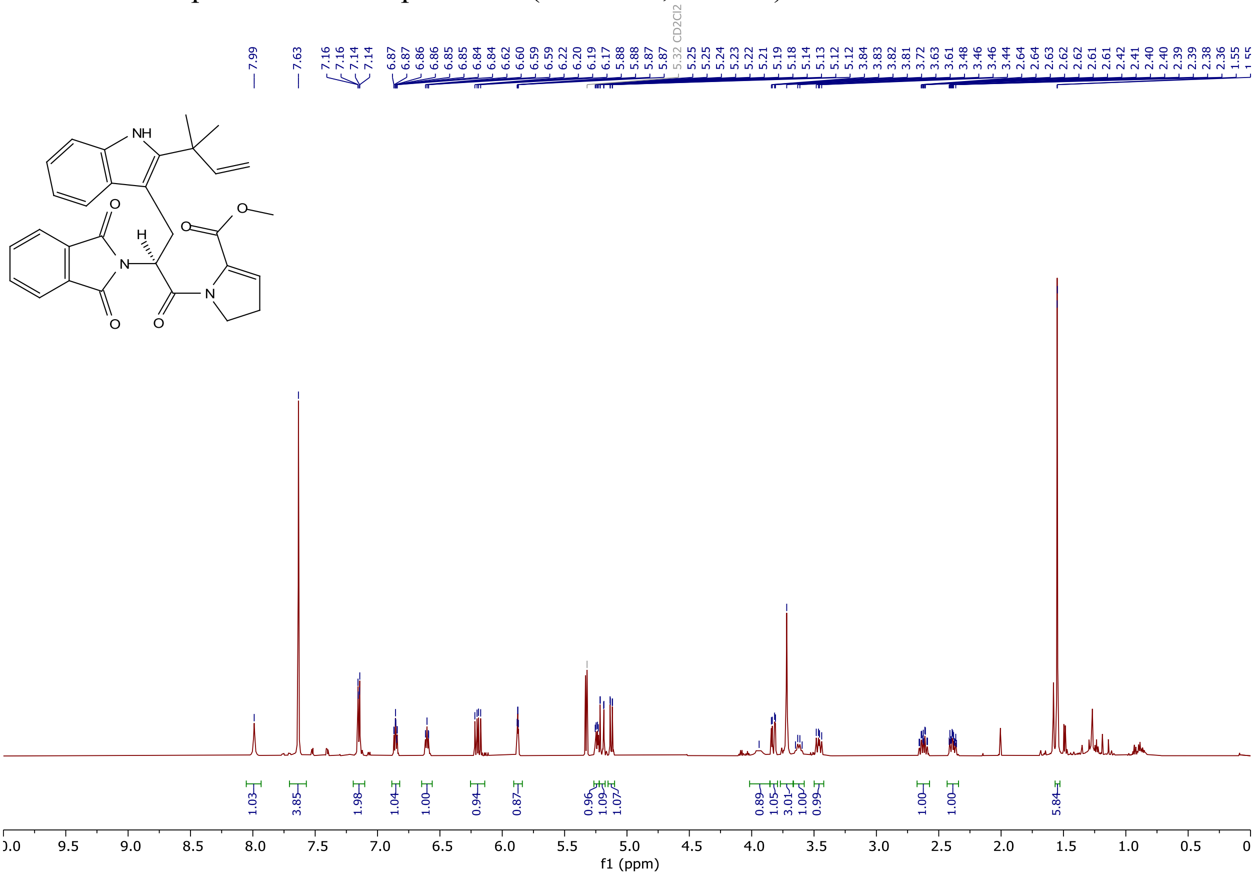

3.10 ^{13}C NMR Spectrum of Compound **21** (151 MHz, CD_2Cl_2)

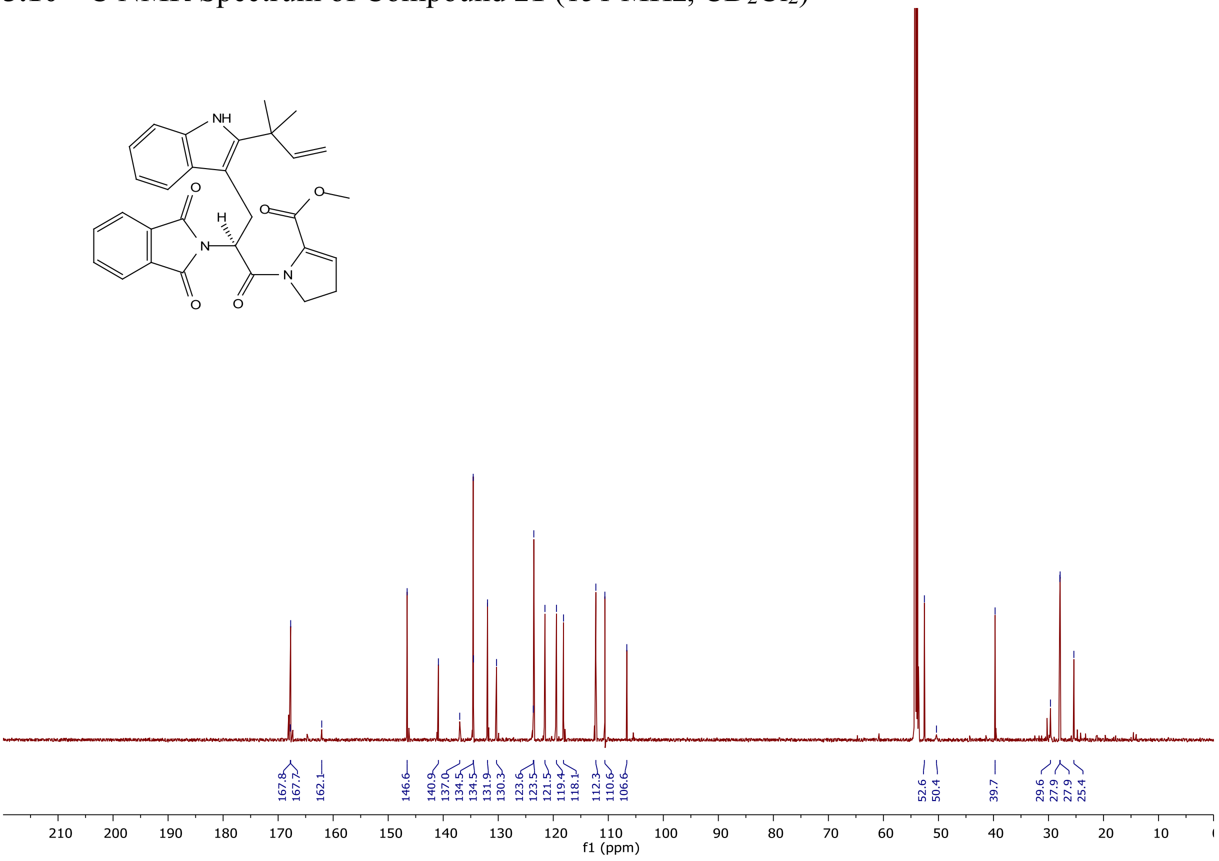

3.11 ^{1}H NMR Spectrum of (+)-dehydrodeoxy-brevianamide E (**9**) (500 MHz, $CDCl_3$)

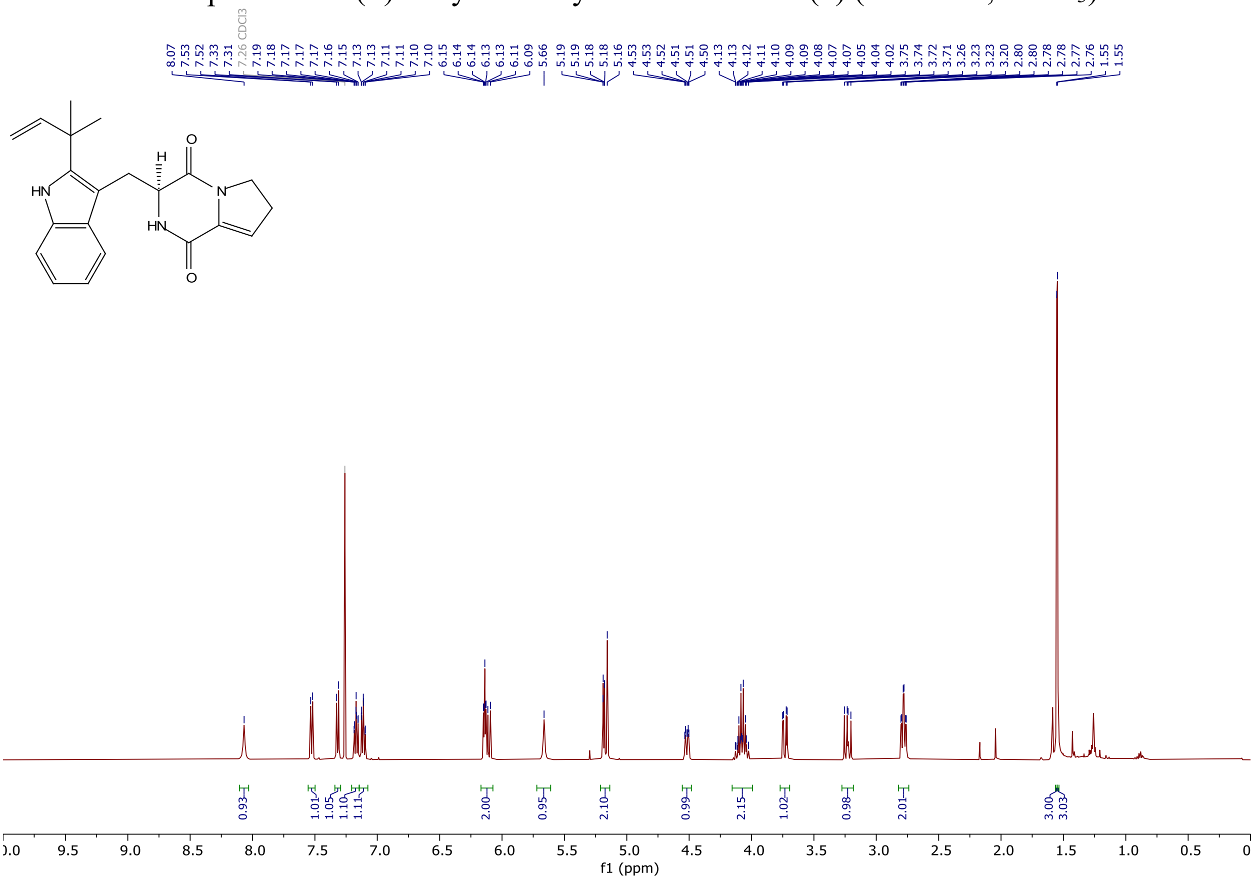

3.12 ^{13}C NMR Spectrum of (+)-dehydrodeoxy-brevianamide E (**9**) (126 MHz, $CDCl_3$)

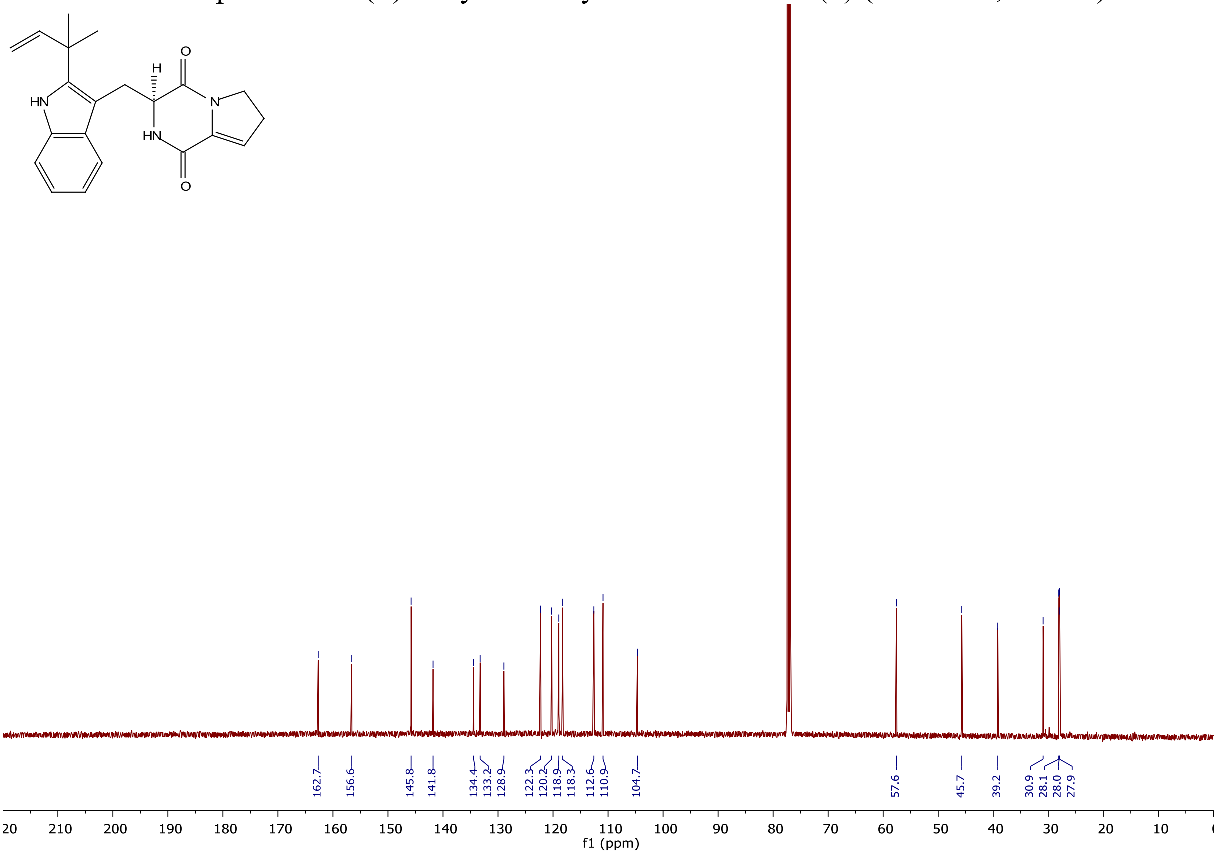

3.13 ^{1}H NMR Spectrum of (+)-dehydro-brevianamide E (**10**) (500 MHz, $CDCl_3$)

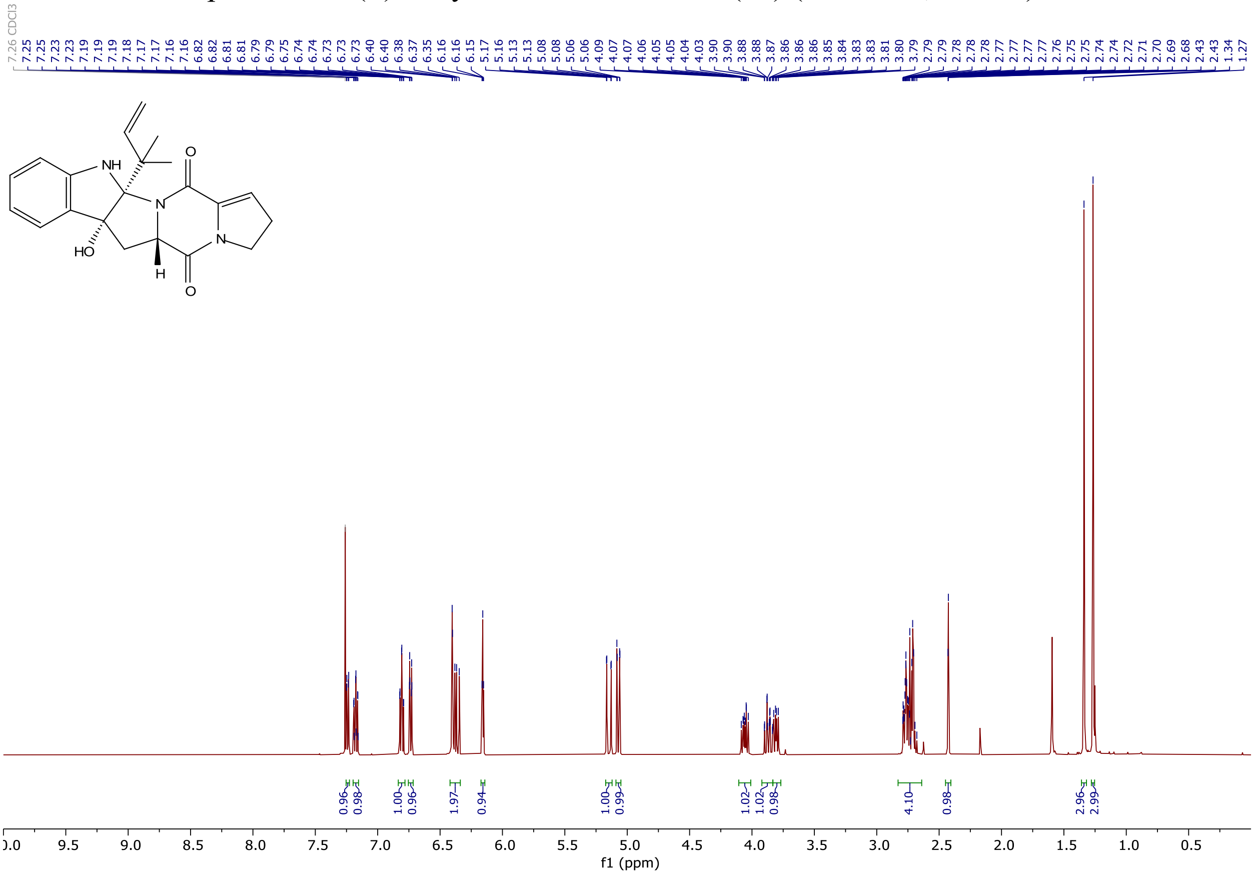

3.14 ^{13}C NMR Spectrum of (+)-dehydro-brevianamide E (**10**) (126 MHz, $CDCl_3$)

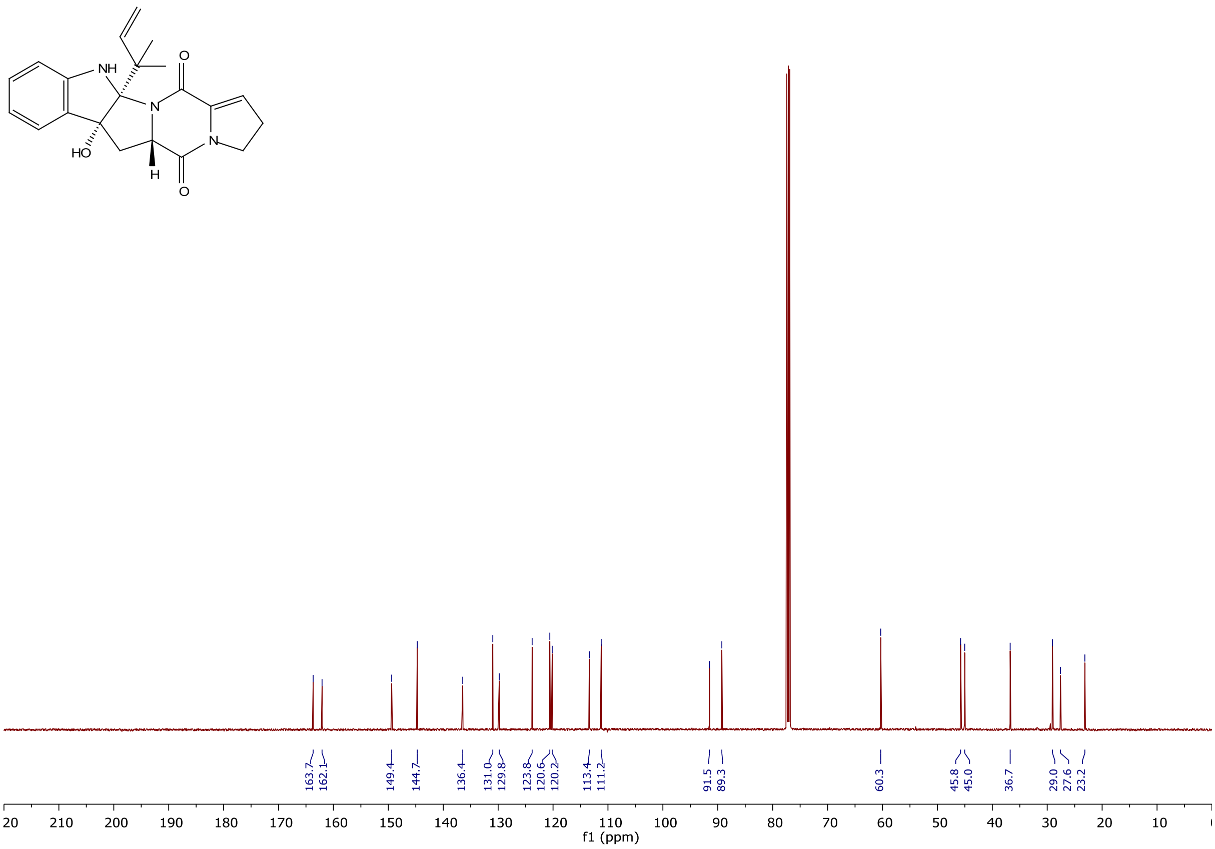

3.15 ^{1}H NMR Spectrum of Compound **22** (500 MHz, $CDCl_3$)

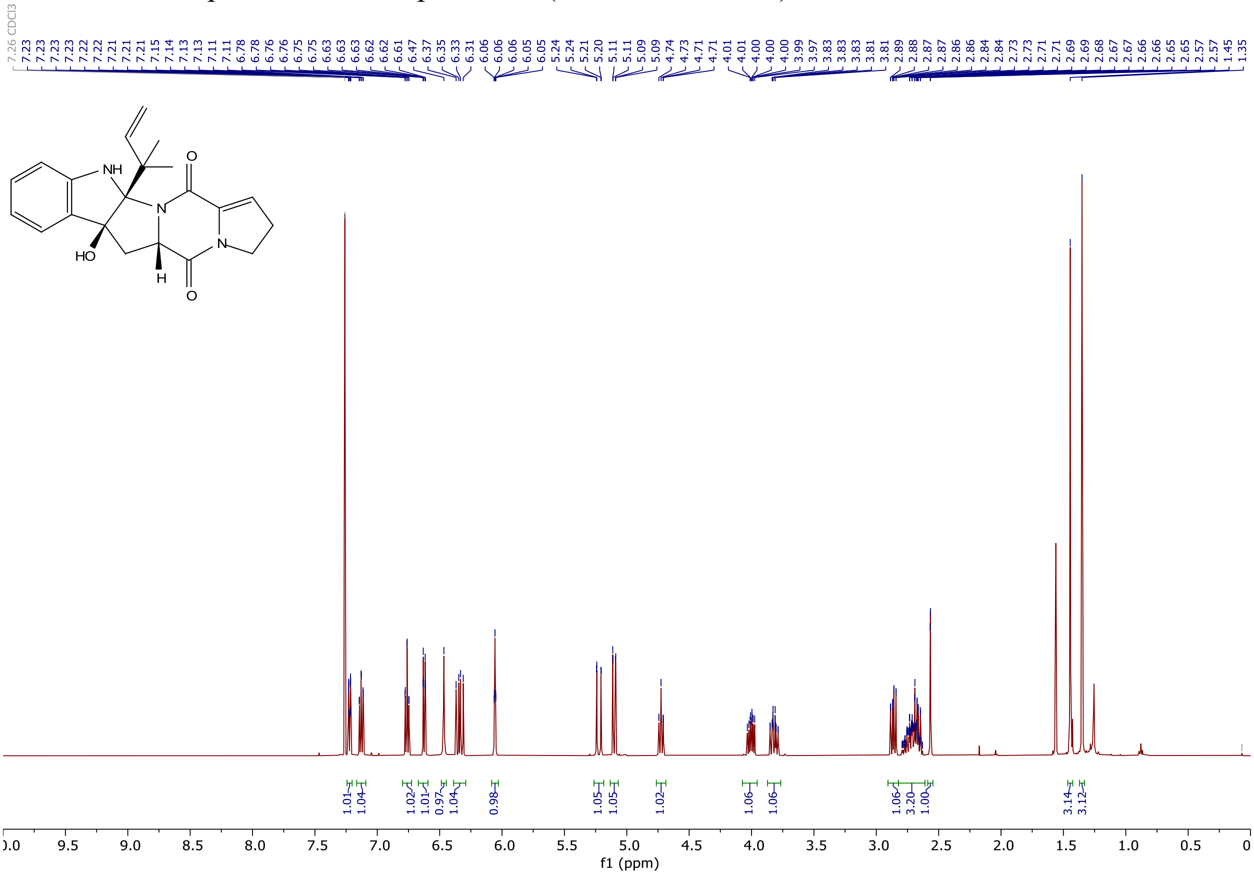

3.16 ^{13}C NMR Spectrum of Compound **22** (126 MHz, $CDCl_3$)

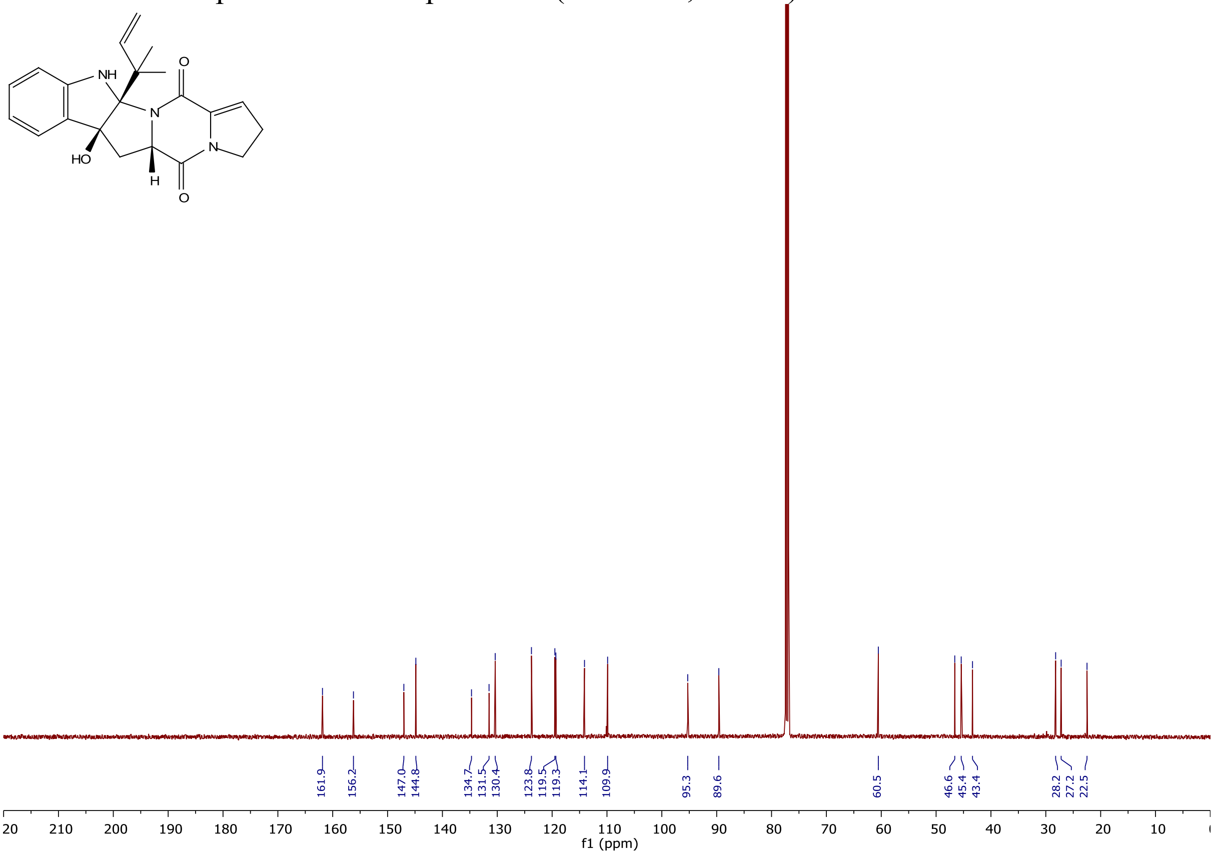

3.17 ^{1}H NMR Spectrum of Compound **22** (500 MHz, $(CD_3)_2SO$)

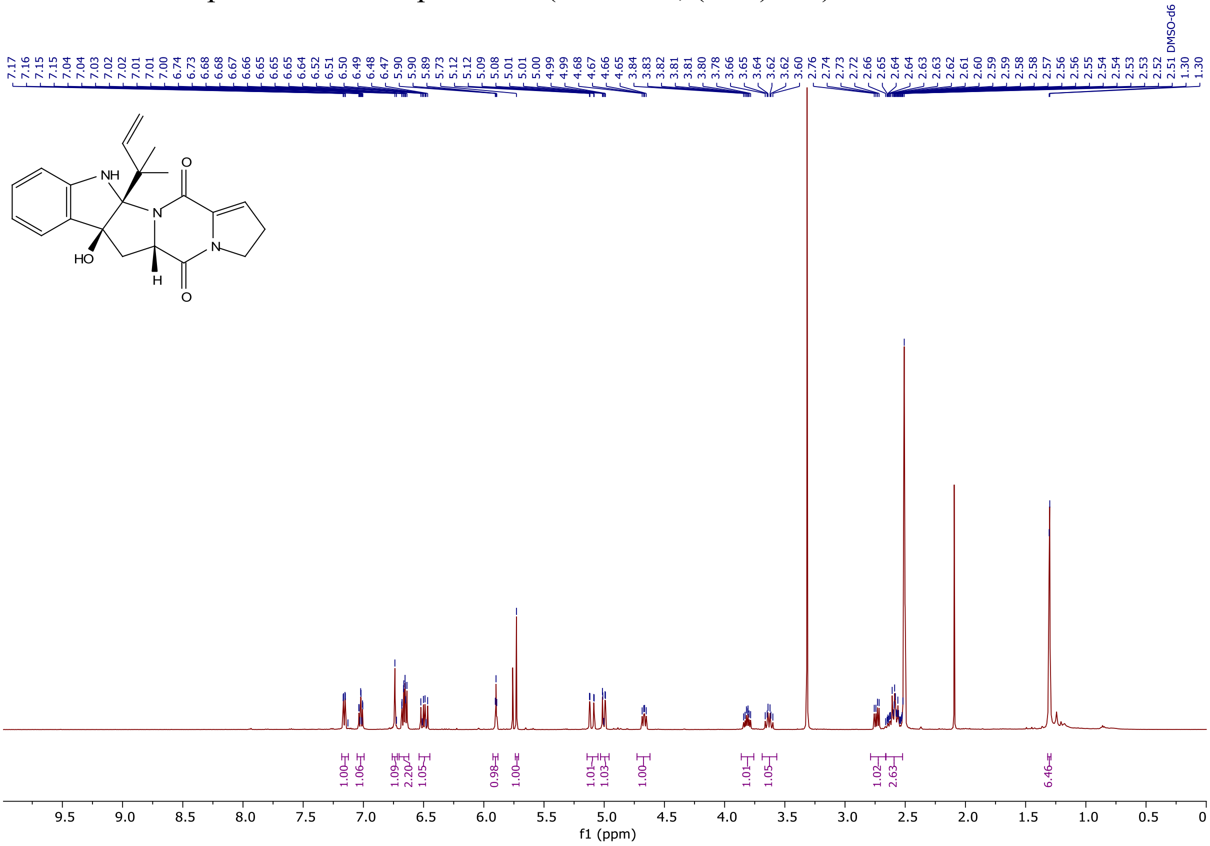

3.18 ^{13}C NMR Spectrum of Compound **22** (126 MHz, $(CD_3)_2SO$)

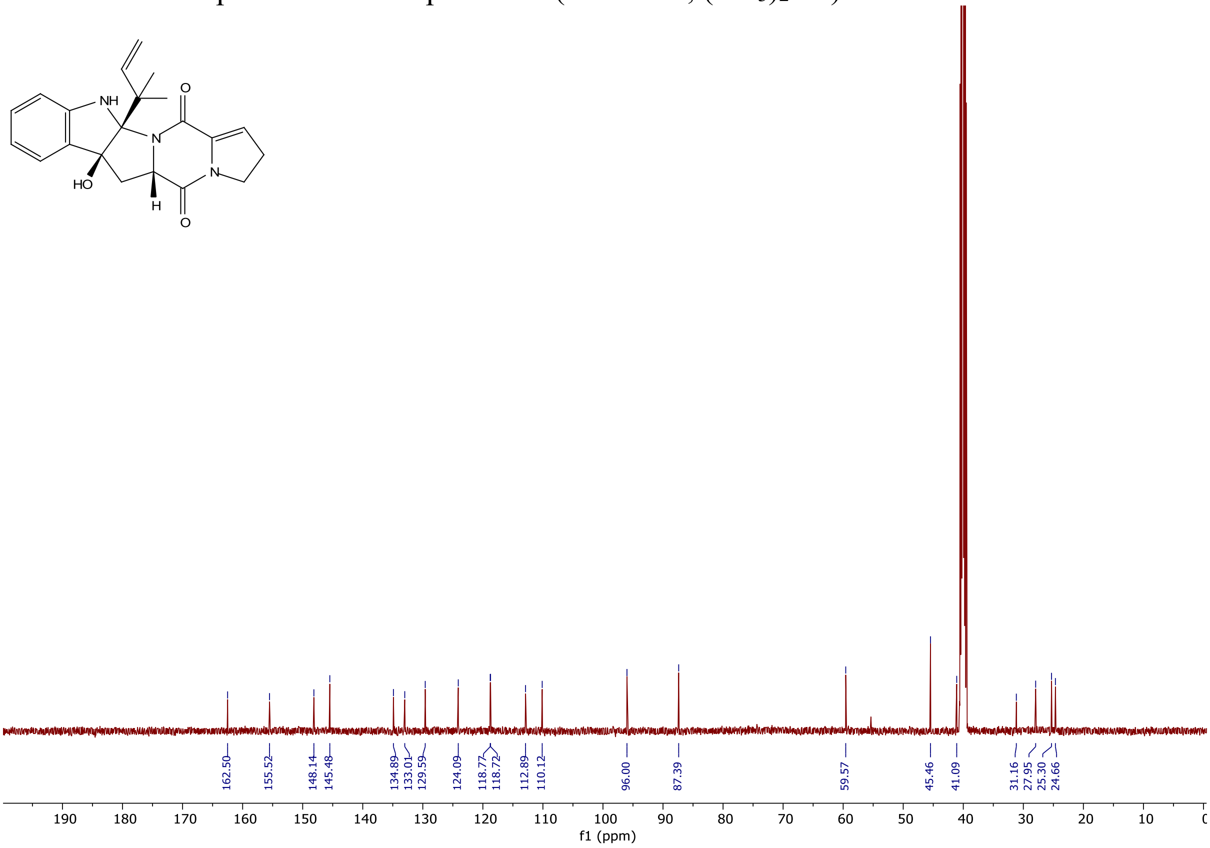

3.19 ^{1}H NMR Spectrum of dehydro-depyranoamoenamide A (601 MHz, $CDCl_3$)

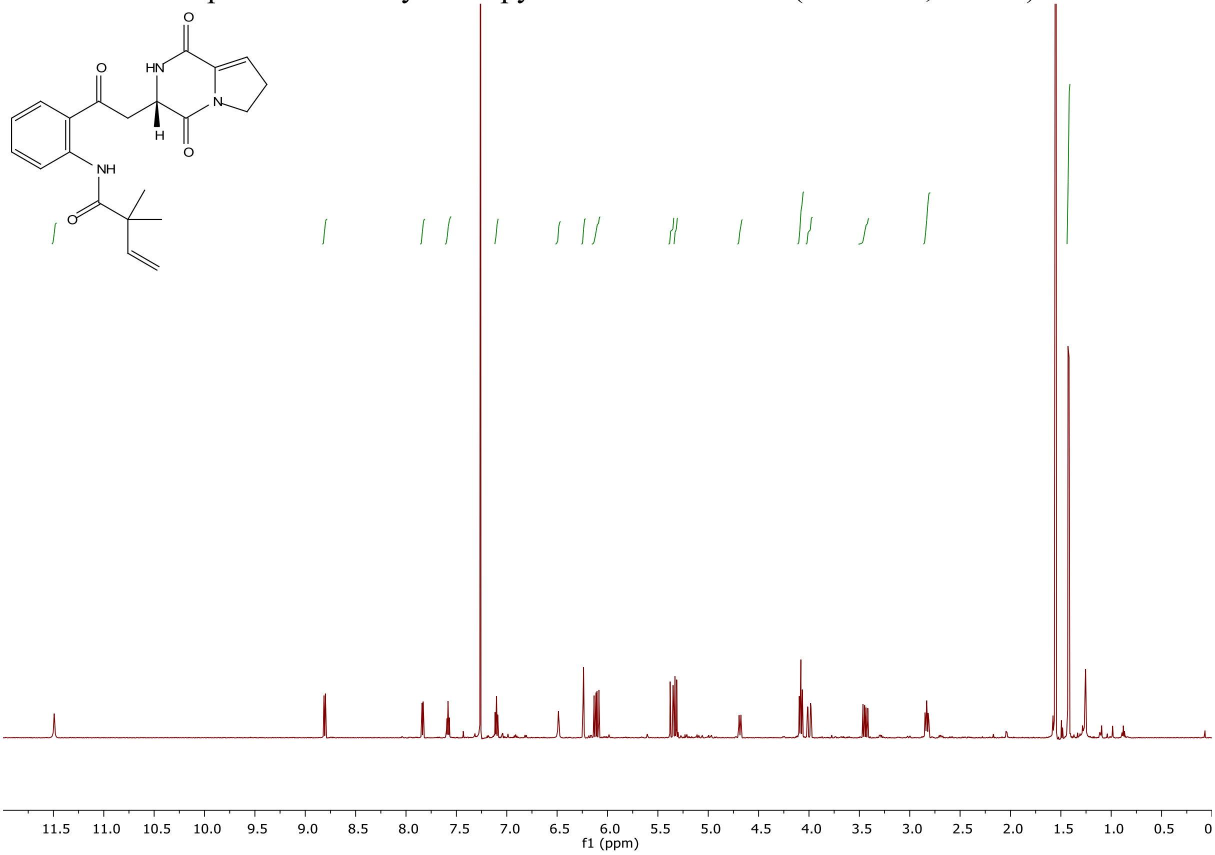

3.20 ^{13}C NMR Spectrum of dehydro-depyranoamoenamide A (151 MHz, $CDCl_3$)

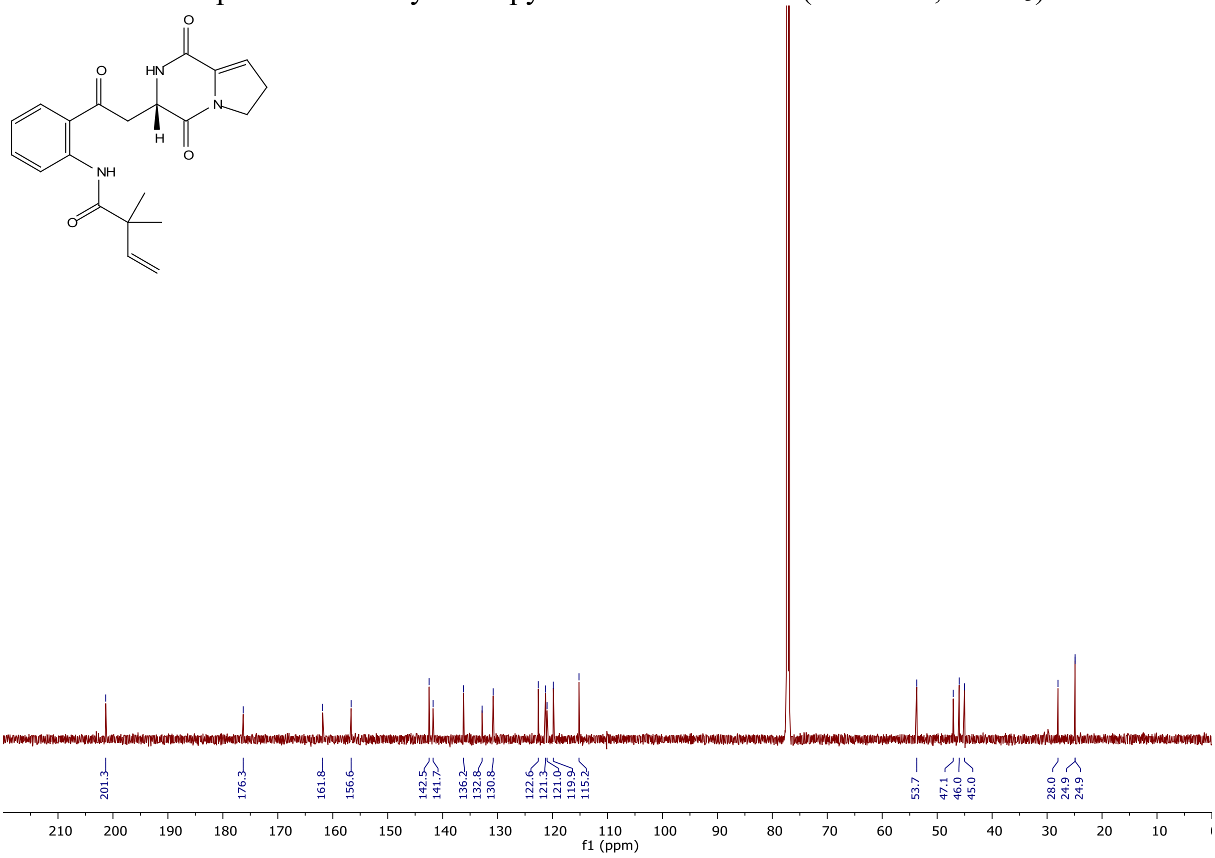

3.21 ¹H NMR Spectrum of Brevianamide A (**1**) (500 MHz, $CDCl_3$, ~3.0 mg/mL)

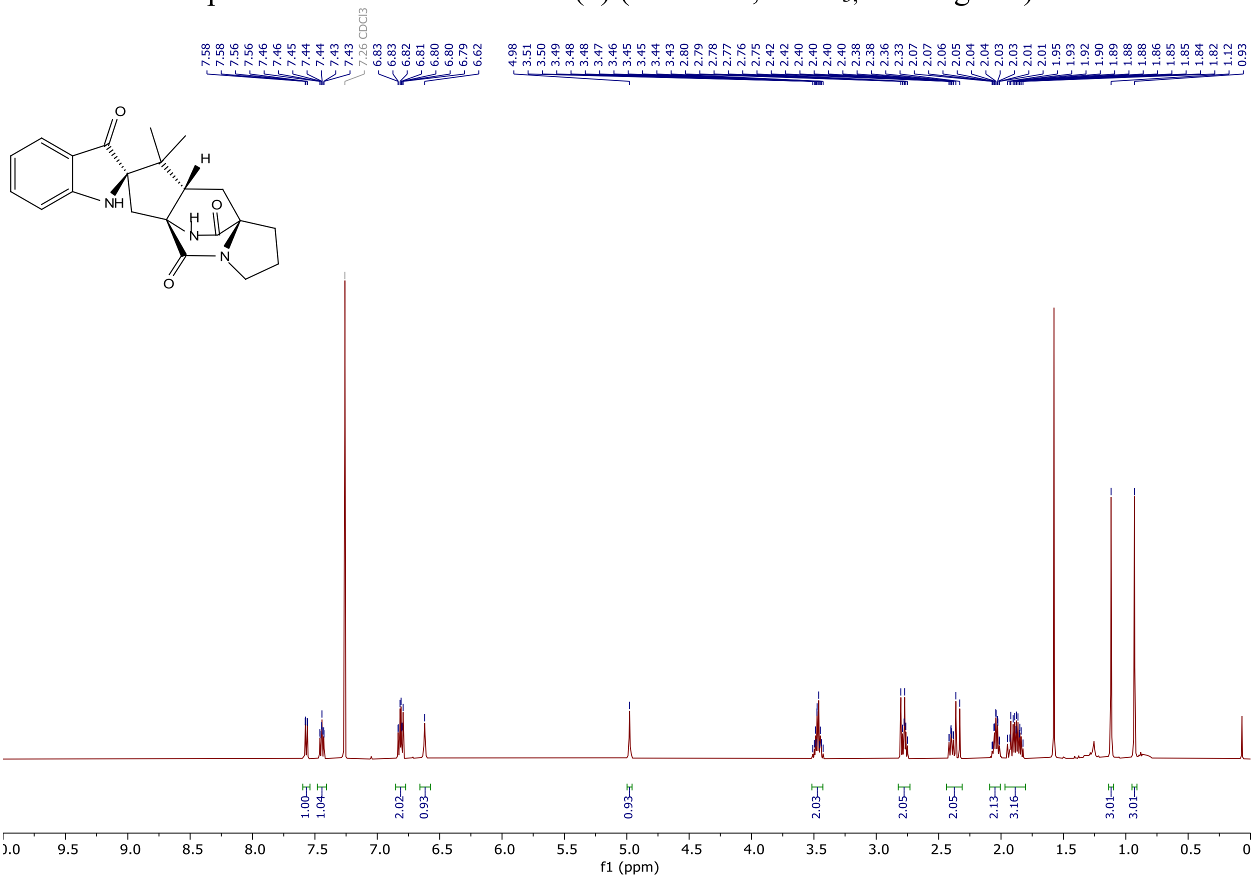

3.22 ¹³C NMR Spectrum of Brevianamide A (**1**) (126 MHz, $CDCl_3$, ~3.0 mg/mL)

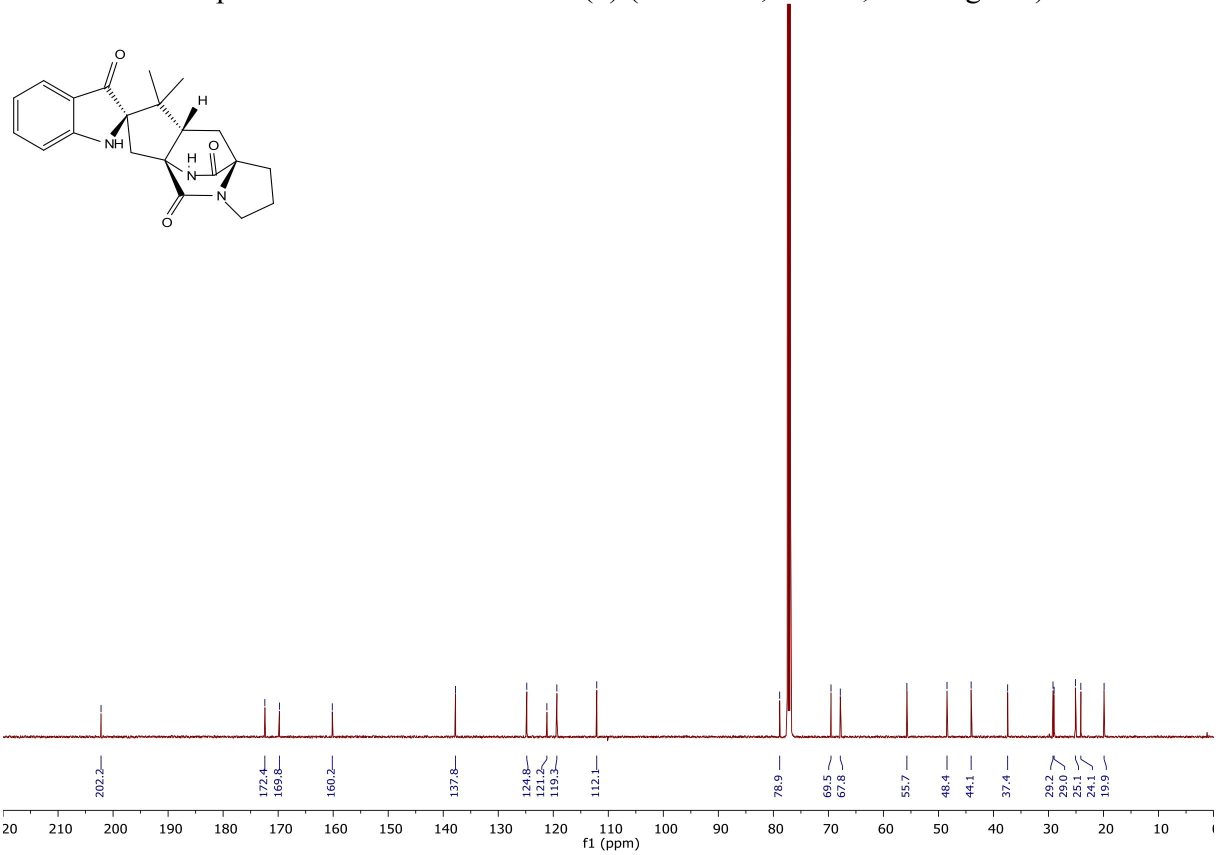

3.23 ^1H-^1H COSY Spectrum of Brevianamide A (**1**) ($CDCl_3$)

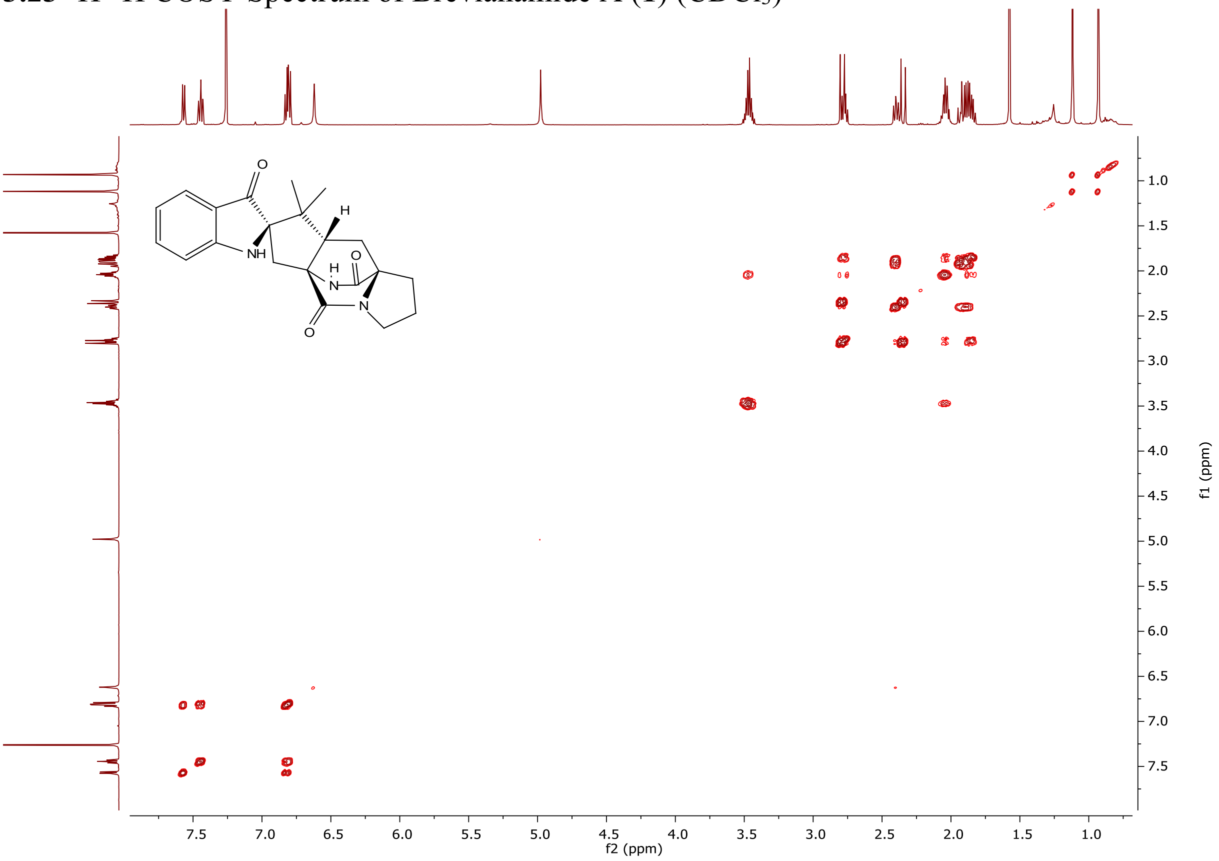

3.24 ^1H-^{13}C HSQC Spectrum of Brevianamide A (**1**) ($CDCl_3$)

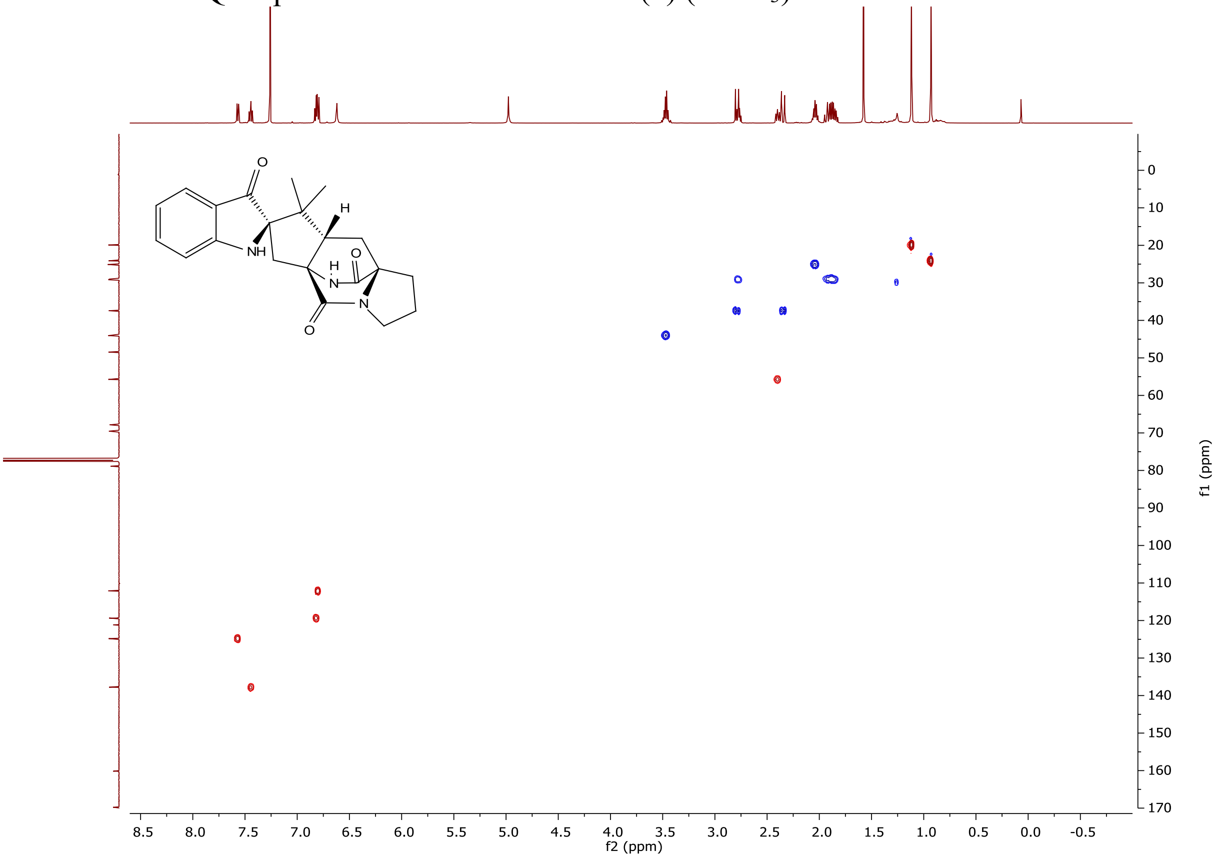

3.25 ^{1}H-^{13}C HMBC Spectrum of Brevianamide A (**1**) (CDCl$_3$)

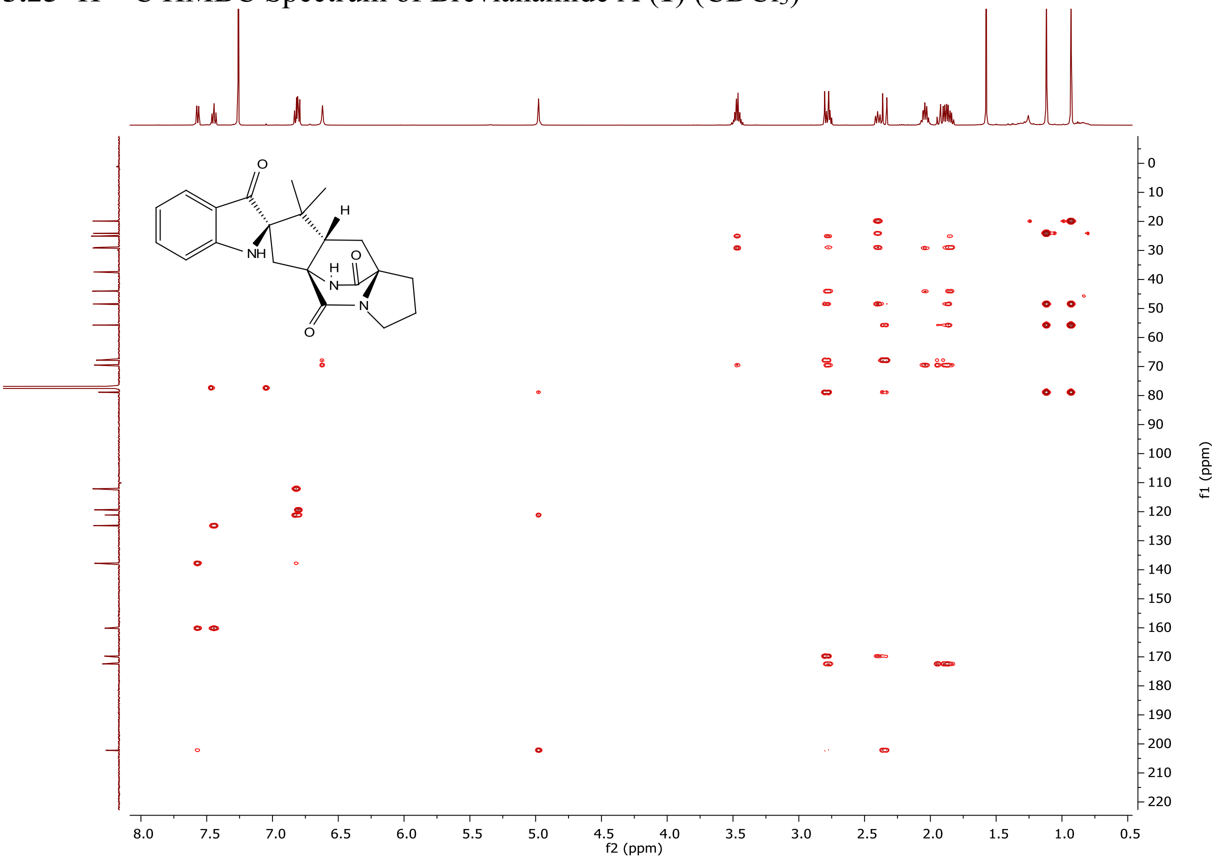

3.26 ^{1}H-^{1}H NOESY Spectrum of Brevianamide A (**1**) (CDCl$_3$)

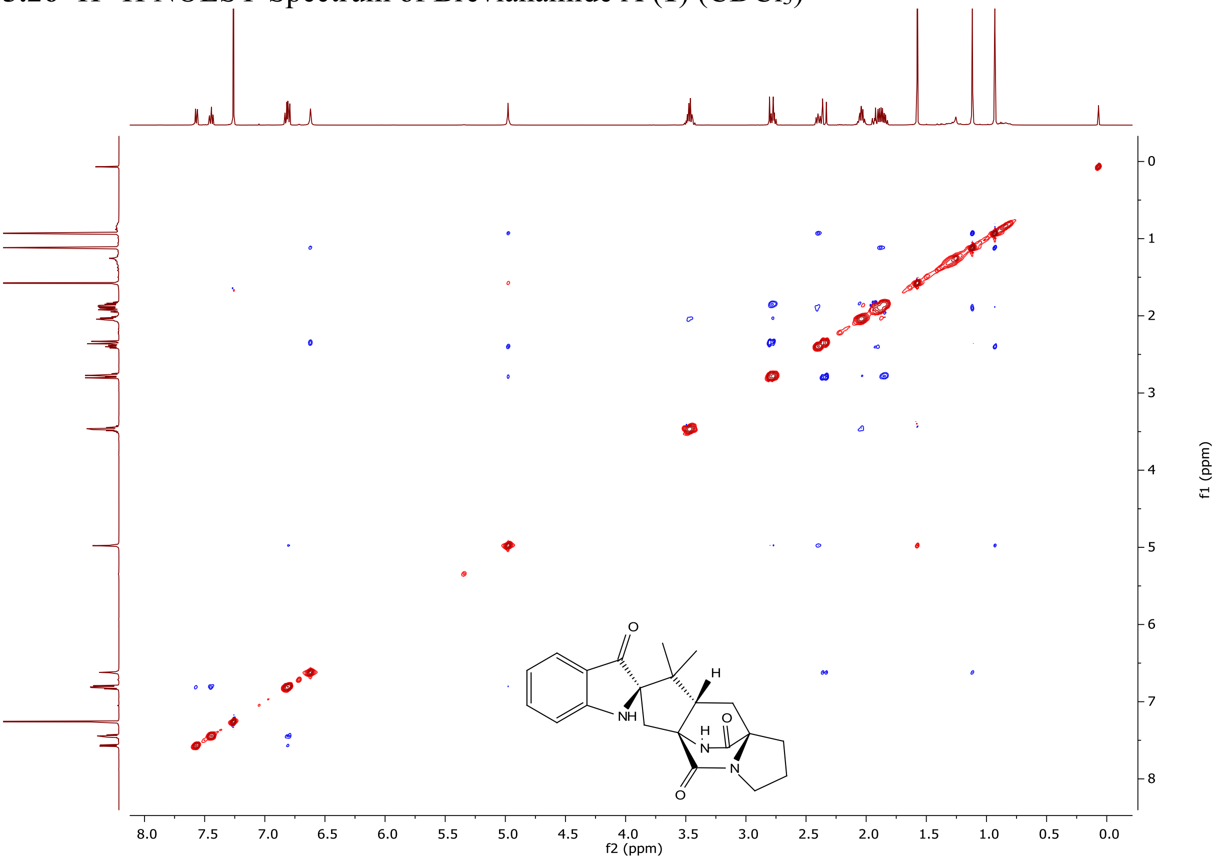

3.27 ^{1}H NMR Spectra of Brevianamide A (**1**) (500 MHz, $CDCl_3$ – conc. dependence)

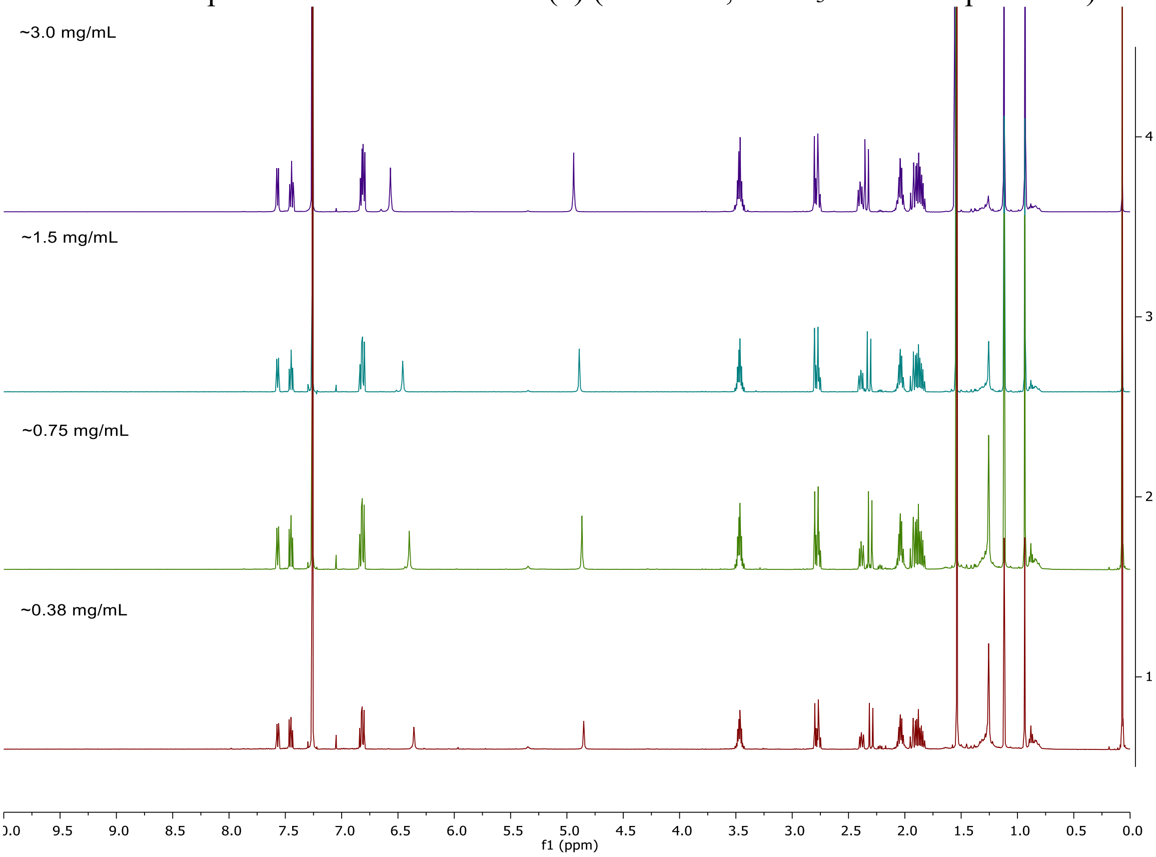

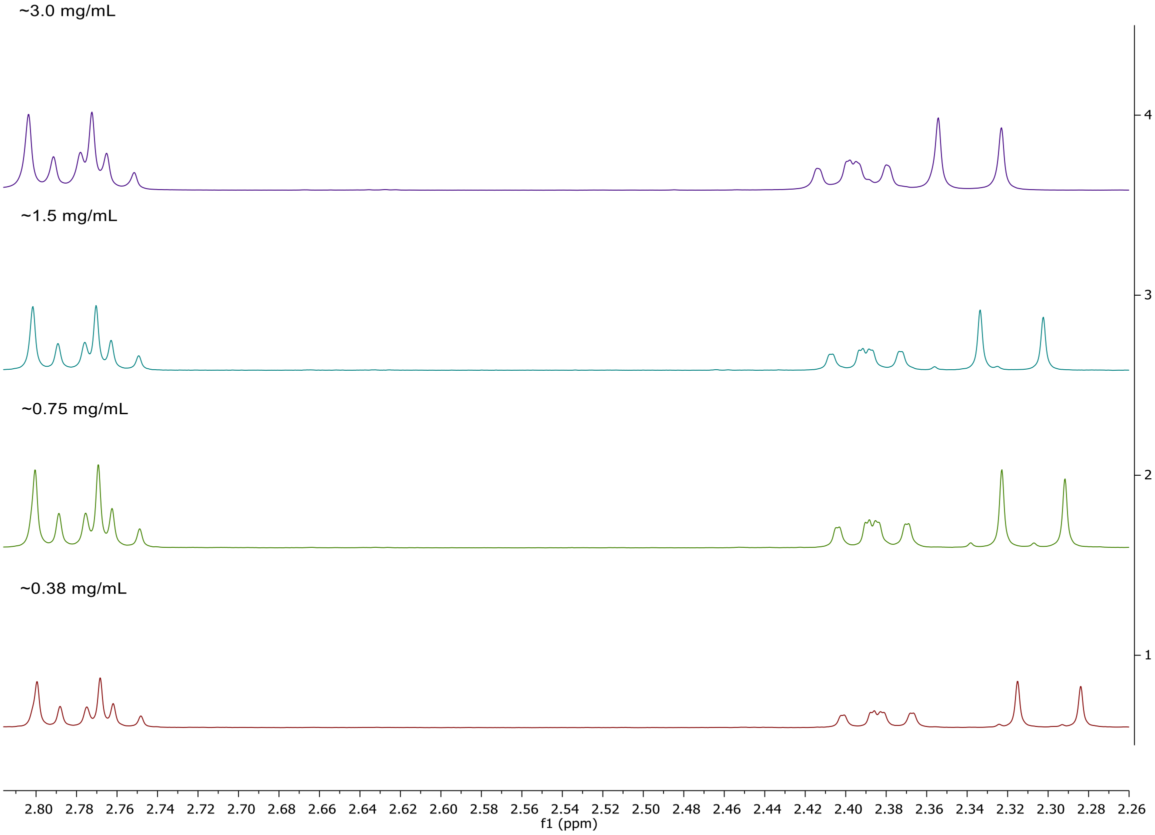

3.28 ^{1}H NMR Spectrum of Brevianamide A (**1**) (500 MHz, $(CD_3)_2SO$)

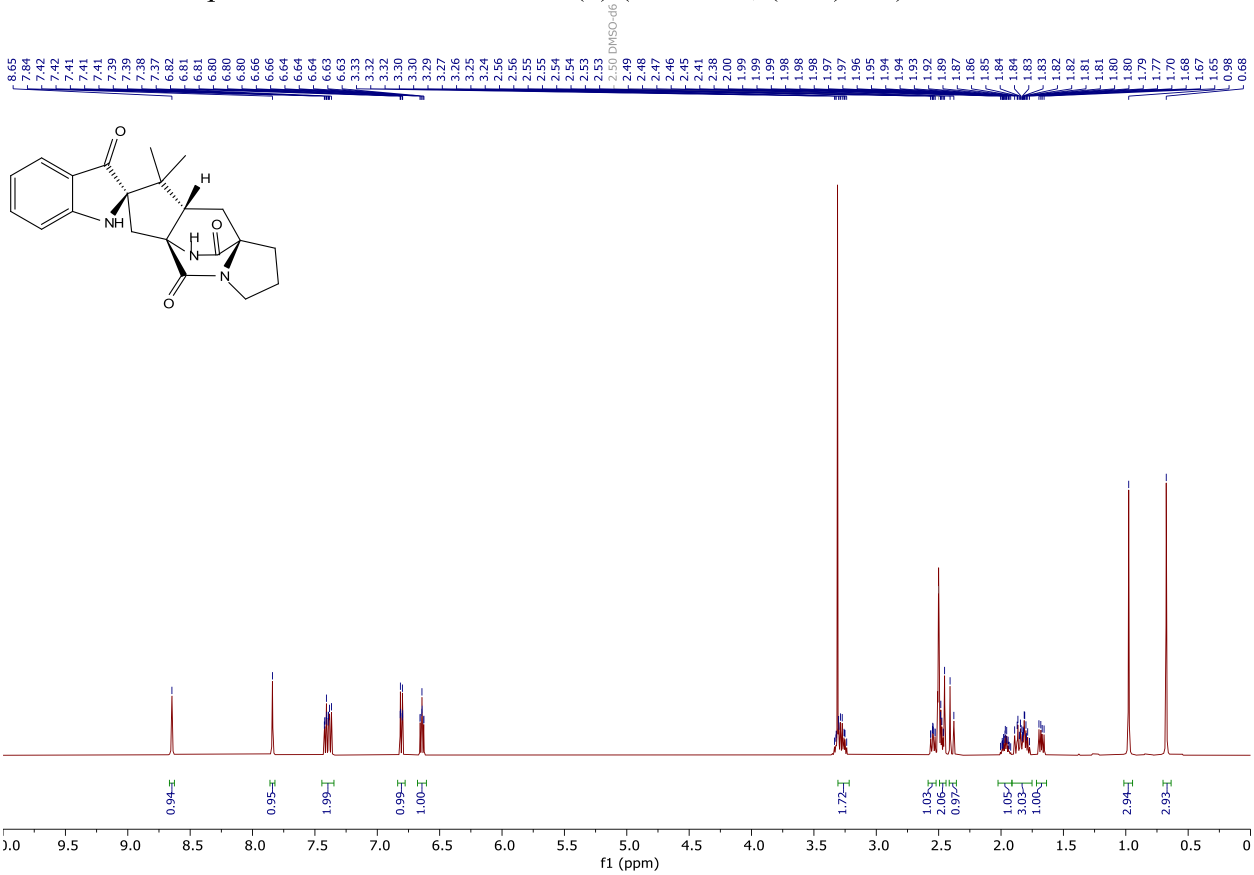

3.29 ^{13}C NMR Spectrum of Brevianamide A (**1**) (126 MHz, $(CD_3)_2SO$)

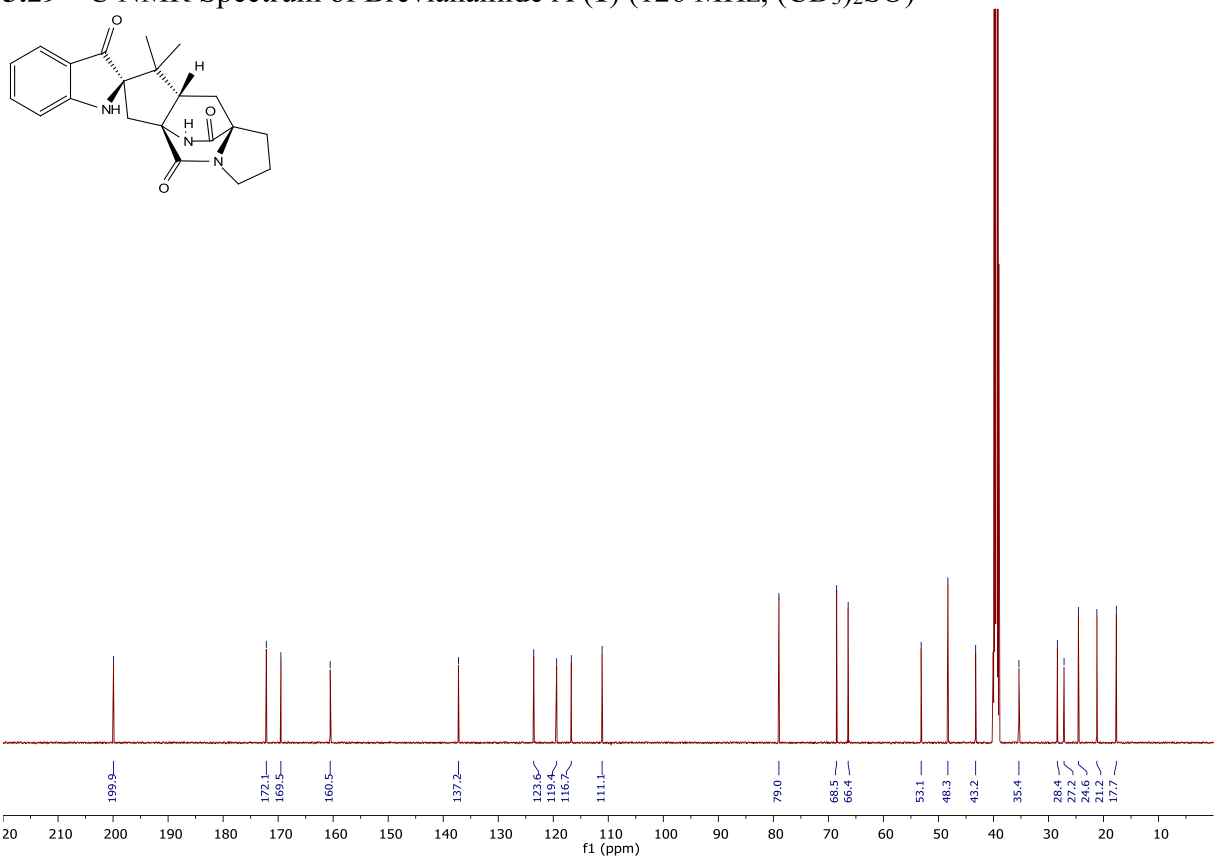

3.30 ¹H-¹H COSY Spectrum of Brevianamide A (**1**) (($(CD_3)_2SO$))

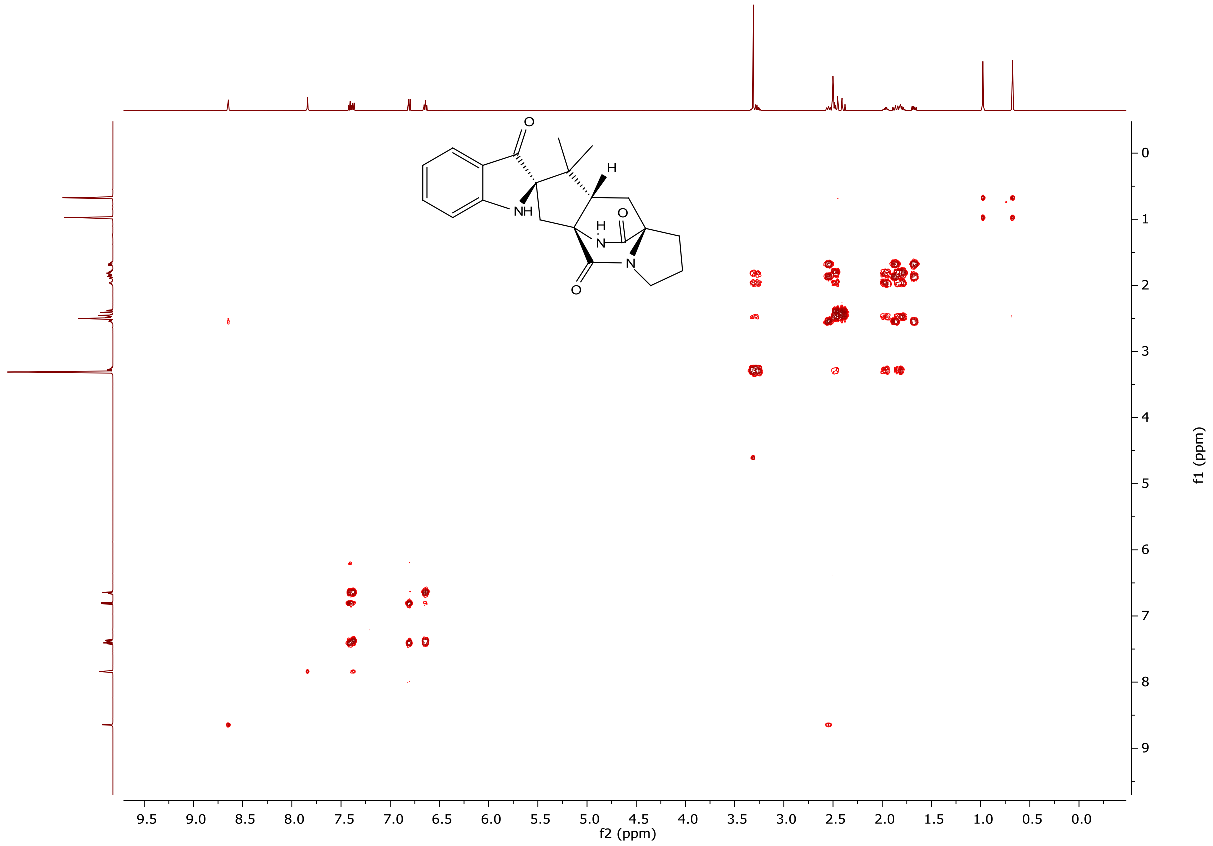

3.31 ¹H-¹³C HSQC Spectrum of Brevianamide A (**1**) (($(CD_3)_2SO$))

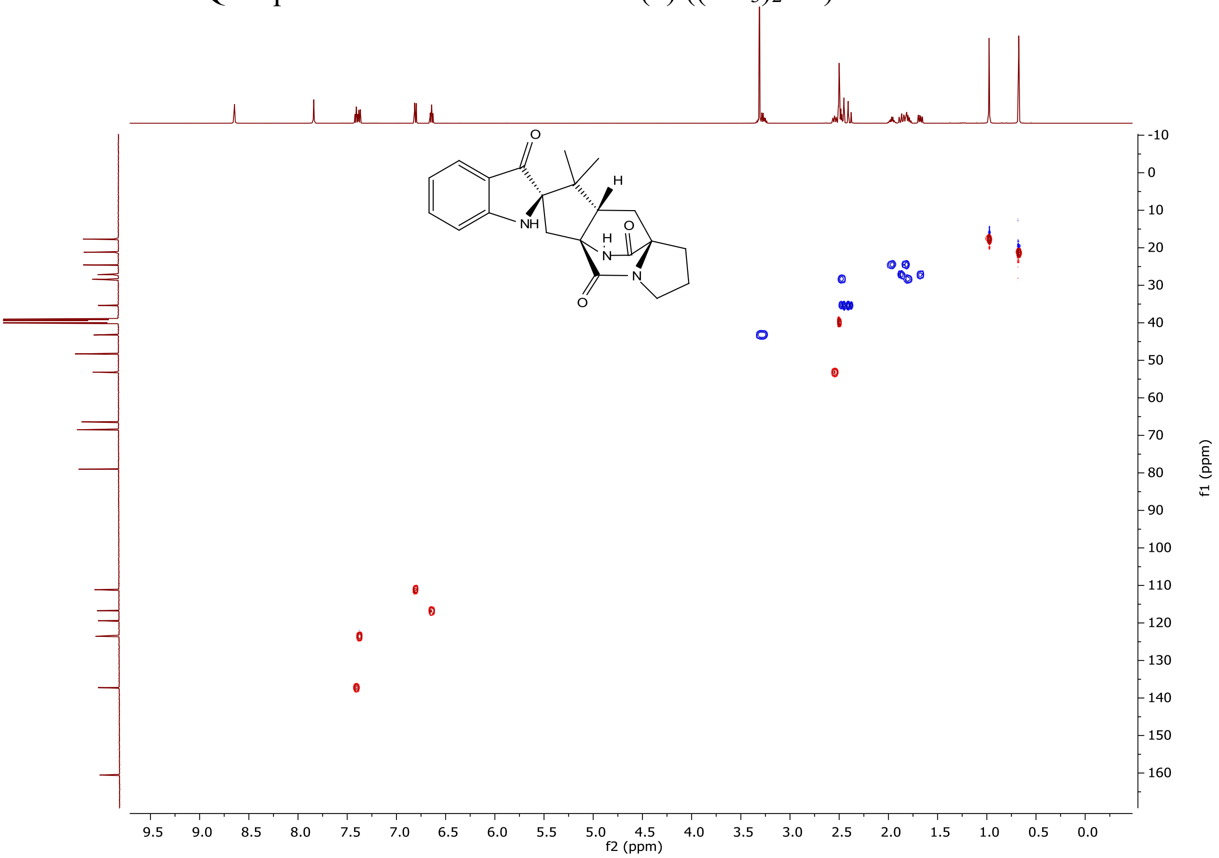

3.32 ^{1}H-^{13}C HMBC Spectrum of Brevianamide A (**1**) ((CD_3)$_2$SO)

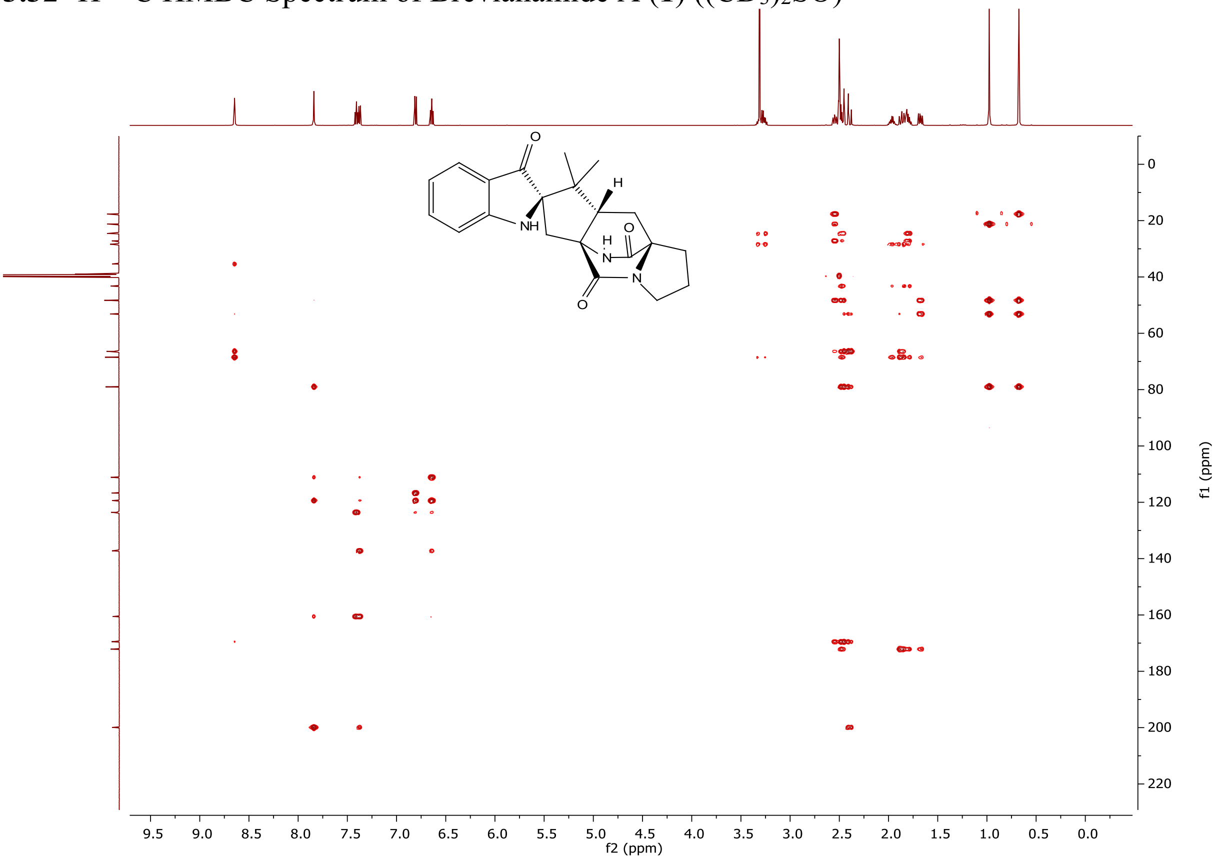

3.33 ^{1}H-^{1}H NOESY Spectrum of Brevianamide A (**1**) ((CD_3)$_2$SO)

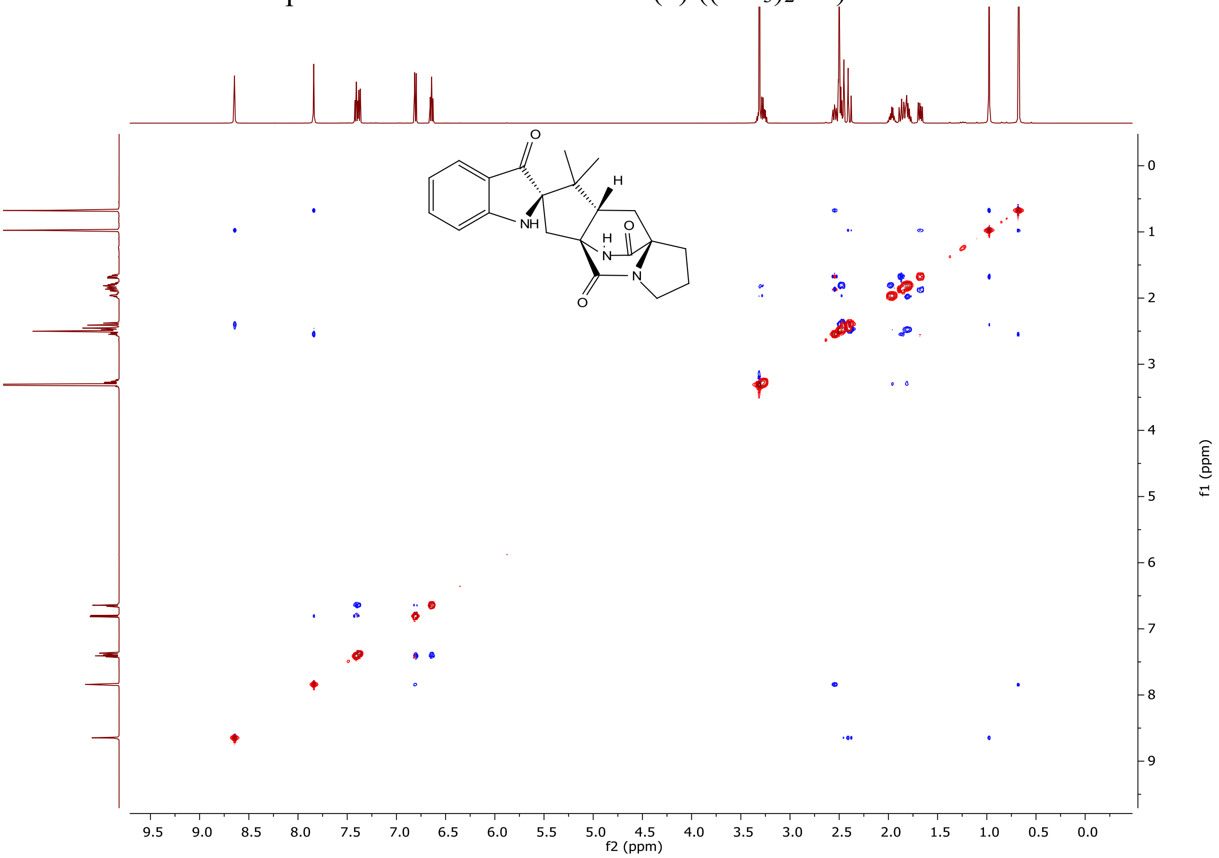

3.34 ^{1}H NMR Spectrum of Brevianamide B (2) (500 MHz, $CDCl_3$)

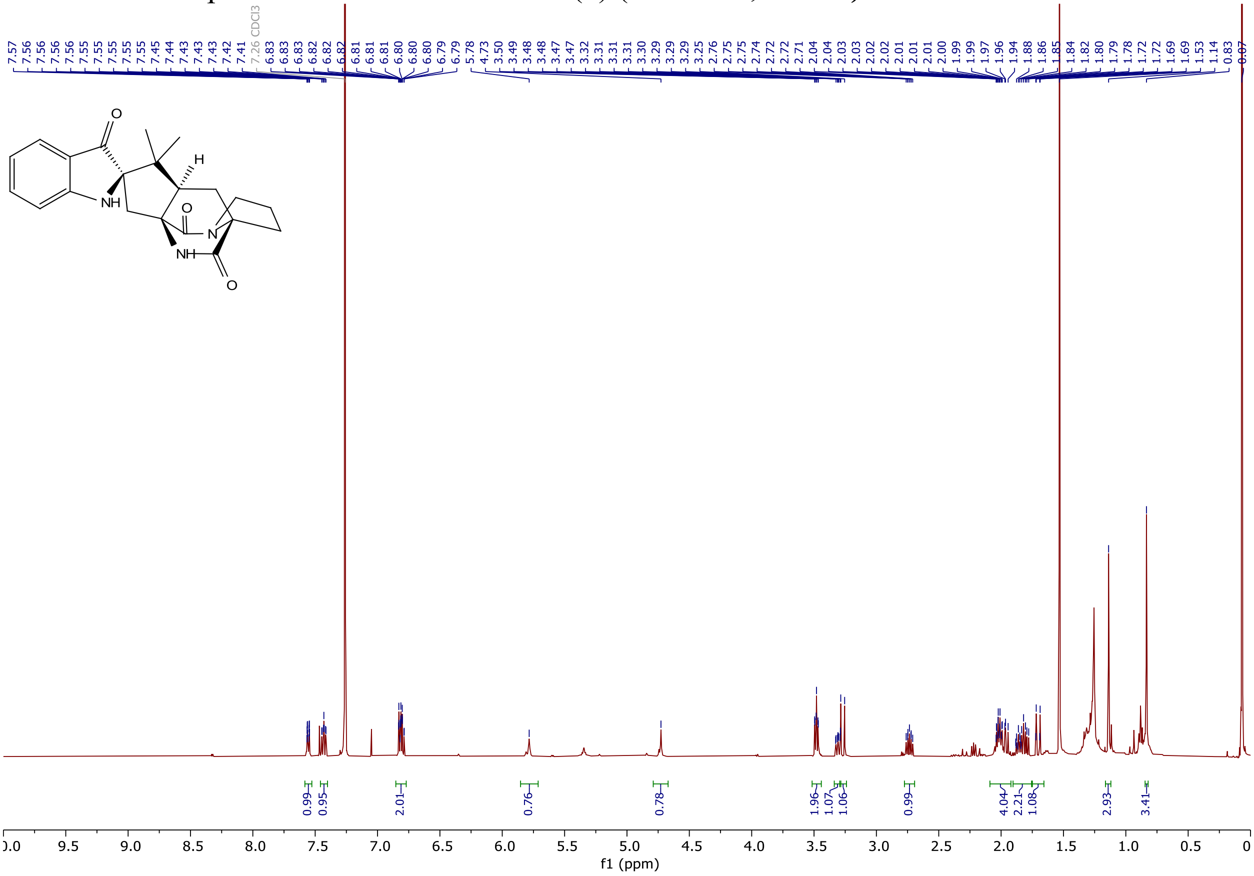

3.35 ^{13}C NMR Spectrum of Brevianamide B (2) (126 MHz, $CDCl_3$)

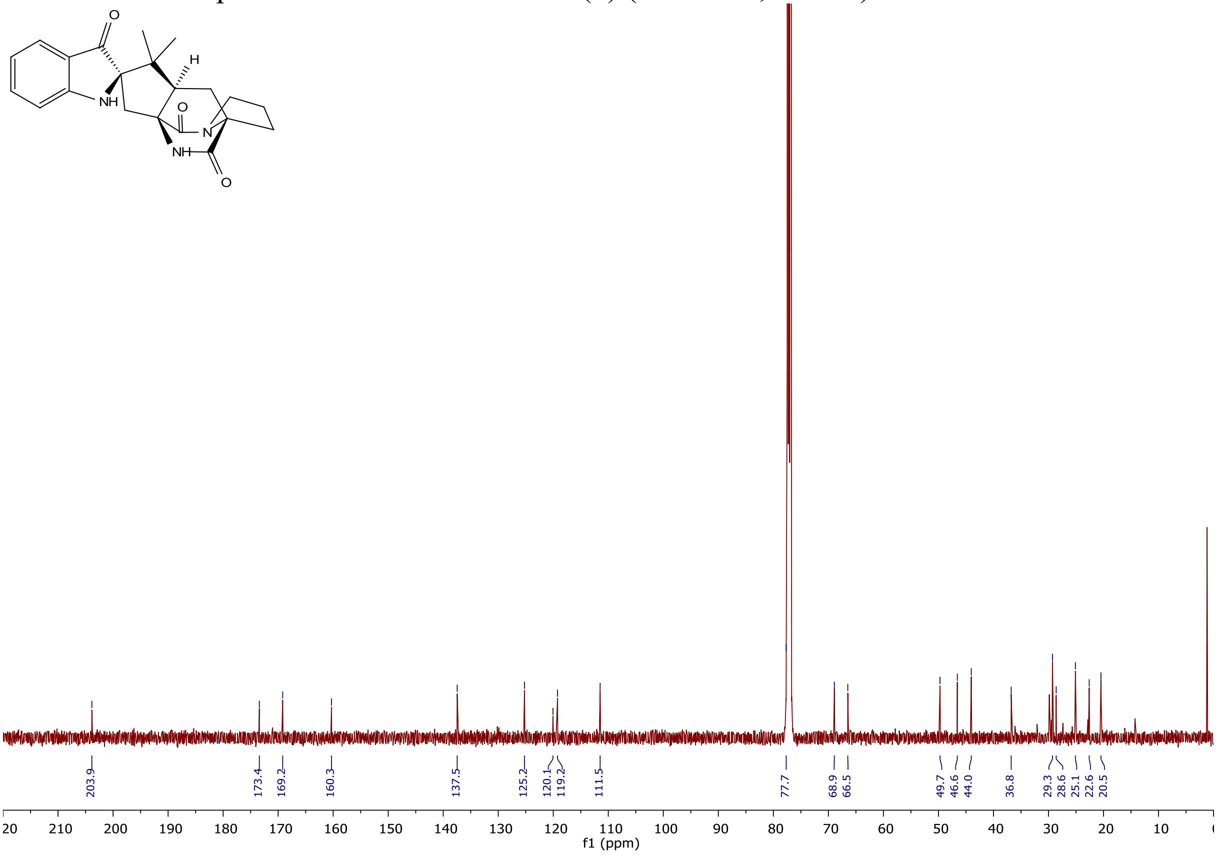

3.36 ^{1}H NMR Spectrum of Brevianamide B (**2**) (500 MHz, $(CD_3)_2SO$)

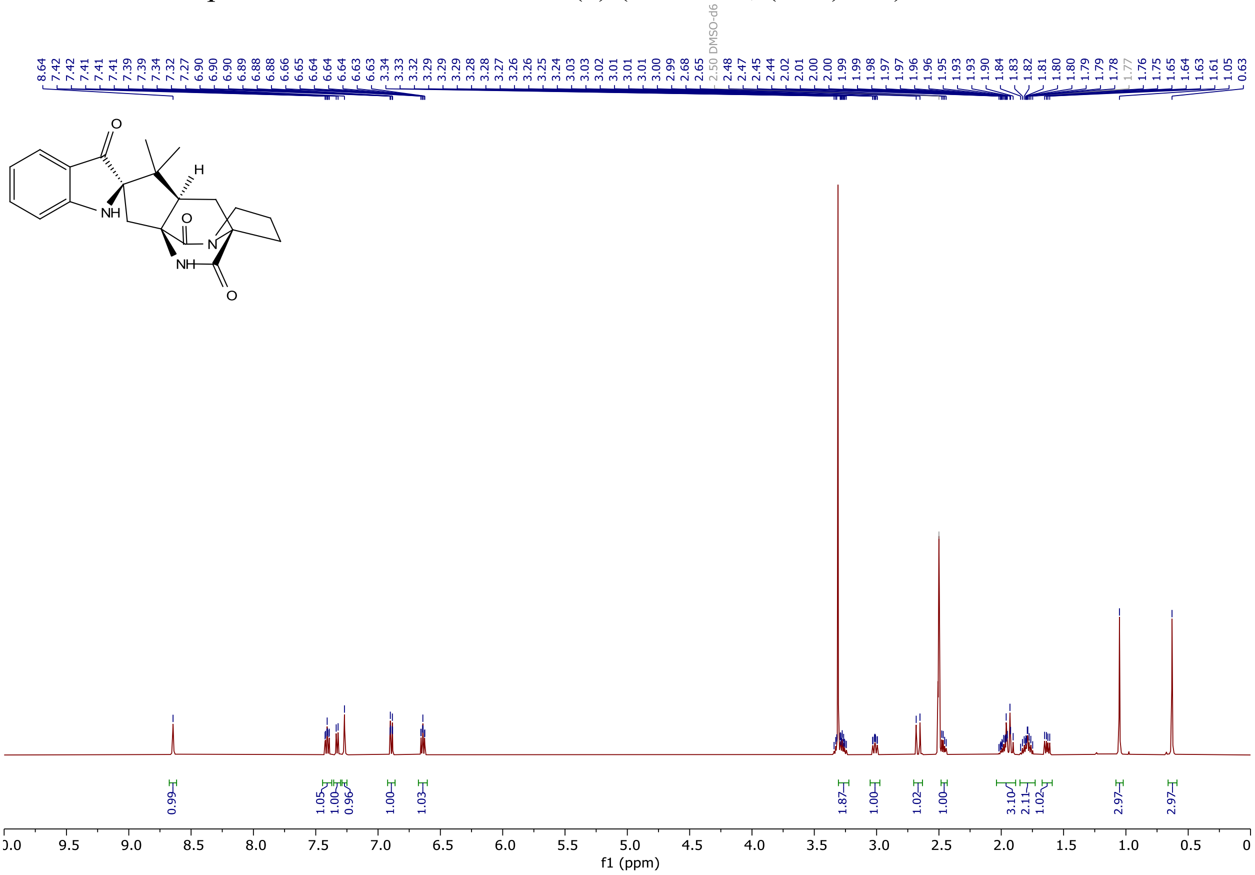

3.37 ^{13}C NMR Spectrum of Brevianamide B (**2**) (126 MHz, $(CD_3)_2SO$)

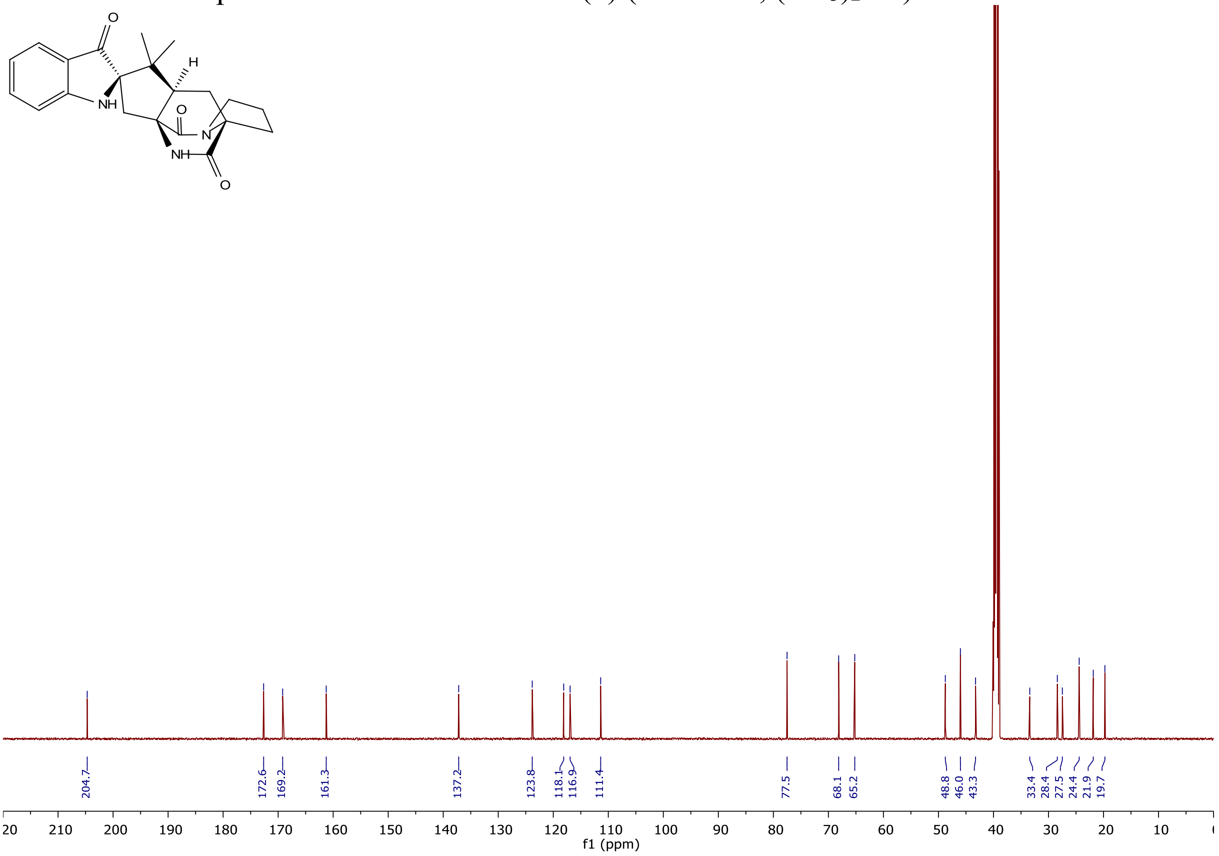

3.38 ^{1}H-^{1}H COSY Spectrum of Brevianamide B (**2**) ($(CD_3)_2SO$)

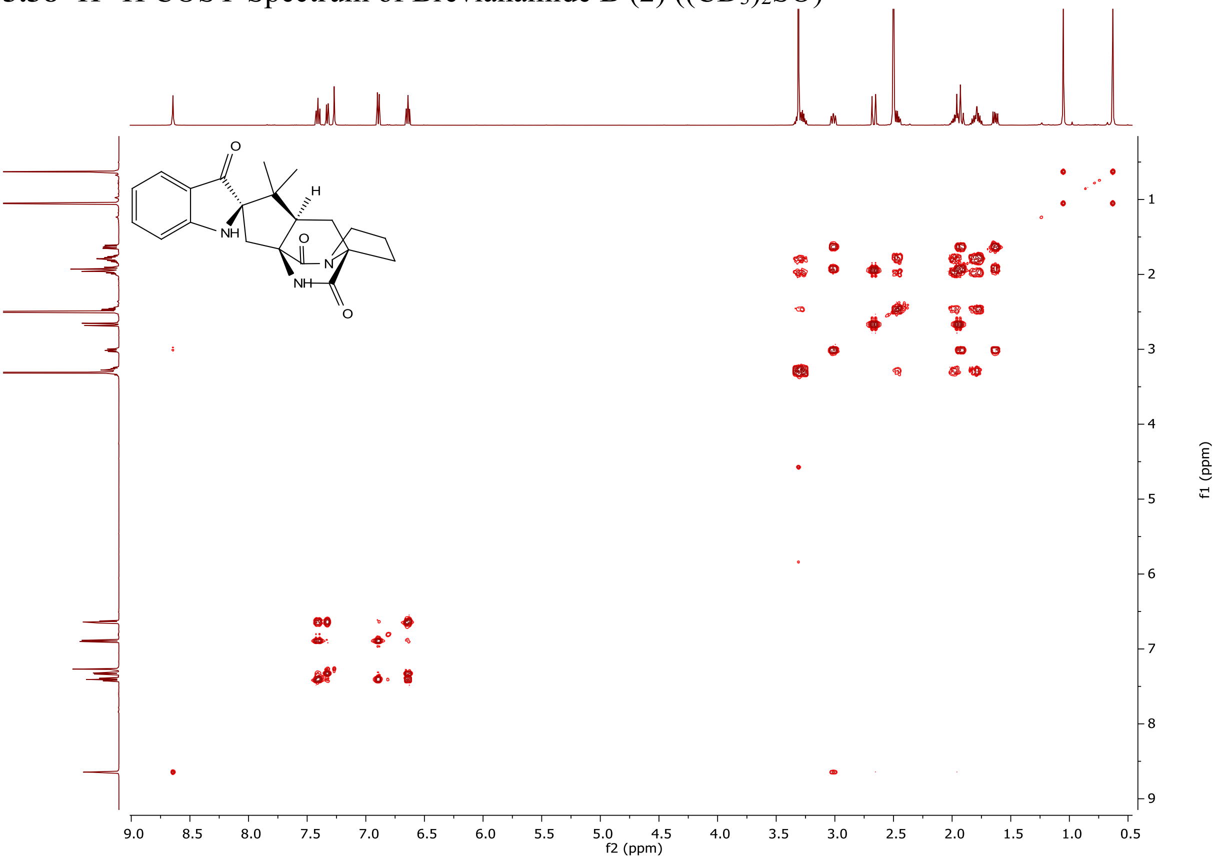

3.39 ^{1}H-^{13}C HSQC Spectrum of Brevianamide B (**2**) ($(CD_3)_2SO$)

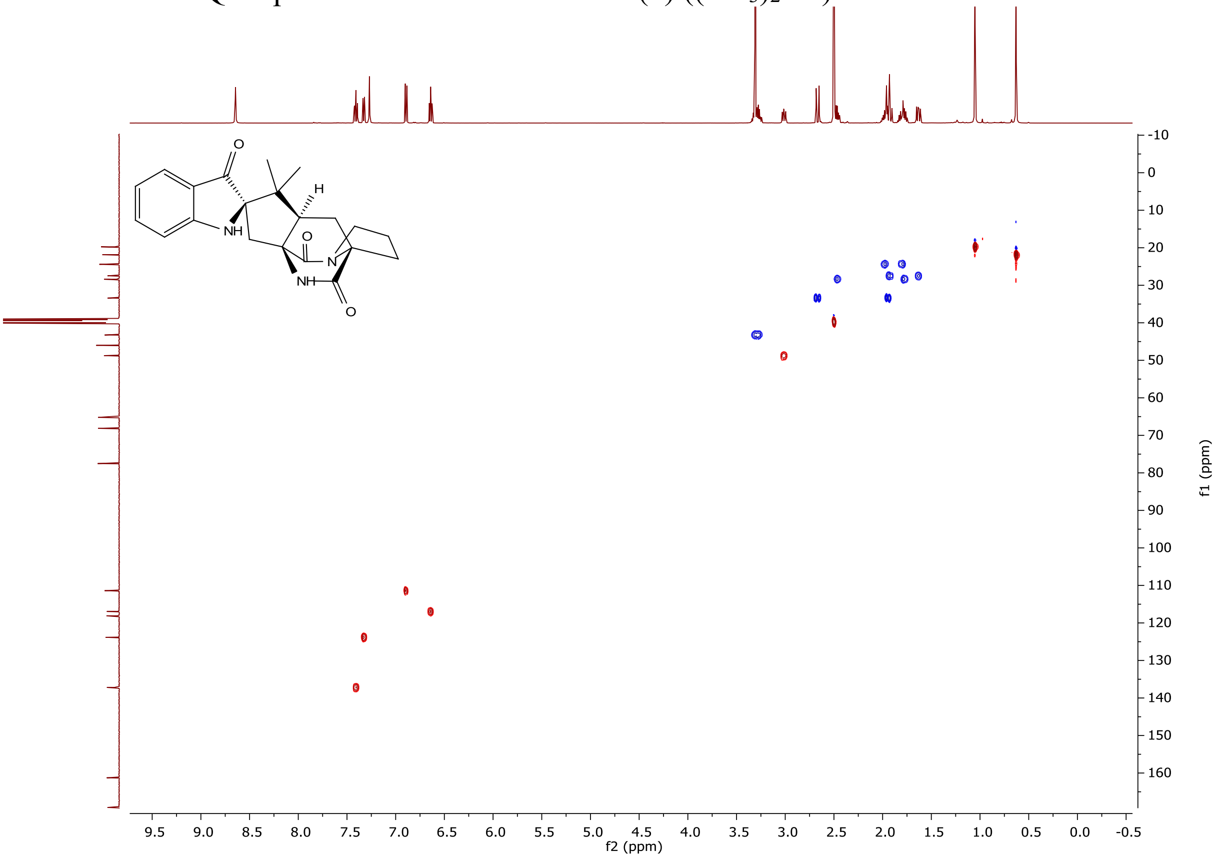

3.40 ^{1}H-^{13}C HMBC Spectrum of Brevianamide B (**2**) ((CD_3)$_2$SO)

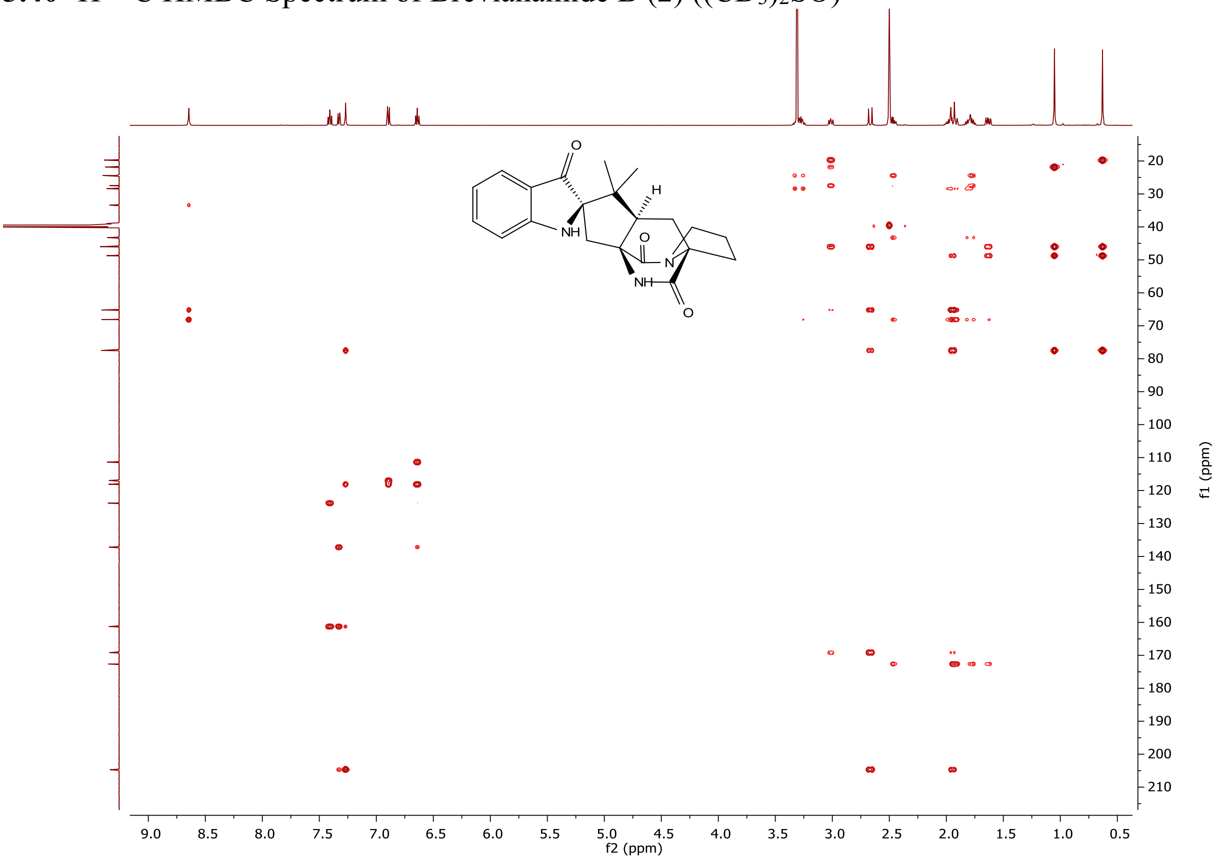

3.41 ^{1}H-^{1}H NOESY Spectrum of Brevianamide B (**2**) ((CD_3)$_2$SO)

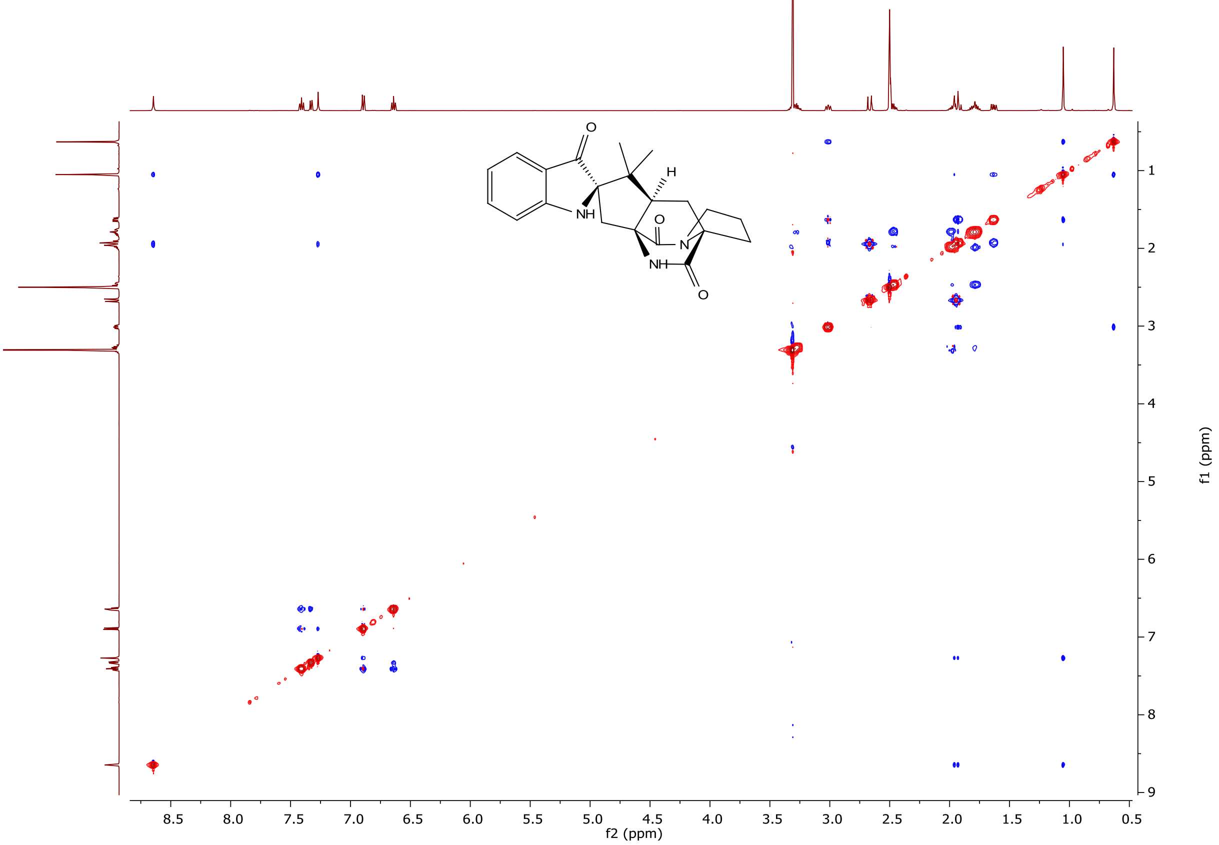

3.42 ^{1}H NMR Spectrum of Compound **20** (500 MHz, $CDCl_3$)

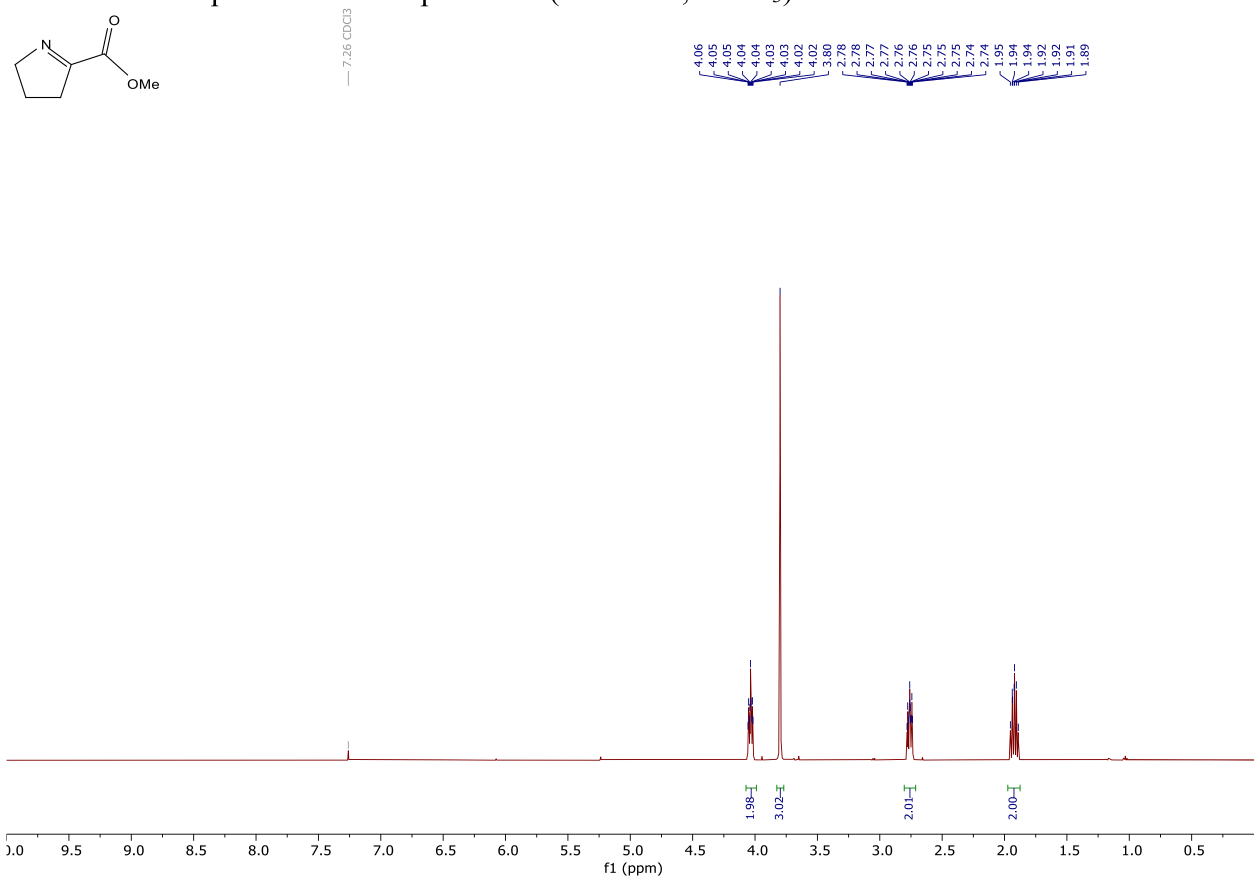

3.43 ^{13}C NMR Spectrum of Compound **20** (126 MHz, $CDCl_3$)

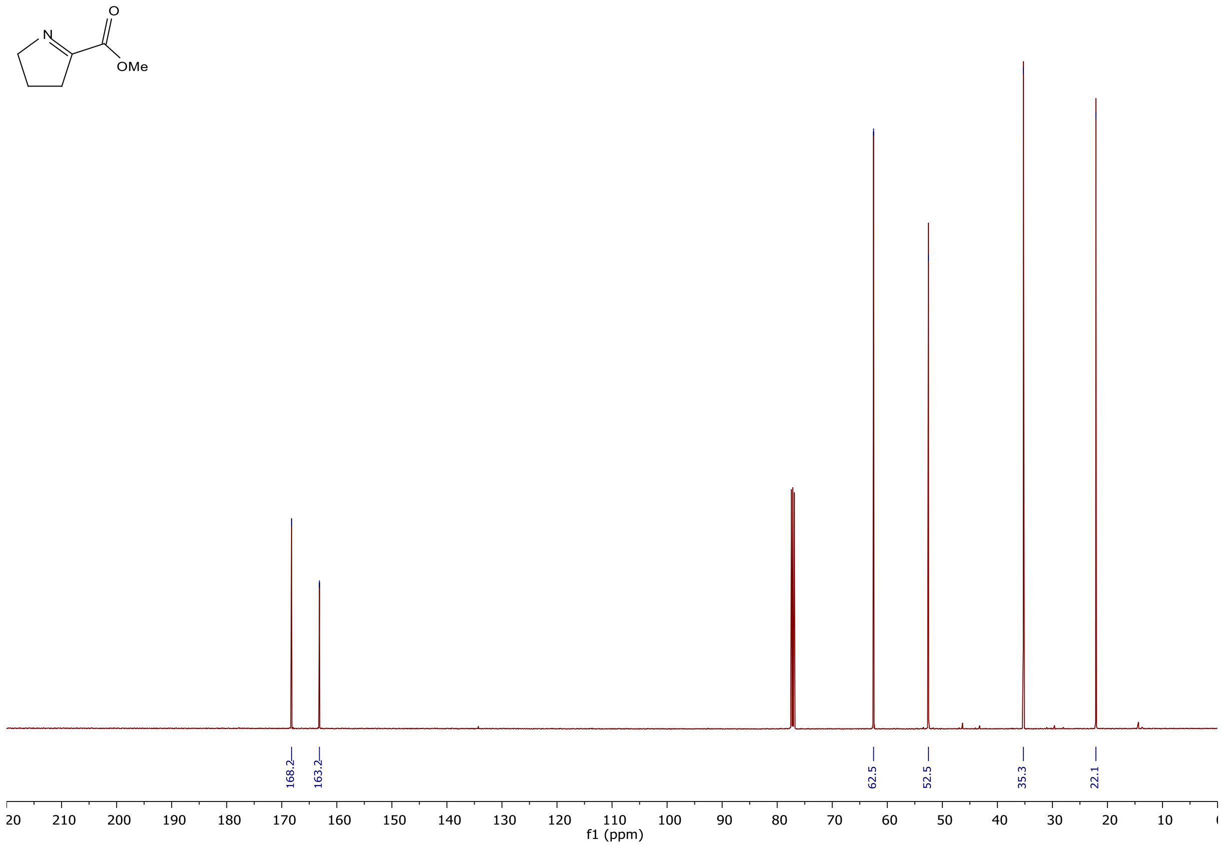

3.44 ^{1}H NMR Spectrum of 3-Chloro-3-methyl-1-butyne (500 MHz, $CDCl_3$)

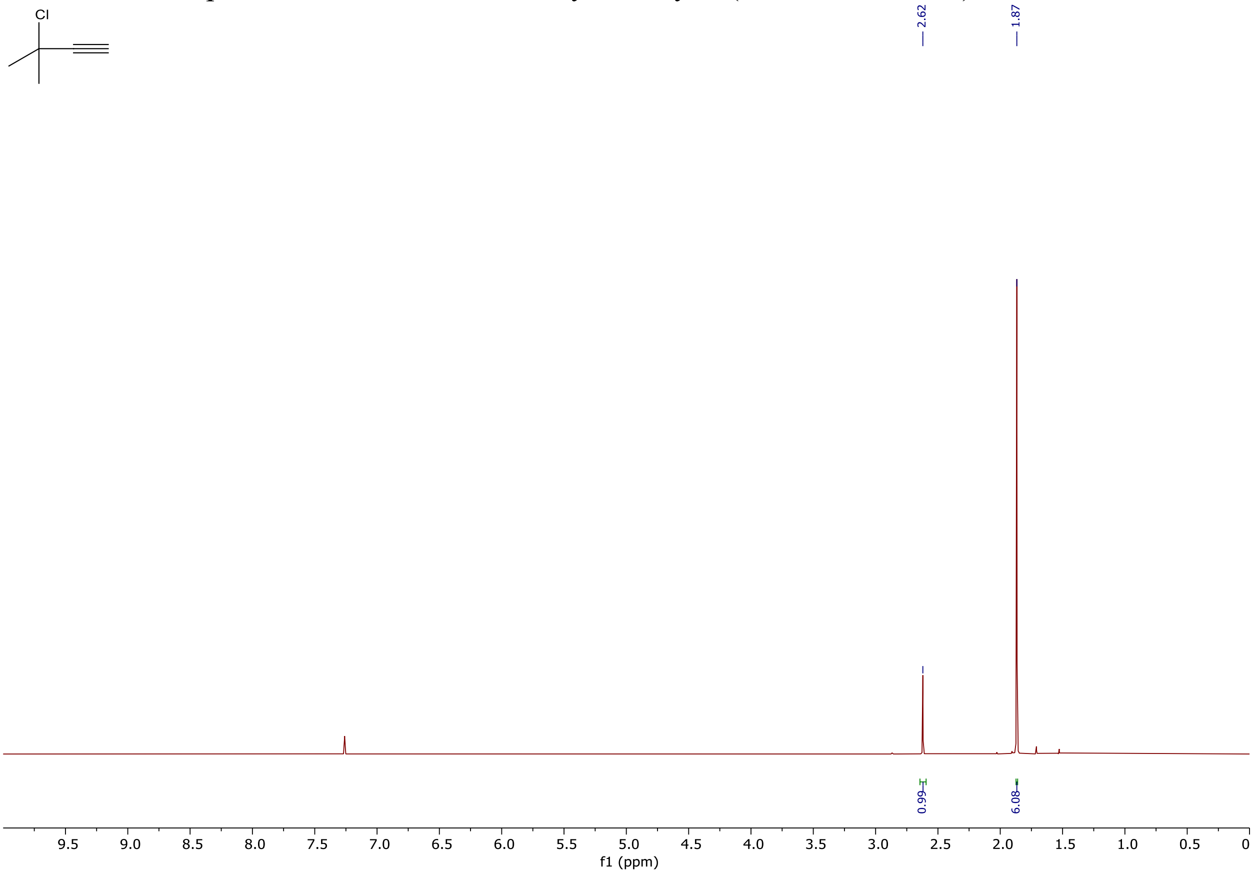

3.45 ^{13}C NMR Spectrum of 3-Chloro-3-methyl-1-butyne (101 MHz, $CDCl_3$)

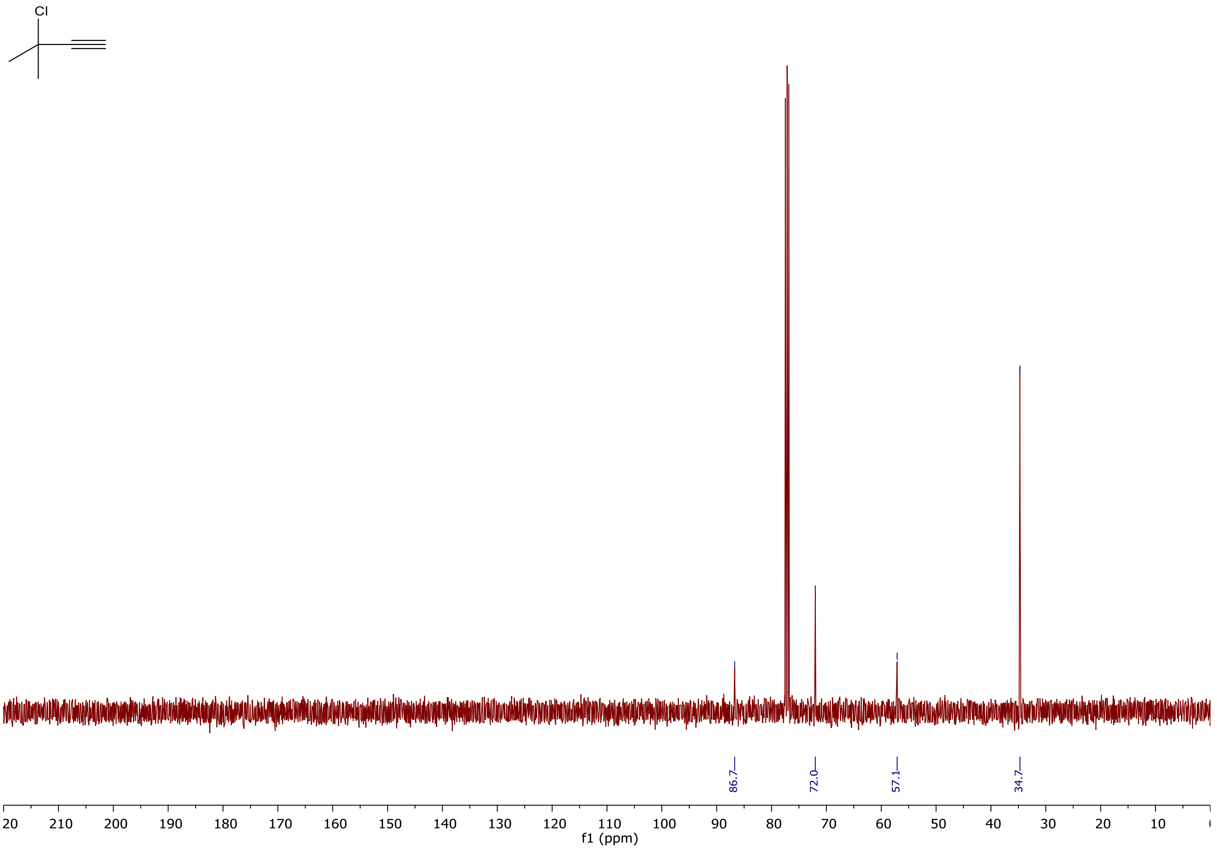

3.46 ^{1}H NMR Spectrum of 1,1-Dimethylallene (500 MHz, $CDCl_3$)

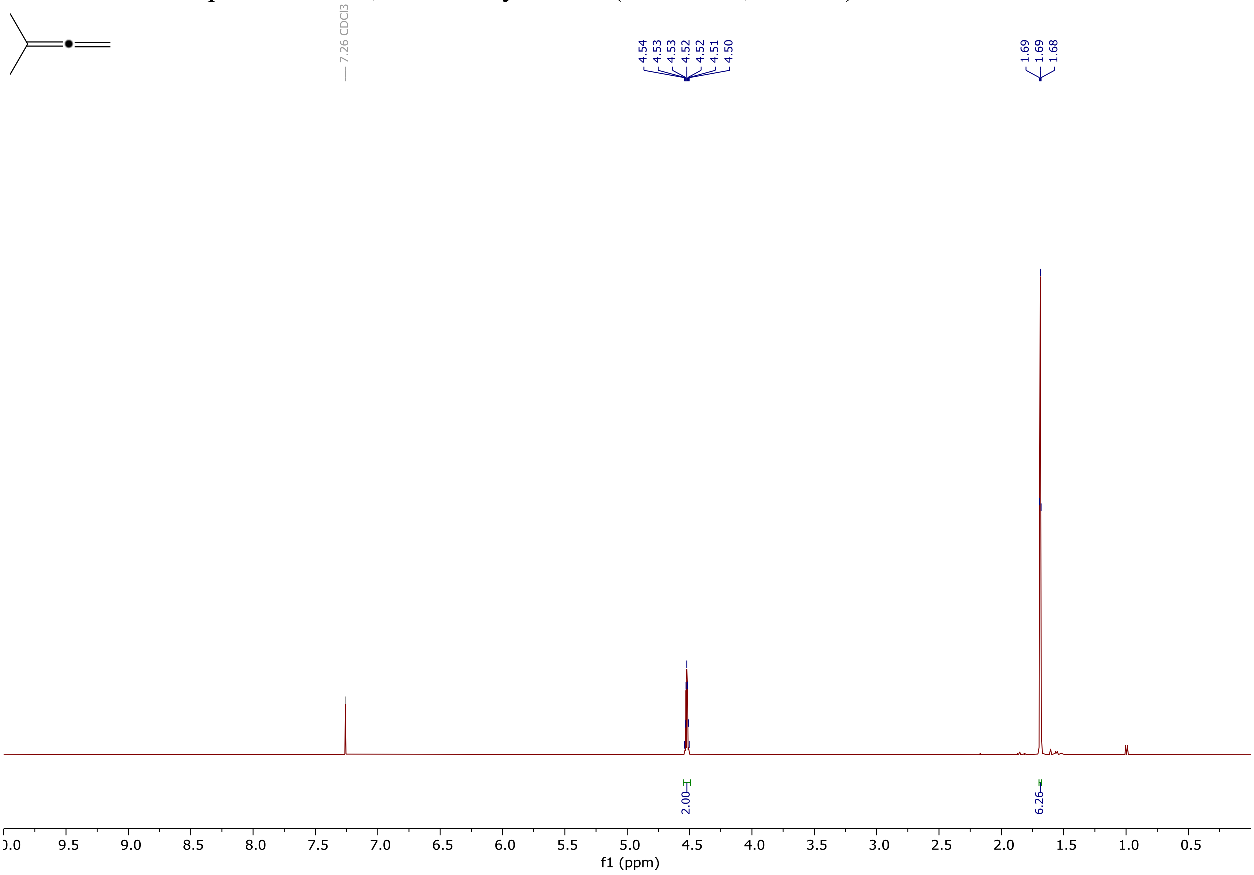

3.47 ^{13}C NMR Spectrum of 1,1-Dimethylallene (151 MHz, $CDCl_3$)

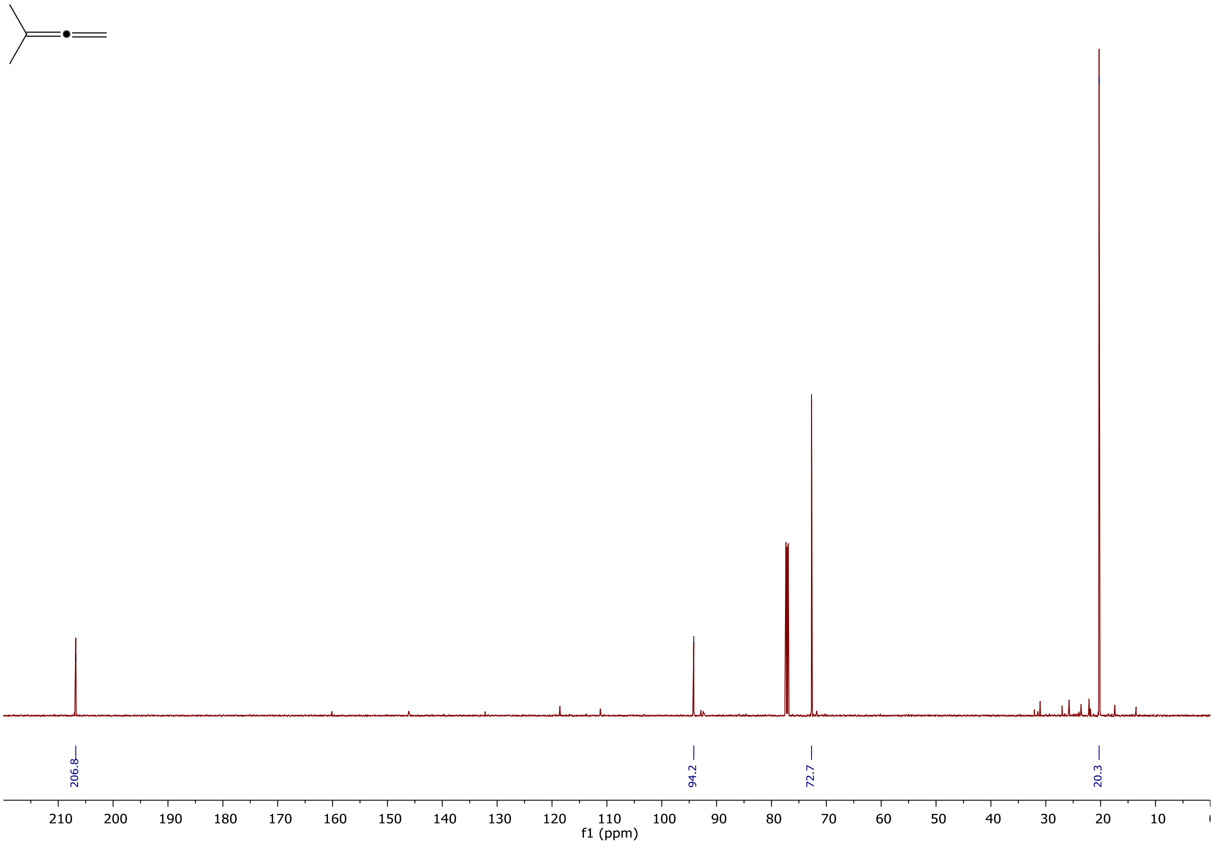

3.48 ^{1}H NMR Spectrum of *t*-BuOCl (500 MHz, $CDCl_3$)

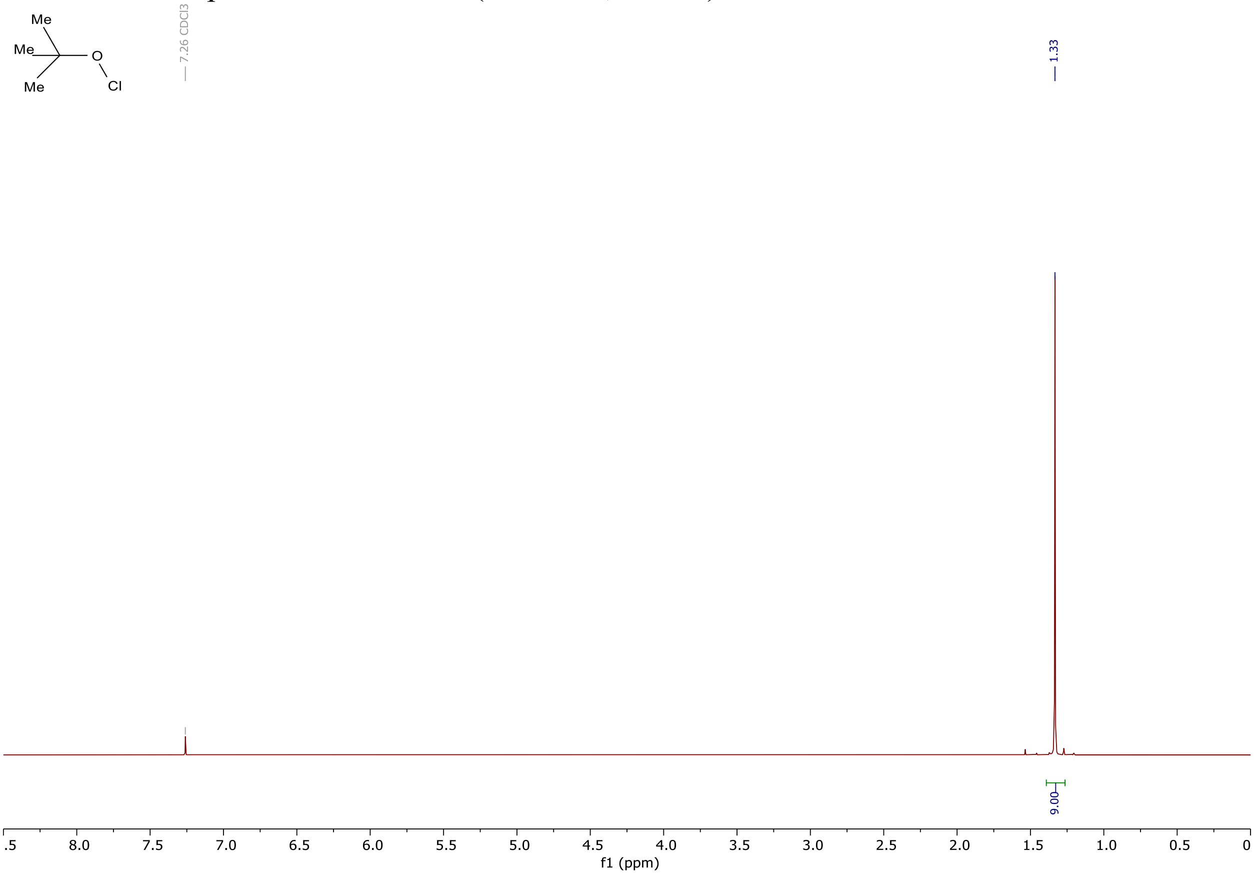

3.49 ^{13}C NMR Spectrum of *t*-BuOCl (126 MHz, $CDCl_3$)

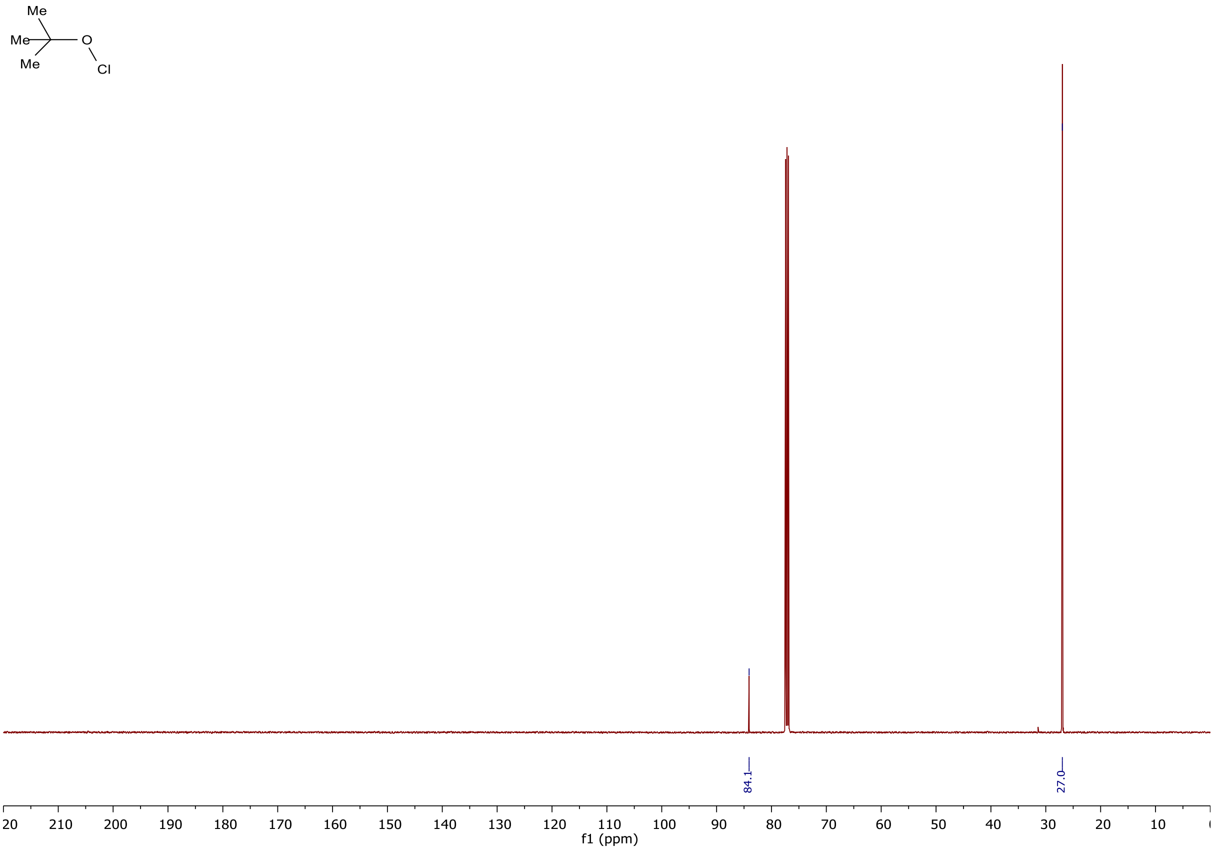

3.50 ^{1}H NMR Spectrum of Compound **23** (500 MHz, $CDCl_3$)

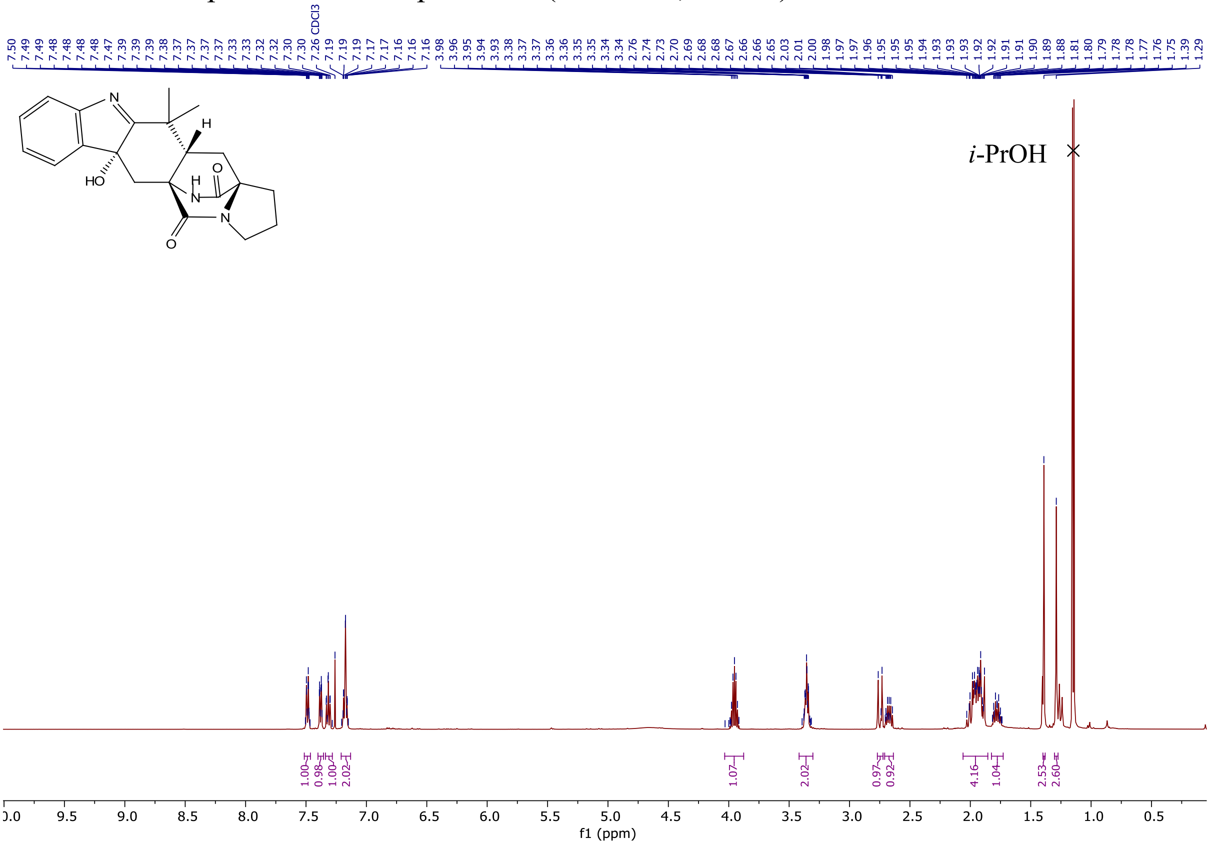

3.51 ^{13}C NMR Spectrum of Compound **23** (126 MHz, $CDCl_3$)

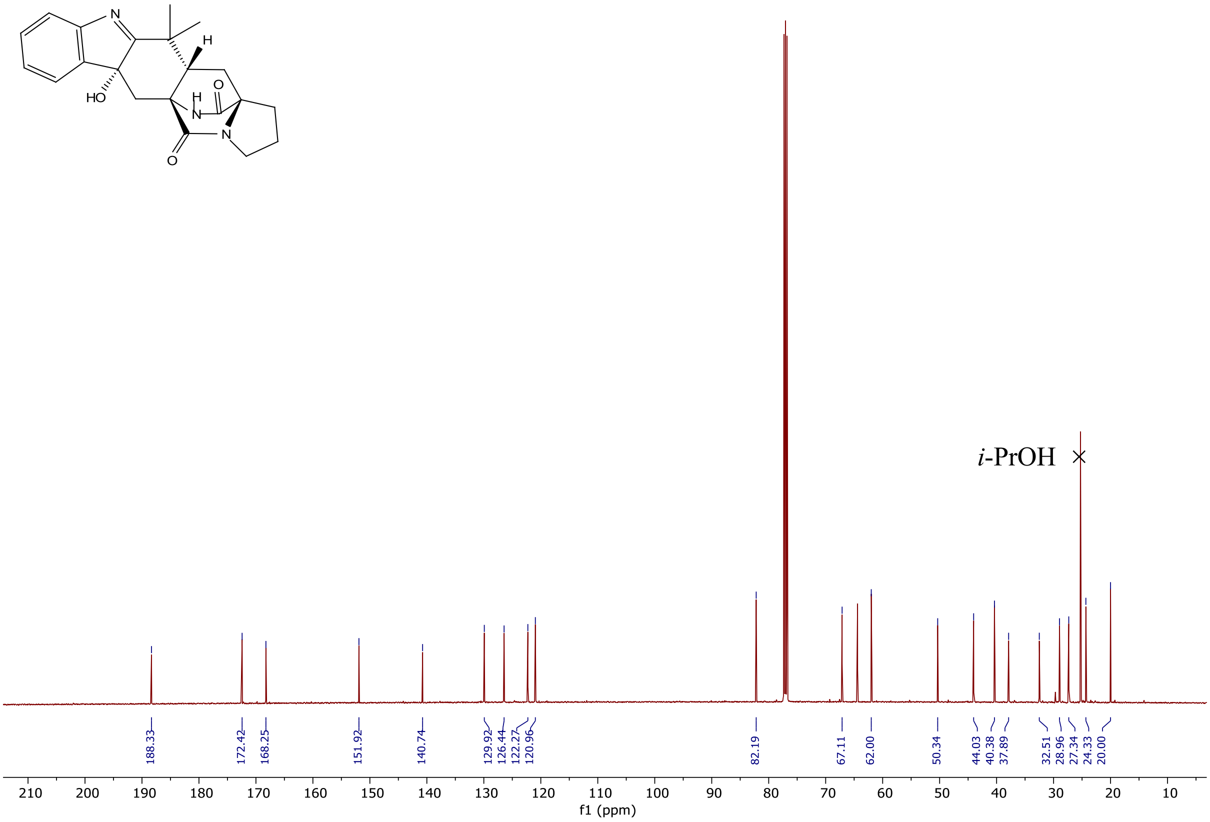

3.52 ^{1}H NMR Spectrum of Compound **23** (500 MHz, $(CD_3)_2SO$)

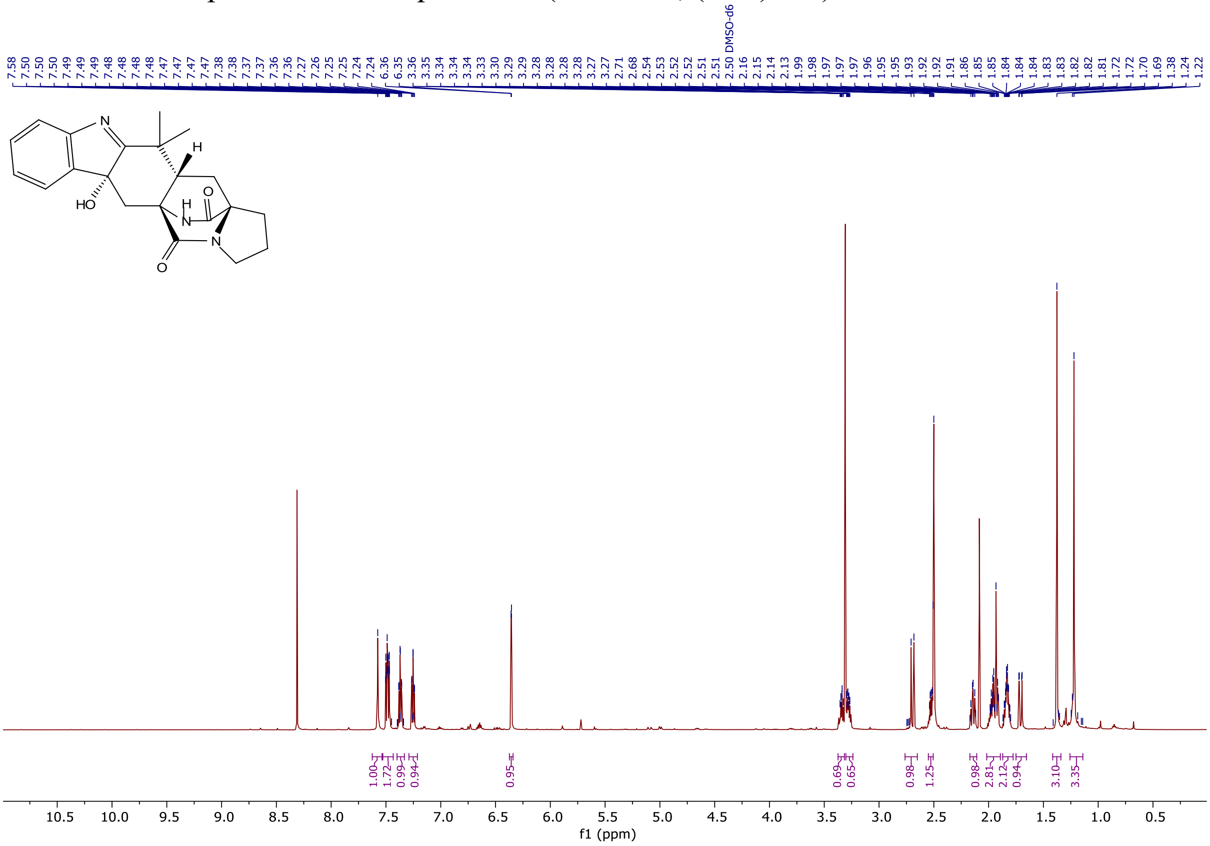

3.53 ^{1}H NMR Spectrum of Compound **24** (500 MHz, $CDCl_3$)

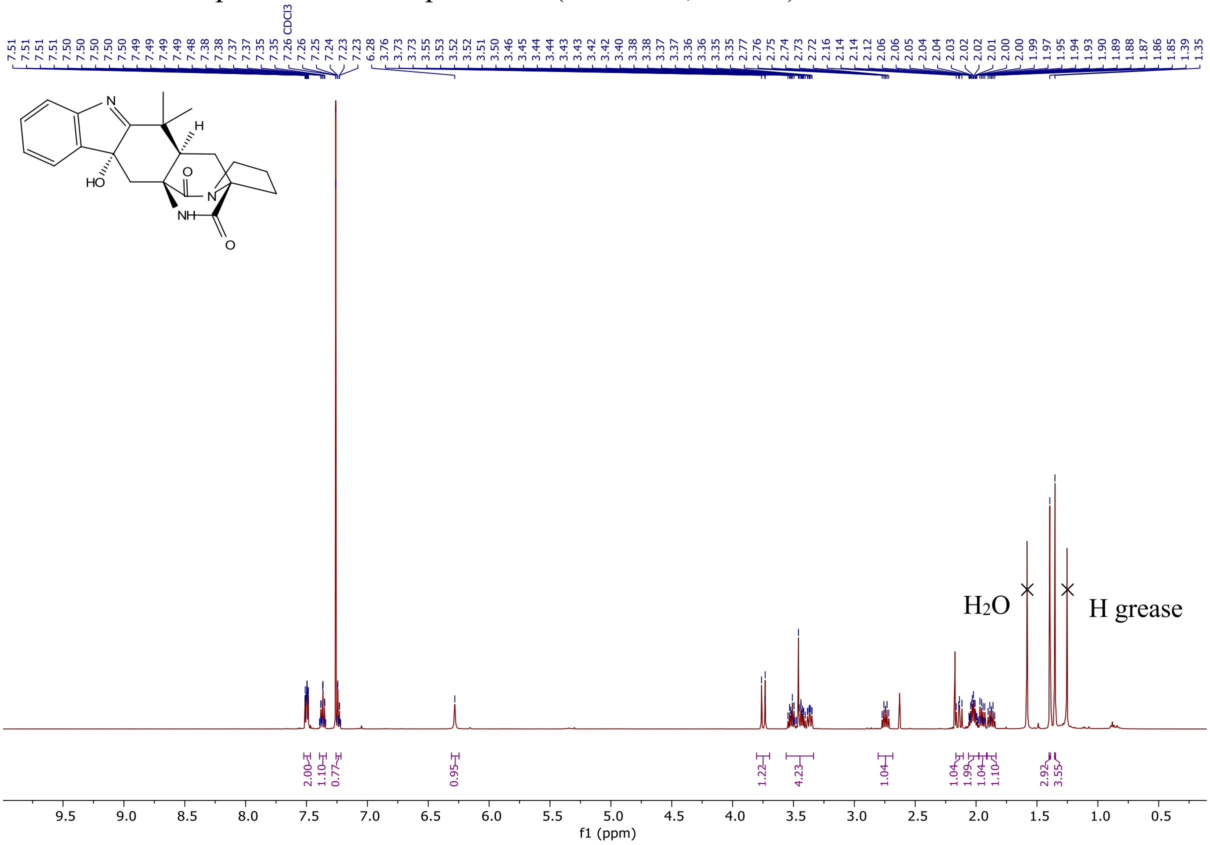

3.54 ^{13}C NMR Spectrum of Compound **24** (126 MHz, $CDCl_3$)

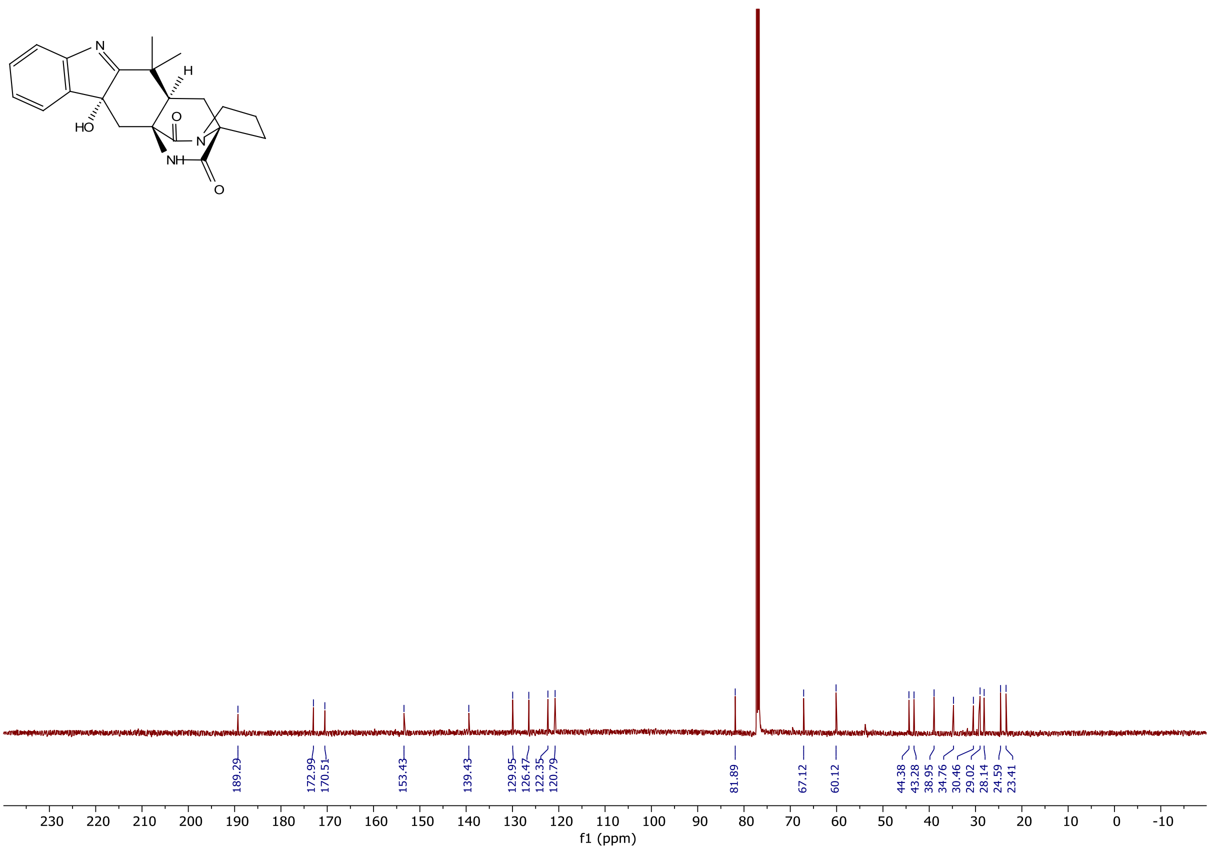

3.55 ^{1}H NMR Spectrum of Compound **24** (500 MHz, $(CD_3)_2SO$)

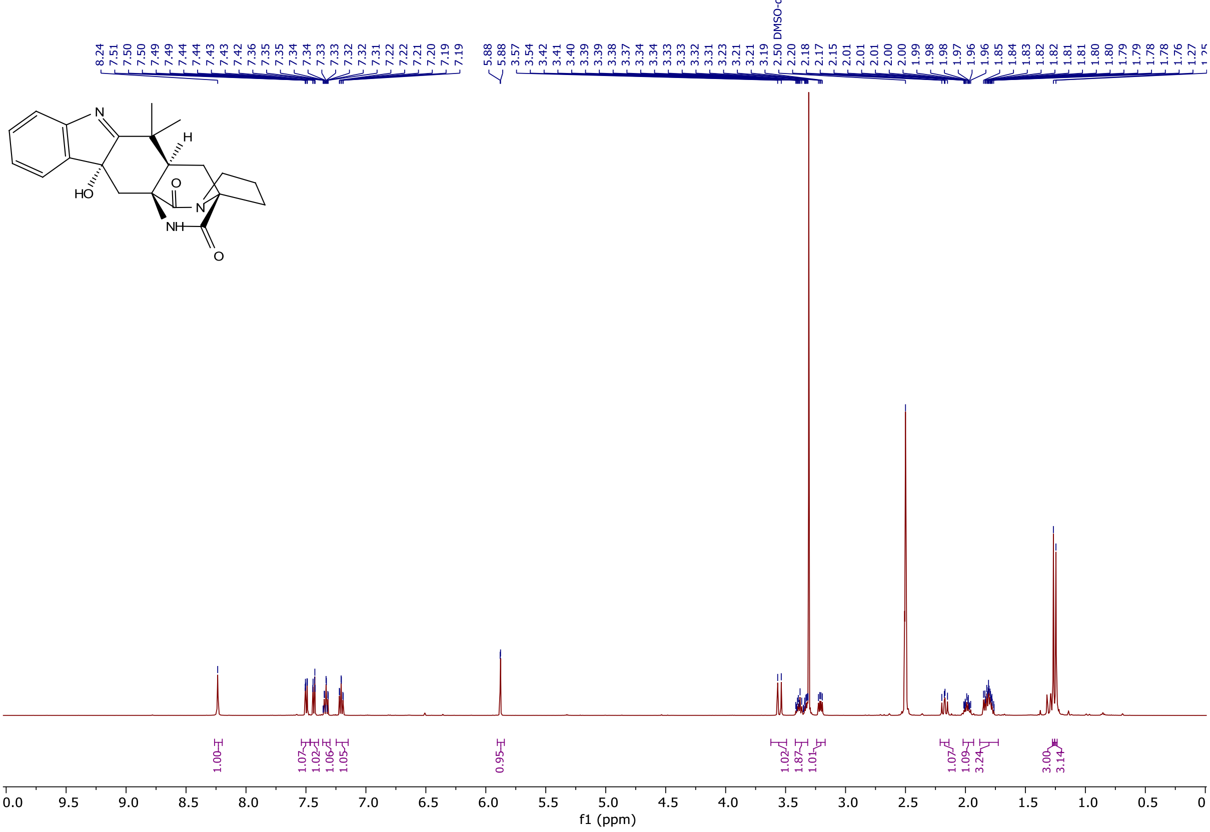

3.56 ^{13}C NMR Spectrum of Compound **24** (126 MHz, $(CD_3)_2SO$)

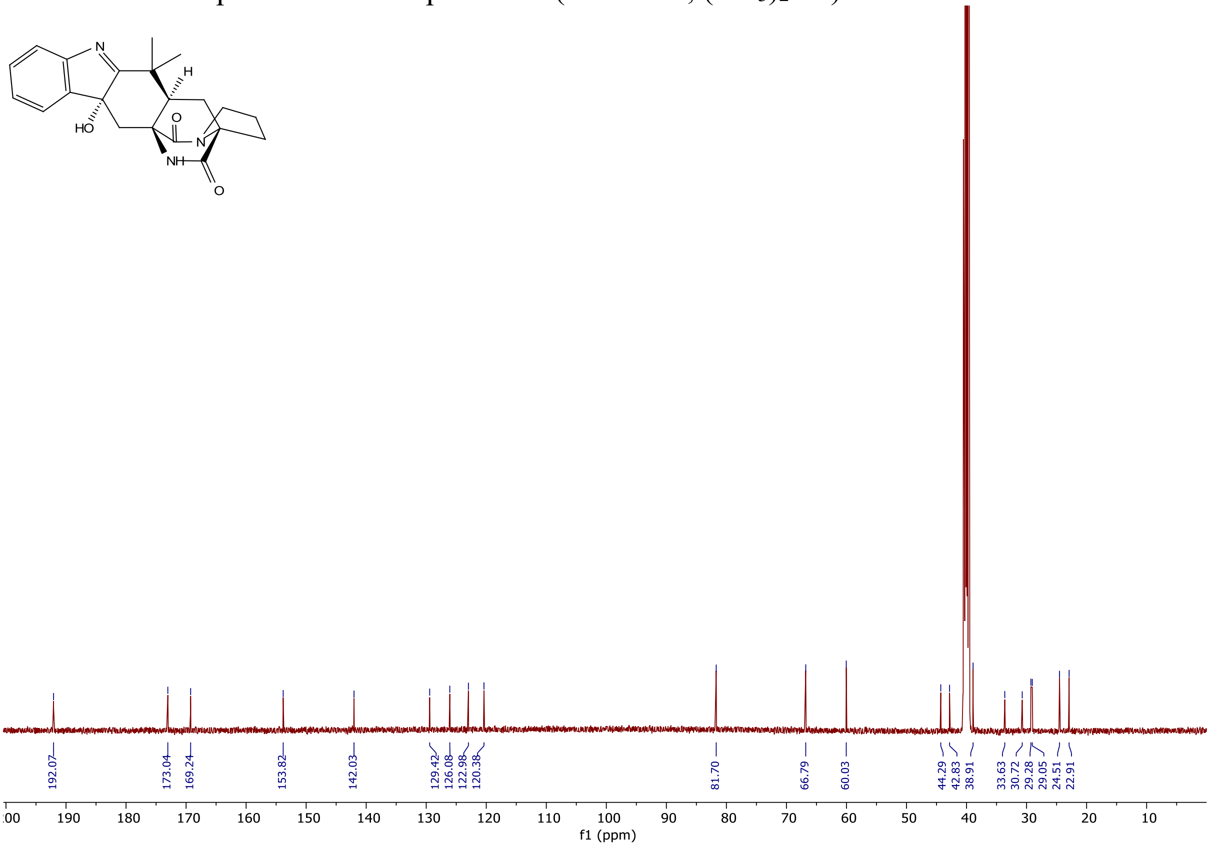

3.57 ^{1}H-^{1}H COSY Spectrum of Compound **24** ($CDCl_3$)

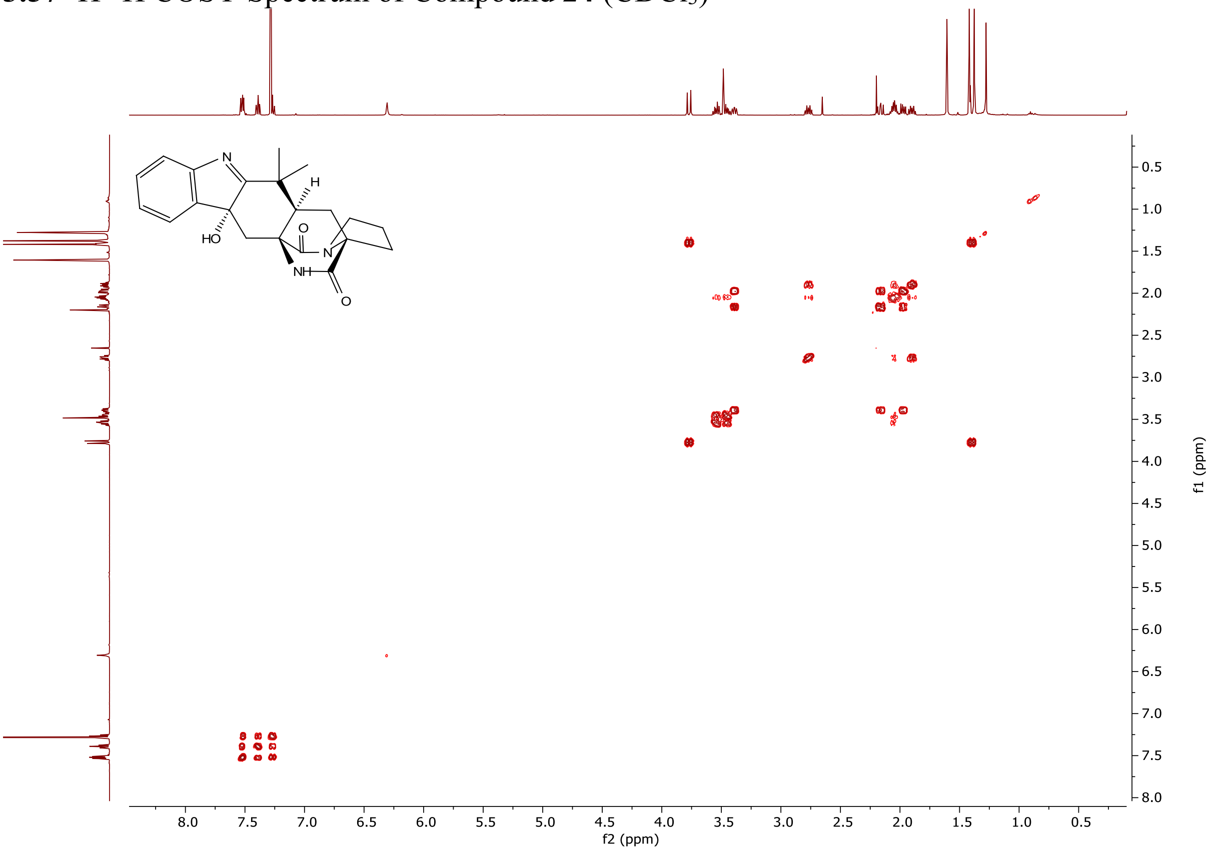

3.58 ^{1}H-^{13}C HSQC Spectrum of Compound **24** ($CDCl_3$)

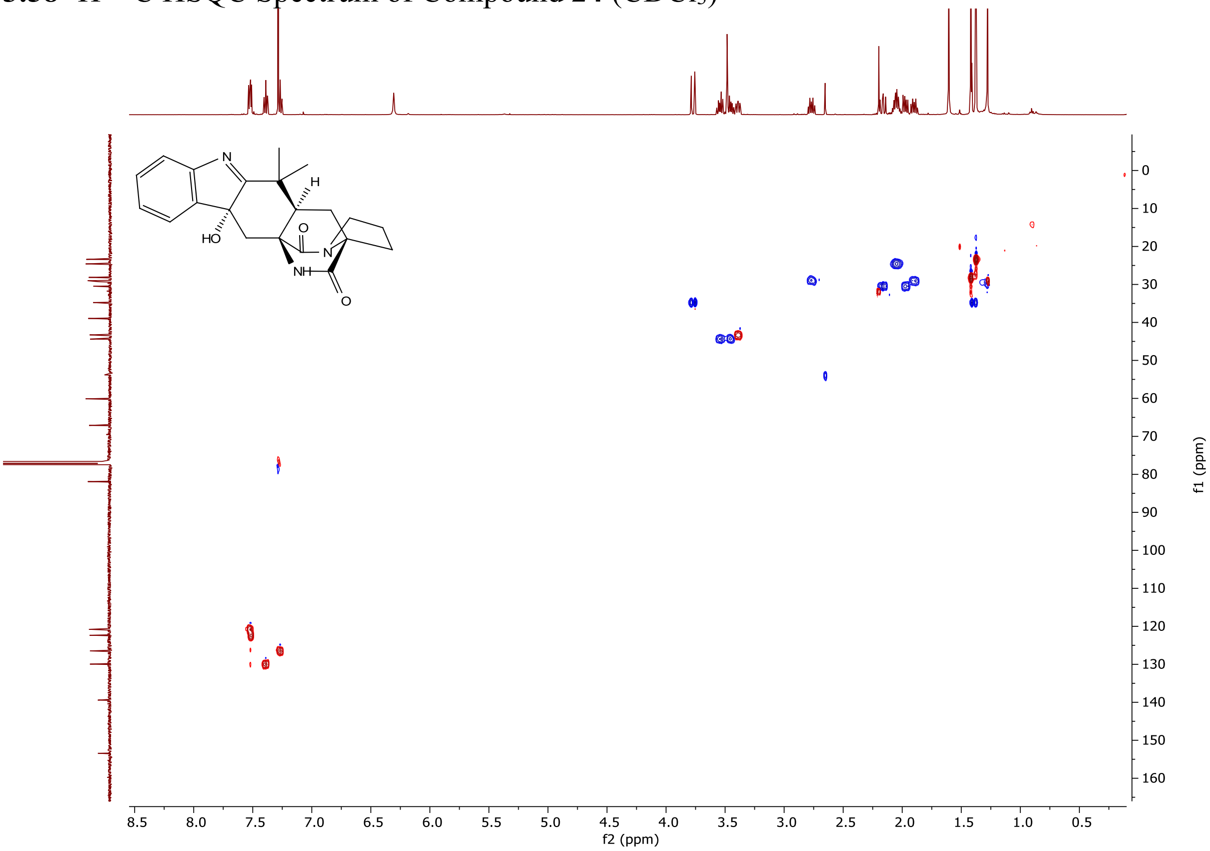

3.59 ^{1}H-^{13}C HMBC Spectrum of Compound **24** ($CDCl_3$)

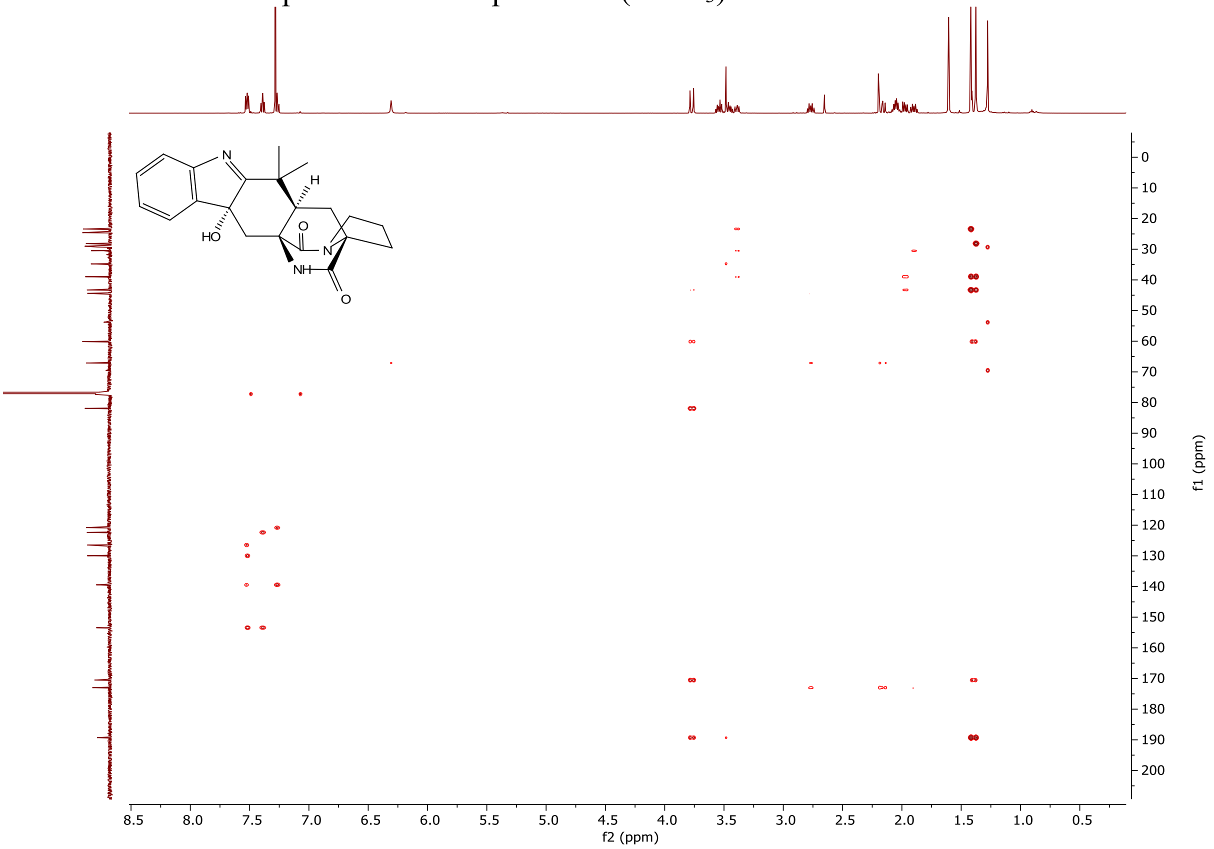

3.60 ^{1}H-^{1}H NOESY Spectrum of Compound **24** ($CDCl_3$)

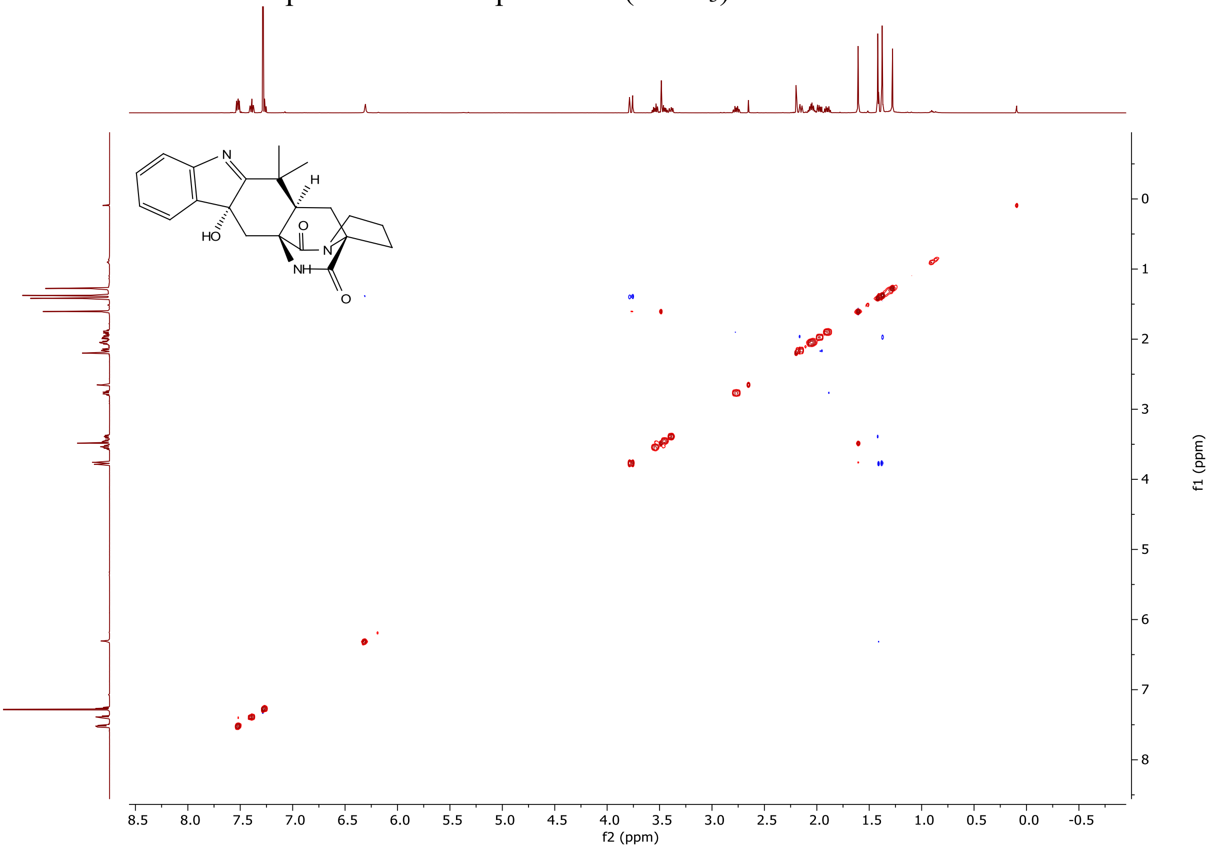

3.61 ^{1}H NMR Spectrum of Compound **11** (500 MHz, $CDCl_3$)

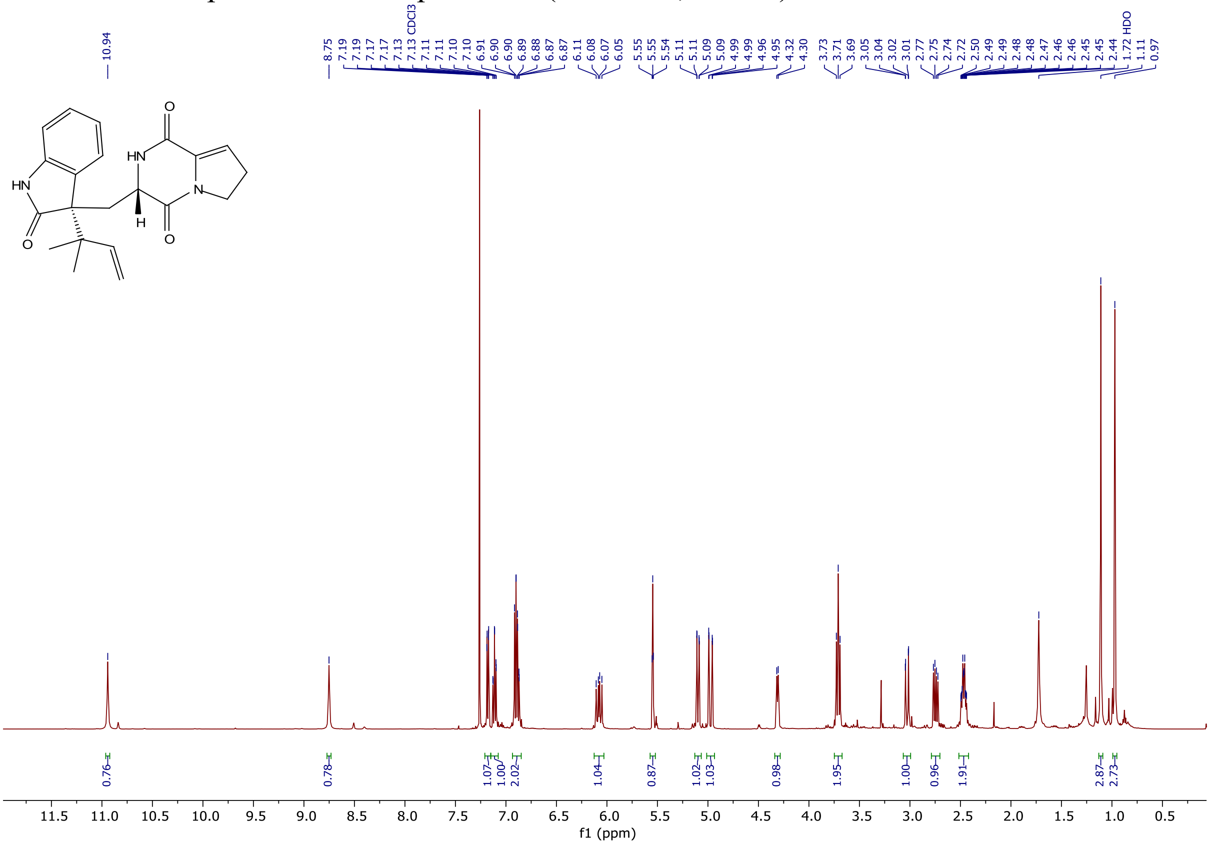

3.62 ^{13}C NMR Spectrum of Compound **11** (126 MHz, $CDCl_3$)

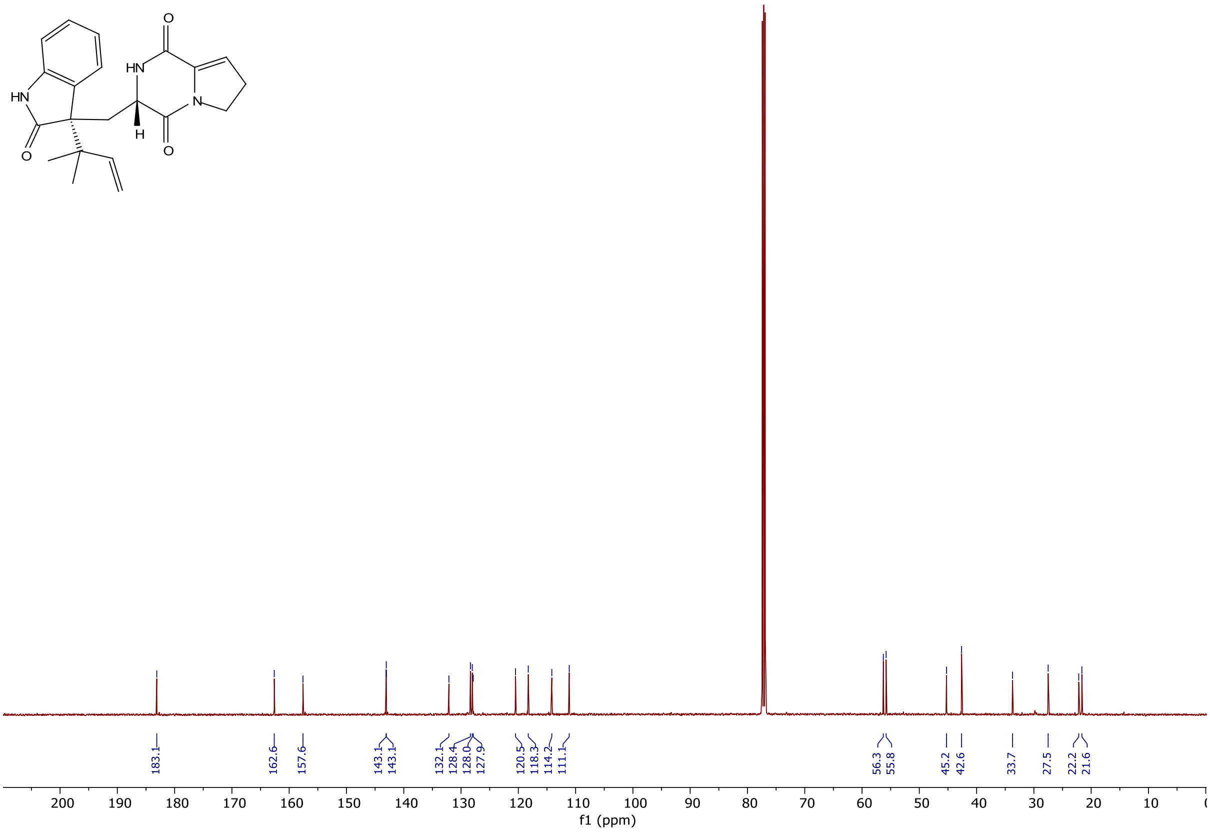

3.63 ^{1}H-^{1}H COSY Spectrum of Compound **11** ($CDCl_3$)

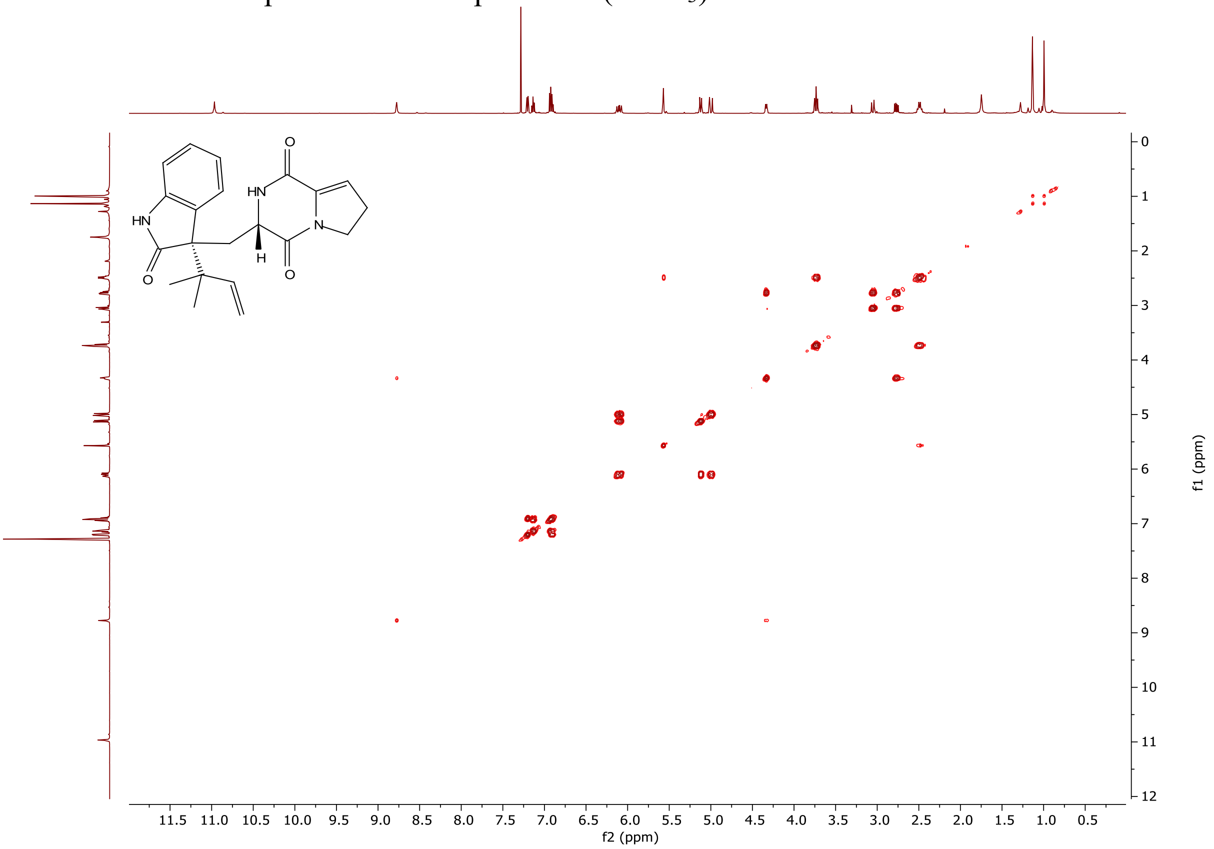

3.64 ^{1}H-^{13}C HSQC Spectrum of Compound **11** ($CDCl_3$)

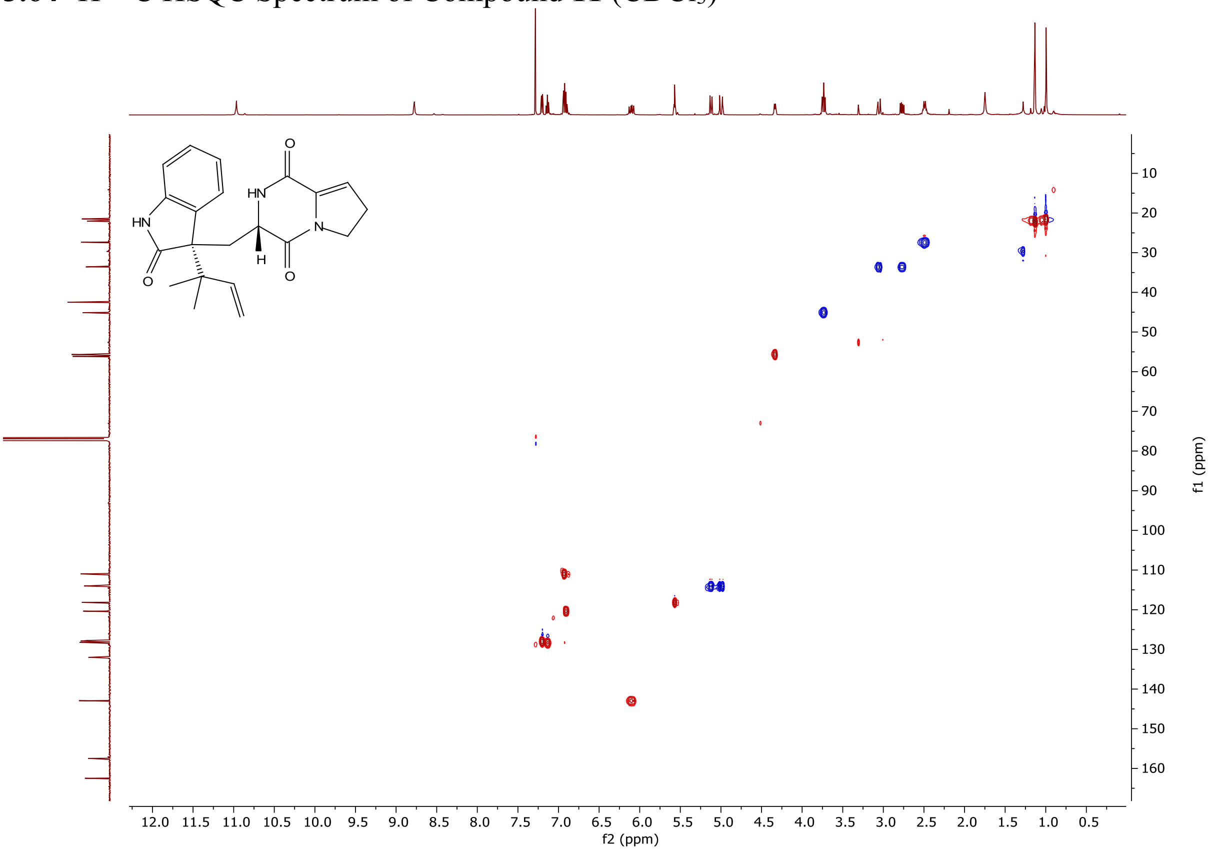

3.65 ^{1}H-^{13}C HMBC Spectrum of Compound **11** ($CDCl_3$)

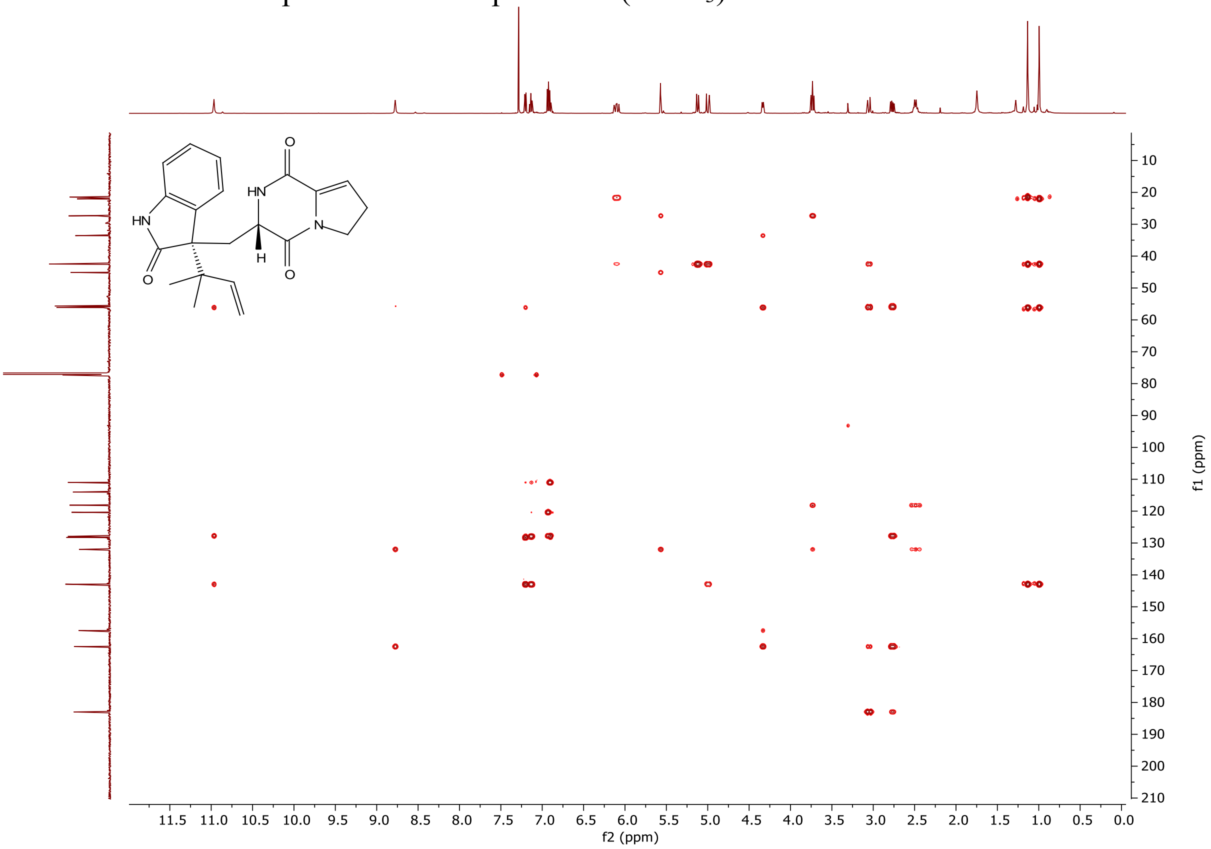

3.66 ^{1}H-^{1}H NOESY Spectrum of Compound **11** ($CDCl_3$)

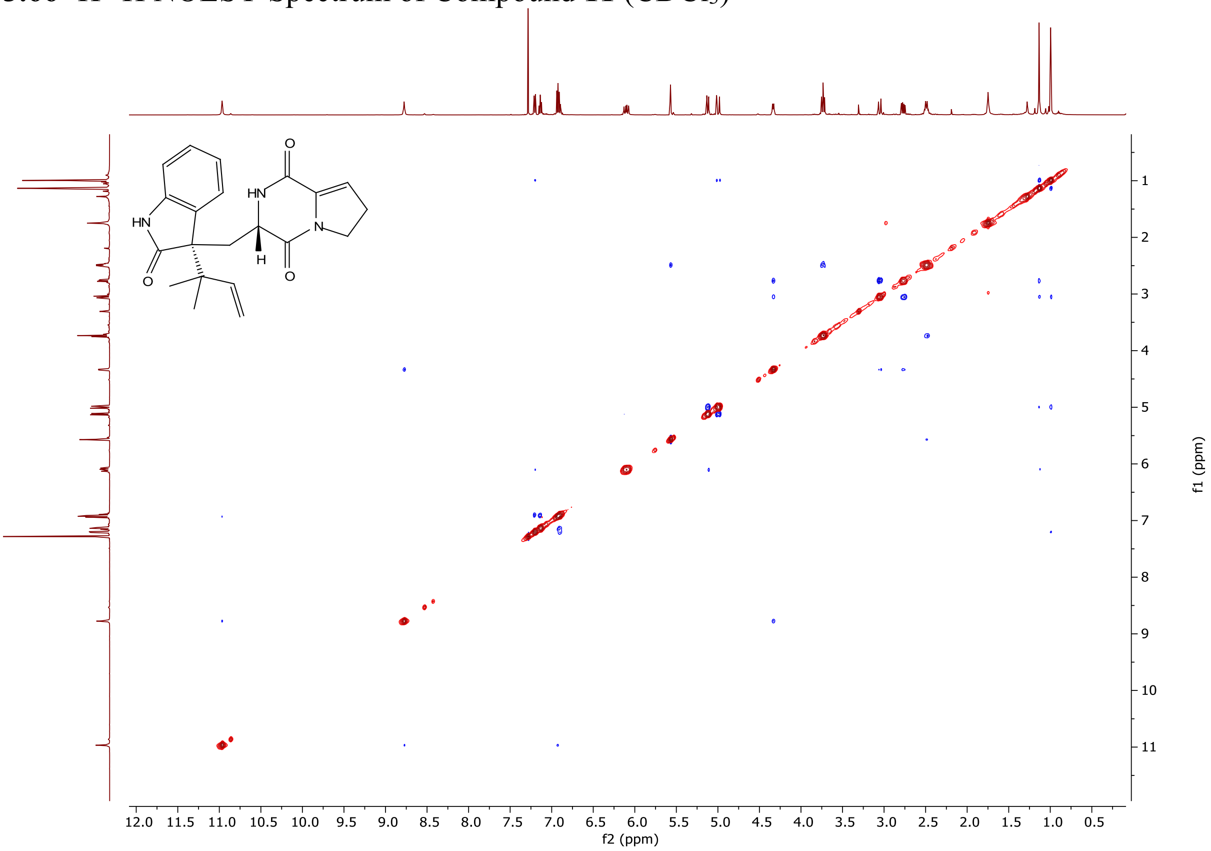

3.67 ^{1}H NMR Spectrum of Compound **27** (500 MHz, $CDCl_3$)

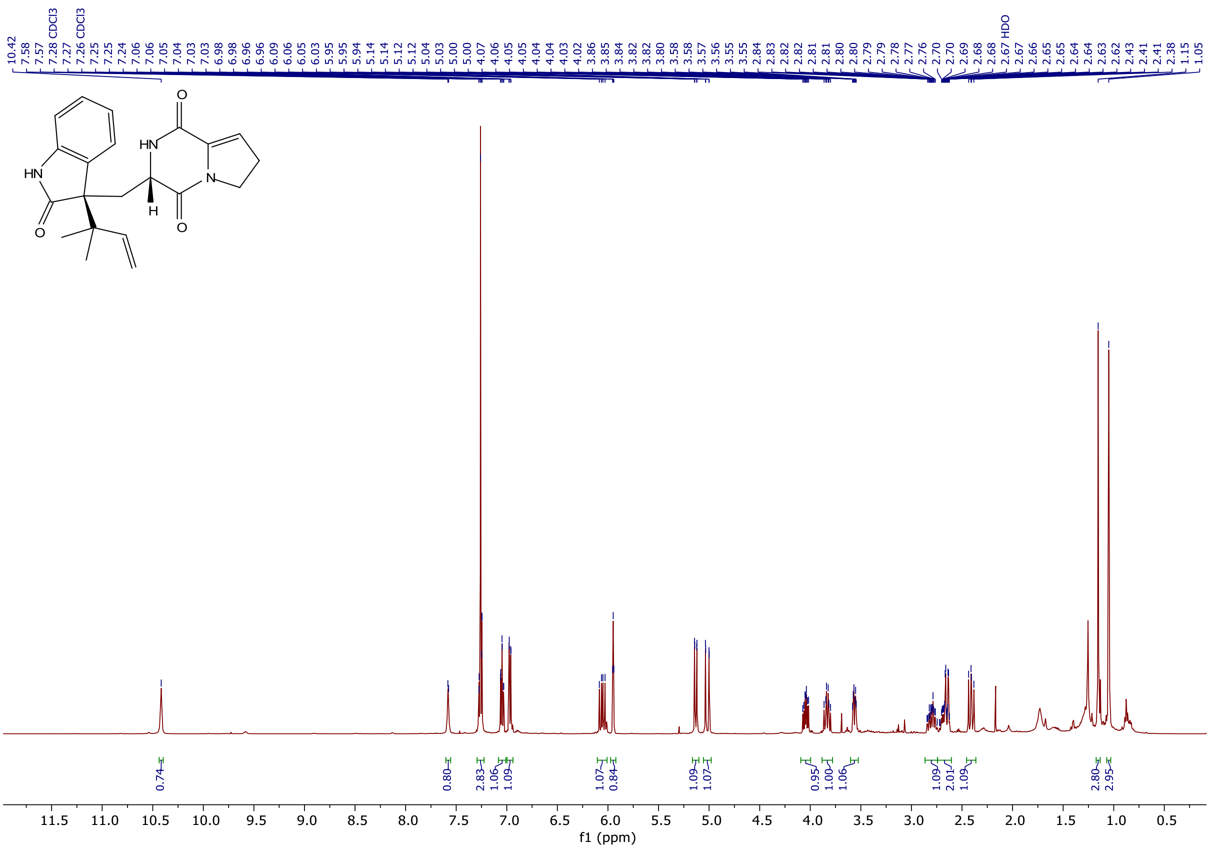

3.68 ^{13}C NMR Spectrum of Compound **27** (126 MHz, $CDCl_3$)

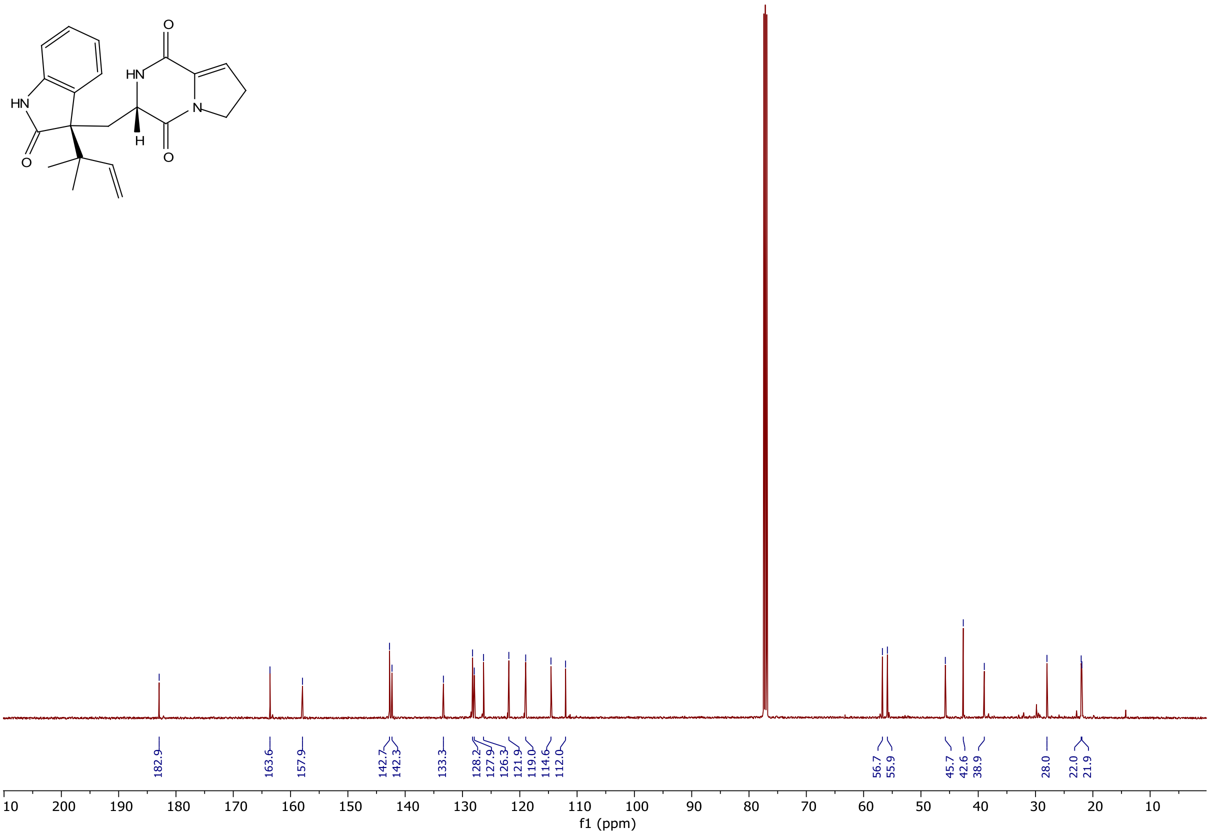

3.69 ¹H-¹H COSY Spectrum of Compound **27** ($CDCl_3$)

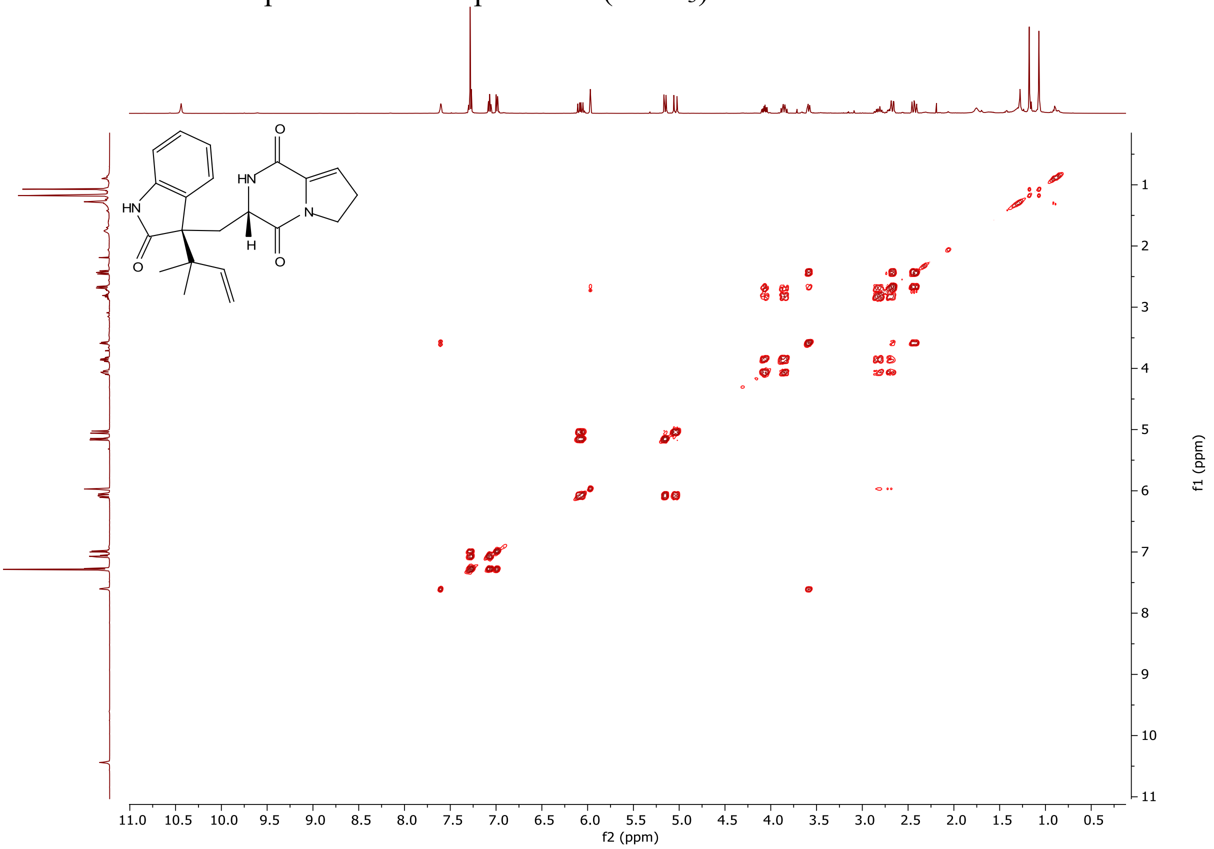

3.70 ¹H-¹³C HSQC Spectrum of Compound **27** ($CDCl_3$)

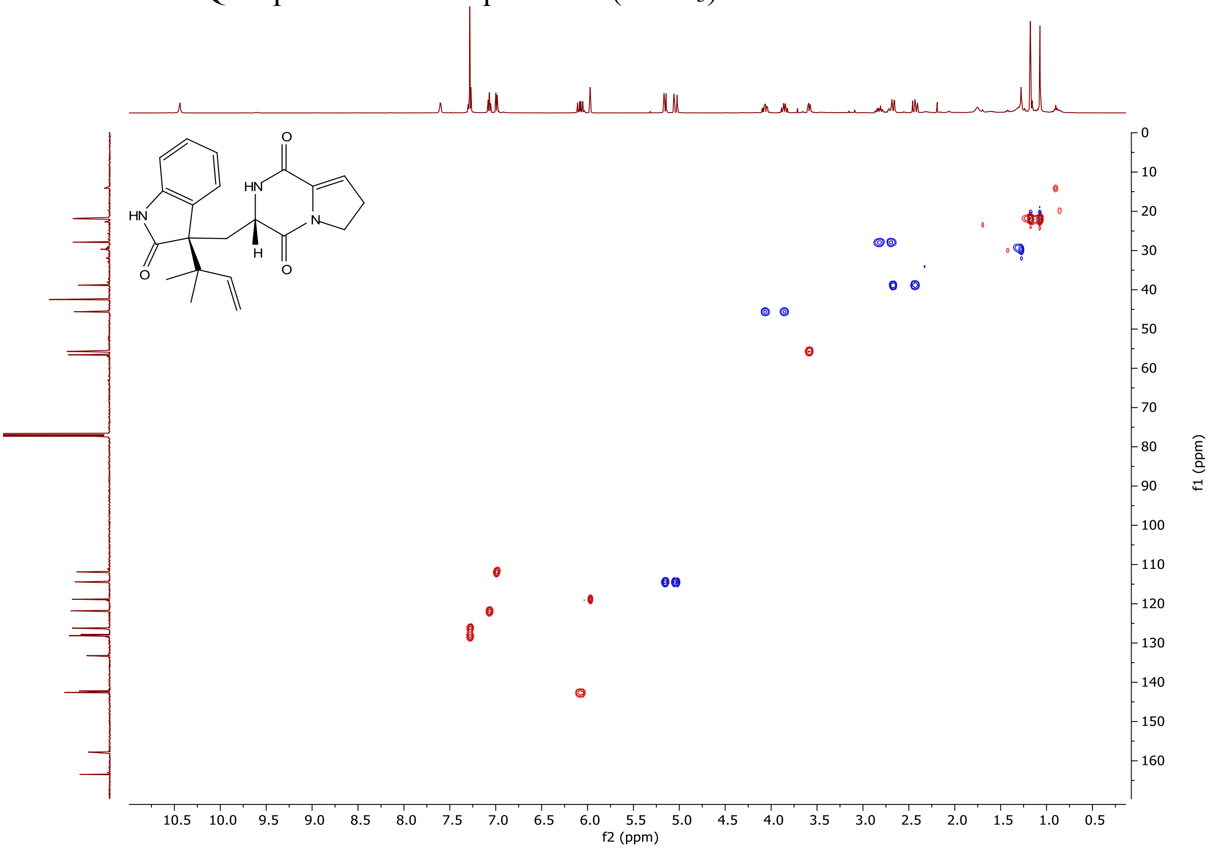

3.71 ^{1}H-^{13}C HMBC Spectrum of Compound **27** ($CDCl_3$)

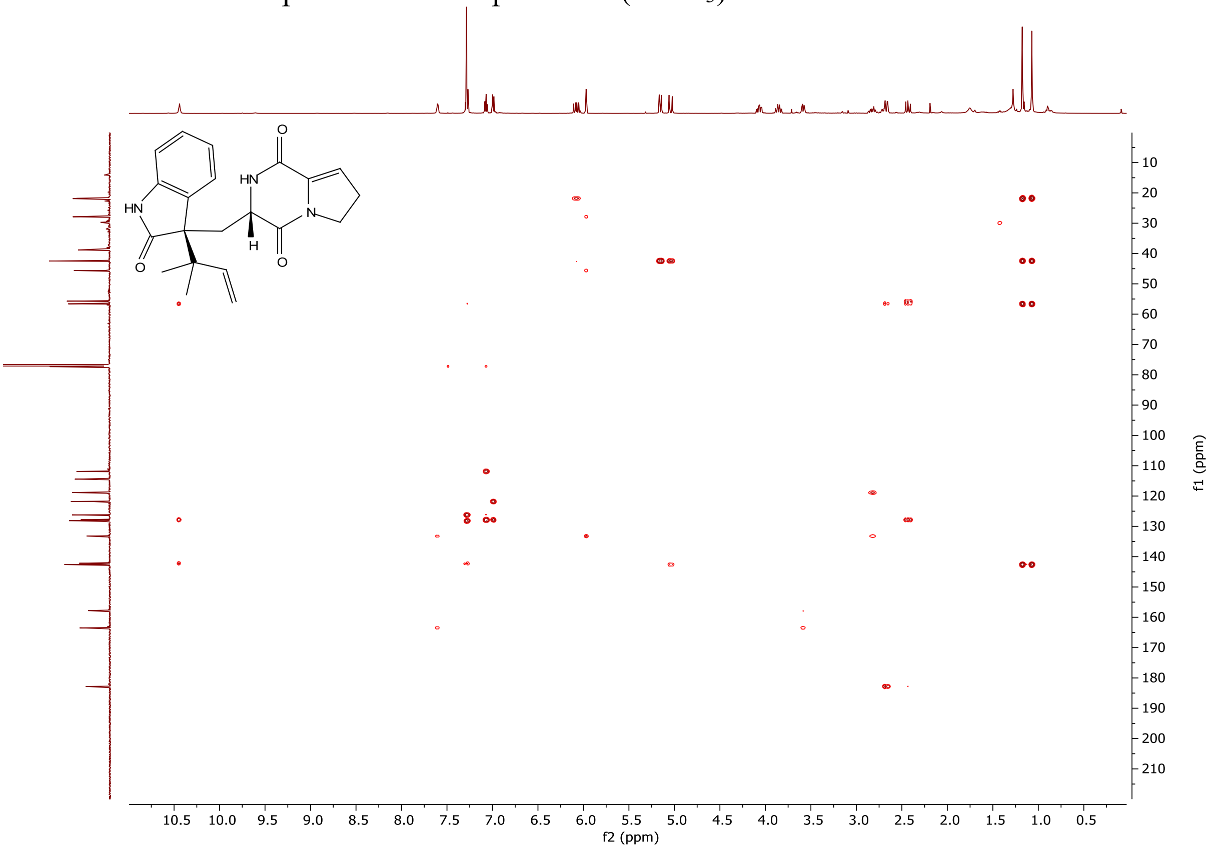

3.72 ^{1}H-^{1}H NOESY Spectrum of Compound **27** ($CDCl_3$)

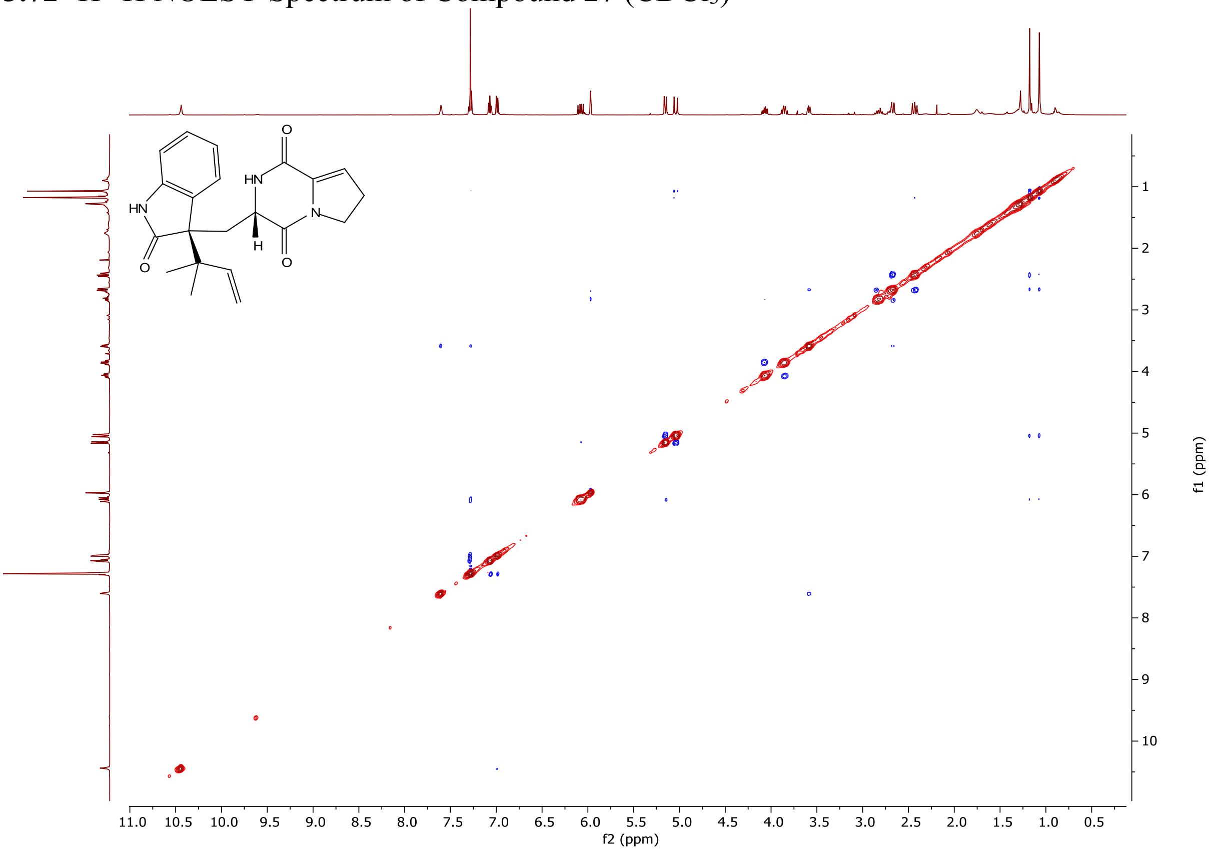

3.73 ^{1}H NMR Spectrum of Brevianamide Y (**4**) (500 MHz, $(CD_3)_2SO$)

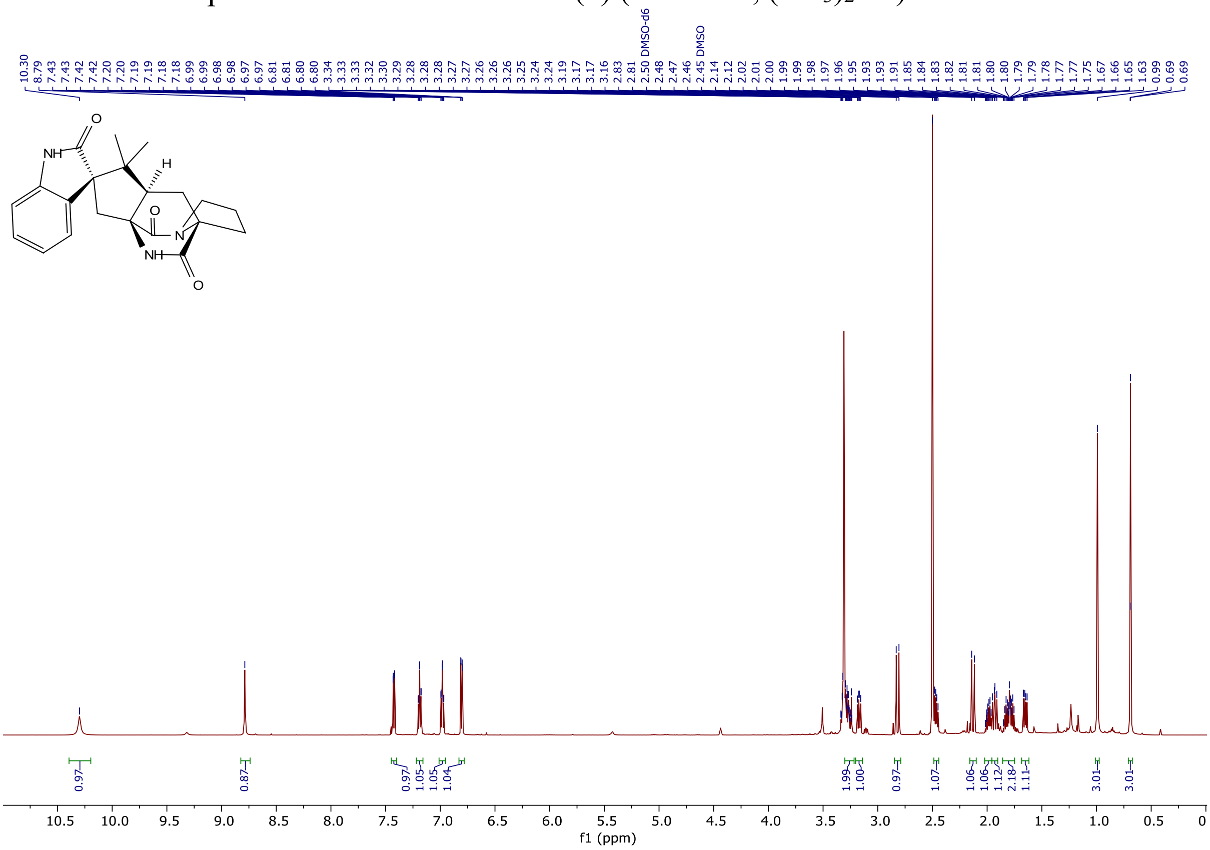

3.74 ^{13}C NMR Spectrum of Brevianamide Y (**4**) (126 MHz, $(CD_3)_2SO$)

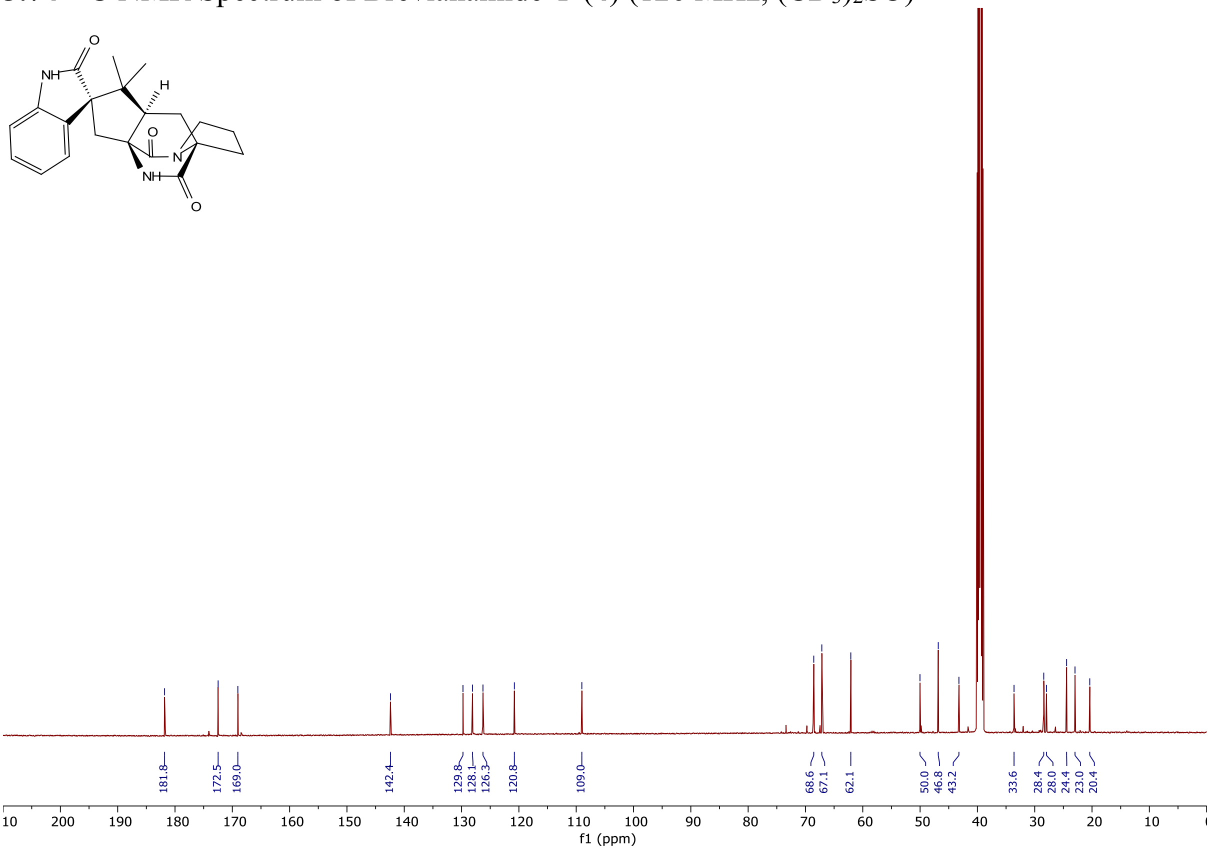

3.75 ^{1}H-^{1}H COSY Spectrum of Brevianamide Y (**4**) ($(CD_3)_2SO$)

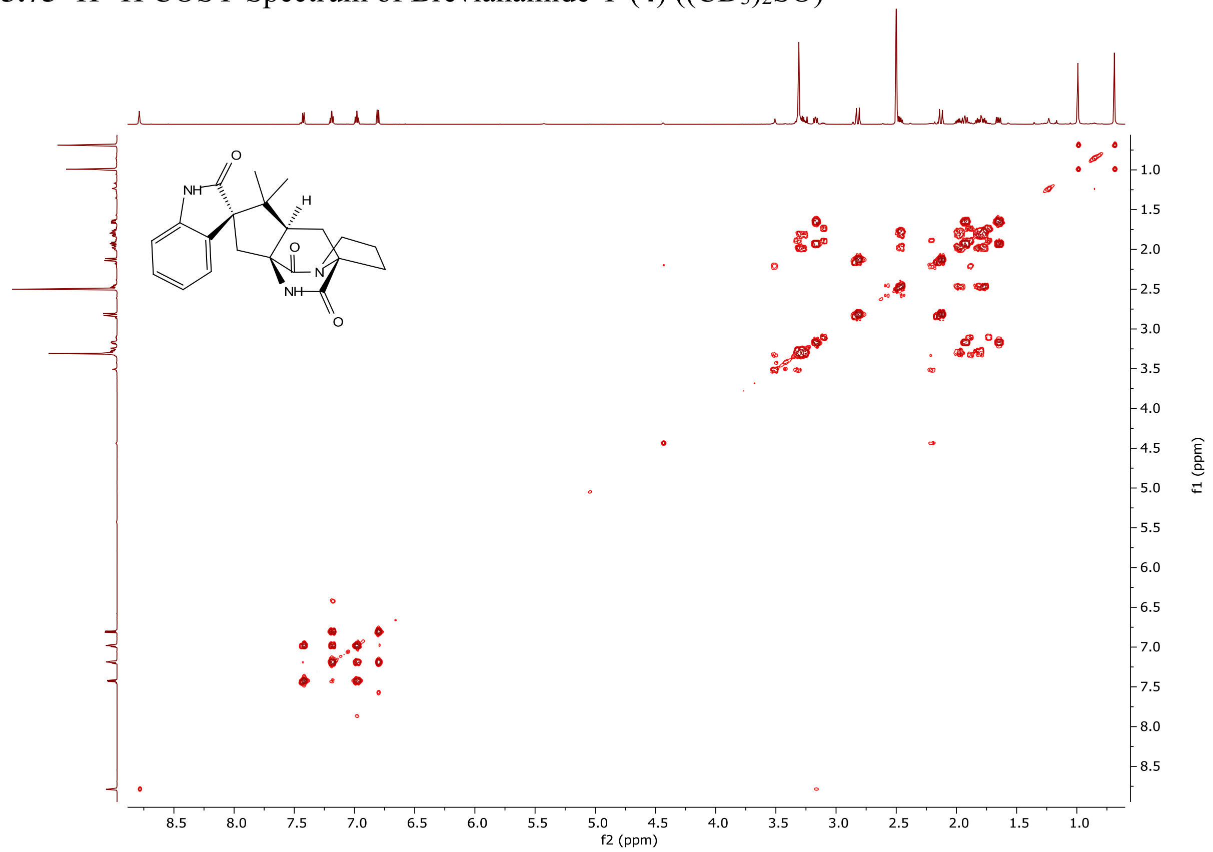

3.76 ^{1}H-^{13}C HSQC Spectrum of Brevianamide Y (**4**) ($(CD_3)_2SO$)

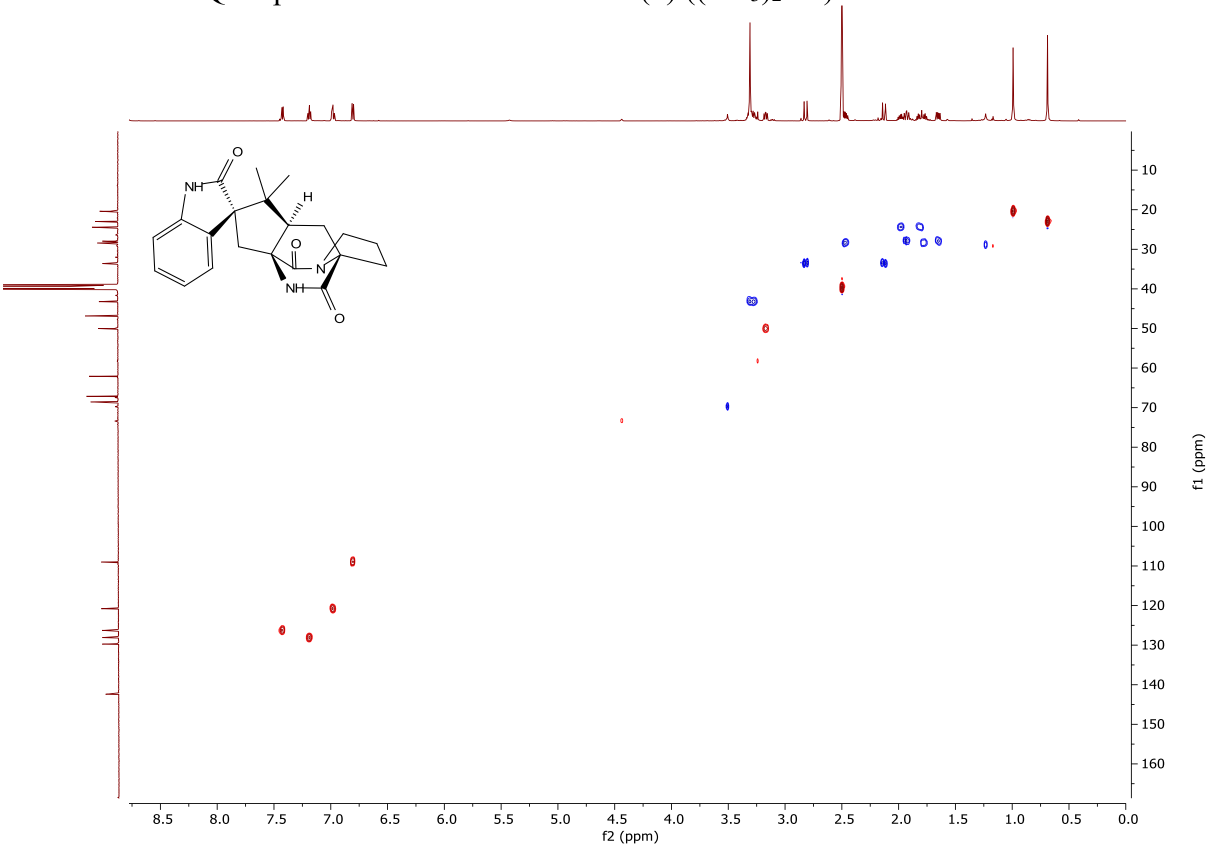

3.77 ^{1}H-^{13}C HMBC Spectrum of Brevianamide Y (**4**) (($(CD_3)_2SO$))

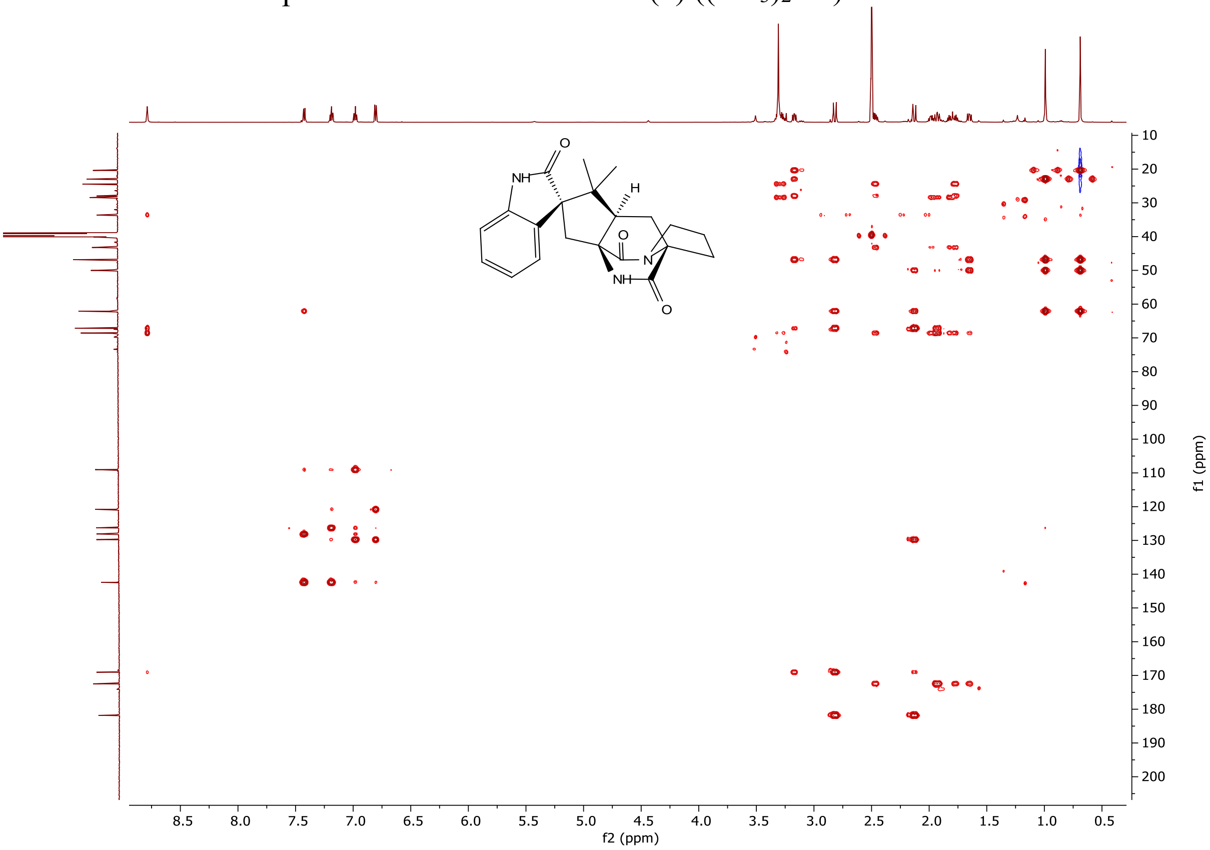

3.78 ^{1}H-^{1}H NOESY Spectrum of Brevianamide Y (**4**) (($(CD_3)_2SO$))

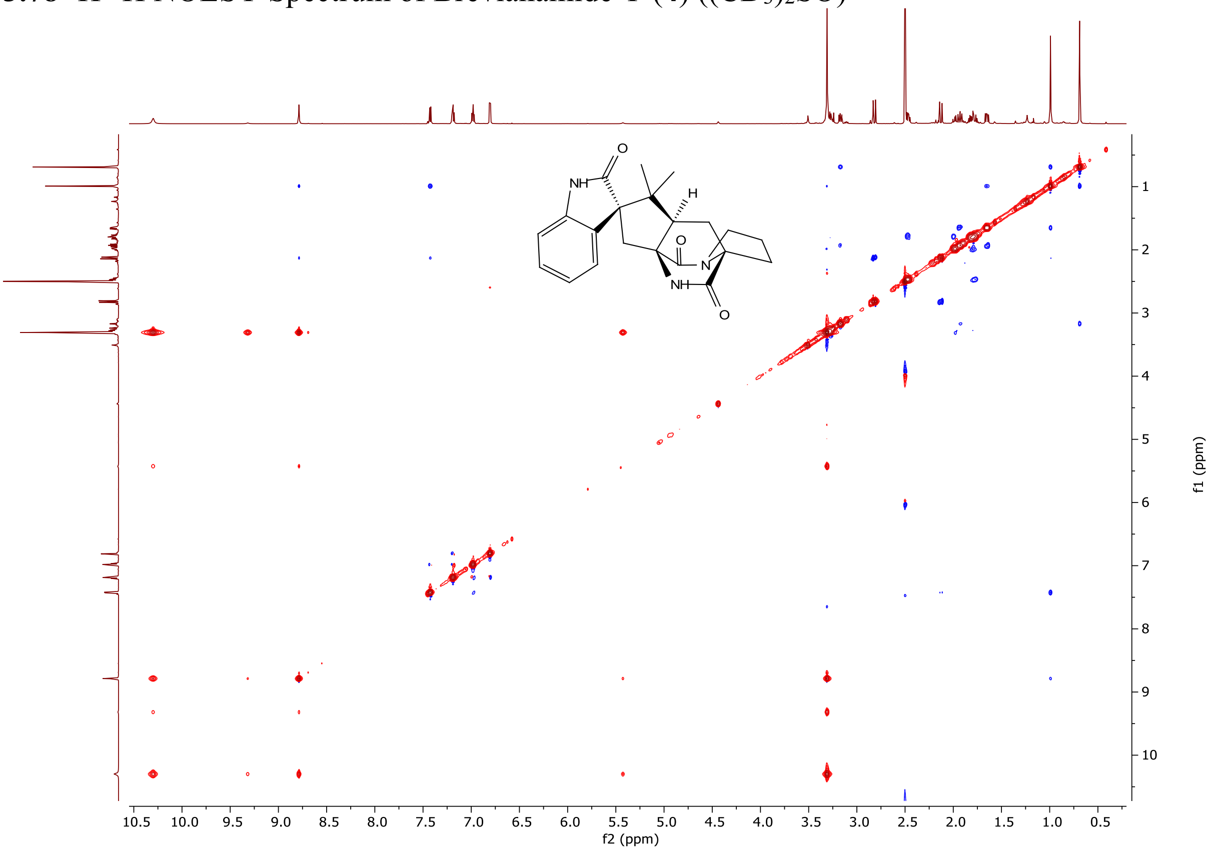

3.79 ^{1}H NMR Spectrum of Brevianamide Z (**5**) (500 MHz, $(CD_3)_2SO$)

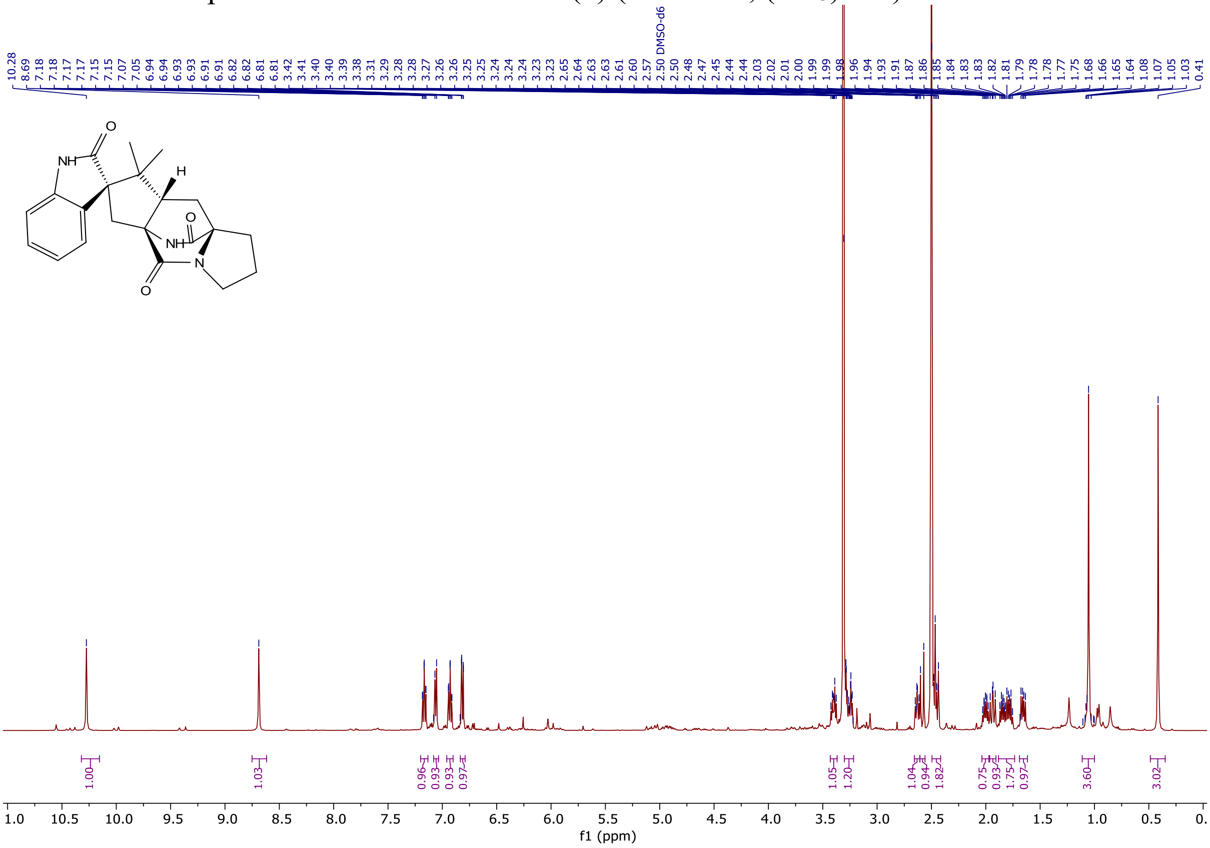

3.80 ^{13}C NMR Spectrum of Brevianamide Z (**5**) (126 MHz, $(CD_3)_2SO$)

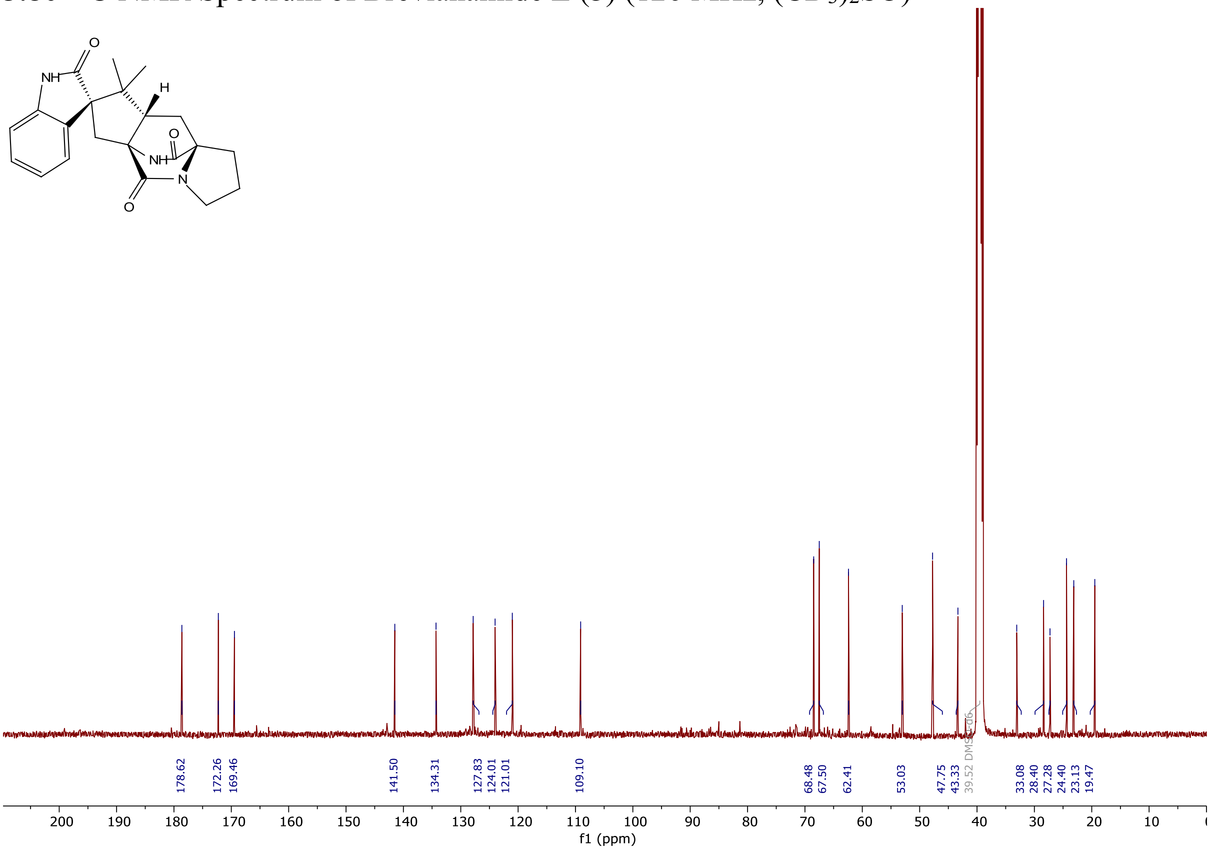

3.81 ^{1}H-^{1}H COSY Spectrum of Brevianamide Z (5) ($(CD_3)_2SO$)

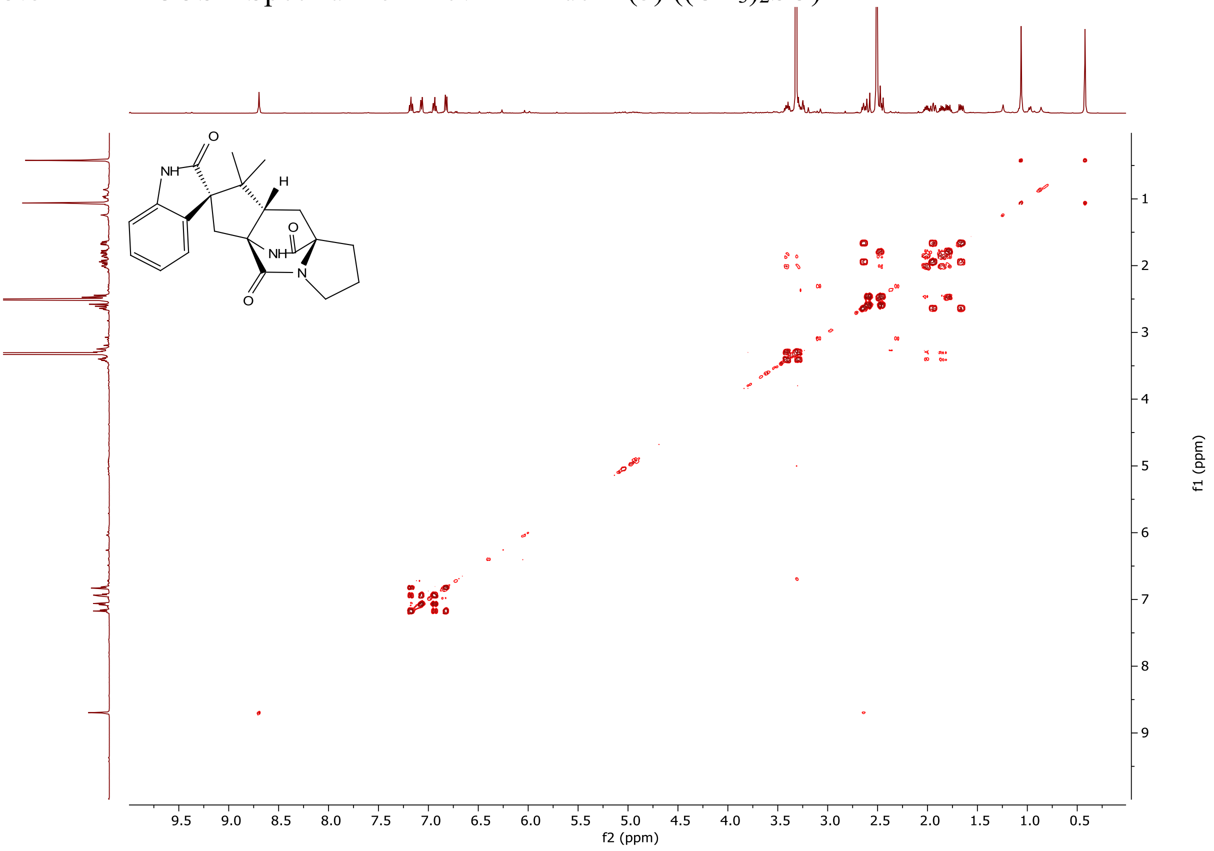

3.82 ^{1}H-^{13}C HSQC Spectrum of Brevianamide Z (5) ($(CD_3)_2SO$)

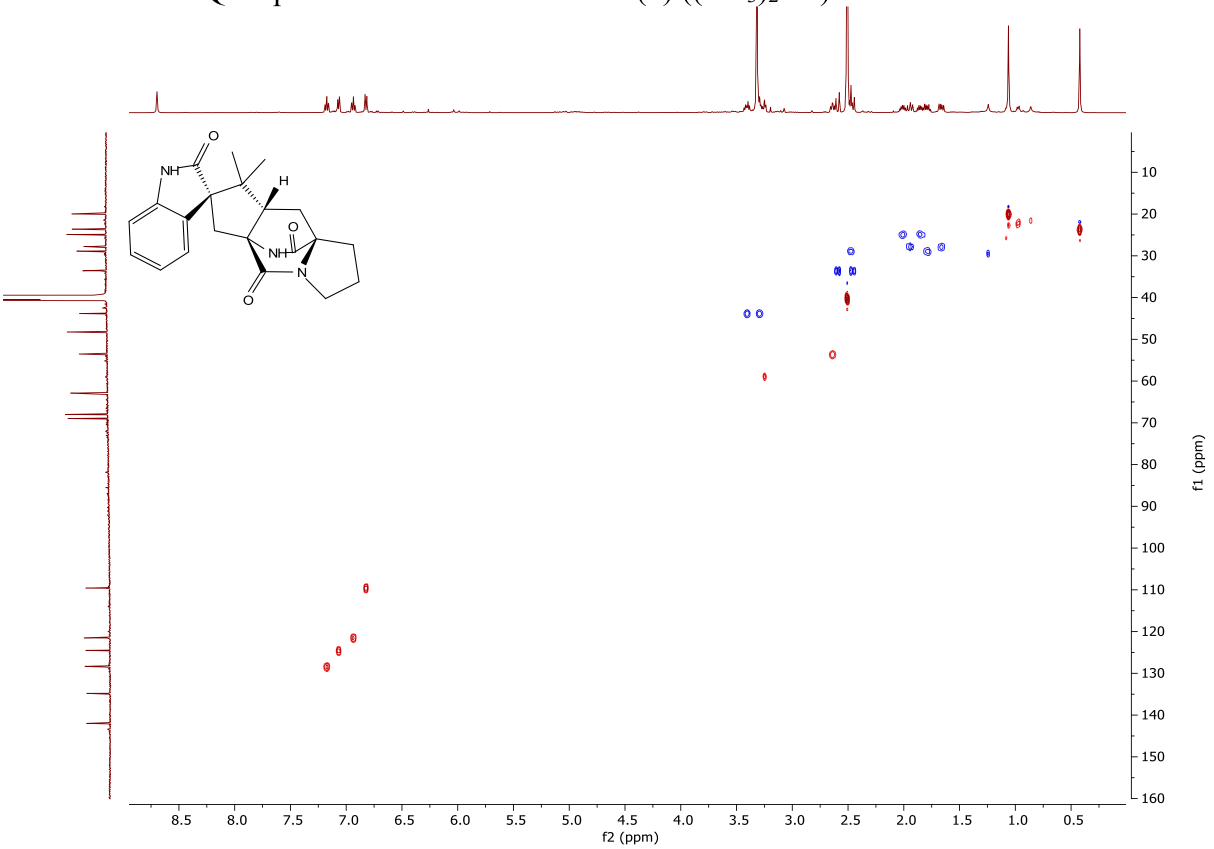

3.83 ^{1}H-^{13}C HMBC Spectrum of Brevianamide Z (**5**) (($CD_3)_2SO$)

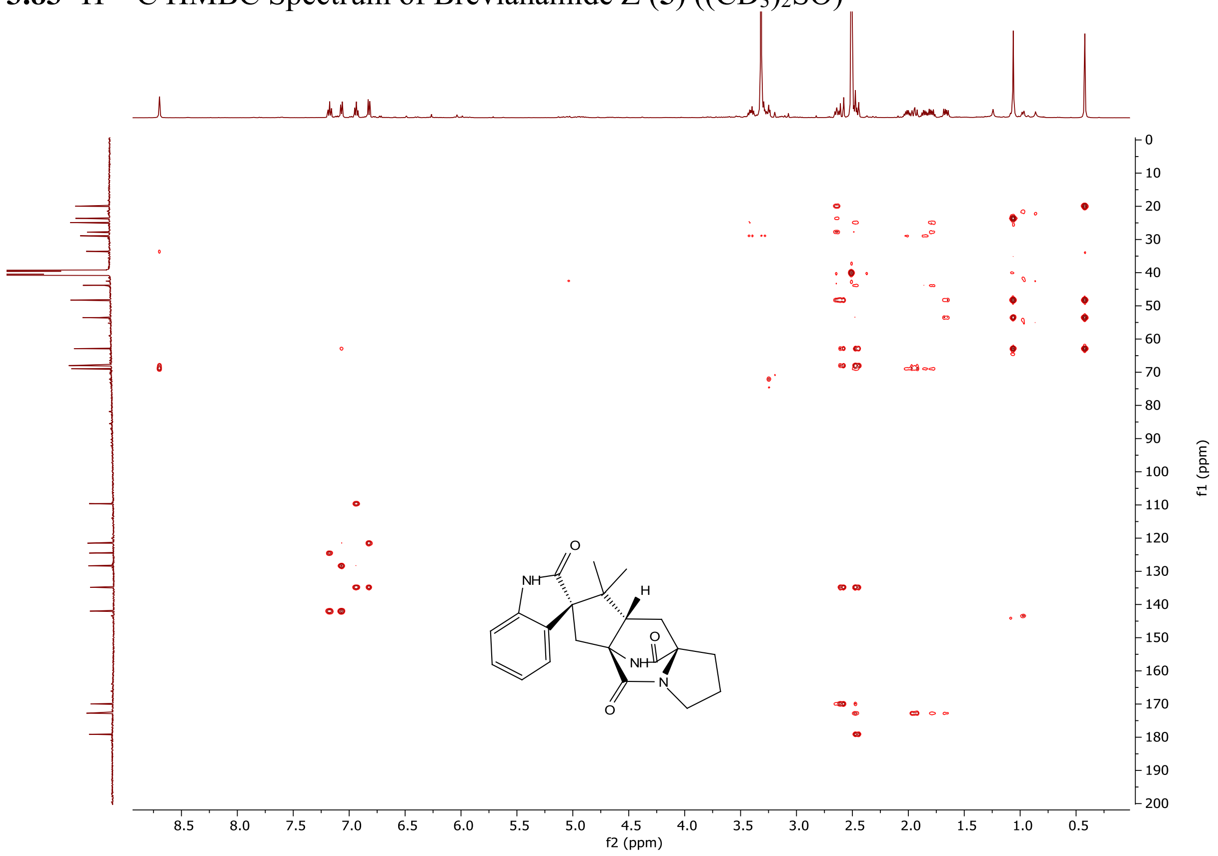

3.84 ^{1}H-^{1}H NOESY Spectrum of Brevianamide Z (**5**) (($CD_3)_2SO$)

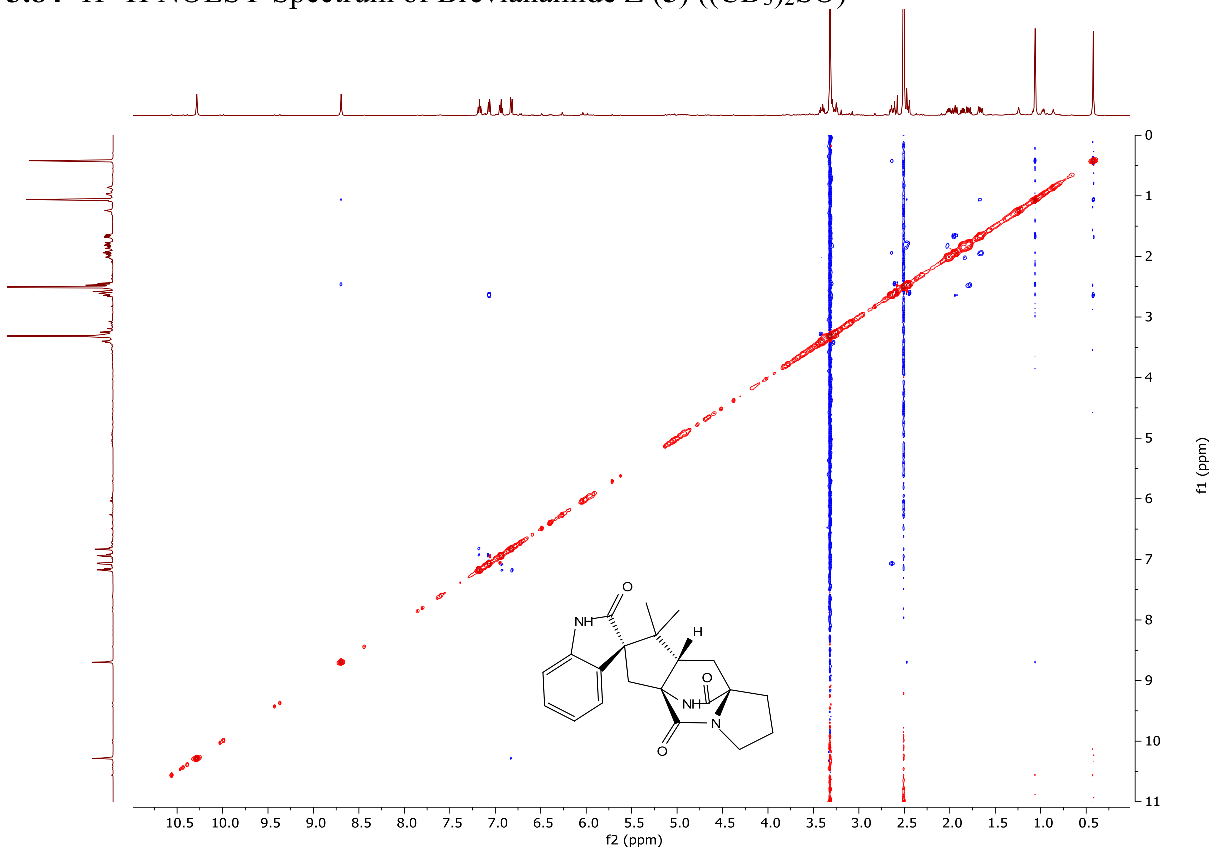

3.85 ^{1}H NMR Spectrum of Compound **(±)-14** (500 MHz, $CDCl_3$)

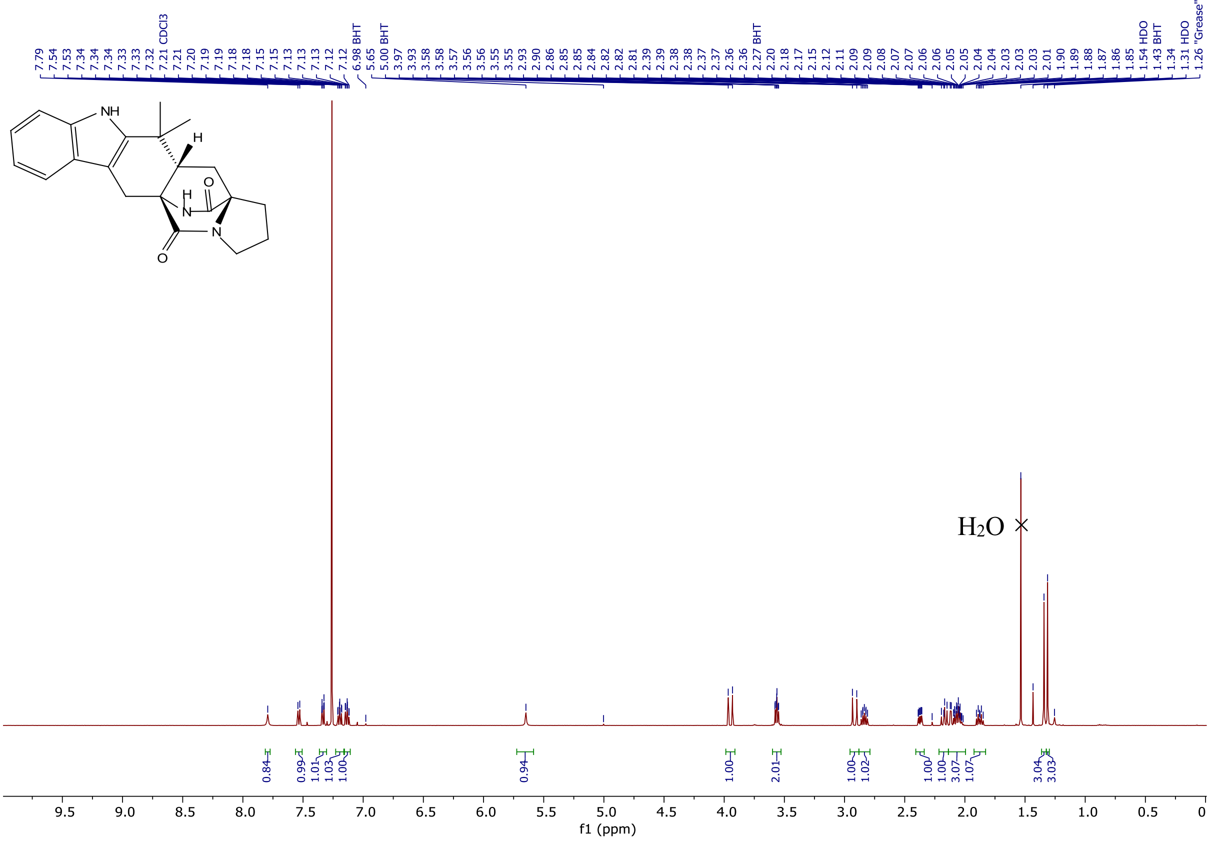

3.86 ^{13}C NMR Spectrum of Compound **(±)-14** (126 MHz, $CDCl_3$)

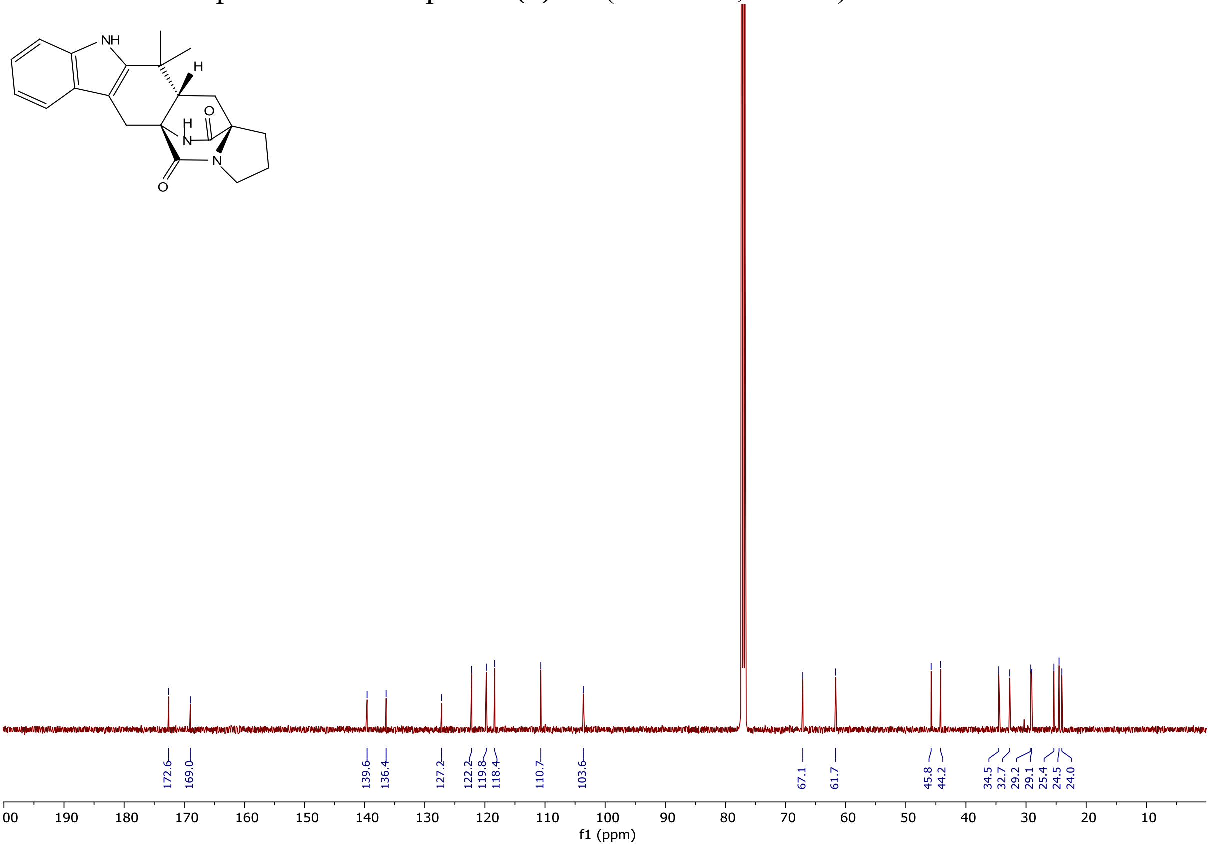

3.87 ^{1}H NMR Spectrum of Compound **(±)-6** (500 MHz, $CDCl_3$)

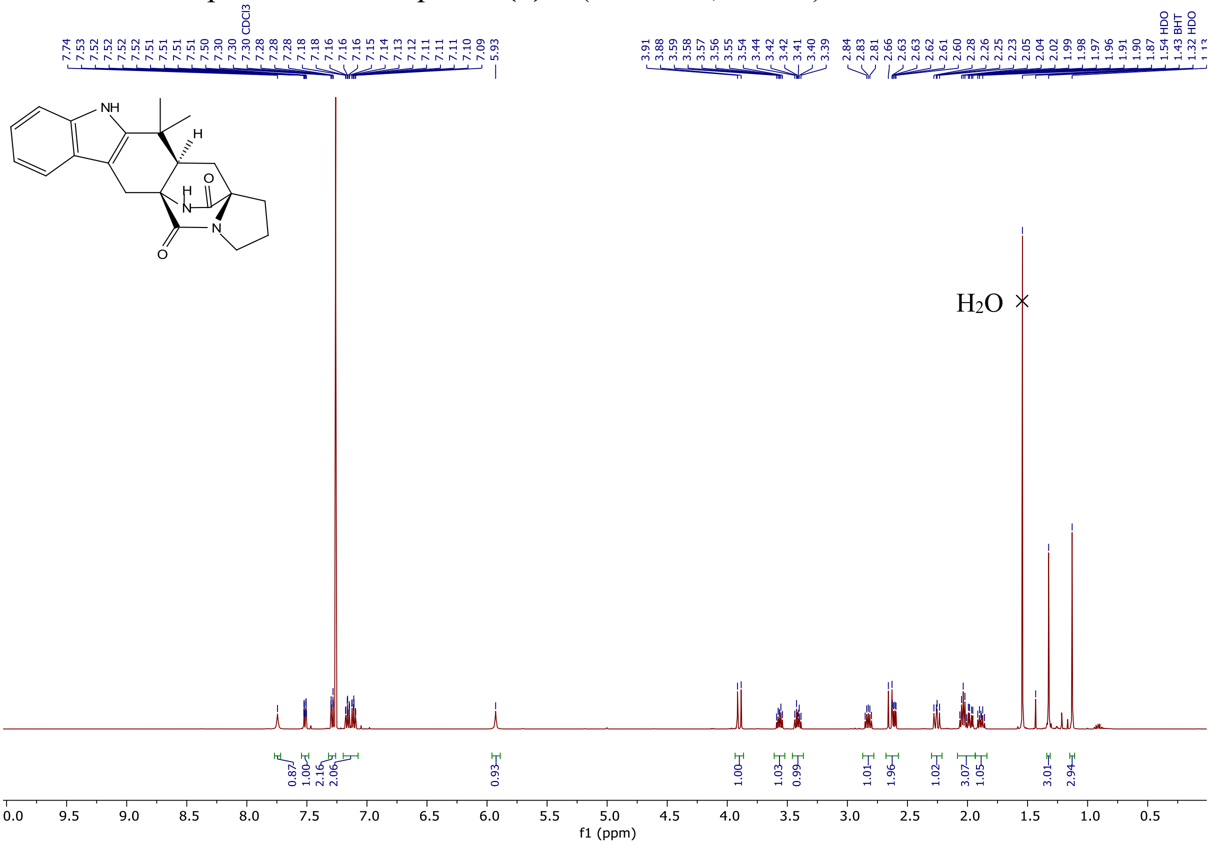

3.88 ^{13}C NMR Spectrum of Compound **(±)-6** (126 MHz, $CDCl_3$)

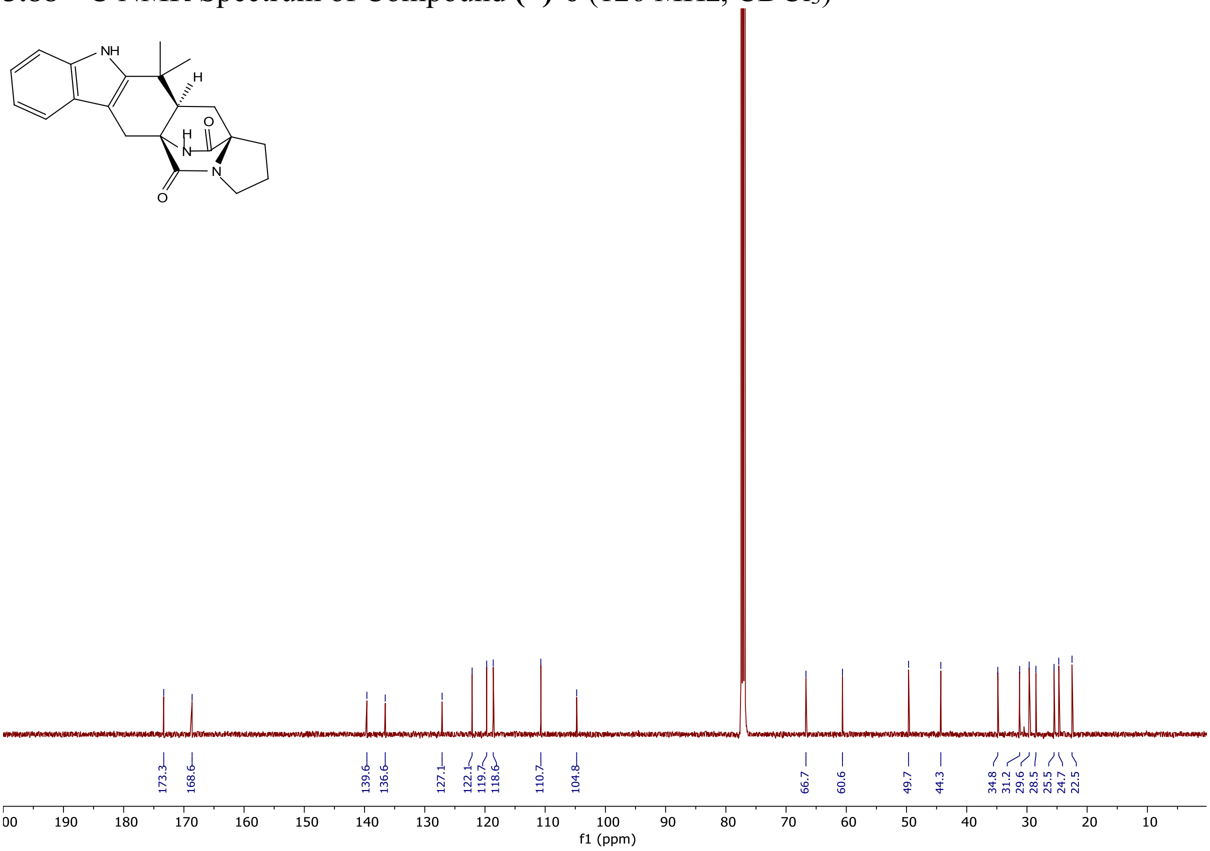

3.89 ^{1}H NMR Spectrum of Brevianamide X (**3**) (500 MHz, $(CD_3)_2SO$)

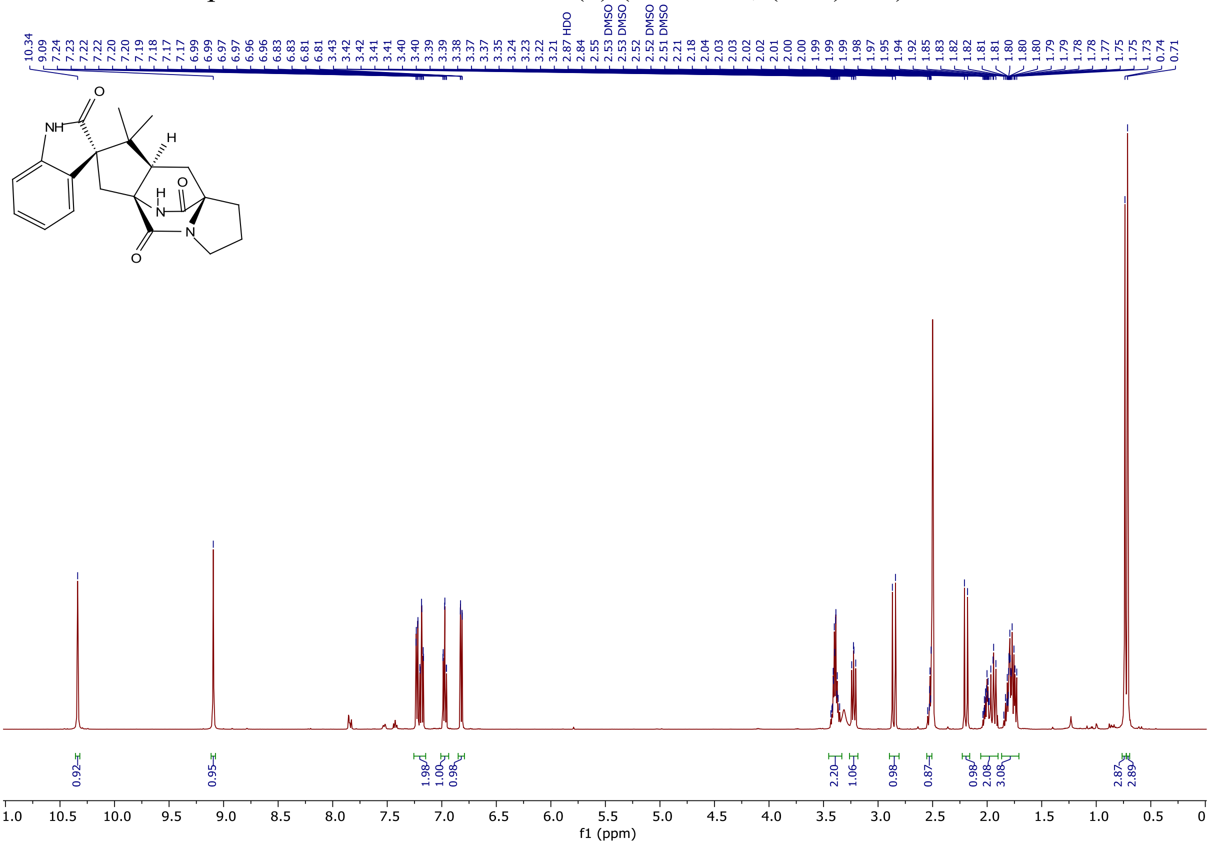

3.90 ^{13}C NMR Spectrum of Brevianamide X (**3**) (126 MHz, $(CD_3)_2SO$)

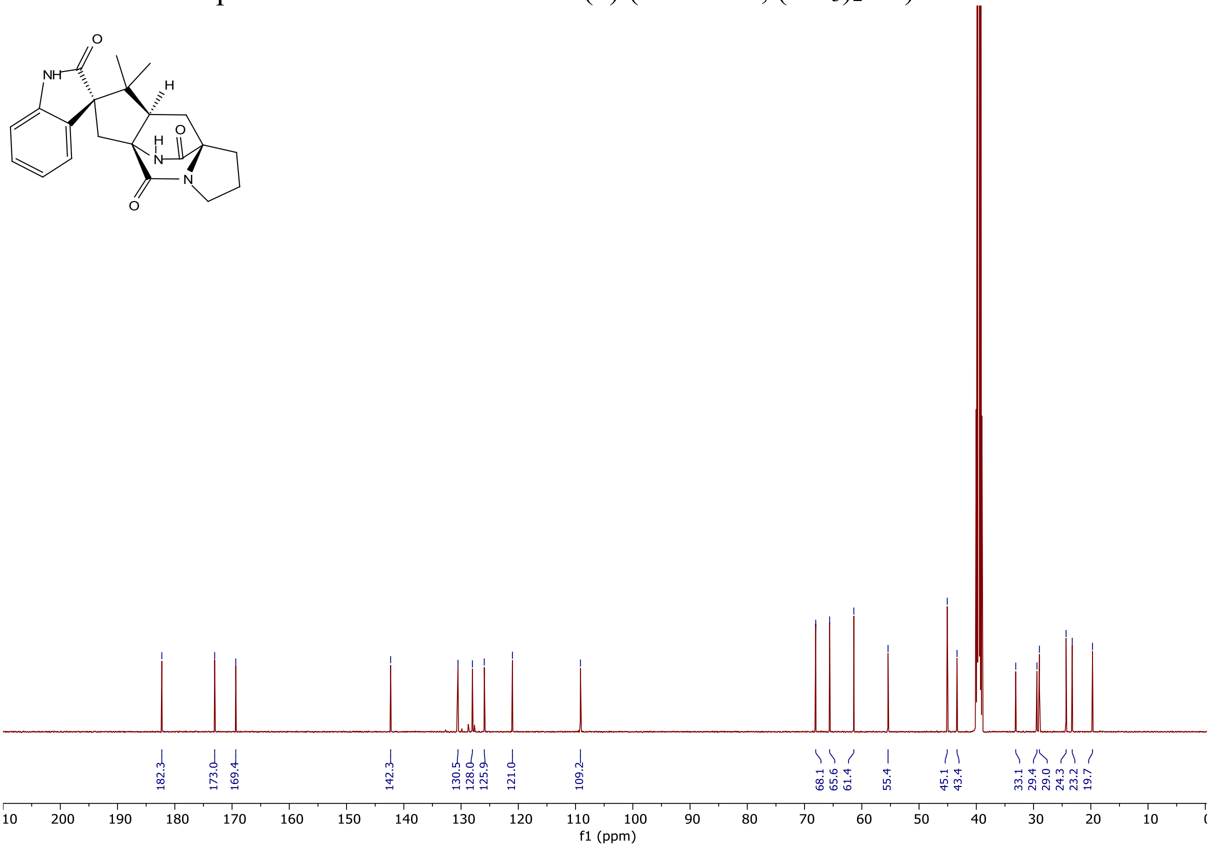

3.91 ^{1}H-^{1}H COSY Spectrum of Brevianamide X (**3**) ($(CD_3)_2SO$)

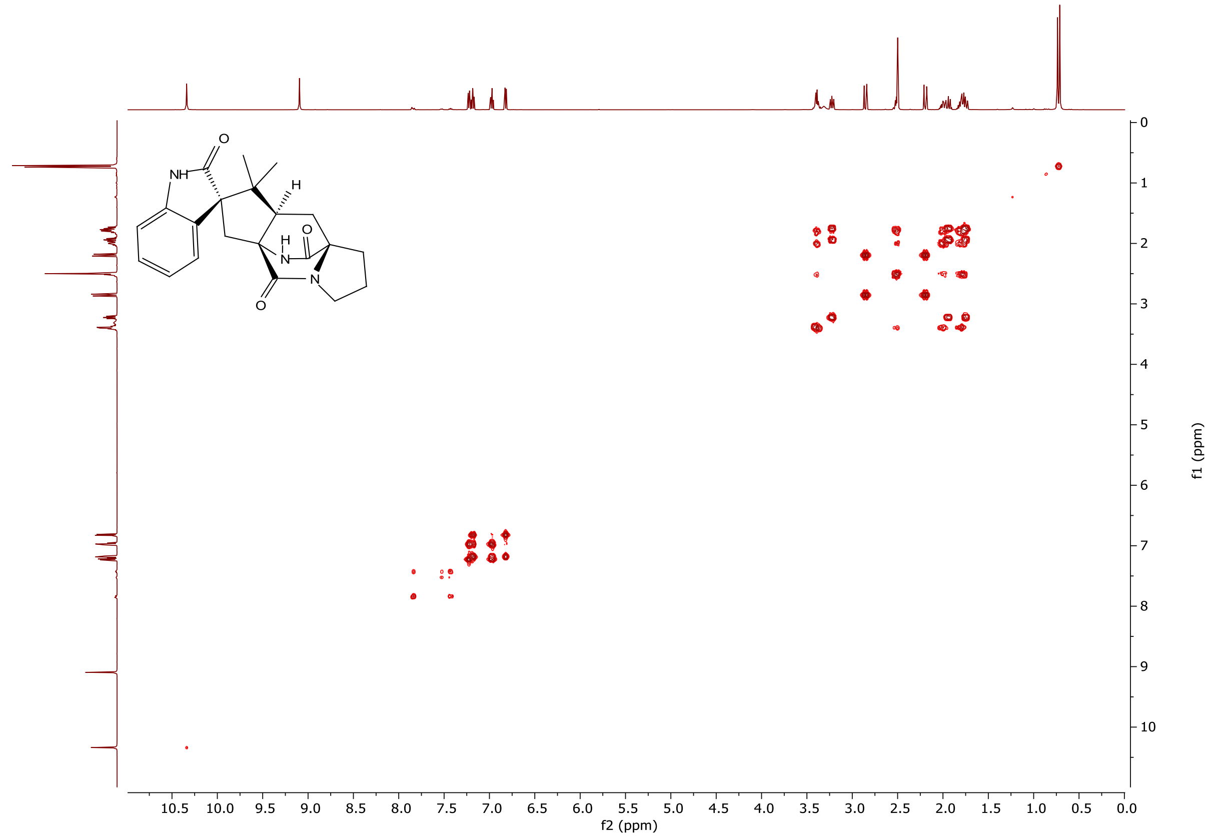

3.92 ^{1}H-^{13}C HSQC Spectrum of Brevianamide X (**3**) ($(CD_3)_2SO$)

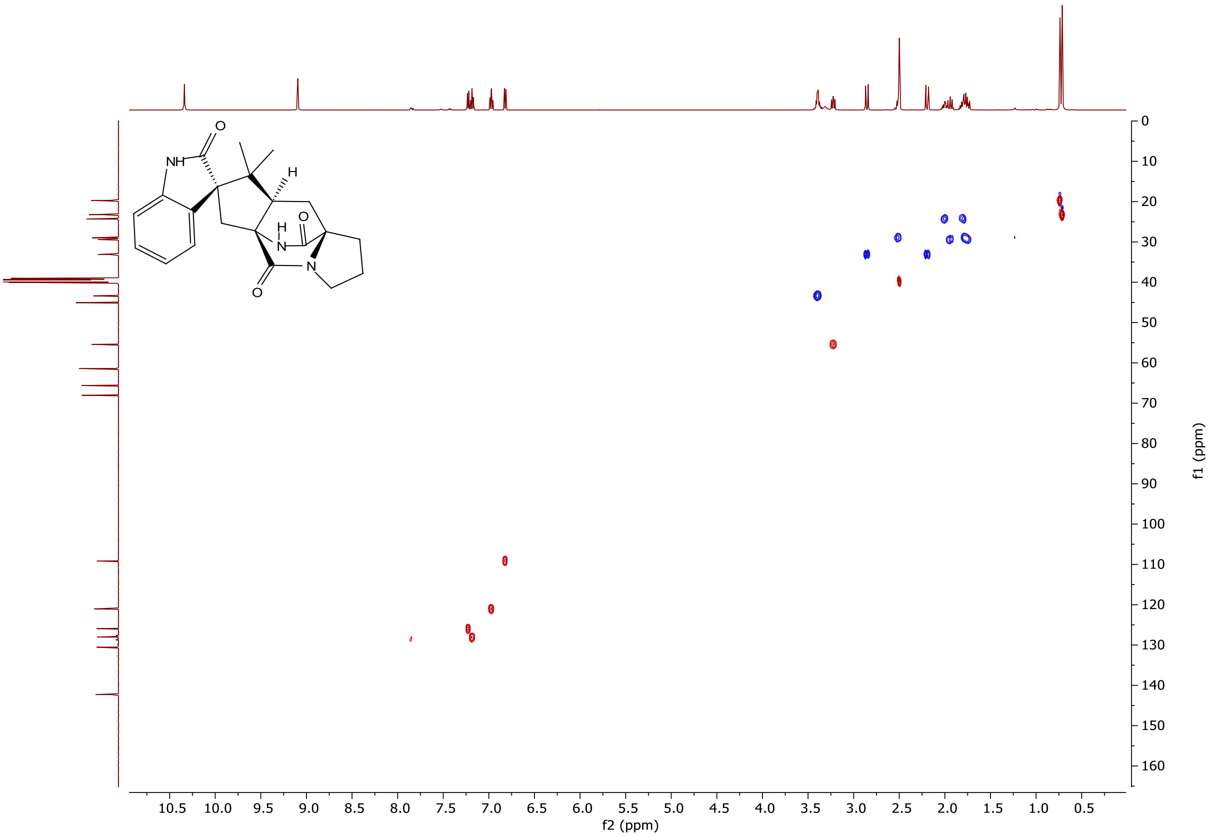

3.93 ^{1}H-^{13}C HMBC Spectrum of Brevianamide X (**3**) ((CD_3)$_2$SO)

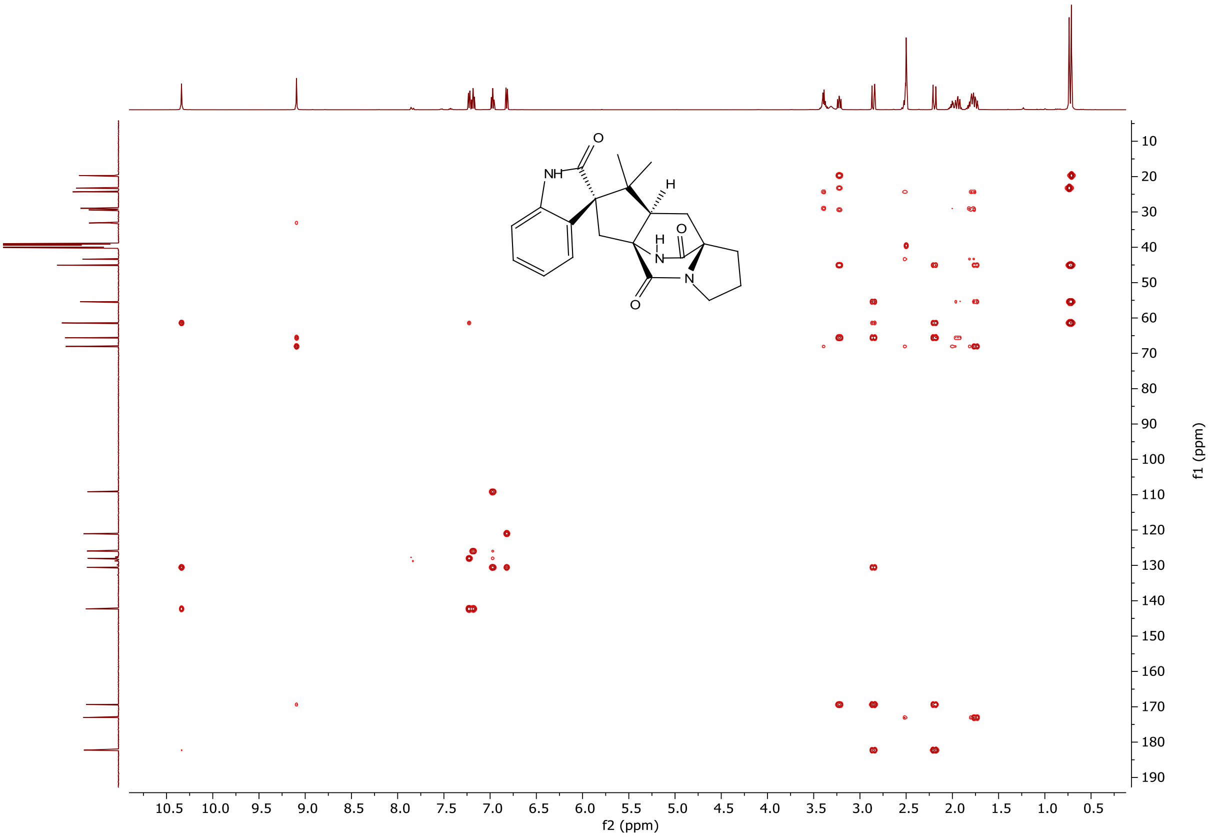

3.94 ^{1}H-^{1}H NOESY Spectrum of Brevianamide X (**3**) ((CD_3)$_2$SO)

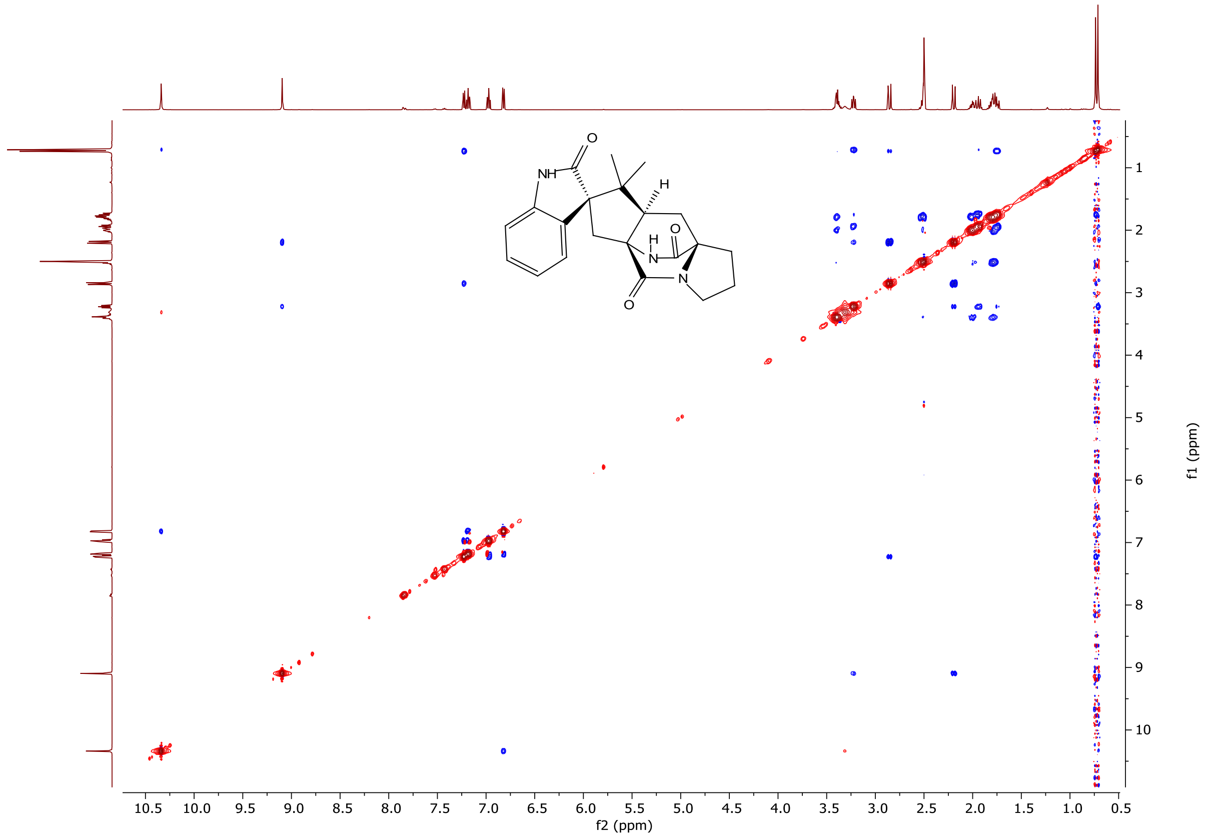

Supplement: SC-013-D1SC05801K-s001 [file SC-013-D1SC05801K-s001.pdf]
